# Localization of the ventricular pacing site from BSPM and standard 12-lead ECG: a comparison study

**DOI:** 10.1038/s41598-023-36768-z

**Published:** 2023-06-14

**Authors:** Ksenia A. Sedova, Peter M. van Dam, Marie Blahova, Lucie Necasova, Josef Kautzner

**Affiliations:** 1 Department of Biomedical Technology, Faculty of Biomedical Engineering, Czech Technical University in Prague, Sitna Sq. 3105, 27201 Kladno, Czech Republic; 2 Department of Cardiology, University Medical Center Utrecht, Utrecht, The Netherlands; 3 Department of Cardiology, Institute for Clinical and Experimental Medicine, Prague, Czech Republic

**Keywords:** Biophysics, Cardiology, Diseases

## Abstract

Inverse ECG imaging methods typically require 32–250 leads to create body surface potential maps (BSPM), limiting their routine clinical use. This study evaluated the accuracy of *PaceView* inverse ECG method to localize the left or right ventricular (LV and RV, respectively) pacing leads using either a 99-lead BSPM or the 12-lead ECG. A 99-lead BSPM was recorded in patients with cardiac resynchronization therapy (CRT) during sinus rhythm and sequential LV/RV pacing. The non-contrast CT was performed to localize precisely both ECG electrodes and CRT leads. From a BSPM, nine signals were selected to obtain the 12-lead ECG. Both BSPM and 12-lead ECG were used to localize the RV and LV lead, and the localization error was calculated. Consecutive patients with dilated cardiomyopathy, previously implanted with a CRT device, were enrolled (n = 19). The localization error for the RV/LV lead was 9.0 [IQR 4.8–13.6] / 7.7 [IQR 0.0–10.3] mm using the 12-lead ECG and 9.1 [IQR 5.4–15.7] / 9.8 [IQR 8.6–13.1] mm for the BSPM. Thus, the noninvasive lead localization using the 12-lead ECG was accurate enough and comparable to 99-lead BSPM, potentially increasing the capability of 12-lead ECG for the optimization of the LV/RV pacing sites during CRT implant or for the most favorable programming.

## Introduction

Cardiac resynchronization therapy (CRT) is an accepted treatment strategy for patients with heart failure with reduced ejection fraction and impaired intraventricular conduction. However, a variable proportion of patients do not improve their clinical status^1^. The optimal pacing lead placement and follow-up monitoring to verify the pacing electrode position have been considered essential determinants of benefit from CRT^2^. Recently, body surface potential mapping (BSPM) and derived inverse ECG imaging methods (ECGI) have been proposed for the optimization of CRT^3,4^. However, BSPM and ECGI are not widely used in clinical practice due to logistic reasons and limited evidence of superiority over standard 12-lead ECG. One of the few commercially available ECGI systems using the standard 12-lead ECG is ViVo^5–8^. This system localizes the ectopic origin of a PVC or VT anywhere in the ventricles, which makes the computation time relative long (minutes). To support patient selection for CRT and guide CRT implants, the system needs only to search the targeted implantation area, right ventricular endocardium, or left epicardium. Recently, we have developed a 12-lead inverse ECG method (iECG) to estimate the endocardial and epicardial ventricular activation^9,10^.

This proof-of-the-concept study aimed to evaluate the accuracy of our novel PaceView iECG method to localize the left or right ventricular (LV and RV, respectively) pacing leads, using either a 99-electrode BSPM or the 12-lead ECG.

## Methods

### Study population

The study recruited outpatients with dilated cardiomyopathy and LBBB, implanted previously with CRT. In most cases, the system consisted of an ICD (12 subjects). Patients were followed in the outpatient clinic. The study was conducted according to the declaration of Helsinki, and all patients gave informed consent. The local ethics committee—*The Ethics Committee of the Institute for Clinical and Experimental Medicine and Thomayer Hospital* approved the study protocol.

All patients underwent BSPM examination using the 99-lead system ProCardio-8^11^. After recording of spontaneous sinus rhythm, the device was programmed to the sequential LV pacing and sequential RV pacing (atrial pacing at 10 bpm above the sinus rate, AV-delay 120 ms).

### ECG data

**Figure 1:**
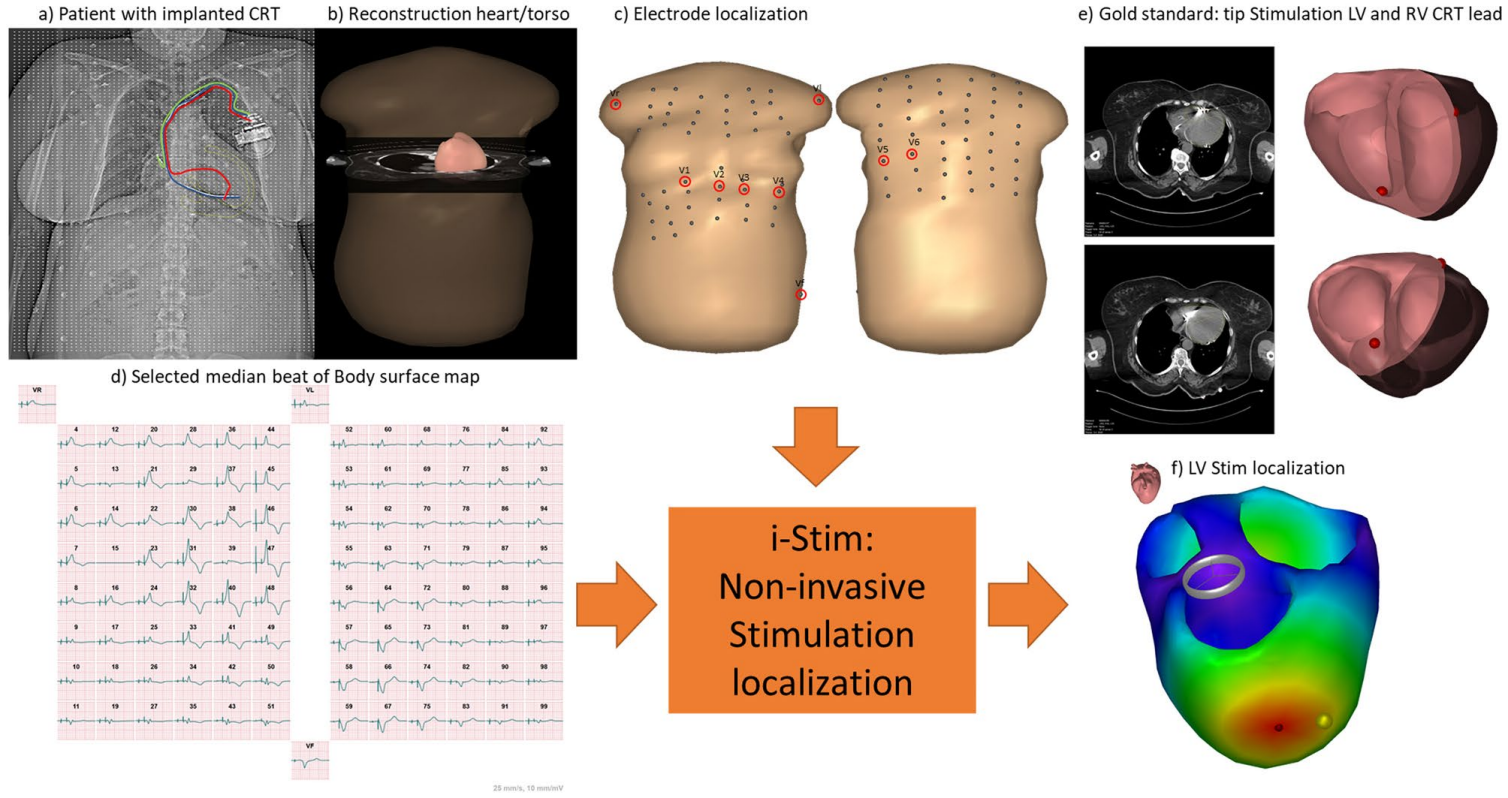
The overview of the used inverse ECG method (i-Stim) to localize right and left ventricular stimulation sites as well as the intrinsic ventricular activation. (**a**) X-ray image of a patient with implanted CRT device, and ECG electrodes attached. (**b**) The CT from which the patient specific model of heart and torso are reconstructed. (**c**) The frontal subset of the 99 body-surface-potential map (BSPM) electrodes. Red circles indicate the used 12-lead ECG positions, where V5/V6 were placed on the back. (**d**) Example of a BSPM with left ventricular (LV) stimulation. (**e**) The used gold standard: the LV and RV CRT lead tip can be localized from the CT, also shown in the heart model as a red dot. (**f**) the output of i-Stim for the localization of the LV stim site (red dot), with the gold standard in yellow.

For each patient, a 99-lead BSPM was recorded in a grid of 12 strips with eight electrodes and three limb electrodes (Fig. 1a,c). All signals were referenced to the Wilson central terminal (Fig. 1d). Each BSPM signal was sampled at 1000 Hz, with a lowpass filter of 250 Hz and no high-pass filter used. A subset of the 99 BSPM electrodes was selected to represent the 12-lead ECG, indicated by red circles in Fig. 1c.

### Imaging and model reconstruction

Patients with all ECG leads in situ underwent non-contrast CT imaging of the chest (Somatom Definition Flash, Siemens, Erlangen, Germany) to derive a patient-specific model of the heart and torso (Fig. 1a–c). ECG electrode positions on the chest as well as the models of ventricles and torso, were reconstructed with GeomPeacs (Peacs BV, the Netherlands)^12^. The same CT scans were also used to localize the implanted CRT LV/RV leads (Fig. 1e,f). The non-contrast CT did not allow for accurate localization of the septum. The GeomPeacs software, however, uses template heart models with a septum to fit the CT. After global positing, the heart was adapted to visible heart contours. The RV pacing lead was used to confirm the estimated location of the septum^12^.

### Estimation of the cardiac activation

To simulate ECGs from an activation sequence, a volume conductor model and a cardiac source model is needed. For the latter, the equivalent double-layer (EDL) model was used^13–16^. In addition, the Boundary element method was used to compute the BSPM signals^17,18^. Assigned conductivity values were 0.2 S/m for the thorax and ventricular muscles, and 0.6 S/m for the blood cavities. Using the EDL source model results in a non-linear, ill-posed inverse problem, requiring an initial estimate to estimate the activation sequence. This method uses the fastest route algorithm^19^ to create the most likely activation sequence for the measured ECG signals. Different ways have been tested to obtain the most likely activation sequence, a multi-focal approach where the activation sequence was iteratively changed by adding more initiating foci^19,20^, and one matching the mean activation isochrone positions with the vectorcardiogram (VCG)-derived position^5^. These methods allowed the origin of cardiac activation to occur anywhere in the heart (Fig. 1f). For both intrinsic and paced rhythm, the inverse procedure has been optimized.

### Estimation of the intrinsic activation

**Figure 2:**
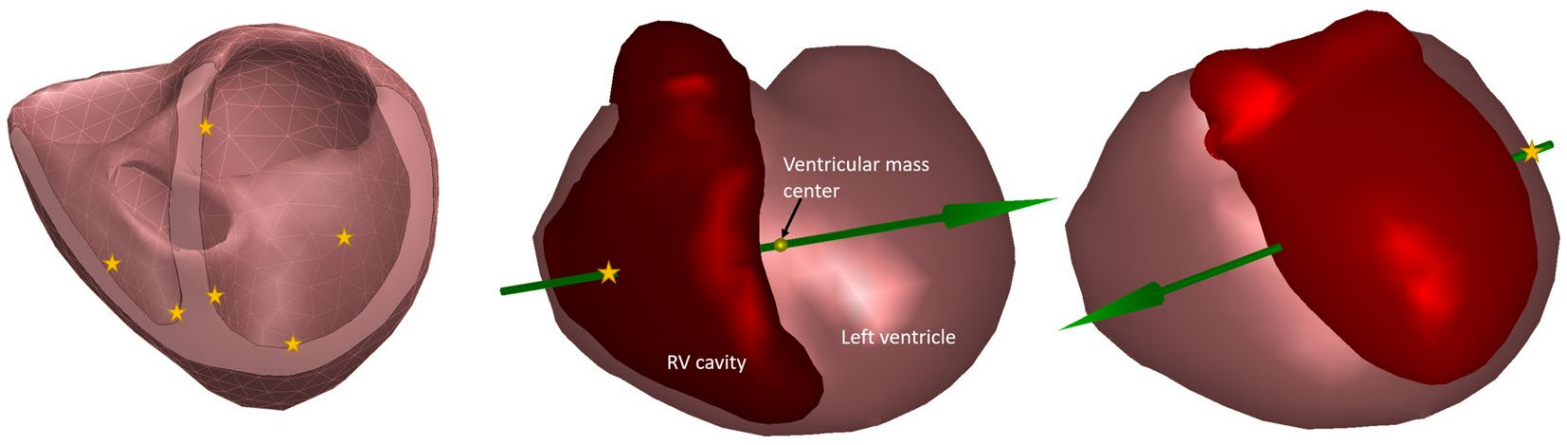
Activation sites for the initial activation. In the left panel the sites identified as potential His-Purkinje excitation sites. The right panels show the right and left ventricular activation sites as identified by the QRS axis. The right ventricular activation site can be either located on the right septal wall (not visible) or on the right free wall.

The non-invasive estimation of intrinsic His-Purkinje cardiac activation uses the method described by Boonstra et al.^21^. In brief, the His-Purkinje system is modeled as a combination of multiple activation wavefronts originating from areas associated with the early break-troughs of the ventricular activation (Fig. 2). The initial position of the discrete nodes on the ventricular myocardium representing the His-Purkinje system is initialized based on electrophysiological knowledge of the normal cardiac activation^22,23^. For normal activations (QRS duration < 110 ms), the initial activation times of the left septal wall were set to 0 ms, i.e., equal to the QRS onset, while the RV and LV activations times were set to 15 ms after the indicated QRS onset. For ECGs with an LBBB pattern (QRS duration >  = 120 ms) the initial timing of the left regions is delayed to 40 ms for the septal regions and 45 ms for the free wall regions. Similarly, for RBBB ECG waveforms (QRS duration > 120 ms), the timing of the RV septal region is set to 45 ms, and the RV free wall to 65 ms.

### Localization of the RV and LV pacing site

The subsequent estimation procedure to localize the left or right pacing site is a two-step process. In the first step, the initial position of the stimulation site is determined. To limit the search space to the initial site of activation, the RV pacing search area was restricted around the two QRS axis RV crossing points in the right chamber or the entrance point at the left epicardium (see Fig. 2 for an LV example). The QRS axis was determined by the average direction of the normalized VCG, where the QRS axis is localized to the center of ventricular mass^24^. In the second step, activation sequences from the identified search area were computed. The fastest route algorithm used realistic myocardial propagation velocity (0.7–0.85 m/s), to compute activation times between the discrete nodes on the closed triangulated modelled myocardial surface mesh^19^. In this algorithm, the anisotropic nature of the myocardial tissue was captured by a 2.5 times slower transmural velocity than the velocity over the ventricular surface. Using the fastest route algorithm, QRS waveforms were simulated starting from nodes in the identified search area. The node resulting in the simulated QRS duration that best matched the recorded QRS duration was selected to be the initial activation estimate for initial stimulation site. In the subsequent step, the option of fusion activation through the AV-node is added to the initial activation estimate. For this purpose, additional His-Purkinje-related activation sites are added to the chamber not being stimulated, i.e., in the case of the LV stimulation site, two extra foci are added in the RV, one in the lower right septum, and one associated with the moderator band on the right free wall^25^. For RV stimulation sites, three extra foci are added in the LV; left mid septum, one on the anterior mid-wall, and one on the lower left posterior wall. These latter two locations are associated with the papillary muscles and early activation from the His-Purkinje system^22^.

### Improvement of the initial estimate and optimization procedure

In the current approach, the His-Purkinje-related anatomical structures could not be identified due to the limitations of the used CT images. Also, estimating the stimulation sites could improve by increasing the match between simulated measures and simulated ECG signals. Therefore, in a subsequent iterative improvement step, the position and timing of all identified foci are changed to optimize the correlation between simulated and measured ECG signals. The result of this improvement step is referred to as the *initial* estimate and is used in the final optimization step. In a final optimization mathematical optimization procedure, the initial activation sequence was optimized with a regularized optimization procedure, using the surface Laplacian as a regularization operator^18^ and a fixed value of 0.0002 as the regularization parameter µ. This regularized optimization procedure adapts the local activation times to better match the simulated and measured ECG waveforms^18,21^ while guarding the spatial smoothness of the local activation times.

### BSPM vs. 12-lead ECG

All intrinsic and pacing ECGs were used to compare the results obtained from the 12-lead ECG and the 99-lead BSPM, where the 12-lead ECG was a subset of the BSPM (see Fig. 1c). The pacing site was determined from the pacemaker lead location derived from the CT and the earliest node activated on the model myocardium (see Appendix). The geodesic distance, i.e., over the surface of the heart model (Fig. 2), was used as the error measure. As the used heart models can only be activated from one of these discrete points, the geodesic distance was determined from the node closest to the indicated lead position.

The intrinsic activation sequences from 12-lead ECG and BSPM were compared by computing the correlation between both estimated activation sequences. Additionally, three values were computed as a measure of global depolarization consistency:

- VEU (Ventricular Electrical Uncoupling) was defined as the difference between the mean activation time of the left free wall and the mean activation time of the right free wall;
- TAT (Total Activation Time) equaled to the latest activated node of the ventricular model i.e., maximal local activation time through ventricles.
- LVAT (Left Ventricular Activation Time) was defined as the maximum difference in activation time of the whole left ventricle, including activation times from the epicardium and endocardium, left free wall and left ventricular septum.

### Statistical analysis

Reliability analysis with Intraclass Correlation Coefficient (ICC, Two-way mixed model, Absolute Agreement type) was performed to assess the similarity of ventricular activation sequences reconstructed from 12-lead ECG and BSPM. ICC was also applied to evaluate the consistency measures derived from initial and final inverse estimated activation sequences.

## Results

### Patient population

The clinical characteristics of 19 patients (58% male) studied are summarized in Table 1. The patients were 60 ± 11 years old with a QRS duration 167 ± 16 ms and LVEF 26 ± 6% prior to CRT implant. All were in sinus rhythm with true LBBB pattern on ECG.

**Table 1:** Patient demographics and clinical characteristics.

Characteristic	Value (n = 19)
Age, years	60 ± 11
Male gender	11 (58%)
NYHA class II, %	10 (53%)
NYHA class III, %	9 (47%)
Pre-CRT QRS duration, ms	167 ± 16
Systolic pressure, mm Hg	123 ± 17
Diastolic pressure, mm Hg	76 ± 10
Pre-CRT LV EF, %	25 (IQR 10)
Diabetes	3 (16%)
Hypertension	9 (47%)
B-blockers	17 (90%)
ACEI/ARB	19 (100%)

Values are presented as mean ± standard deviation, or median and interquartile range (IQR) if asymmetric distribution. Categorical variables are presented as frequencies and percentages.

*NYHA* New York Heart Association, *LBBB* Left bundle branch block, *RBBB* Right bundle branch block, *LV* Left ventricular, *EF* Ejection fraction, *ACEI/ARB* Angiotensin converting enzyme inhibitor or angiotensin II receptor blocker.

### Pacing site localization

**Figure 3:**
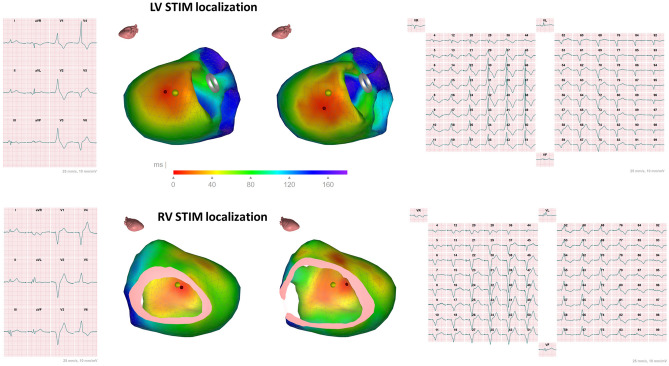
Example of the localized RV and LV lead of patient 4. A geodesic error of 0 mm means that the identified lead location was on the triangle to which the CT derived lead location was projected. For instance, see initial estimate RV stim location. The dark red point is the vertex from which the simulated activation started; the yellow dot is the gold standard lead location derived from CT.

The median geodesic distance from the LV pacing site localized by the initial inverse solution to the CT-determined lead position was 8.3 [IQR 0.0–10.3] mm for the 12-lead ECG and 9.7 [IQR 7.7–13.1] mm for BSPM. After final optimization, the LV lead was localized with a median error of 7.7 [IQR 0.0–10.3] mm and 9.8 [IQR 8.6–13.1] mm for 12-lead ECG and BSPM, respectively. The localization error of the RV pacing lead estimated from 12-lead ECG or BSPM demonstrated an error of 9.0 [IQR 0.0–13.4] mm and 8.4 [IQR 3.0–14.2] mm, respectively, after initial estimation. The final localization error for RV lead position was 9.0 [IQR 4.8–13.6] mm for the 12-lead ECG and 9.1 [IQR 5.4–15.7] mm for the BSPM (Table 2, Figs. 3 and 5a,b). The optimization step improved the correlation between the measured ECG and the initial activation-derived ECG, and the measured ECG and the optimized activation-derived ECG signals significantly, from 40–60% initial to 95–98% (Table 2).

**Table 2:** Localization error of the right and left ventricular lead from either the 12-lead ECG or a 99-lead body surface potential map.

Median [Intra quartile range] (min max)		LV 12-lead ECG	LV BSPM	RV 12-lead ECG	RV BSPM
Geodesic Localization error	Initial	8.3 [0.0–10.3] (0.0–16.5)	9.7 [7.7–13.1] (0.0–16.9)	9.0 [0.0–13.4] (0.0–18.5)	8.4 [3.0–14.2] (0.0–18.2)
	Final	7.7 [0.0–10.3] (0.0–16.5)	9.8 [8.6–13.1] (0.0–16.9)	9.0 [4.8–13.6] (0.0–29.1)	9.1 [5.4–15.7] (0.0–31.1)
Correlation	Initial	48.9 [− 7.6–62.4] (− 100–84.3)	54.4 [31.0–66.8] (− 7.1–77.9)	61.0 [53.4–72.4] (6.5–84.8)	40.6 [27.2–48.3] (− 15.2–55.8)
	final	96.4 [91.4–98.0] (47.9–98.5)	95.1 [95.1–96.7] (80.1–97.8)	97.7 [97.4–97.9] (96.1–98.7)	97.1 [95.4–97.4] (74.5–97.8)

Values are presented as median, interquartile range [IQR], and minimal/maximal values (min—max).

*BSPM* Body surface potential map, *LV* Left ventricular, *RV* Right ventricle.

Absolute agreement in pacing site localization was found between initial and optimized inverse solutions. For LV pacing lead localization intraclass correlation coefficient (ICC) was 0.99 (95% CI 0.98–0.99; *p* < 0.0001) for 12-lead ECG, and 0.97 (95% CI 0.93–0.99; *p* < 0.0001) for BSPM, while for RV pacing site localization correlation between initial and optimized methods was a little lower. ICC was 0.84 (95% CI 0.59–0.94; *p* < 0.0001) for 12-lead ECG and 0.77 (95% CI 0.39–0.91; *p* = 0.001) for BSPM.

### Intrinsic and paced depolarization sequence

**Figure 4:**
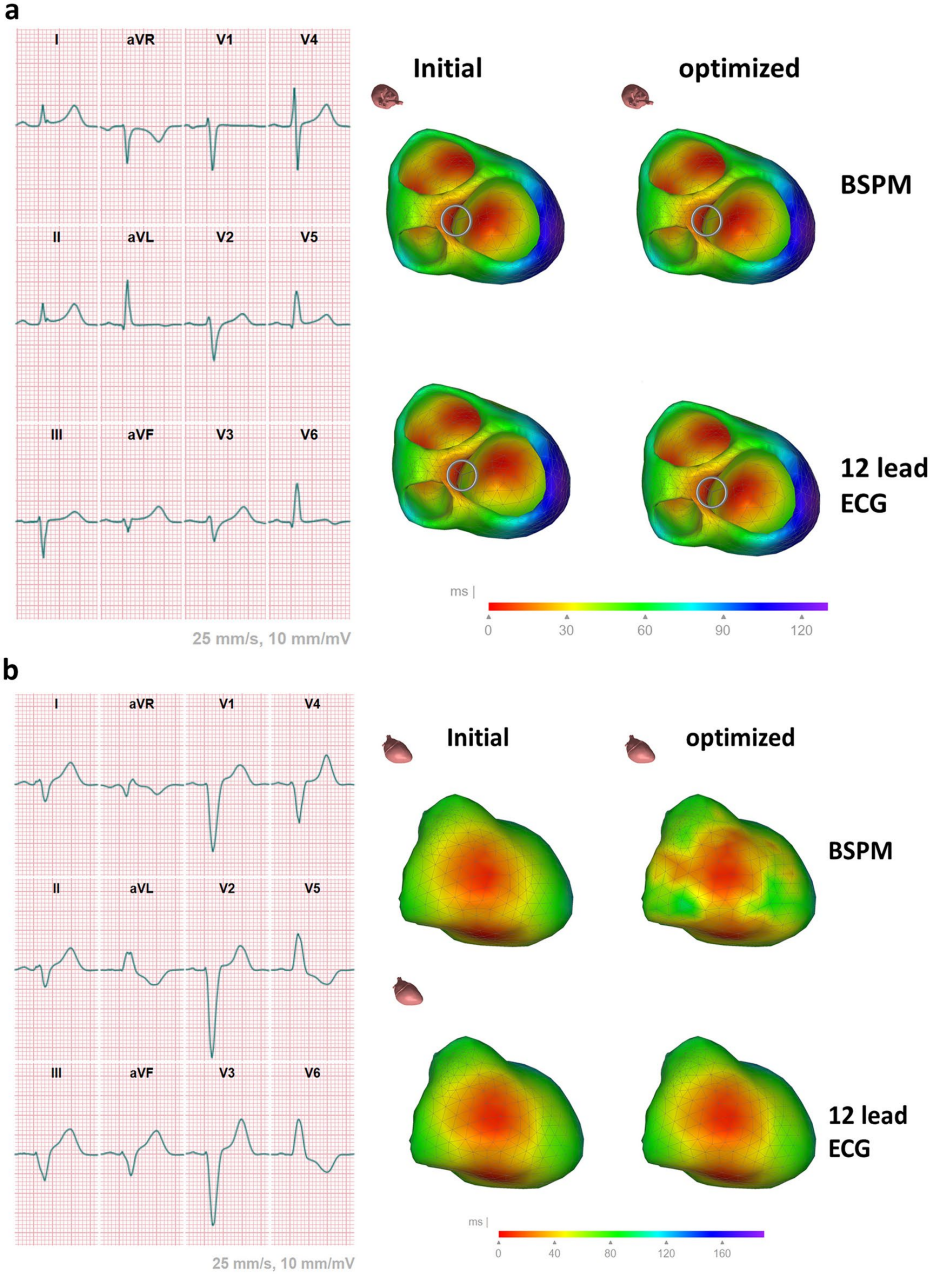
comparison between 12-lead ECG derived activation and BSPM of patient 1 and 7. Patient 1 shows early activation on the left septum with late activation on the left lateral wall, whereas patient 7 shows a typical left bundle block pattern with only initial activation on the right free wall. The optimization of the activation sequence changed limited when the 12-lead ECG was used, slightly more changes were found in the estimations from BSPMs.

**Figure 5:**
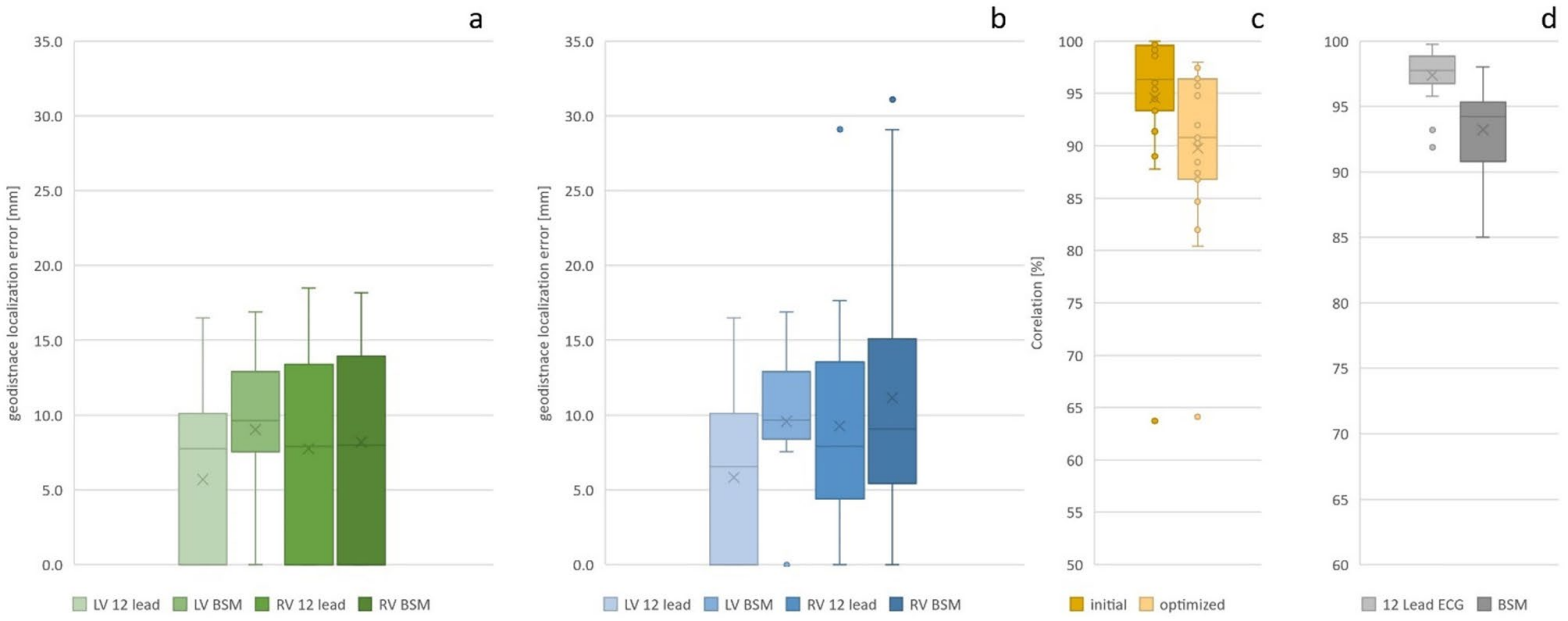
Localization errors and correlations between different localizations. (**Panel a**) Geodesic localization error for the initial (green) and optimized (blue) activation sequence (**panel b**). A geodesic error of 0 mm means that the identified lead location was on the triangle to which the CT derived lead location was projected. (**Panel c**) shows the correlation for the normal sinus rhythm activation maps derived from the 12-lead ECG and the BSPM for the initial sequence (dark yellow) and after the final optimization (light yellow). In (**Panel d**) the correlation of the activation sequence before and after optimization in case the 12-lead ECG was used (light gray) and when the BSPM was used (dark gray). The found correlations were in most cases well above 90% , i.e. the initial and optimized map were similar.

For the intrinsic ECGs, the activation sequences derived from the 12-lead ECG and the BSPM were similar, both when comparing the initial sequences (median ICC 0.98, *p* < 0.0001) as well as the optimized ones (median ICC 0.95, *p* < 0.0001), see Fig. 4 for an example. The correlation between the initial and optimized activation sequences derived from both the 12-lead ECG and BSPM are shown in Fig. 5c. The median correlation was initially 96.3% (range 64–100%) and after optimization 91% (range 64–98%). The optimization did not change the activation sequence much; the correlation between the activation sequences was well above 90% for all patients (Fig. 5d). Using the BSPM, the correlation changed slightly with a minimum of 85% between the initial and optimized activation sequence.

During sequential ventricular pacing, the final activation sequences reconstructed from 12-lead ECG and BSPM were similar, indicating a high degree of agreement with median ICC 0.94 (*p* < 0.0001) for RV and median ICC 0.89 (*p* < 0.0001) for LV pacing modes.

Myocardial parameters of electrical dyssynchrony, such as total activation time of ventricles (TAT), left ventricular activation time (LVAT), and the ventricular electrical uncoupling (VEU) were calculated based on reconstructed activation maps to obtain the quantitative metrics of ventricular depolarization. For the intrinsic rhythm and during sequential LV or RV pacing, TAT, LVAT and VEU derived from the 12-lead ECG were significantly similar with ones obtained from BSPM (Table 3), confirming the efficacy of 12-lead imaging approach for myocardial electrical function assessment.

**Table 3:** Similarity of ventricular depolarization metrics measured from 99-lead BSPM and 12-lead ECG.

Parameter	12-lead ECG	BSPM	ICC	95% CI	*P* value
Intrinsic rhythm					
TAT, ms	169 ± 23	166 ± 24	0.94	0.84–0.98	< 0.0001
LVAT, ms	144 ± 20	143 ± 19	0.93	0.81–0.97	< 0.0001
VEU, ms	48 ± 12	42 ± 17	0.83	0.52–0.94	< 0.0001
LV pacing					
TAT, ms	179 ± 26	180 ± 29	0.84	0.58–0.94	< 0.0001
LVAT, ms	156 ± 21	153 ± 21	0.83	0.58–0.95	< 0.0001
VEU, ms	-50 ± 32	-39 ± 35	0.85	0.60–0.94	< 0.0001
RV pacing					
TAT, ms	177 ± 24	179 ± 21	0.95	0.86–0.98	< 0.0001
LVAT, ms	143 ± 24	140 ± 23	0.92	0.78–0.97	< 0.0001
VEU, ms	31 ± 16	30 ± 16	0.94	0.84–0.98	< 0.0001

Data are presented as mean ± standard deviation.

*BSPM* Body surface potential map, *ICC* Interclass correlation coefficient, *CI* Confidence interval, *TAT* Total activation time, *LVAT* Left ventricular activation time, *VEU* Ventricular electrical uncoupling.

## Discussion

The main results of this study can be described as follows: 1) the novel noninvasive inverse ECG method, PaceView, is able to estimate ventricular activation sequence from different number of ECG leads, either 99-lead BSPM or the standard 12-lead ECG, 2) the accuracy of PaceView was determined by localizing the pacing lead in patients with implanted CRT devices, either to the right ventricular cavity or the left ventricular epicardium—the typical CRT lead implant sites, 3) the PaceView algorithm showed high agreement between the results obtained from 99-lead BSPM or the 12-lead ECG, 4) for paced rhythm, the localization errors were comparable (median localization error less than 10 mm), while for the intrinsic activation, a correlation above 96% was reached for the initial estimated activation sequences, 5) PaceView could be a clinical relevant tool to support the CRT patient selection process (intrinsic activation) and the implantation procedure to verify the optimal localization of the CRT pacemaker leads.

### Normal intrinsic ventricular activation

In recent developments, we used a His-Purkinje system model to obtain a realistic His-Purkinje system activation pattern^21^. The ECGI approach most commonly used a potential-based method, localizing the activation to the epicardium only, thereby omitting the septal activation^26,27^. Better insight into the global ventricular activation, including the septum, might support the patient selection process^28^. The use of the readily available standard 12-lead ECG in ECGI methods is still minimal, especially for intrinsic His-Purkinje-mediated activation sequences^6,29^, and also being a limitation of the commercially available ViVo system, which is tuned to localize a single focus activation (PVC/VT)^5^. This study showed that the 12-lead ECG could also be used to estimate the intrinsic activation sequence for patients with a partially blocked His-Purkinje system. The PaceView algorithm captured the LBBB activation pattern well, showing the earliest activation on the endocardium of the right free wall and late activation in the epicardium of the left free wall for most cases. A similar sequence of ventricular depolarization was demonstrated for LBBB patients using the ECGI approach with a 244-electrode vest^30^. Although current ECGI technologies provide essential features of cardiac activation^31–34^, they still require multichannel ECG recording unsuitable for routine clinical practice. Our study demonstrated that the reconstructed depolarization sequence derived from 99-lead BSPM was similar to the depolarization sequence from 12-lead ECG, either in sinus rhythm or in RV/LV pacing modes. Thus, our findings represent important perspectives for using 12-lead ECG for inverse solution in electrophysiology.

### Localization CRT lead pacing sites

The position of the pacing leads in a CRT patients is important for optimizing CRT outcome. It appears that focusing on the relationship of RV lead regarding LV lead location has a clinical importance^35^. Proposed new approach localizing the pacing lead in both ventricles based on 12-lead ECG can help to ensure that lead electrodes are placed in the targeted locations to provide the clinical improvement.

Septal RV lead placement can only be localized in ECGI methods using a heart model with the septum^28,36^. Such targeted RV septal activation interacts with LV epicardial activation during the biventricular pacing. Omitting the septum, ECGI methods estimating only the epicardial potentials cannot assess the asynchronous activation of the left ventricle and its subsequent correction by biventricular pacing. Consequently, epicardial ECGI methods, at best, can optimize the LV epicardial lead location^2,26^. On the other hand, our strategy allows both septal and epicardial activation assessment. More importantly, we showed that the RV and LV pacing lead locations can be estimated from the 12-lead ECG, without the need for BSPM. A similar observation was reported by Pezzuto et al.^29^. In our study, the localization error of the RV or LV lead was less than 10 mm, irrespective of the number of ECG leads used, 99-lead BSPM, or the 12-lead ECG. That is a localization error of the same range as reported in studies with 184–224 body surface leads^30,34,37^. Interestingly, the localization error in our study is lower than the comparable study using the standard 12-lead ECG by Pezzuto et al.^29^, who reported localization errors of more than 10 mm. Localization error ≈16 mm was described in a pig model with 184-leads BSPM^32^. Inverse mapping with a meshless method of fundamental solutions was better than finite/boundary element methods. A localization error of about 6 mm was demonstrated using three-dimensional cardiac activation imaging with 40–60 body surface leads in rabbits^33^, indicating the importance of transmural reconstruction for accurate assessment of electrical processes in the heart.

Notably, the localization procedure in this study was confined to a particular area of the heart, RV endocardium, and LV epicardium. The modeling approach was to limit the errors and the solution space to realistic dimensions, which is the case when implanting a CRT device. This approach has also limited the computation time significantly, about one second for the initial estimate and 2–3 s for the optimization. This limited computation time could allow the system to be used in a clinical setting during the implantation and/or programming of the CRT device. The limited search area in the ventricles, on the other hand, will not allow the localization of PVC/VT origin, as determined by the ViVo system^8^.

### Impact of regularized optimization

In the method presented here, an electrophysiologically based initial estimate is first generated, subsequently optimized using the Laplacian regularization operator. The initial estimate showed to be highly effective in the localization of pacemaker leads and approximating the intrinsic His-Purkinje mediated activation^21^. However, the resulting ECGs from these initial activation sequences did not match the measured ECGs very well, and negative correlation coefficients were found frequently. Interestingly, the subsequent optimization did not change the dominant features of the initially estimated activation sequence. Due to the limited number of leads in the 12-lead ECG, the optimization does not influence the resulting activation sequence much. The fact that the initial and optimized activation sequence share the same nature indicates that the electrophysiological model used to generate the initial estimate is of utmost importance to obtain these realistic results.

Finally, we noticed that the 12-lead ECG on average, provides a higher correlation after the optimization than the BSPMs. Although this study does not offer conclusive data on this subject, we assume that the difference is caused by the used volume conductor model. Errors in the volume conductor model are for instance, the omission of lungs and other inhomogeneities, and the assumed homogenous anisotropic ventricular myocardium^38^. During the optimization step, these errors are incorporated into the estimated activation sequence. Due to the number of BSPM leads, the errors in the volume conductor model are more likely to influence the optimal activation sequence.

### Clinical implications

Since the LV lead position has been identified as an important determinant for CRT response^39^, reconstructed intrinsic ventricular activation sequence using standard 12-lead ECG may allow localization of target area and individualized LV lead placement. This may enhance CRT response outcomes. In addition, this technique can help to optimize programming of the device to achieve the best fusion between two pacing wavefronts.

Quantitative parameters of electrical dyssynchrony, namely TAT, LVAT, or VEU, usually obtained by BSPM, have been shown to be reliable metrics for the assessment of the CRT effect^2,24,27^. Our observations showed that 12-lead ECG can be used for the reliable determination of TAT, LVAT, and VEU.

Since the computation of an initial and optimized activation sequences takes between 3–4 s, it might be used intra-procedurally to identify the latest site of activation. We believe that using a general model of the heart, the system may provide such clinically useful tool.

### Limitations

The studied cohort is relatively small and limited to patients with nonischemic cardiomyopathy and true LBBB. Thus, our results may not be generalized to patients with ischemic cardiomyopathy or patients with other patterns of intraventricular conduction abnormalities. Despite that, the selected cohort is believed to be sufficient for our model study focused on the accuracy of the novel inverse ECG method and comparison between 12-lead ECG and 99-lead BSPM. Another limitation of this study relates to 12-lead ECG electrode positions since precordial unipolar leads (V1–V6) were selected from the array of 99-lead BSPM. Thus, the position of these electrodes slightly varied from the standard position of these leads. Using non-contrast cardiac computed tomography decreased the accuracy of the interventricular septum positioning in the heart model but did not influence the main findings.

## Conclusions

The novel noninvasive inverse ECG method, PaceView, permits to estimation of ventricular activation sequence from a different number of ECG leads, either 99 BSPM or the standard 12-lead ECG. In addition, it allows localization of the pacing lead position in the RV or LV. Our results show that the noninvasive pacing lead localization error using the 12-lead ECG, is small and comparable to 99-lead BSPM. Therefore, PaceView might be clinically helpful in selecting appropriate candidates for CRT, optimizing the LV/RV pacing sites during CRT implant, and/or adjusting the programing of the device.

## Supplementary Information

Supplementary Information.

## Data Availability

The data underlying this article is not publicly available due to the privacy protection of patients that participated in the study. Results of data analysis for individuals are included in the Supplemental Appendix. The detailed analysis can be shared by the corresponding author upon reasonable request.

## References

[1] Varma N (2019). Evaluation, management, and outcomes of patients poorly responsive to cardiac resynchronization device therapy. J. Am. Coll. Cardiol..

[2] Singh JP (2011). Left ventricular lead position and clinical outcome in the multicenter automatic defibrillator implantation trial-cardiac resynchronization therapy (MADIT-CRT) trial. Circulation.

[3] Ploux S (2015). Electrical dyssynchrony induced by biventricular pacing: implications for patient selection and therapy improvement. Heart Rhythm.

[4] Johnson WB (2017). Body surface mapping using an ECG belt to characterize electrical heterogeneity for different left ventricular pacing sites during cardiac resynchronization: Relationship with acute hemodynamic improvement. Heart Rhythm.

[5] van Dam PM, Boyle NG, Laks MM, Tung R (2016). Localization of premature ventricular contractions from the papillary muscles using the standard 12-lead electrocardiogram: A feasibility study using a novel cardiac isochrone positioning system. Europace.

[6] Misra S (2018). Initial validation of a novel ECGI system for localization of premature ventricular contractions and ventricular tachycardia in structurally normal and abnormal hearts. J. Electrocardiol..

[7] Lesina K (2022). Performance and robustness testing of a non-invasive mapping system for ventricular arrhythmias. Front. Physiol..

[8] Griffiths JR (2023). Non-invasive electrocardiographic mapping on the ward to guide ablation of premature ventricular contractions. J. Electrocardiol..

[9] Fruelund PZ (2023). Novel non-invasive ECG imaging method based on the 12-lead ECG for reconstruction of ventricular activation: A proof-of-concept study. Front. Cardiovasc. Med..

[10] Roudijk RW (2021). Comparing non-invasive inverse electrocardiography with invasive endocardial and epicardial electroanatomical mapping during sinus rhythm. Front. Physiol..

[11] Rosik V, Karas S, Heblakova E, Tysler M, Filipova S (2007). Portable device for high resolution ECG mapping. Meas. Sci. Rev..

[12] van Dam PM, Gordon JP, Laks MM, Boyle NG (2015). Development of new anatomy reconstruction software to localize cardiac isochrones to the cardiac surface from the 12 lead ECG. J. Electrocardiol..

[13] van Oosterom A (2001). Genesis of the T wave as based on an equivalent surface source model. J. Electrocardiol..

[14] Janssen AM, Potyagaylo D, Dössel O, Oostendorp TF (2018). Assessment of the equivalent dipole layer source model in the reconstruction of cardiac activation times on the basis of BSPMs produced by an anisotropic model of the heart. Med. Biol. Eng. Comput..

[15] Geselowitz DB (1989). On the Theory of the electrocardiogram. Proc. IEEE.

[16] Geselowitz DB (1992). Description of cardiac sources in anisotropic cardiac muscle. Application of bidomain model. J. Electrocardiol..

[17] Meijs JW, Weier OW, Peters MJ, van Oosterom A (1989). On the numerical accuracy of the boundary element method. IEEE Trans. Biomed. Eng..

[18] van Dam PM, Oostendorp TF, Linnenbank AC, van Oosterom A (2009). Non-invasive imaging of cardiac activation and recovery. Ann. Biomed. Eng..

[19] van Dam PM, Oostendorp TF, van Oosterom A (2009). Application of the fastest route algorithm in the interactive simulation of the effect of local ischemia on the ECG. Med. Biol. Eng. Comput..

[20] Oosterhoff P (2016). Experimental validation of noninvasive epicardial and endocardial activation imaging. Circ. Arrhythm. Electrophysiol..

[21] Boonstra MJ (2022). Modeling the His-Purkinje effect in non-invasive estimation of endocardial and epicardial ventricular activation. Ann. Biomed. Eng..

[22] Durrer D (1970). Total excitation of the isolated human heart. Circulation.

[23] Opthof T (2017). Cardiac activation-repolarization patterns and ion channel expression mapping in intact isolated normal human hearts. Heart Rhythm.

[24] van Dam PM, Boonstra M, Locati ET, Loh P (2021). The relation of 12-lead ECG to the cardiac anatomy: The normal CineECG. J. Electrocardiol..

[25] Sadek MM (2015). Idiopathic ventricular arrhythmias originating from the moderator band: Electrocardiographic characteristics and treatment by catheter ablation. Heart Rhythm.

[26] Ghosh S (2011). Electrophysiologic substrate and intraventricular left ventricular dyssynchrony in nonischemic heart failure patients undergoing cardiac resynchronization therapy. Heart Rhythm.

[27] Ploux S (2013). Noninvasive electrocardiographic mapping to improve patient selection for cardiac resynchronization therapy: Beyond QRS duration and left bundle branch block morphology. J. Am. Coll. Cardiol..

[28] Dawoud F (2016). Non-invasive electromechanical activation imaging as a tool to study left ventricular dyssynchronous patients: Implication for CRT therapy. J. Electrocardiol..

[29] Pezzuto S (2021). Reconstruction of three-dimensional biventricular activation based on the 12-lead electrocardiogram via patient-specific modelling. Europace.

[30] Jia P (2006). Electrocardiographic imaging of cardiac resynchronization therapy in heart failure: Observation of variable electrophysiologic responses. Heart Rhythm.

[31] Ghanem RN (2005). Noninvasive electrocardiographic imaging (ECGI): Comparison to intraoperative mapping in patients. Heart Rhythm.

[32] Bear LR (2018). How accurate is inverse electrocardiographic mapping? A systematic in vivo evaluation. Circ. Arrhythm. Electrophysiol..

[33] Han C, Liu Z, Zhang X, Pogwizd S, He B (2008). Noninvasive three-dimensional cardiac activation imaging from body surface potential maps: A computational and experimental study on a rabbit model. IEEE Trans. Med. Imaging.

[34] Cluitmans MJM (2017). In vivo validation of electrocardiographic imaging. JACC EP.

[35] Ali-Ahmed F (2021). Right ventricular lead location and outcomes among patients with cardiac resynchronization therapy: A meta-analysis. Prog. Cardiovasc. Dis..

[36] Nguyen UC (2019). Integration of cardiac magnetic resonance imaging, electrocardiographic imaging, and coronary venous computed tomography angiography for guidance of left ventricular lead positioning. Europace.

[37] Revishvili AS (2015). Validation of the mapping accuracy of a novel non-invasive epicardial and endocardial electrophysiology system. Europace.

[38] Boonstra M (2023). ECG-based techniques to enhance clinical practice in cardiac genetic disease management. J. Electrocardiol..

[39] Khan FZ (2012). Targeted left ventricular lead placement to guide cardiac resynchronization therapy: The TARGET study: A randomized, controlled trial. J. Am. Coll. Cardiol..

