# Supplementary material for: Localization of the ventricular pacing site from BSPM and standard 12-lead ECG: a comparison study
Source: Sci Rep. 2023 Jun 14;13:9618. doi: 10.1038/s41598-023-36768-z (PMC10267217; doi:10.1038/s41598-023-36768-z)
Supplement: Supplementary file 1 — Supplementary Information. [file 41598_2023_36768_MOESM1_ESM.pdf]

# All results for "Localization of the Ventricular Pacing Site from BSPM and standard 12-lead ECG: a Comparison Study"

Per patient 3 pages:

- Page 1:  Location of CRT pacing sites in right chamber and left epicard
- Page 2: Localized RV and LV lead of patient. A geodesic error of 0 mm means that the identified lead location was on the triangle to which the CT derived lead location was projected. For instance see initial estimate RV stim location. The dark red point is the vertex from which the simulated activation started, the yellow dot is the gold standard lead location derived from CT.
- Page 3: Activation sequence for the intrinsic rhythm derived from 12-lead ECG or 99 lead Body surface potential map.

# Pat001

RV Stim site

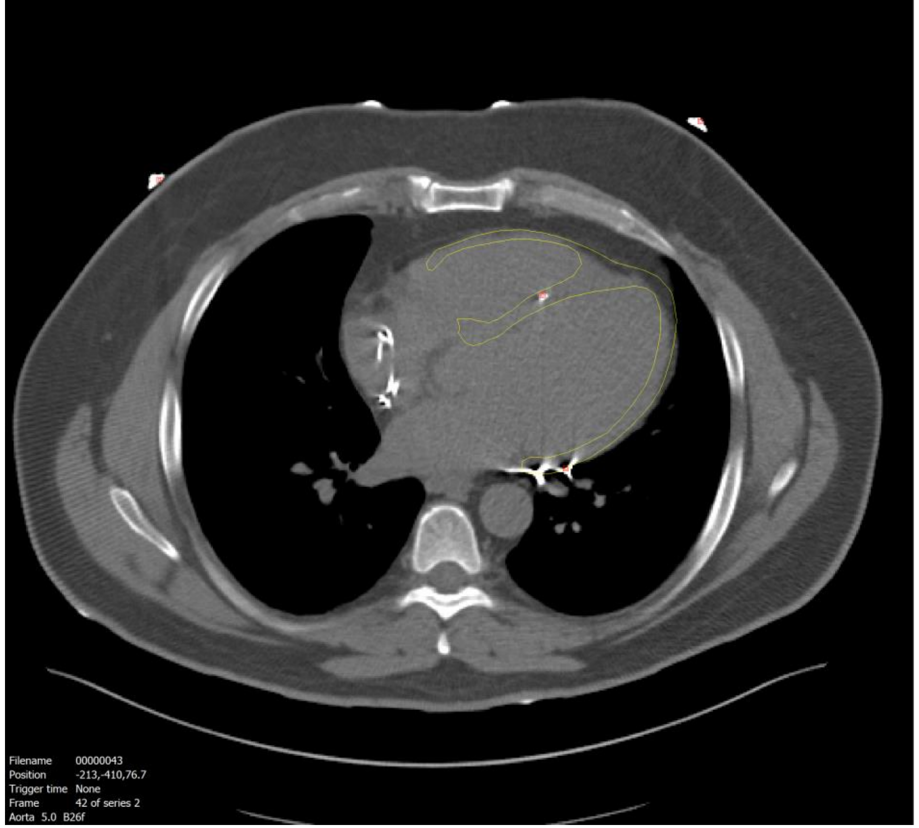


LV stim site

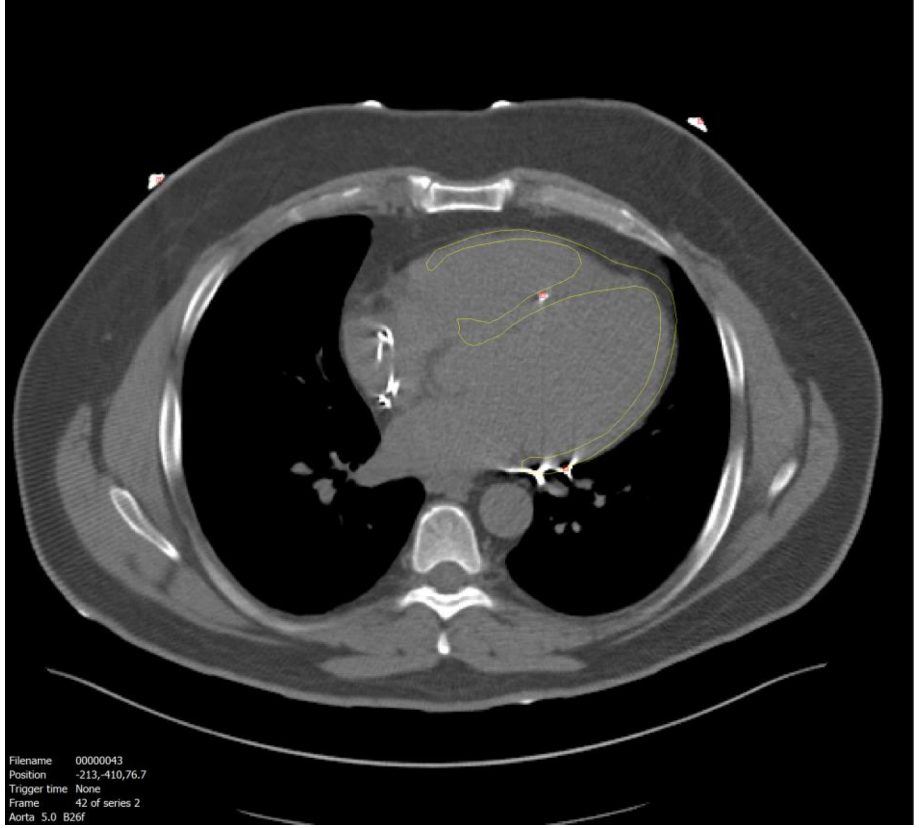


3D positions

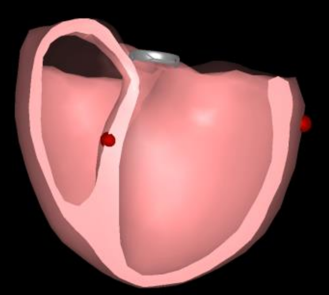

# LV STIM localization

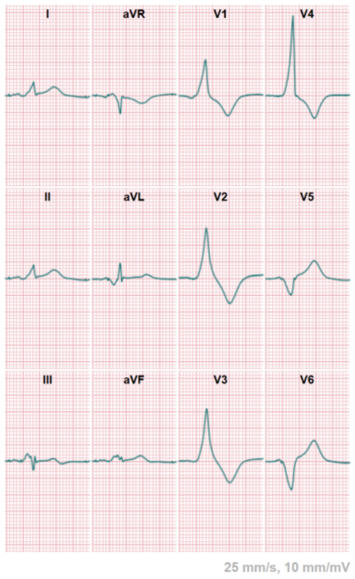


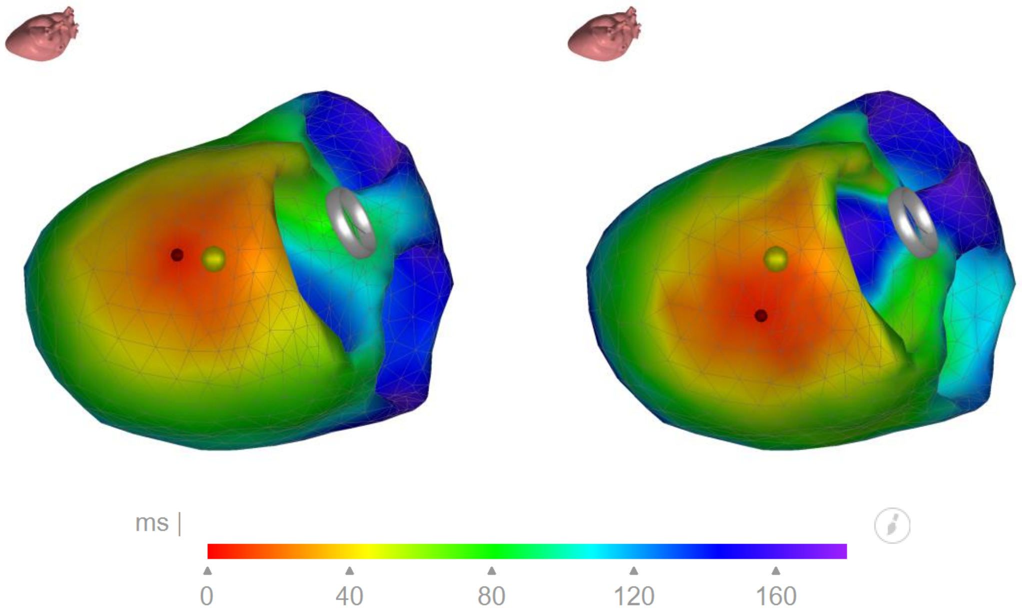


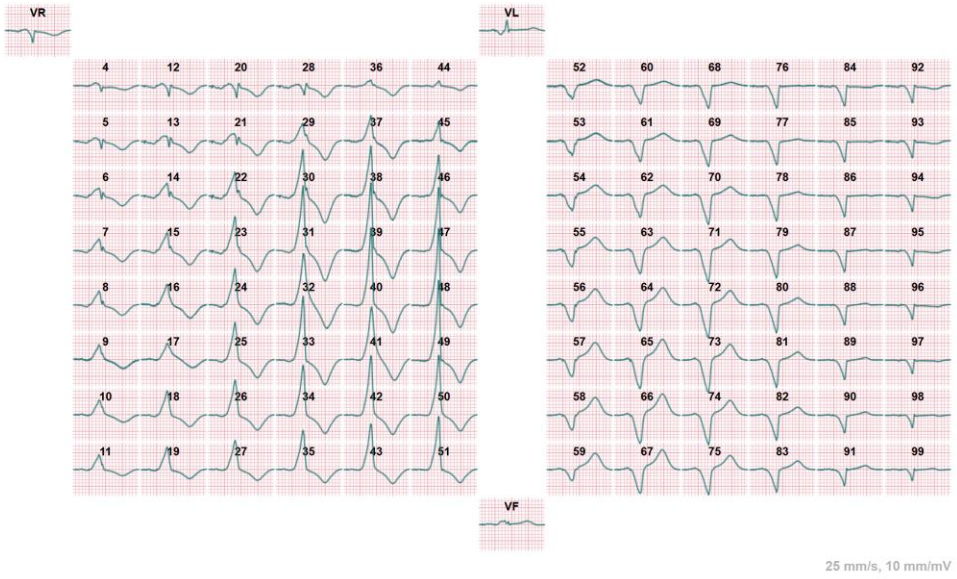


# RV STIM localization

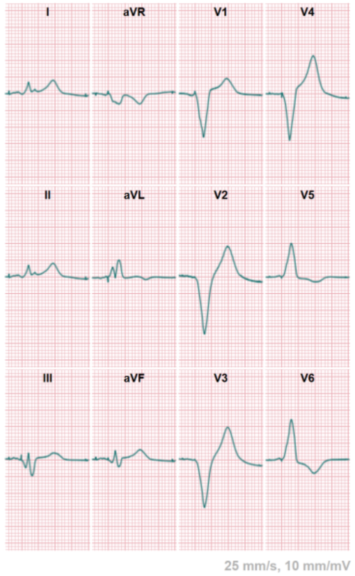


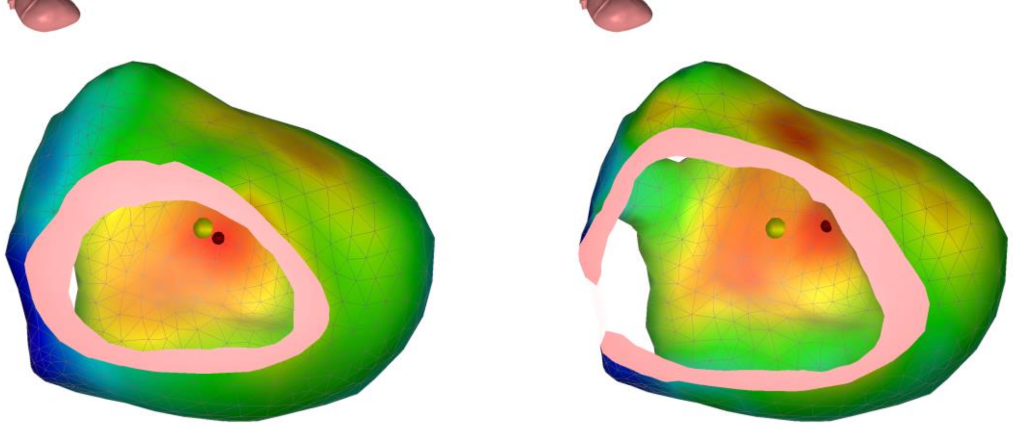

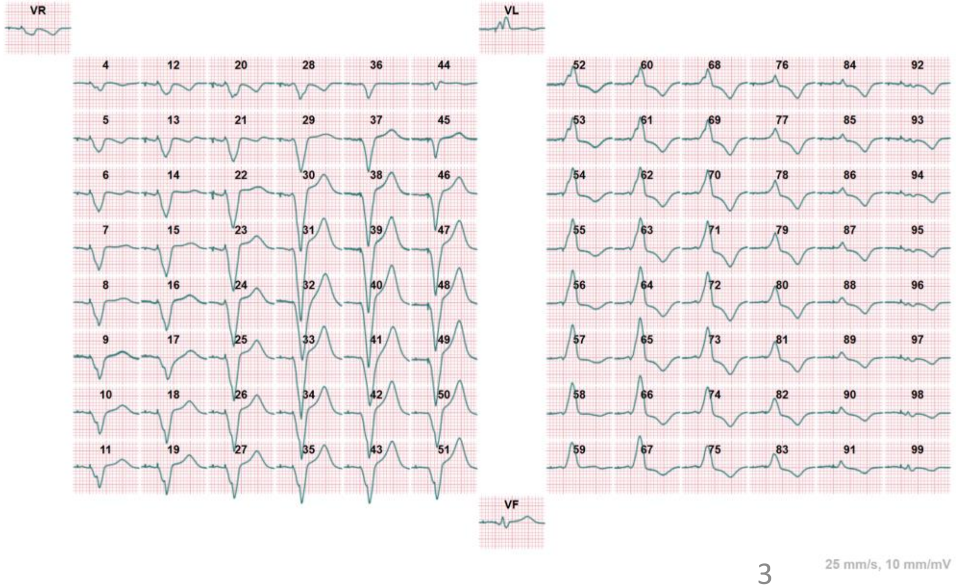

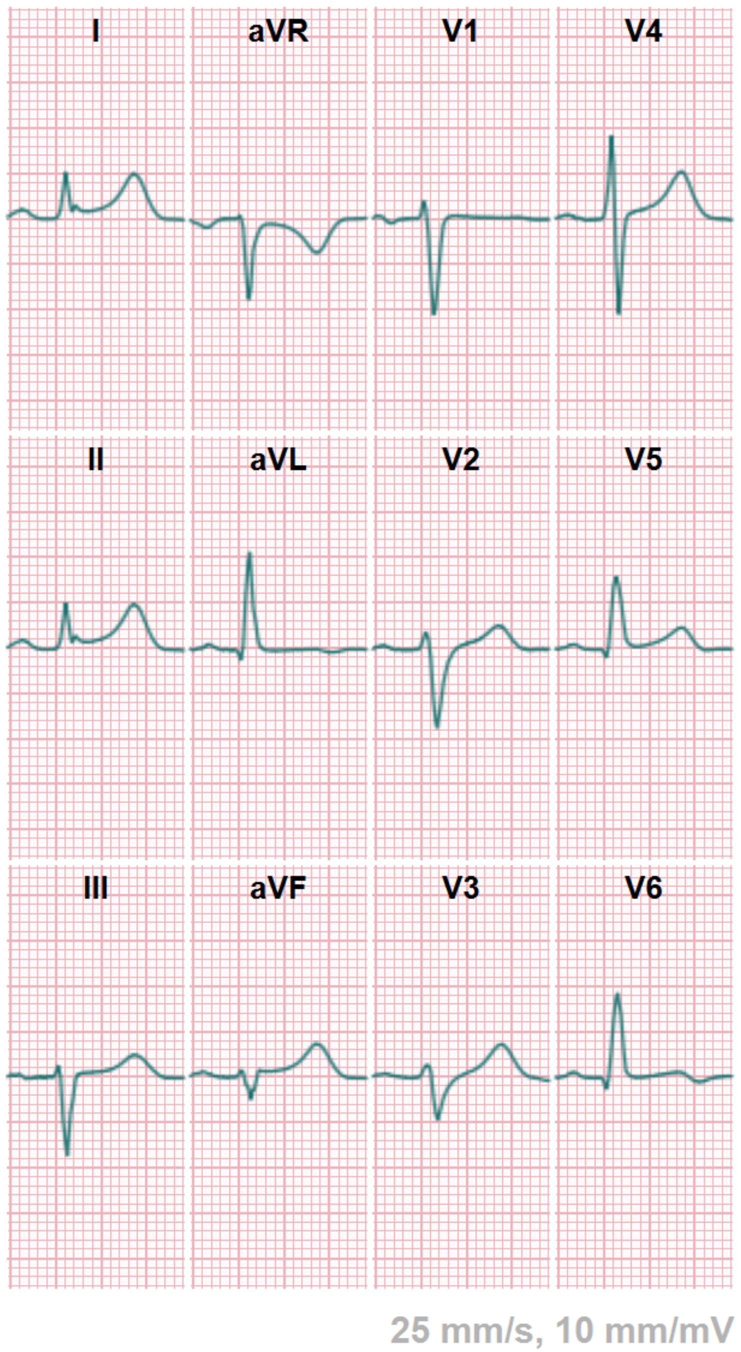
I
aVR
V1
V4
II
aVL
V2
V5
III
aVF
V3
V6
25 mm/s, 10 mm/mV

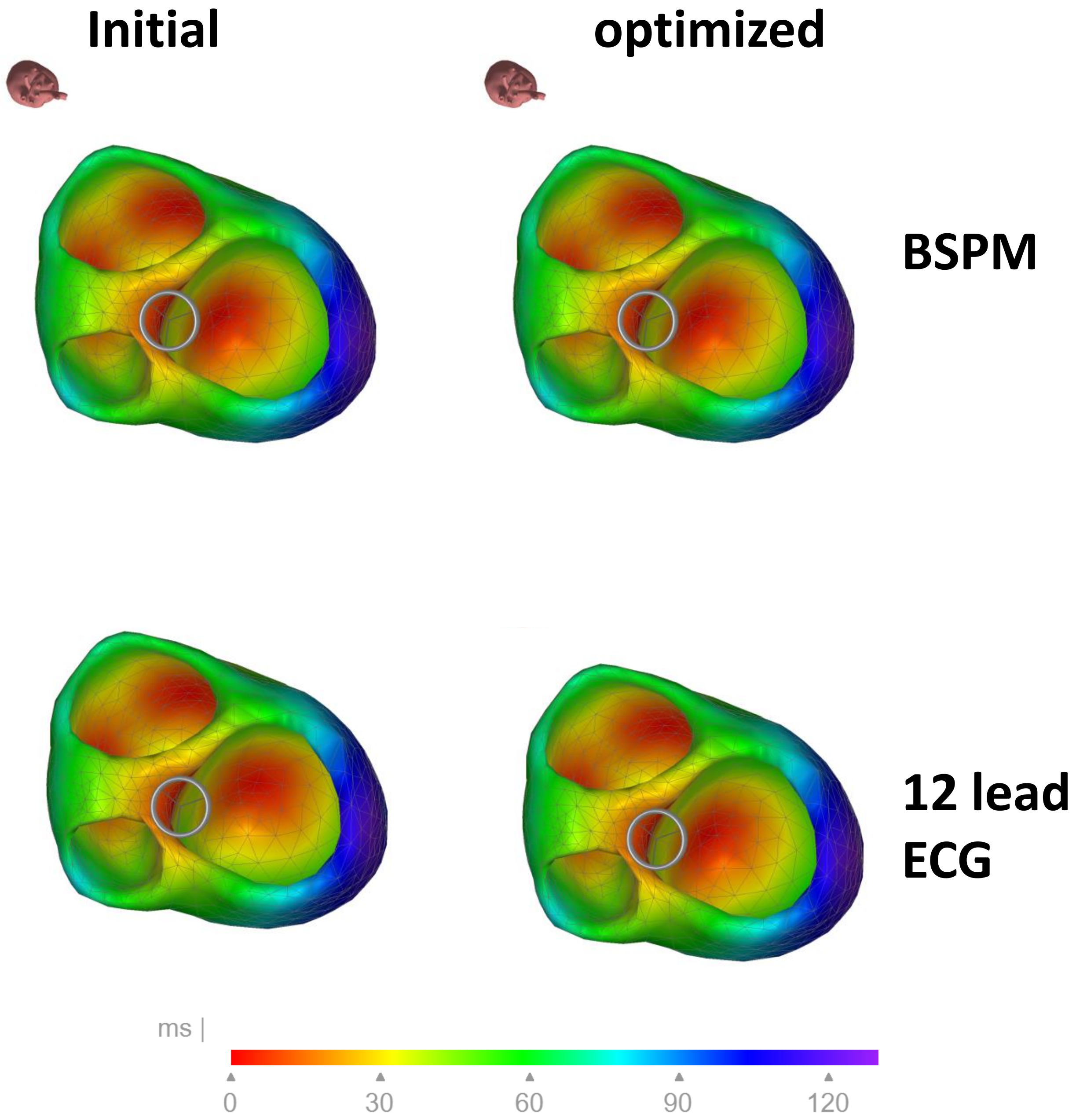
Initial
optimized
BSPM
12 lead
ECG
ms |
0
30
60
90
120

# Pat002

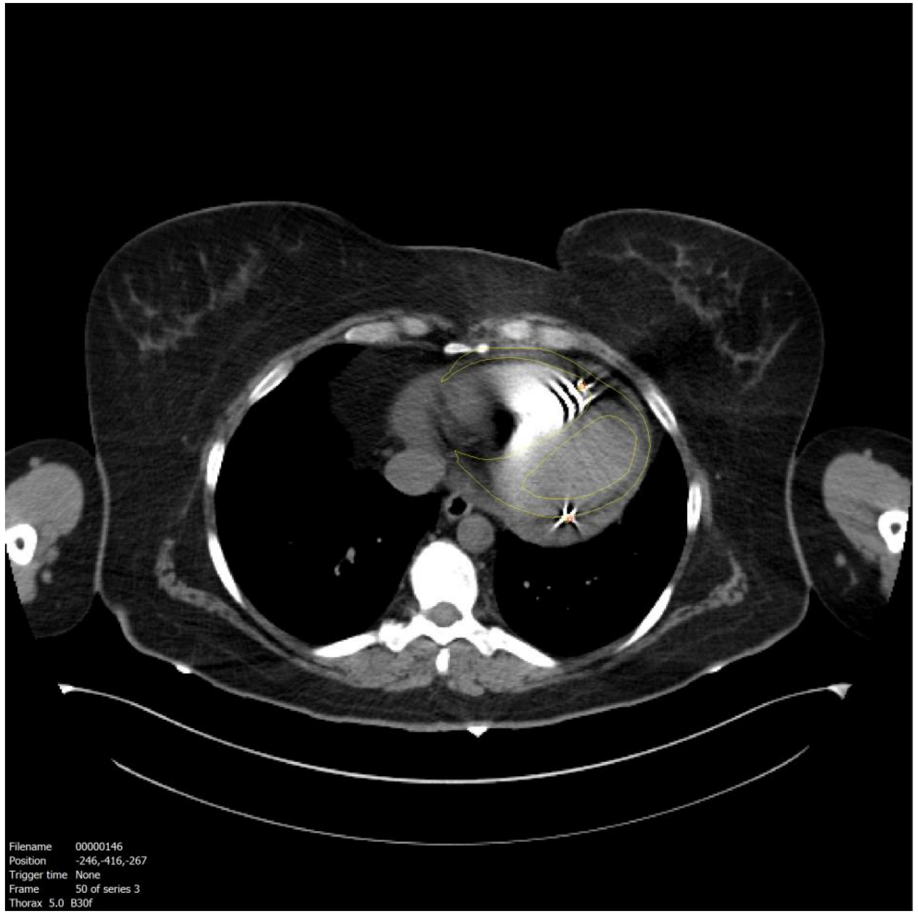
Filename 00000146
Position -246,-416,-267
Trigger time None
Frame 50 of series 3
Thorax 5.0 B30f

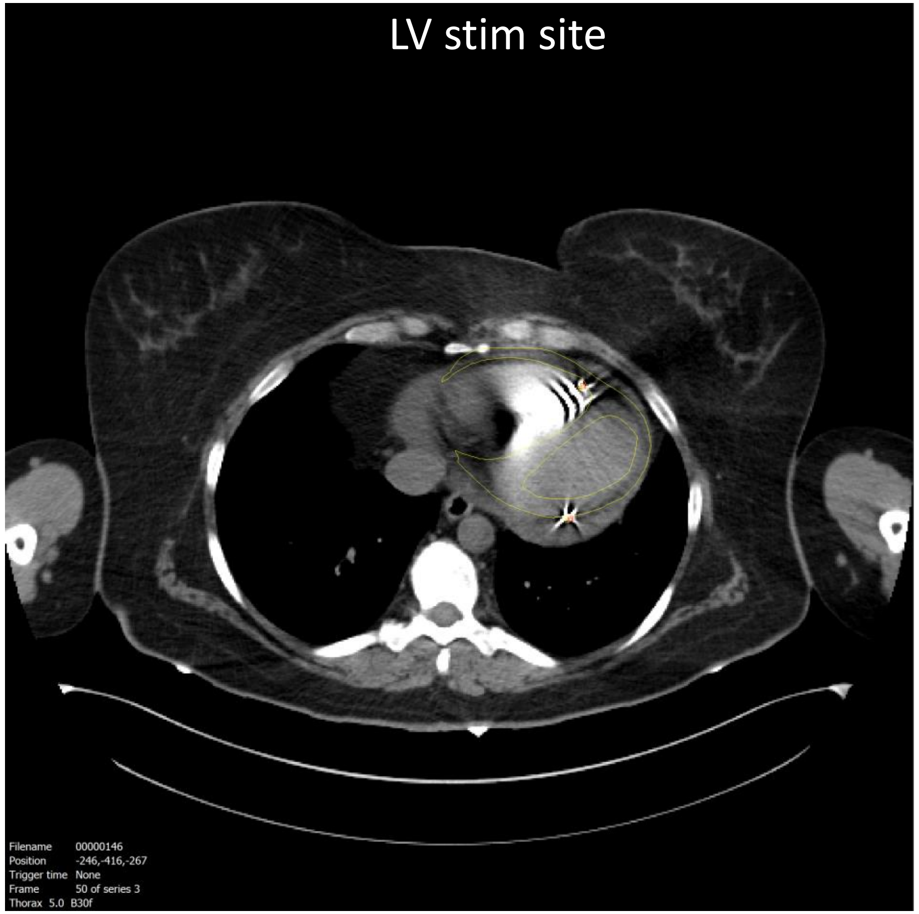
LV stim site
Filename 00000146
Position -246,-416,-267
Trigger time None
Frame 50 of series 3
Thorax 5.0 B30f

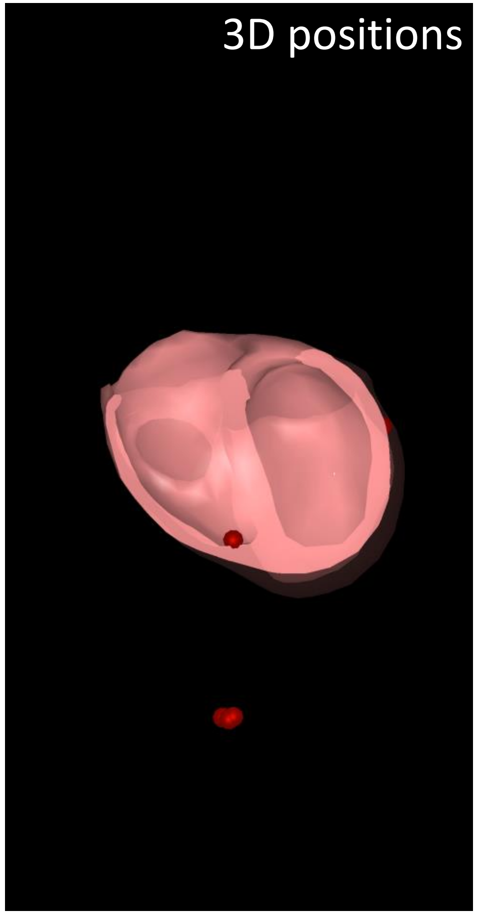
3D positions

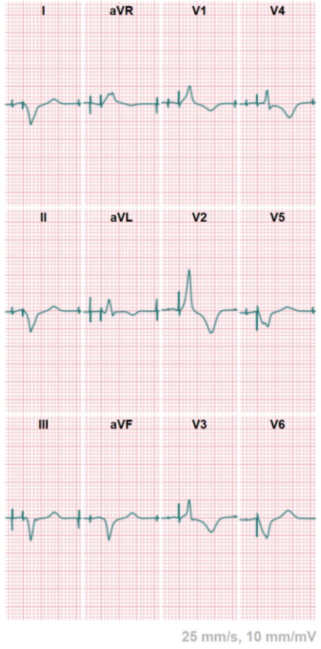


## LV STIM localization

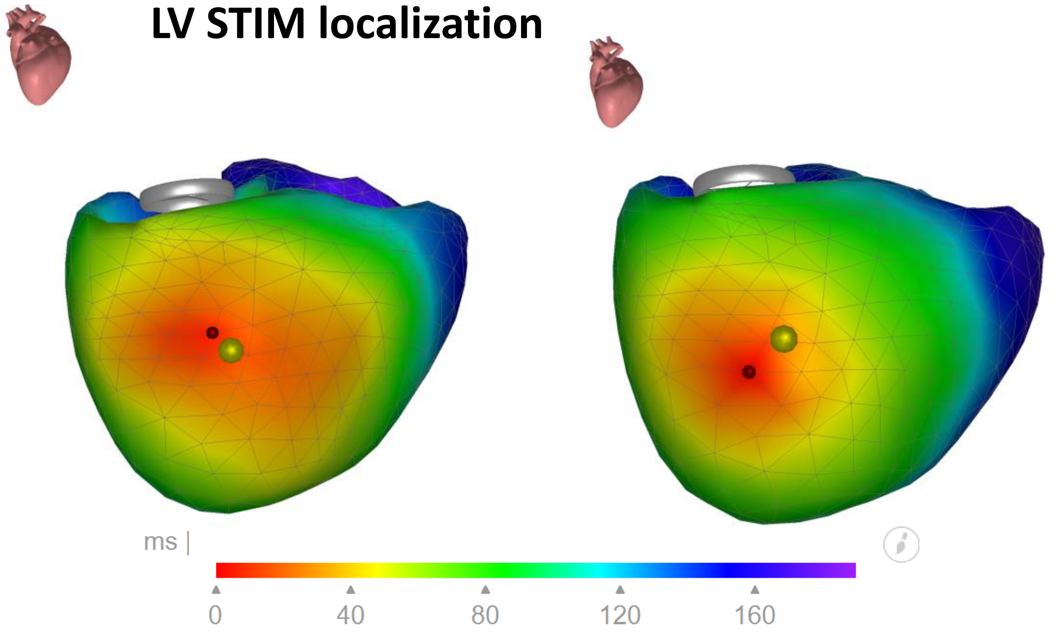


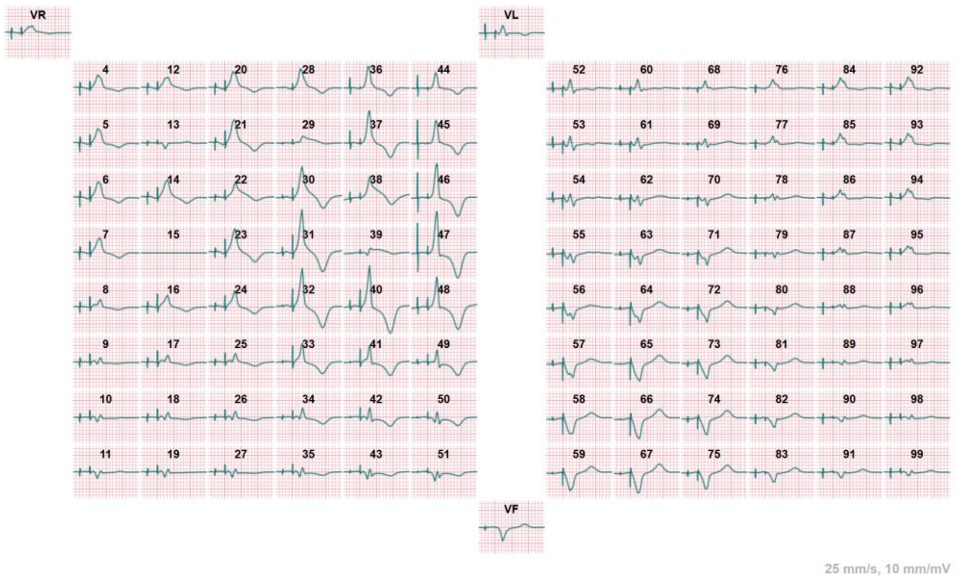


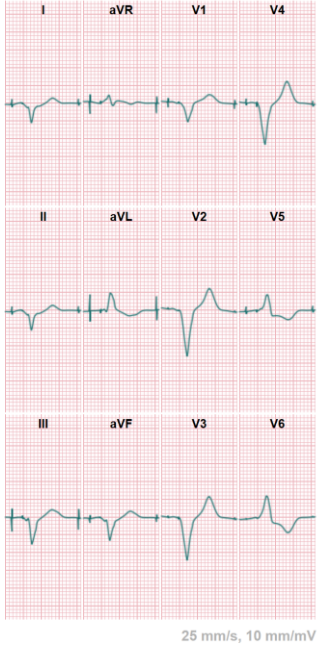


## RV STIM localization

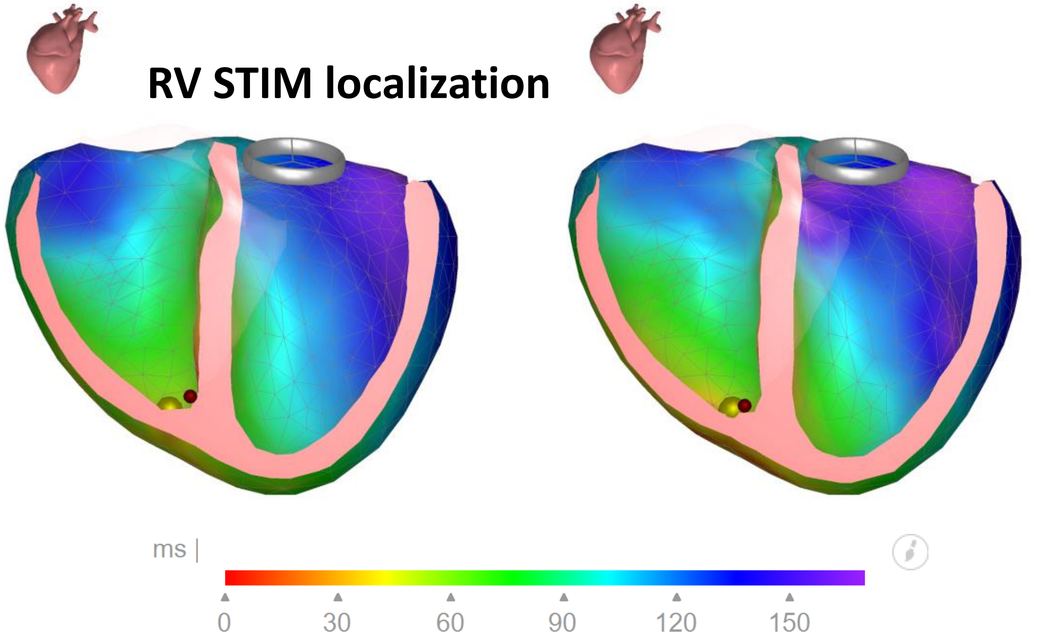


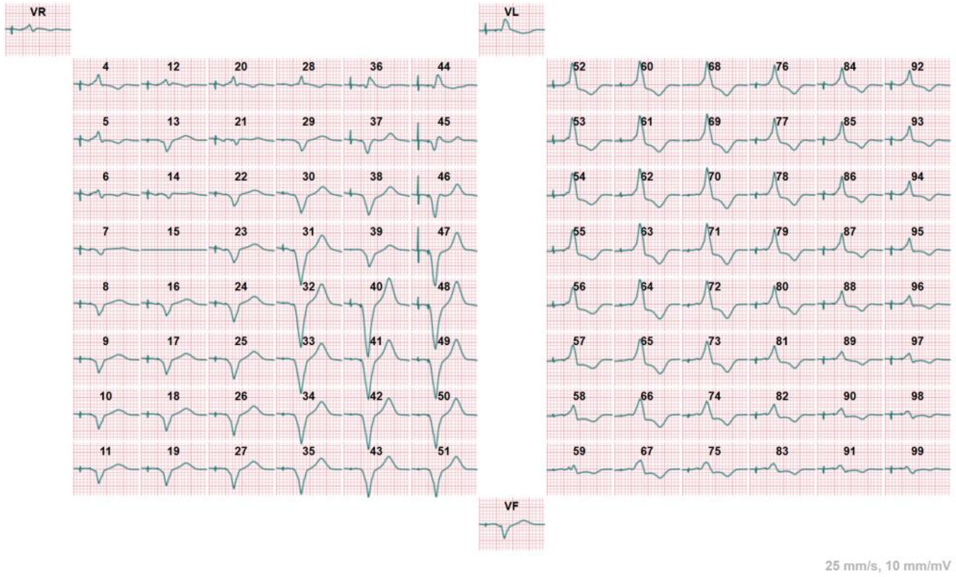

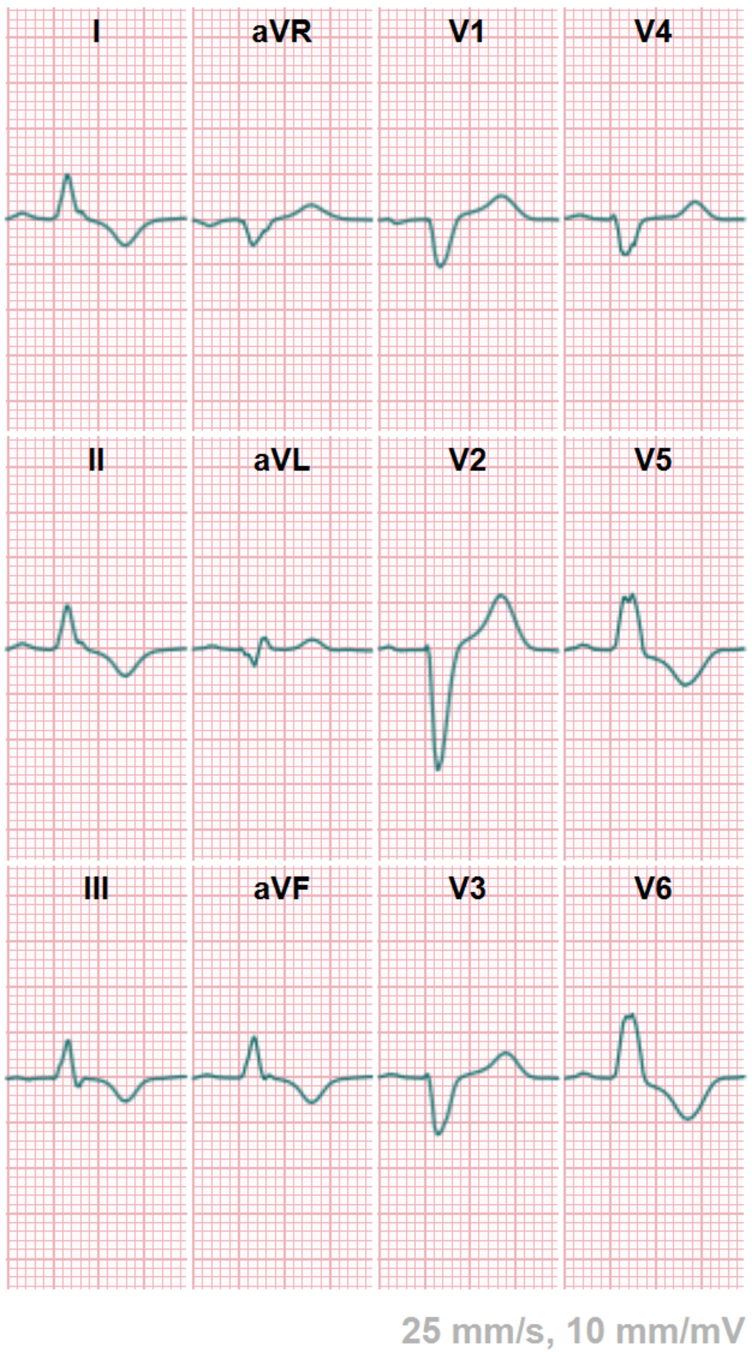
I
aVR
V1
V4
II
aVL
V2
V5
III
aVF
V3
V6
25 mm/s, 10 mm/mV

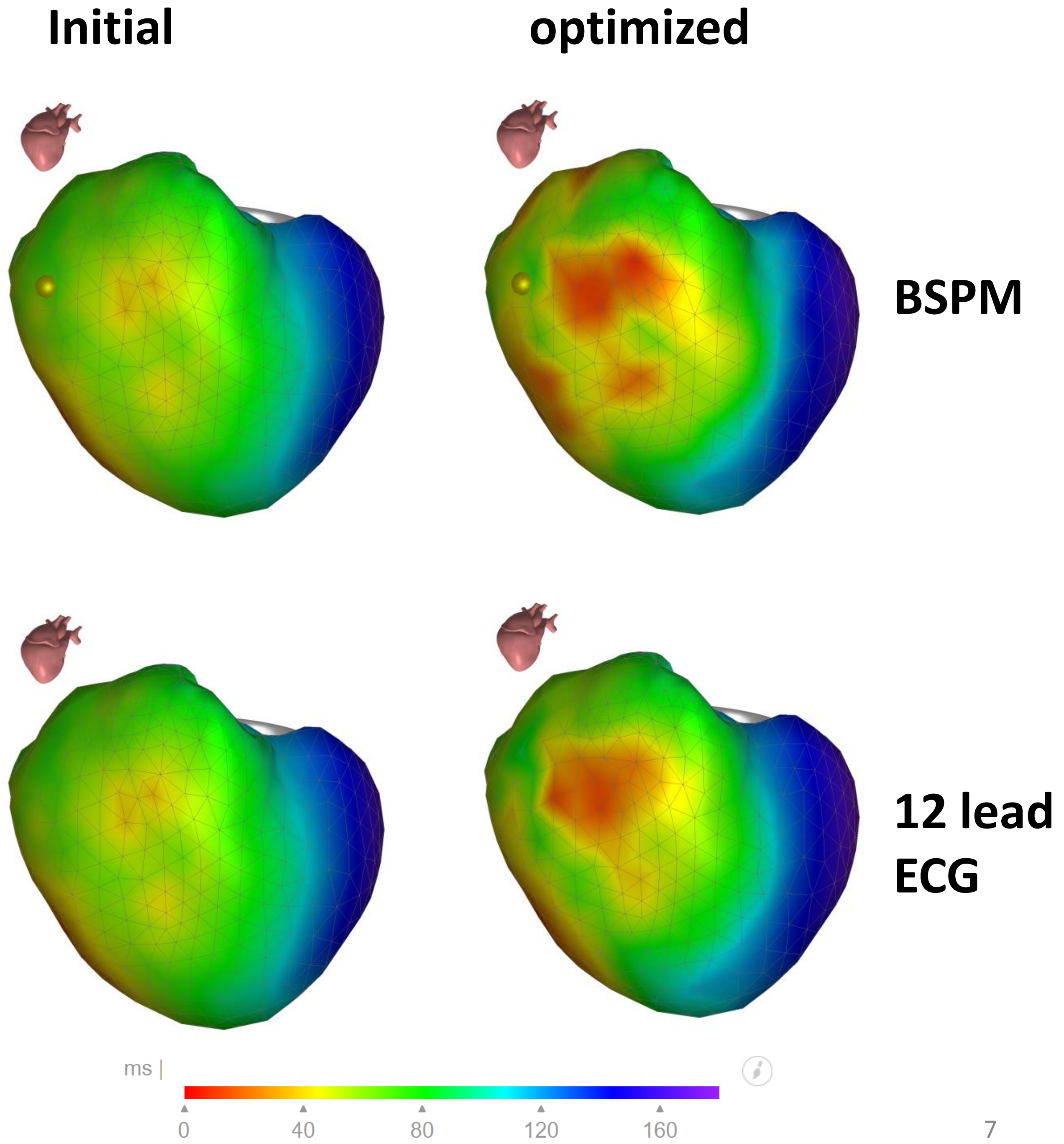
Initial
optimized
BSPM
12 lead
ECG
ms |
0
40
80
120
160

# Pat003

RV Stim site

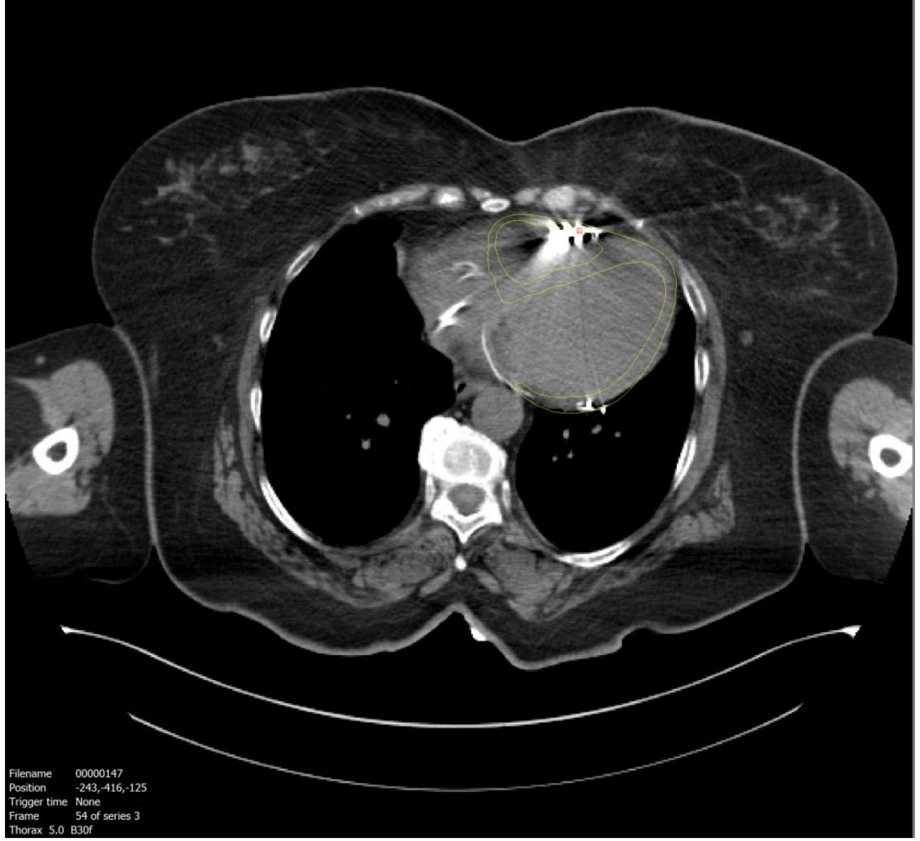


LV stim site

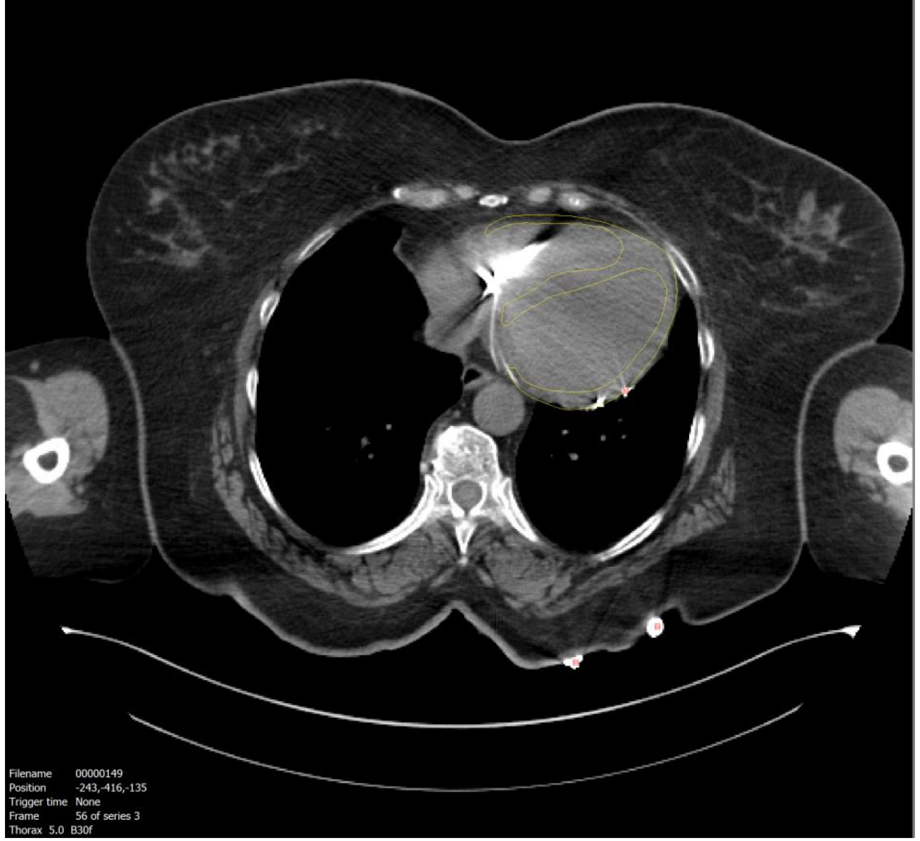


3D positions

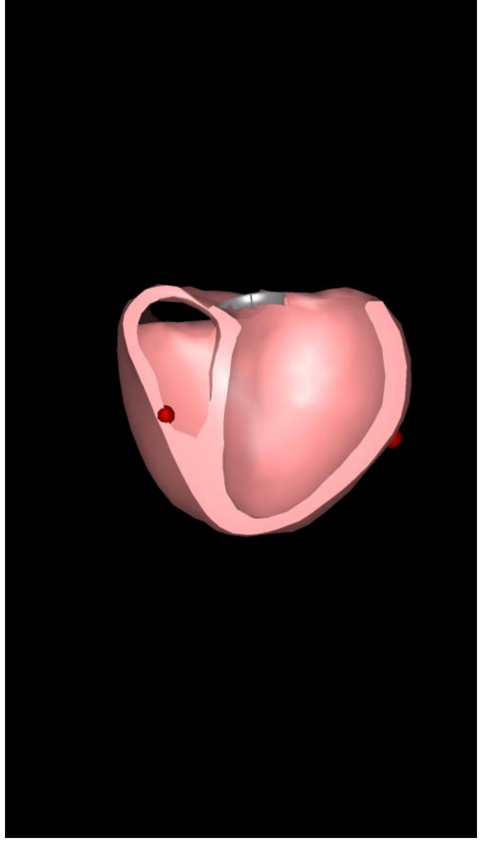

initial

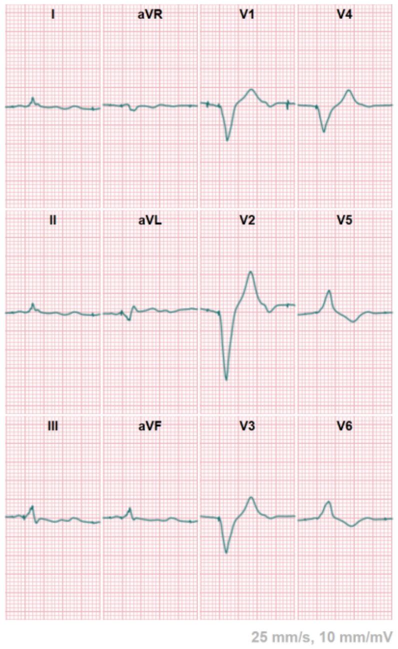


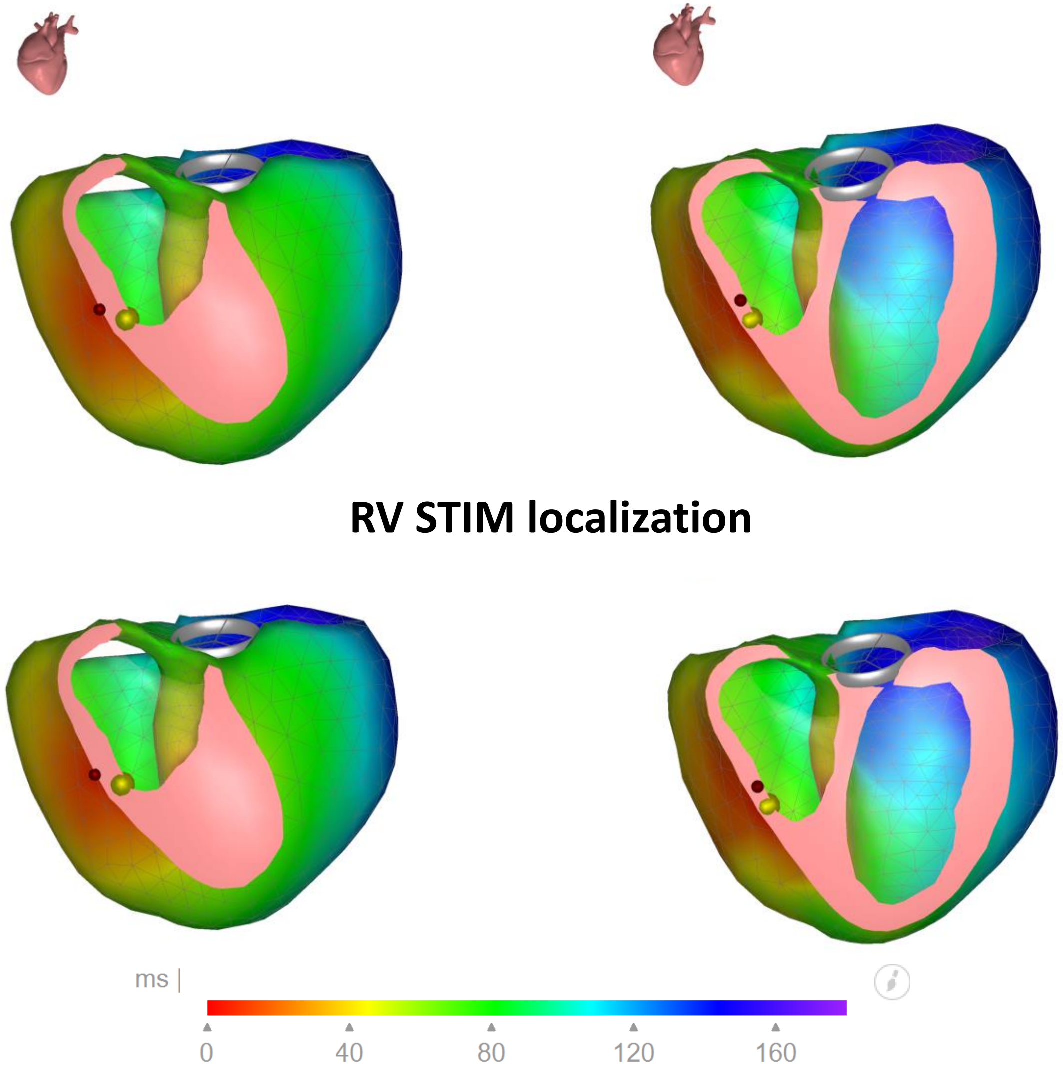


# RV STIM localization

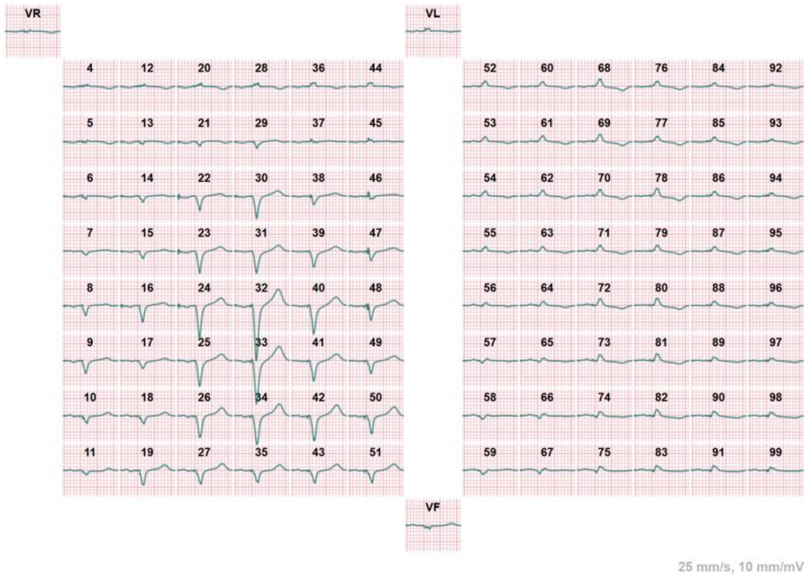

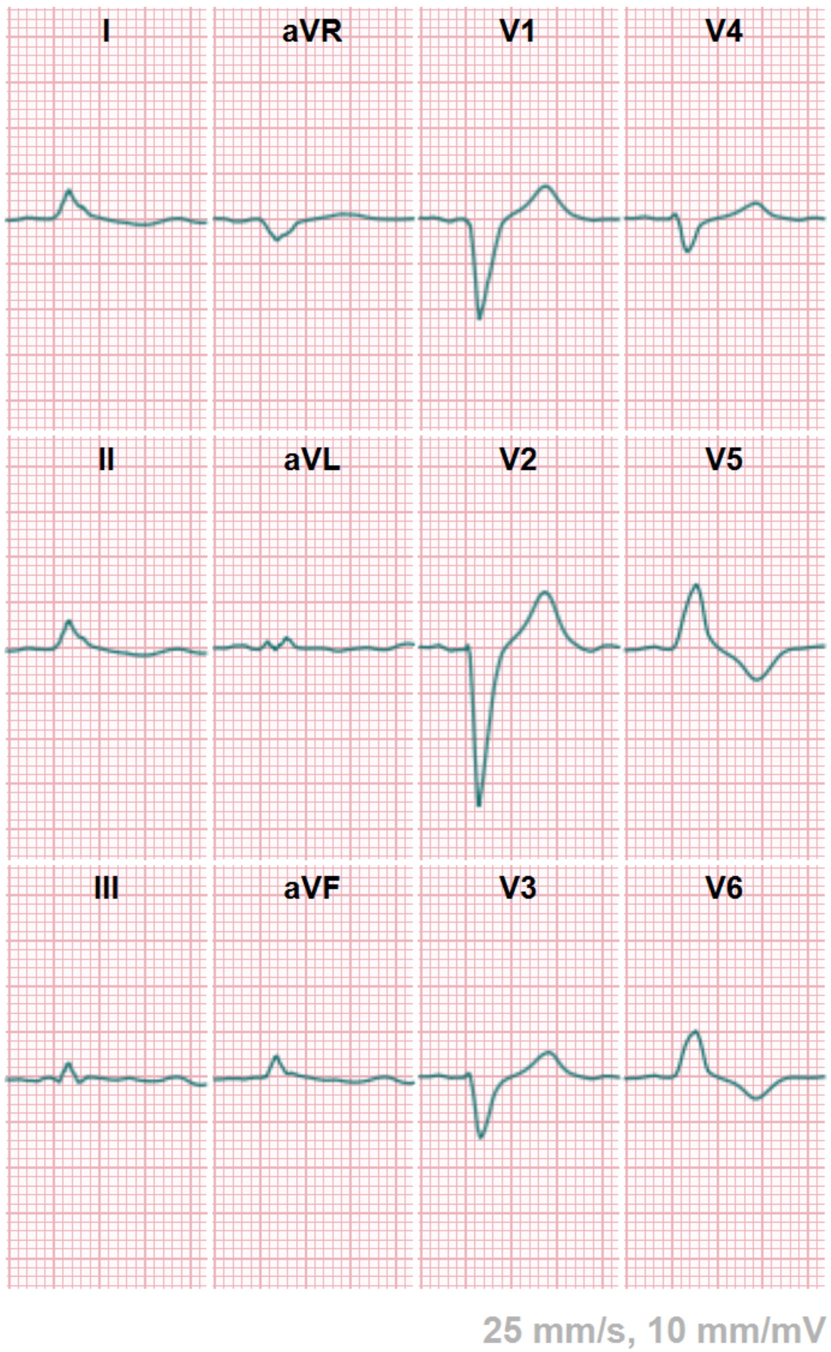
I
aVR
V1
V4
II
aVL
V2
V5
III
aVF
V3
V6
25 mm/s, 10 mm/mV

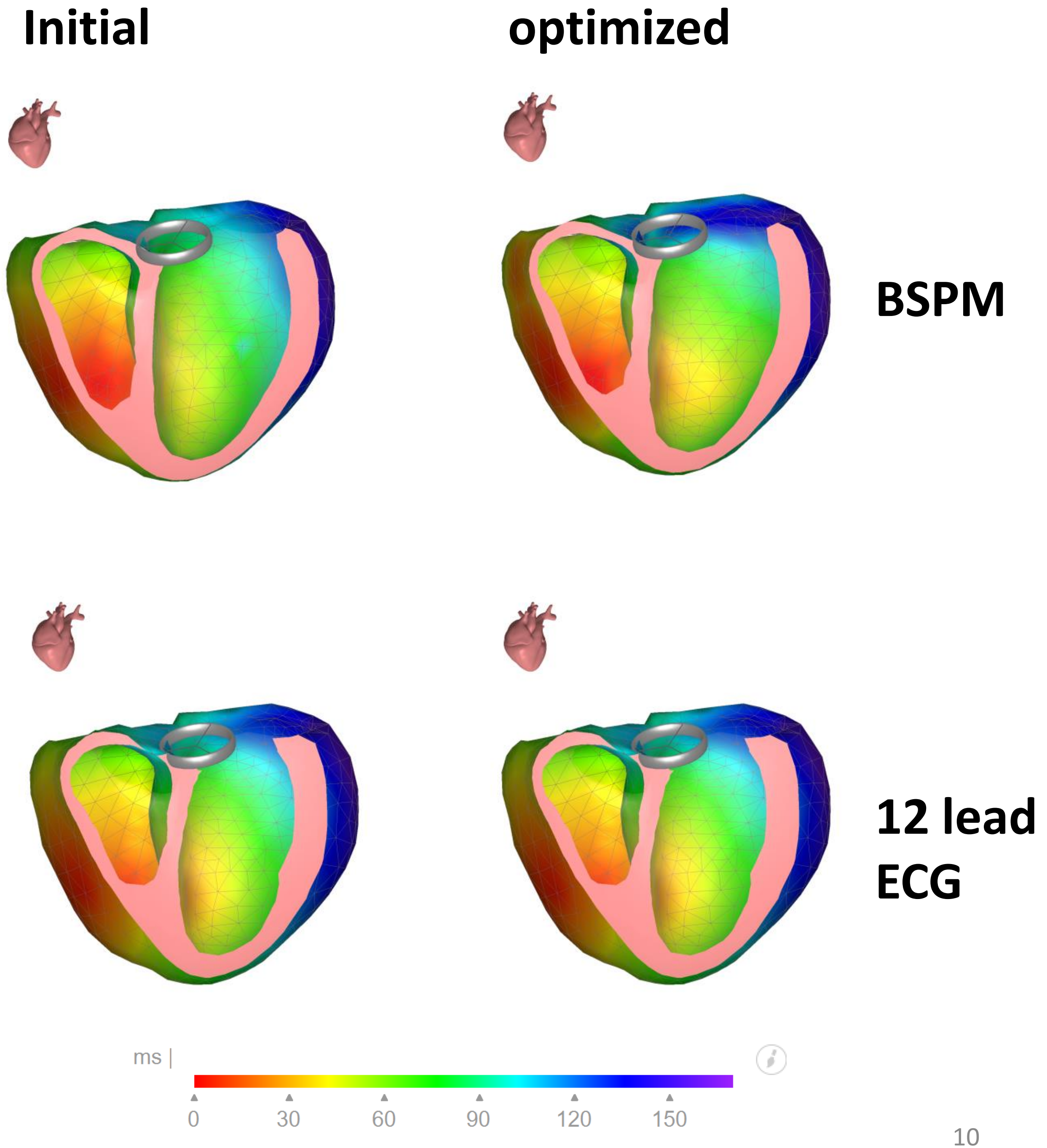
Initial
optimized
BSPM
12 lead
ECG
ms
0
30
60
90
120
150

# Pat004

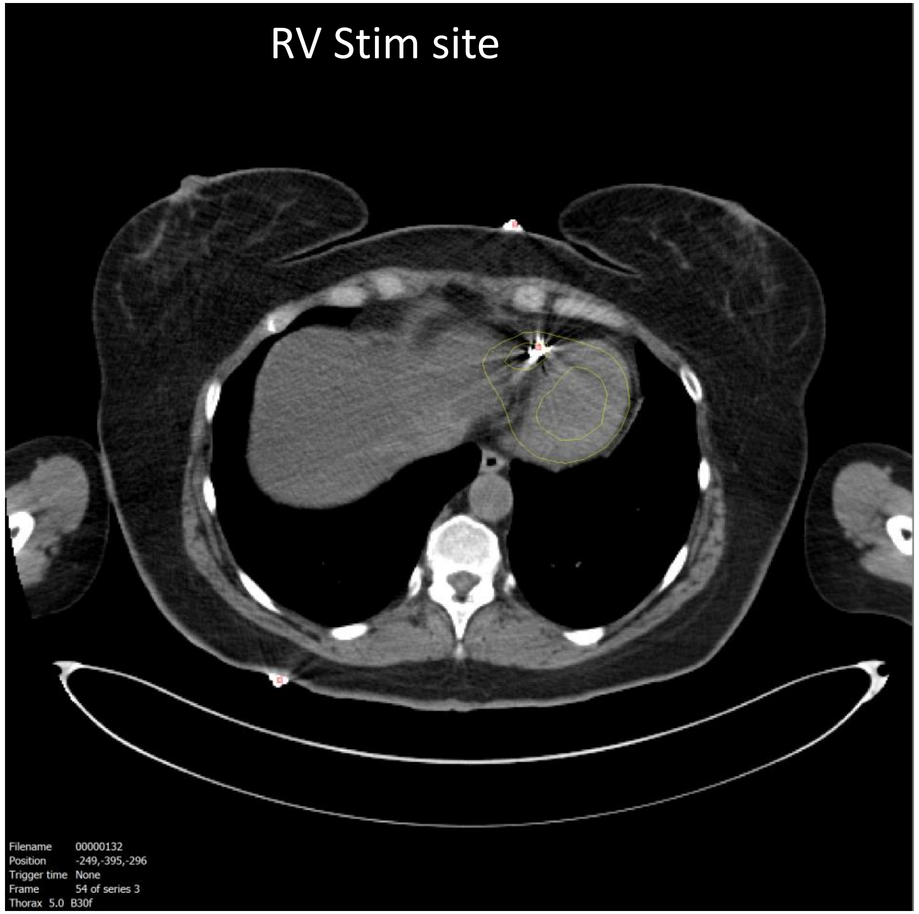
RV Stim site
Filename 00000132
Position -249,-395,-296
Trigger time None
Frame 54 of series 3
Thorax 5.0 B30f

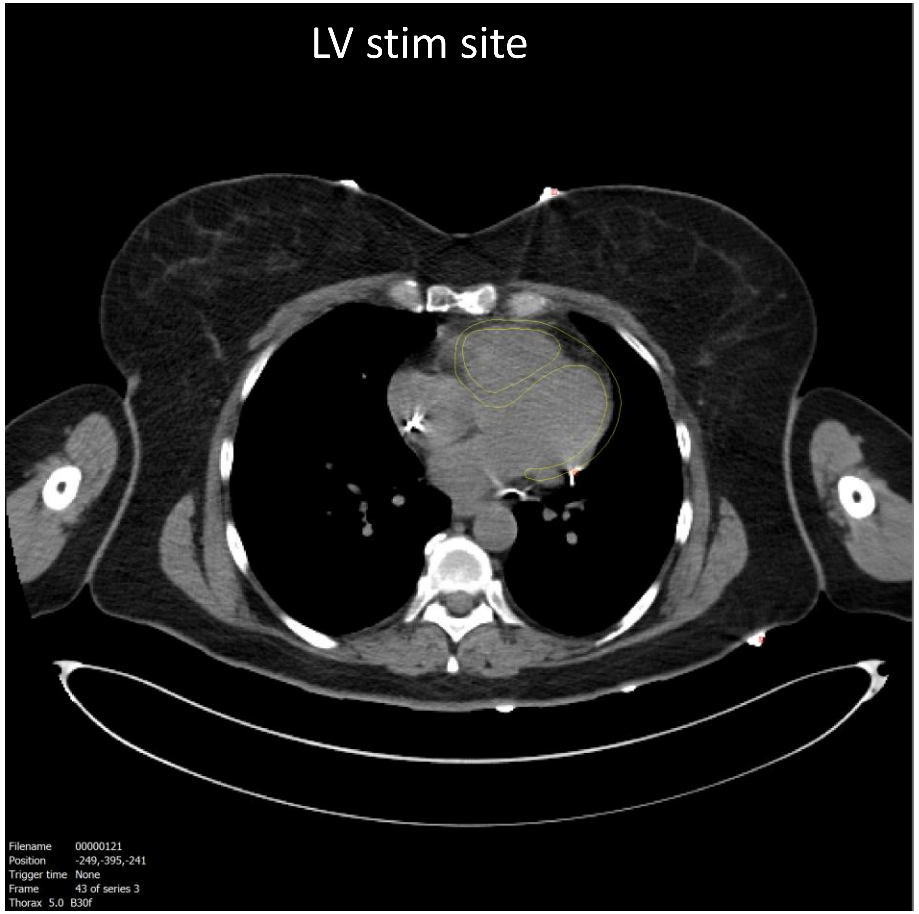
LV stim site
Filename 00000121
Position -249,-395,-241
Trigger time None
Frame 43 of series 3
Thorax 5.0 B30f

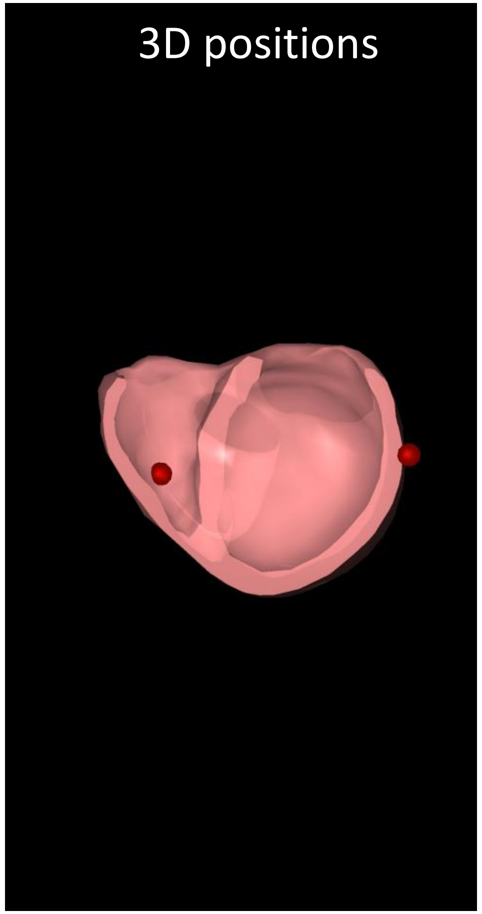
3D positions

# LV STIM localization

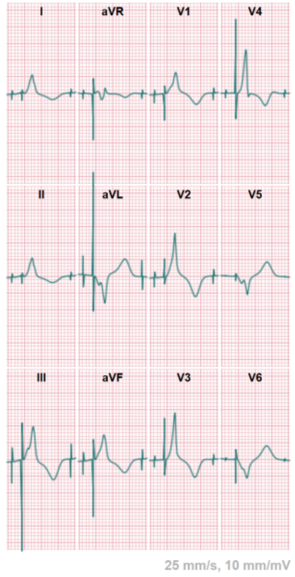


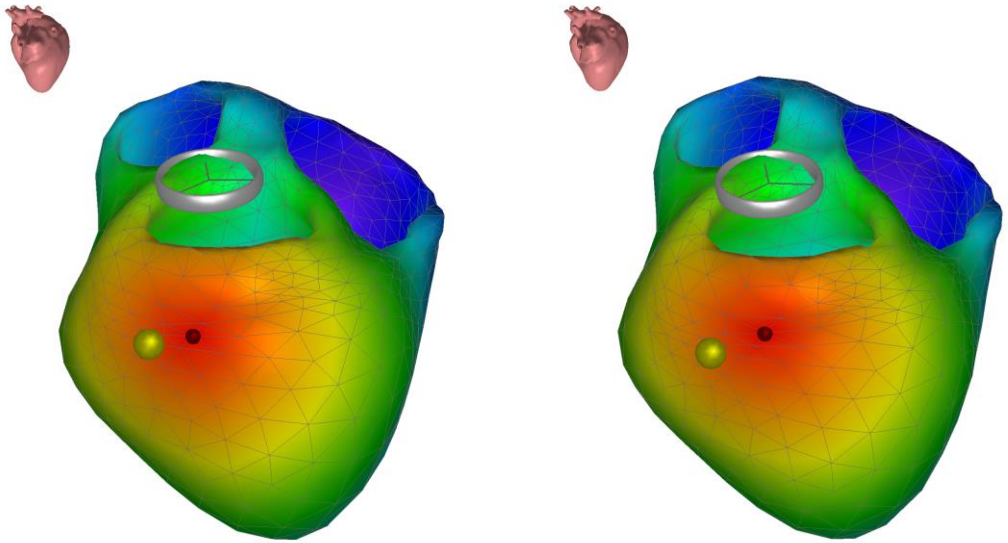

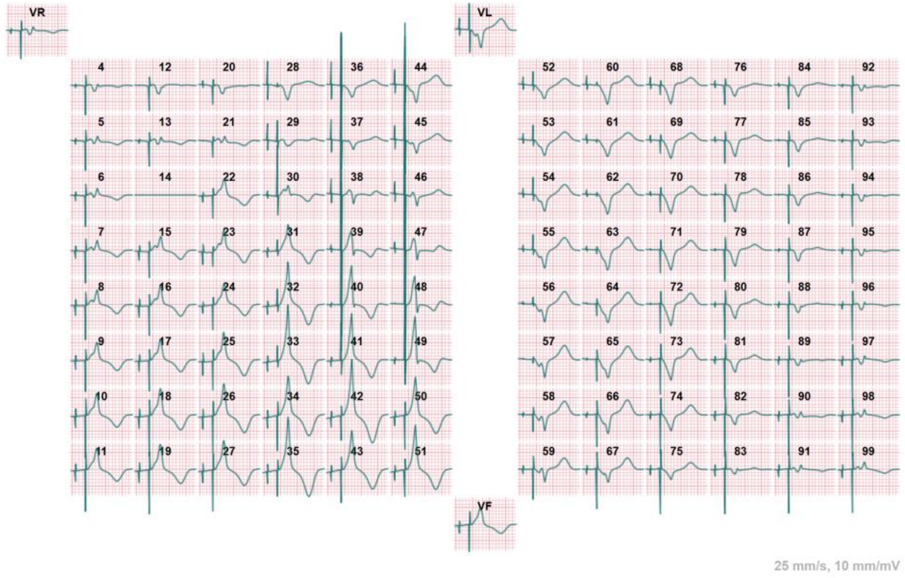


# RV STIM localization

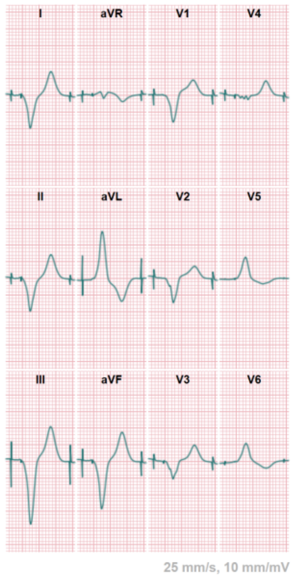


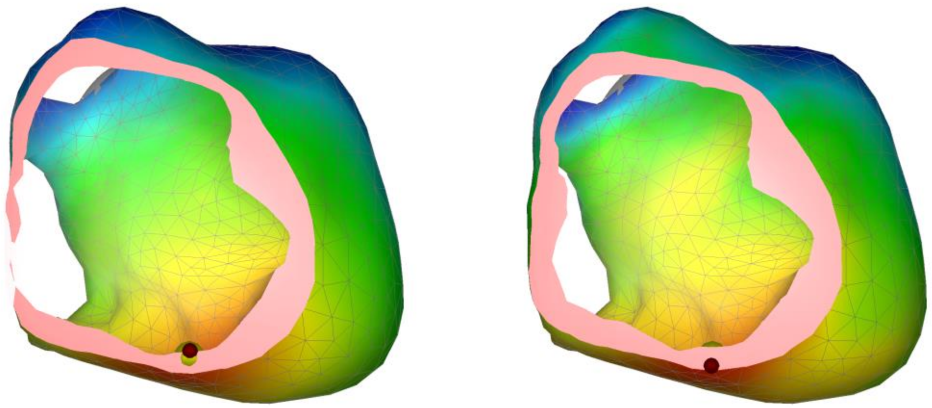

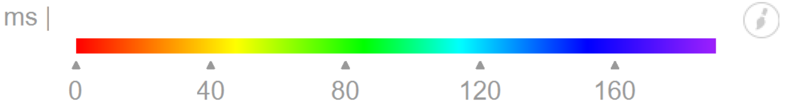


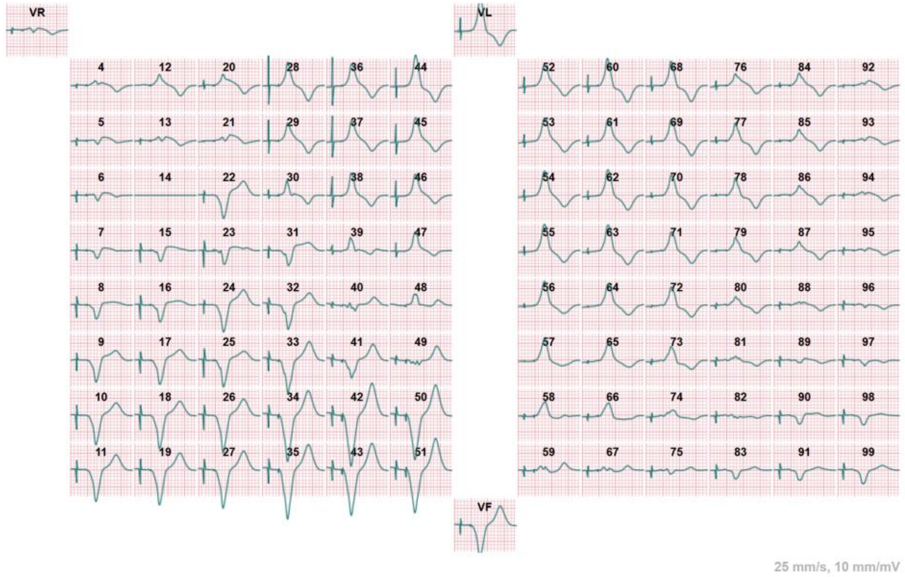

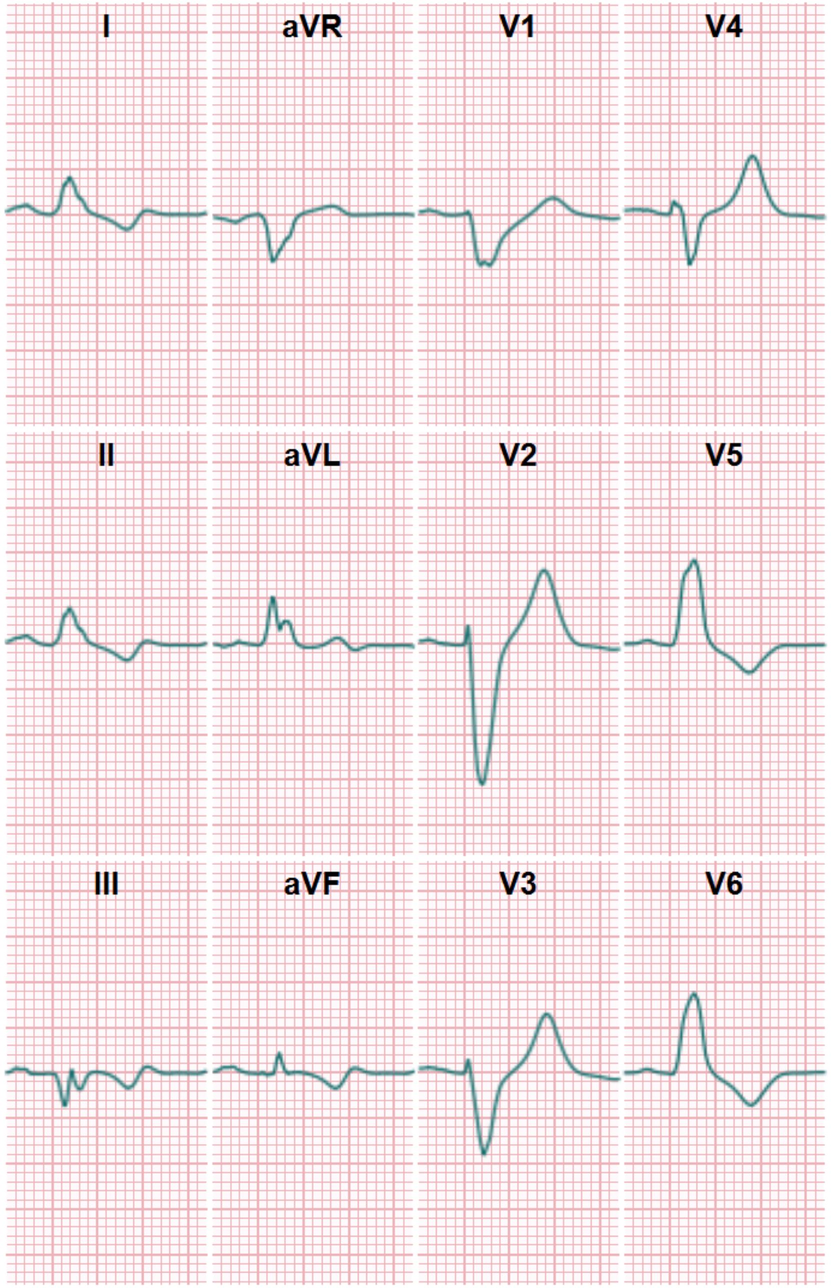


25 mm/s, 10 mm/mV

Initial

optimized

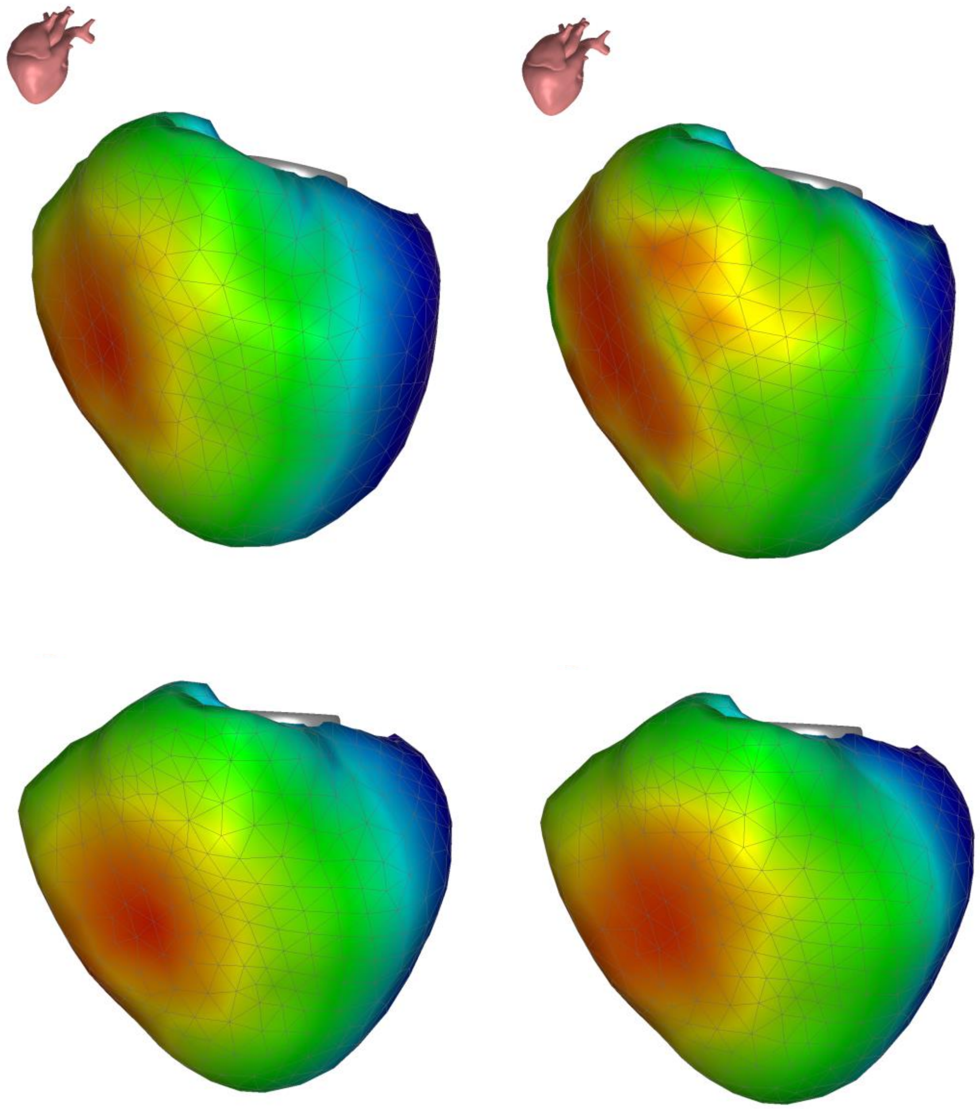

BSPM

12 lead ECG

# Pat005

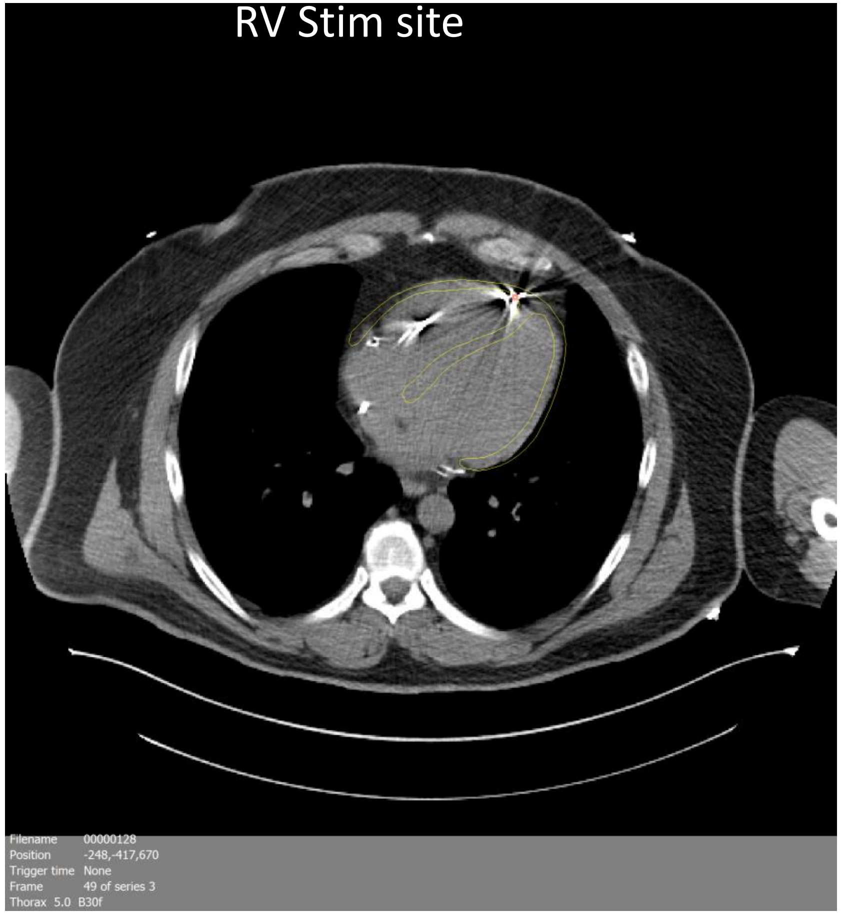
RV Stim site
Filename 00000128
Position -248,-417,670
Trigger time None
Frame 49 of series 3
Thorax 5.0 B30f

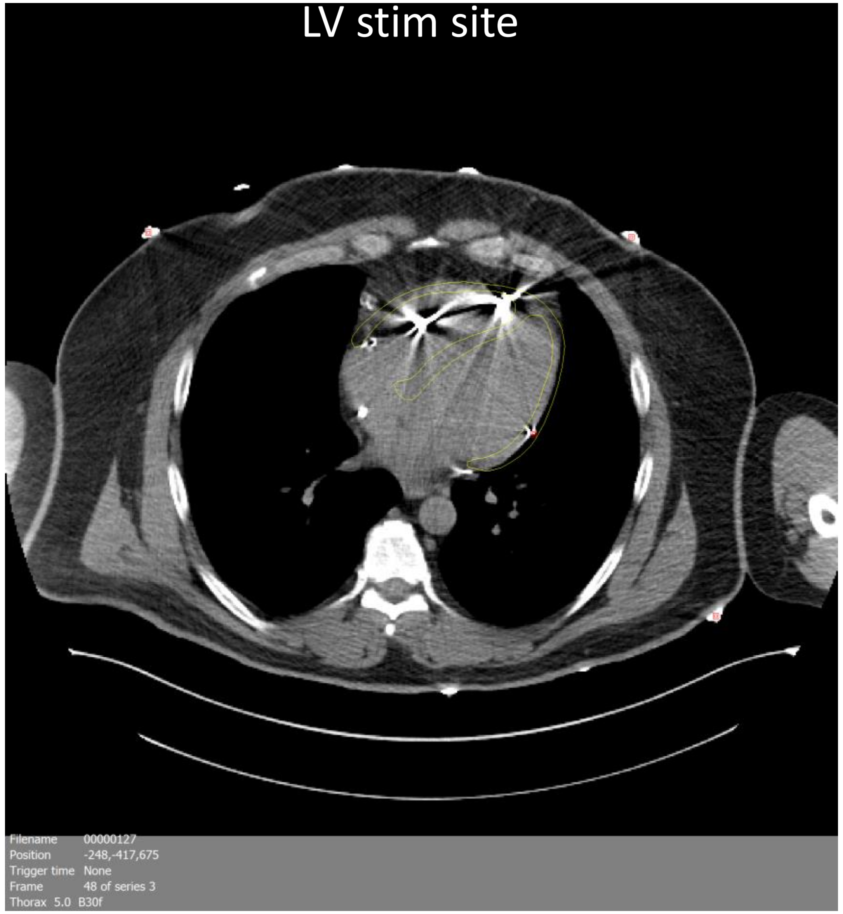
LV stim site
Filename 00000127
Position -248,-417,675
Trigger time None
Frame 48 of series 3
Thorax 5.0 B30f

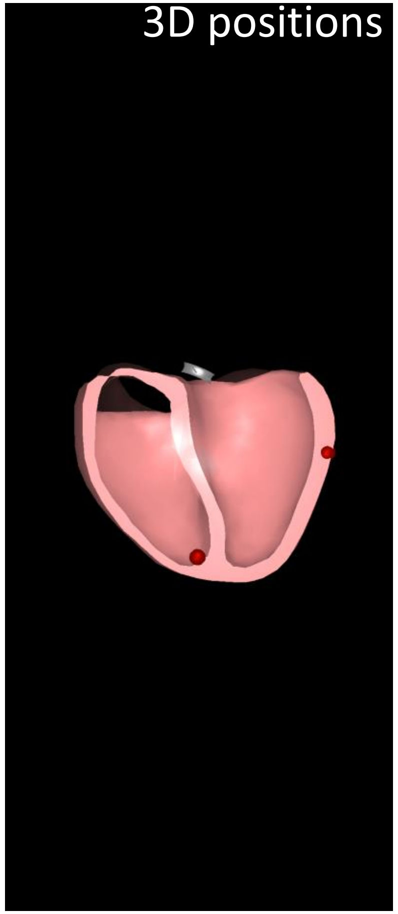
3D positions

# LV STIM localization

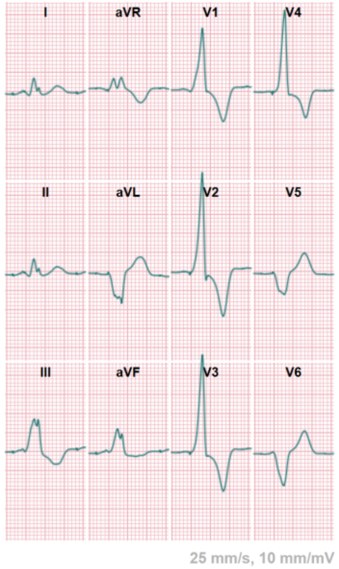


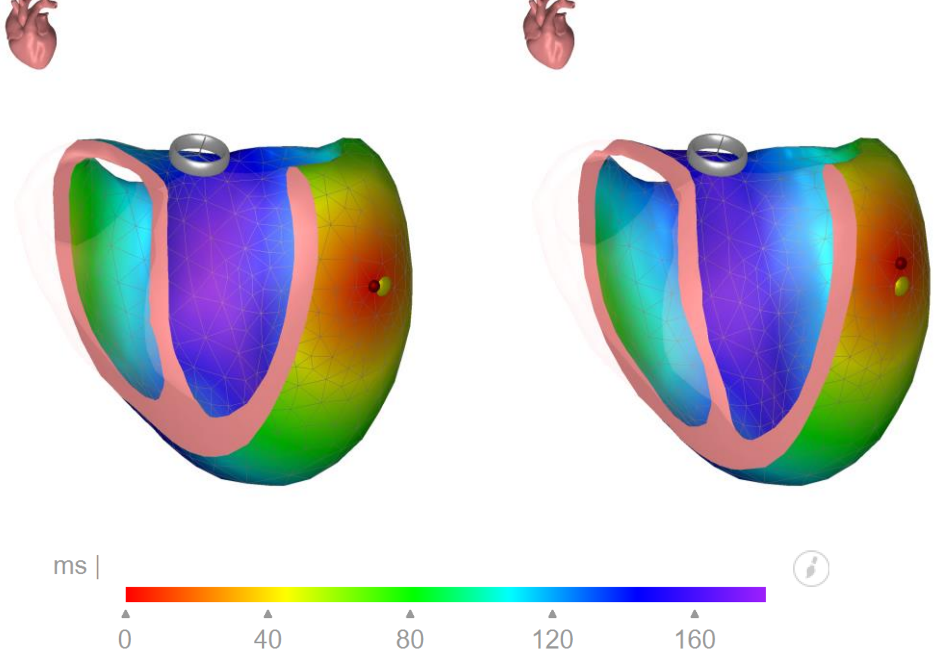


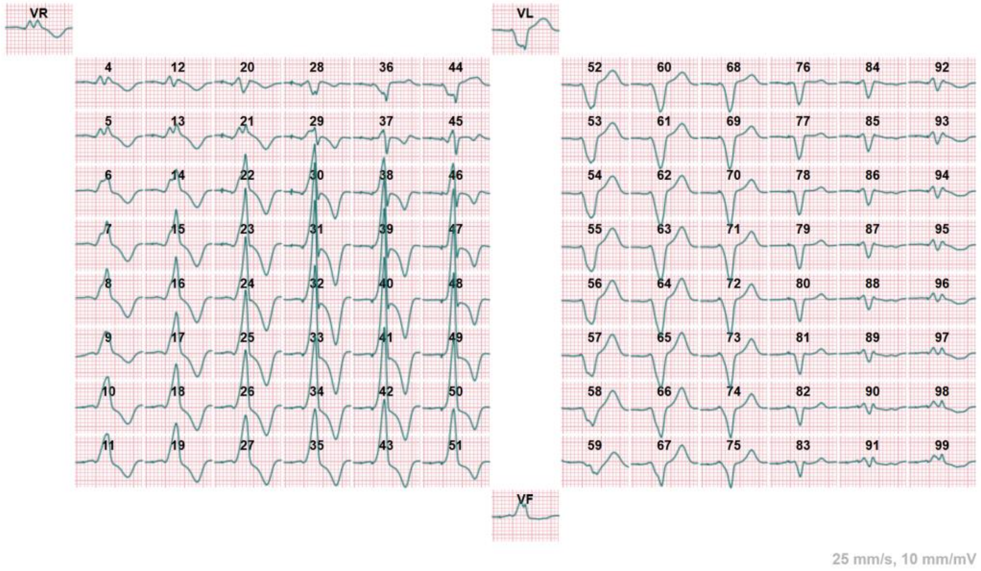


# RV STIM localization

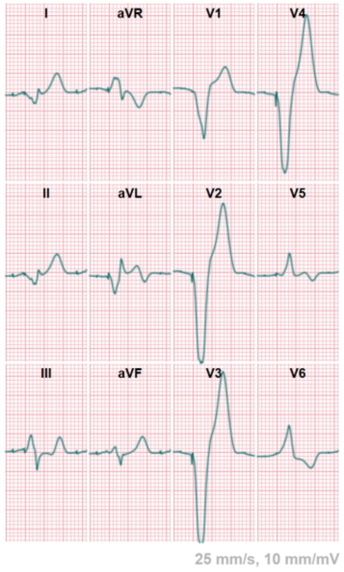


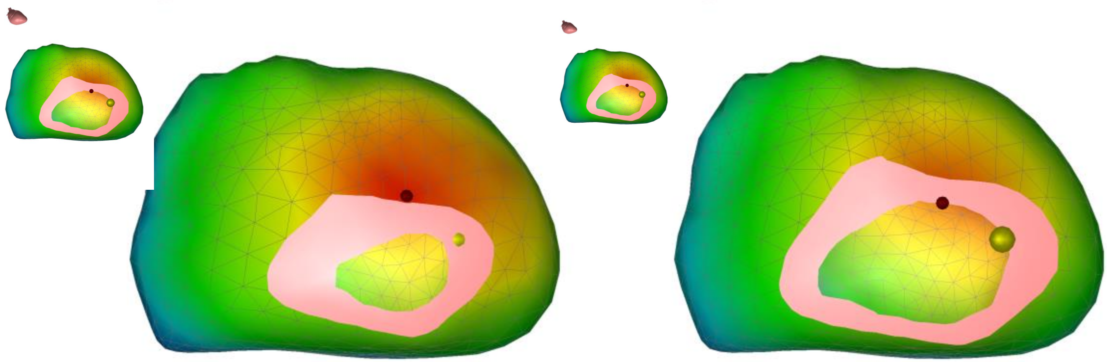

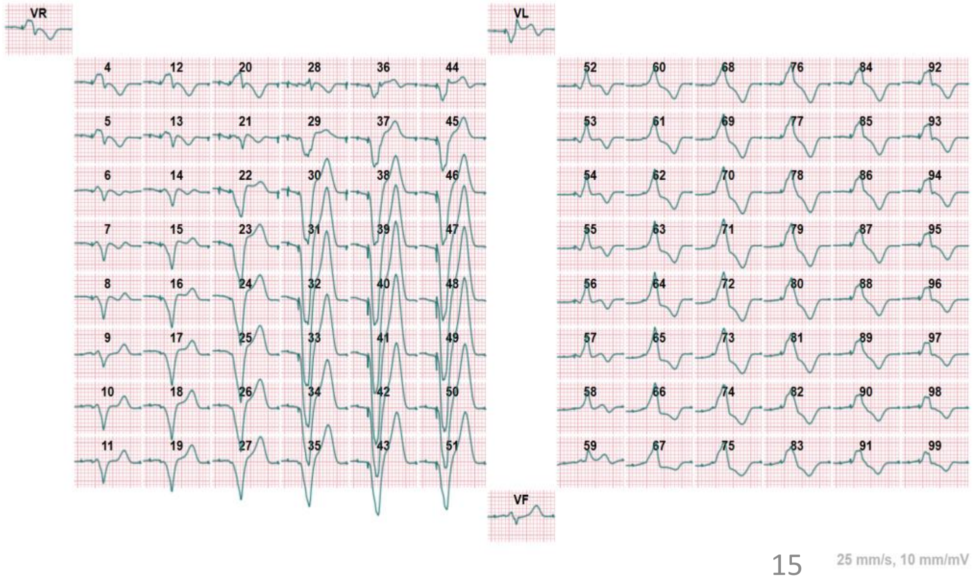

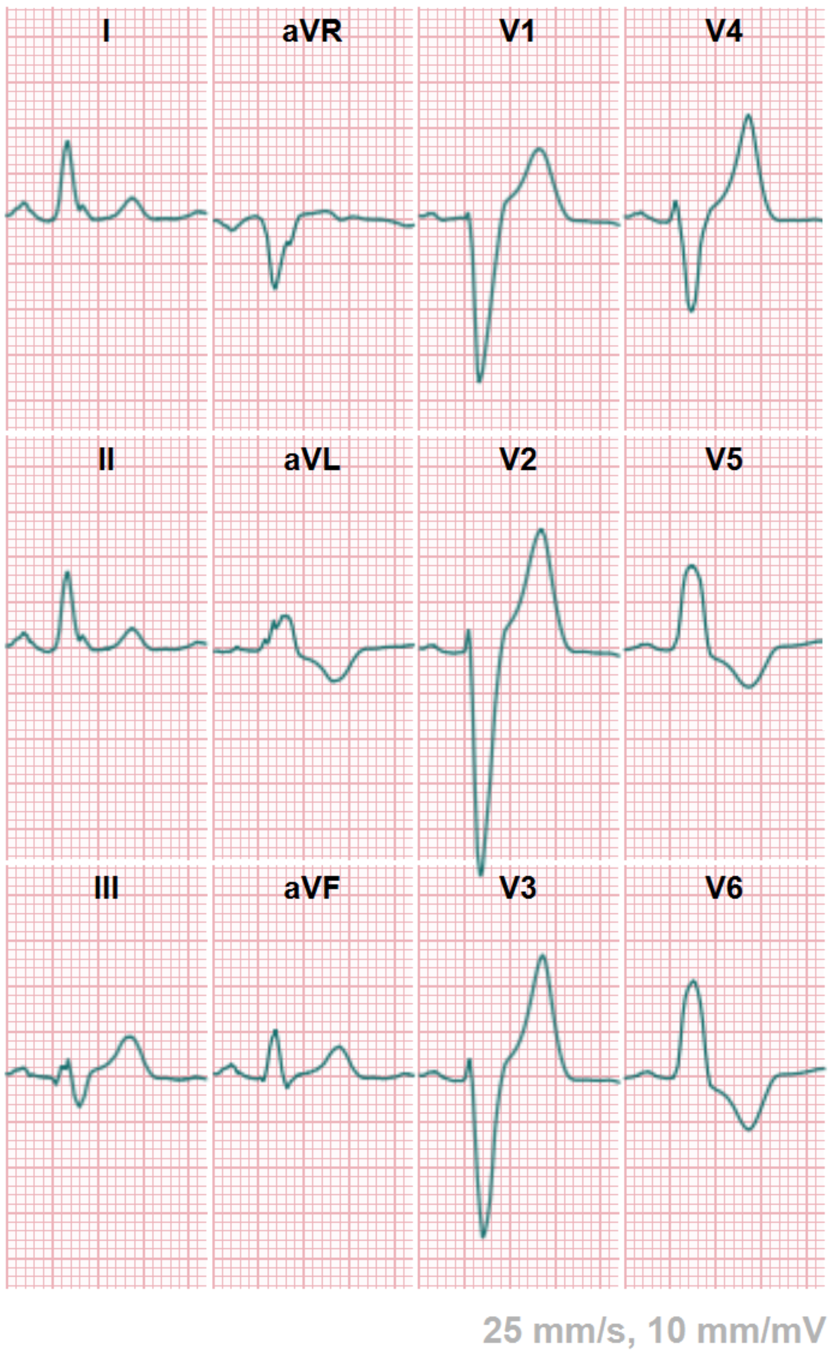


Initial

optimized

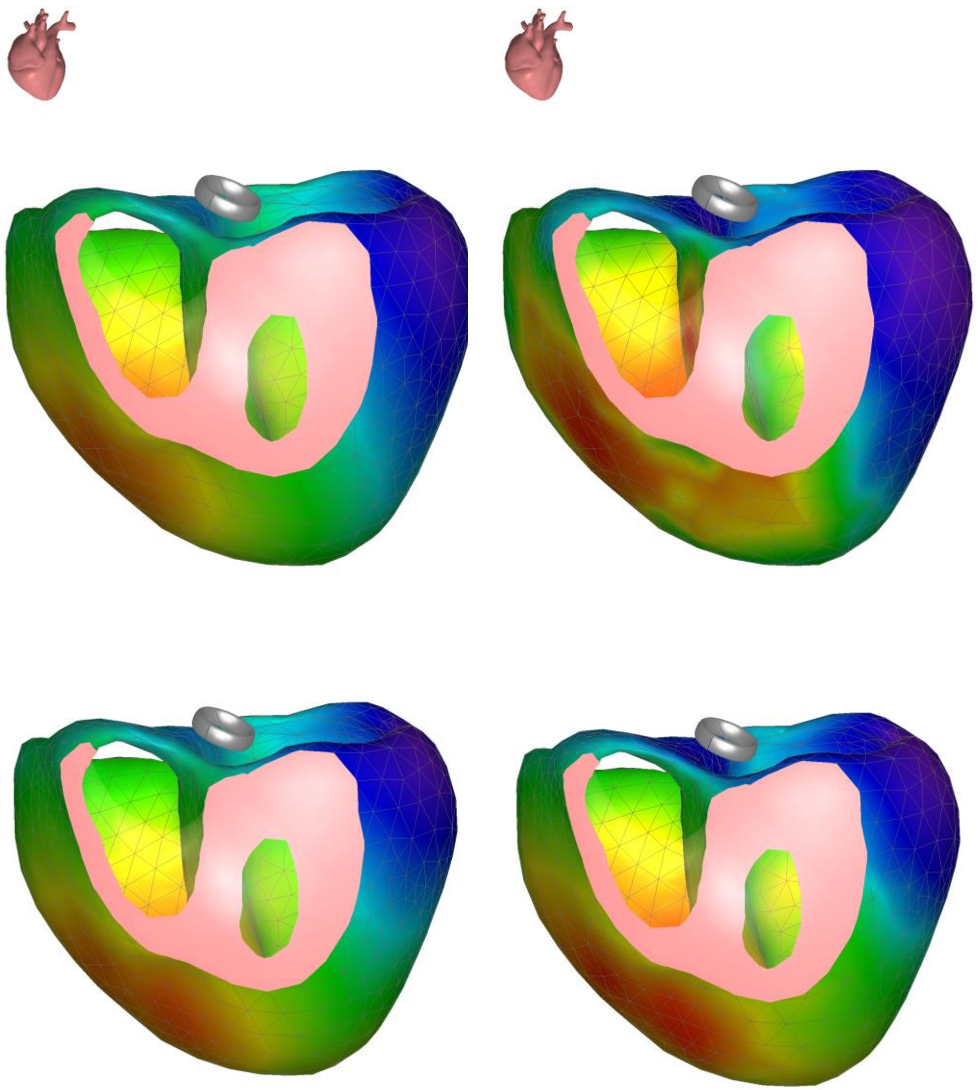

BSPM

12 lead
ECG

# Pat006

RV Stim site

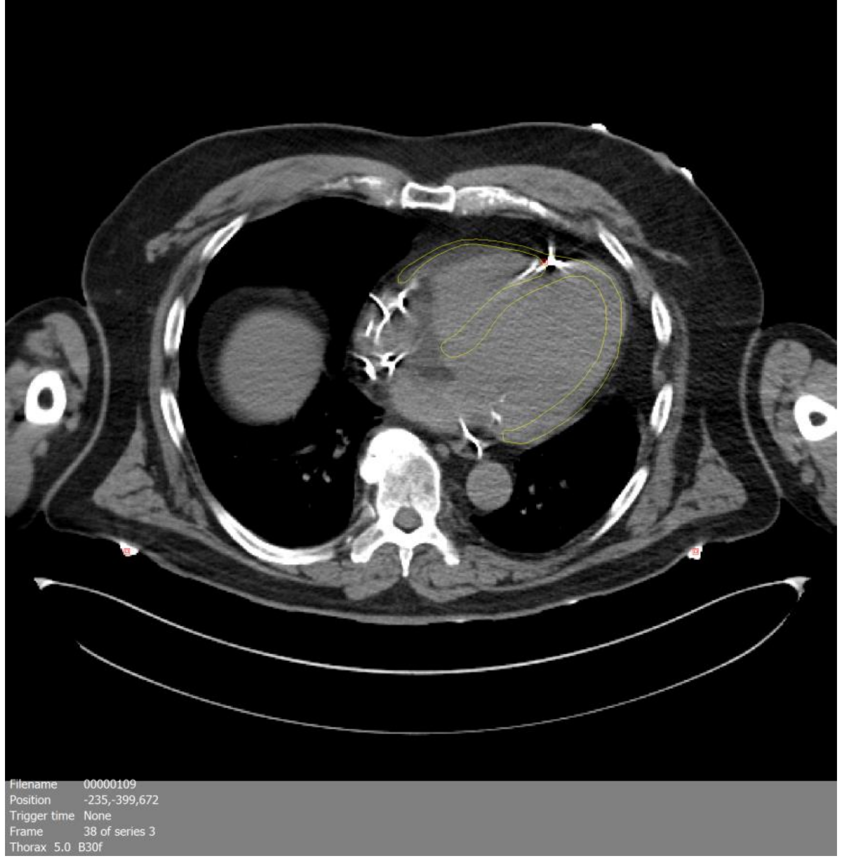


LV stim site

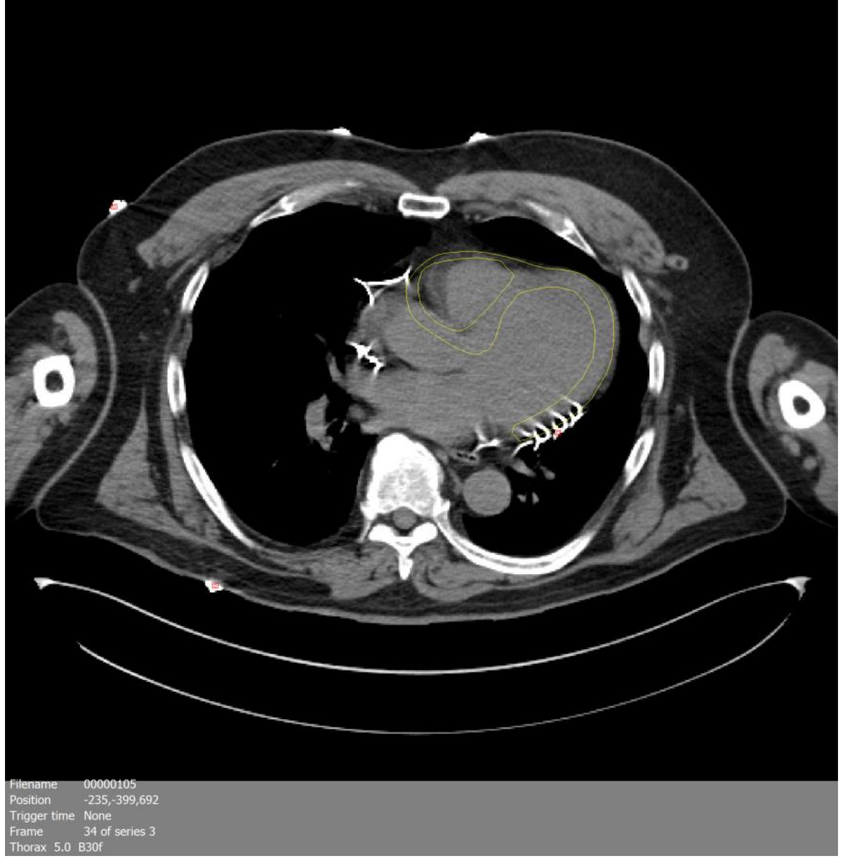


3D positions

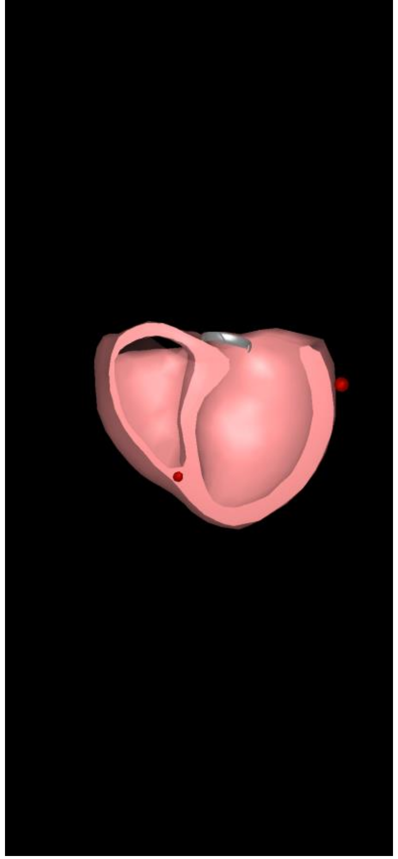

## LV STIM localization

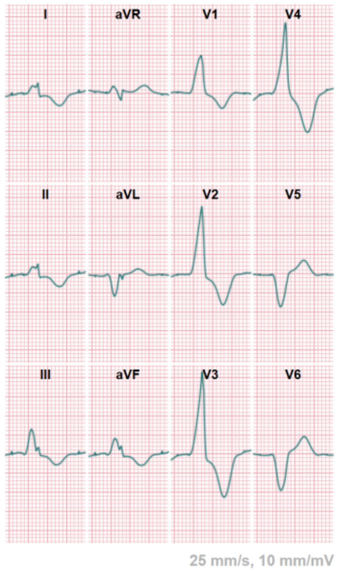


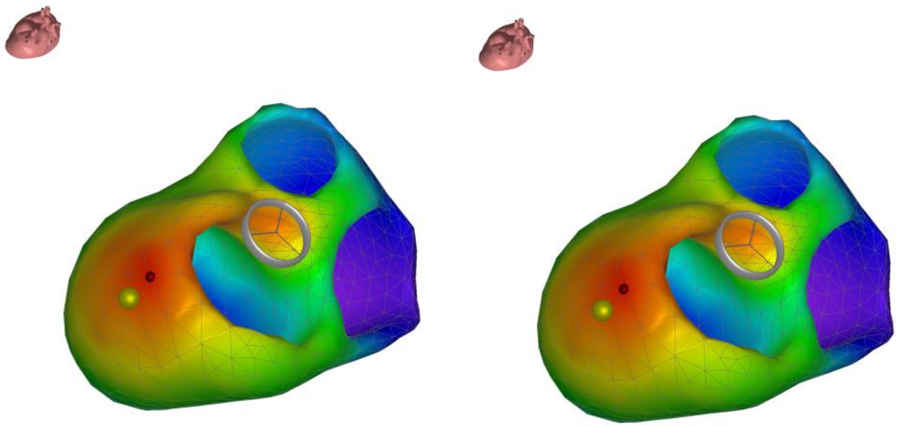

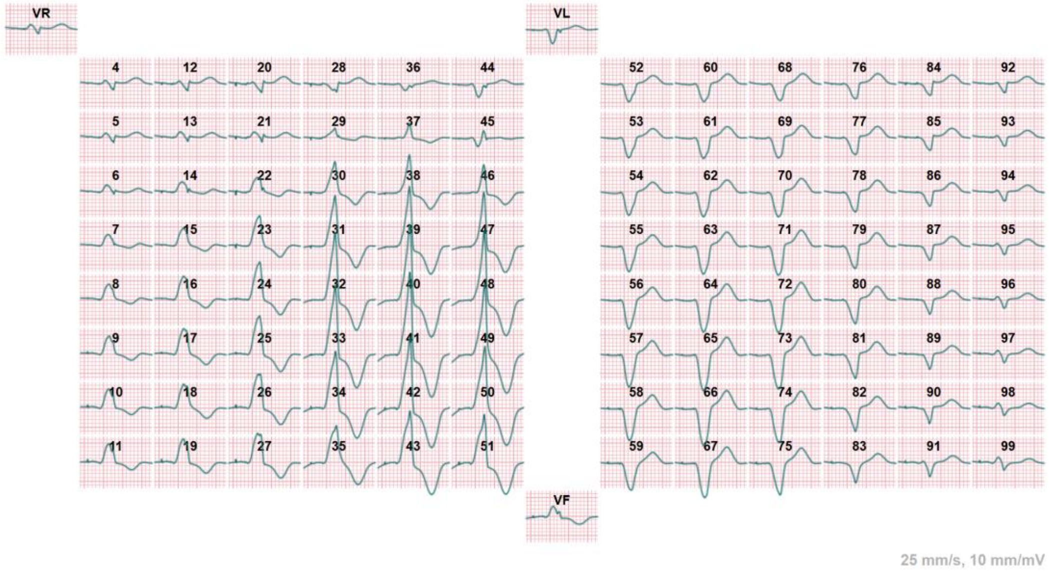


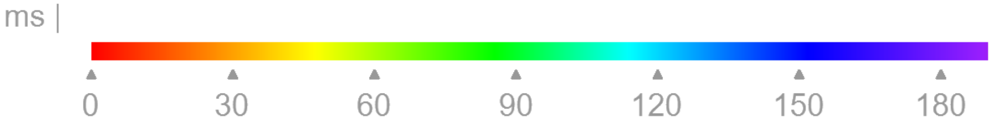


## RV STIM locali

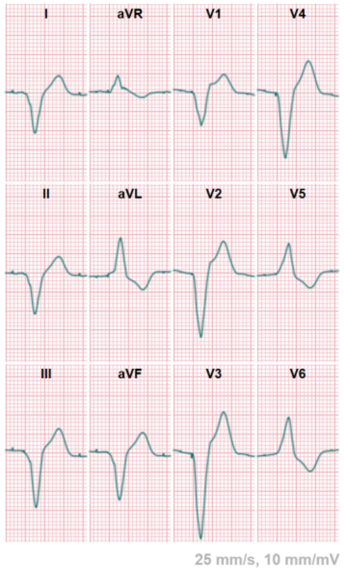


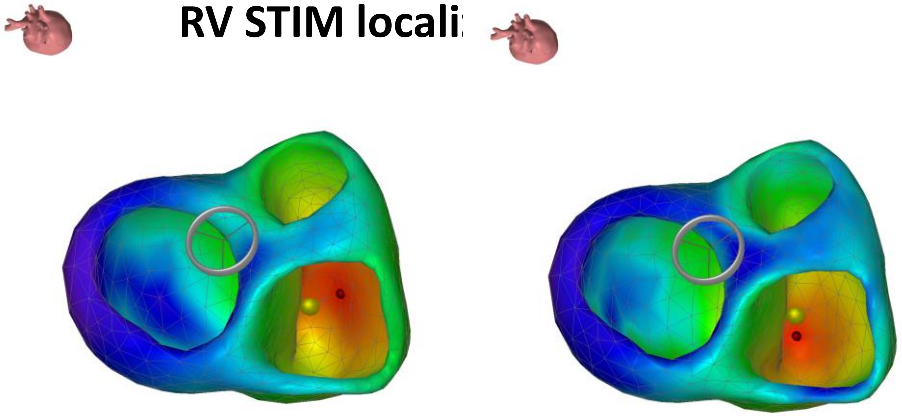

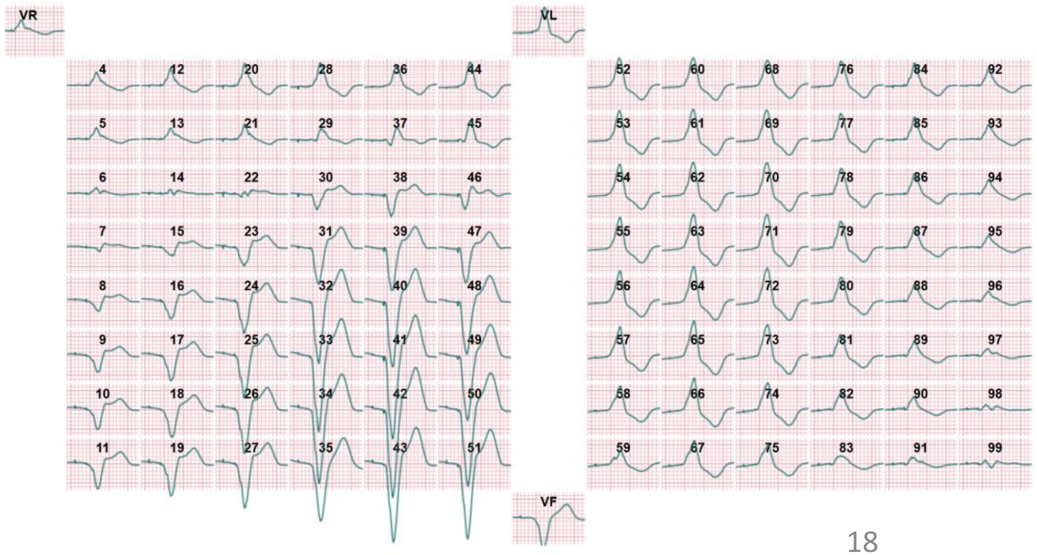

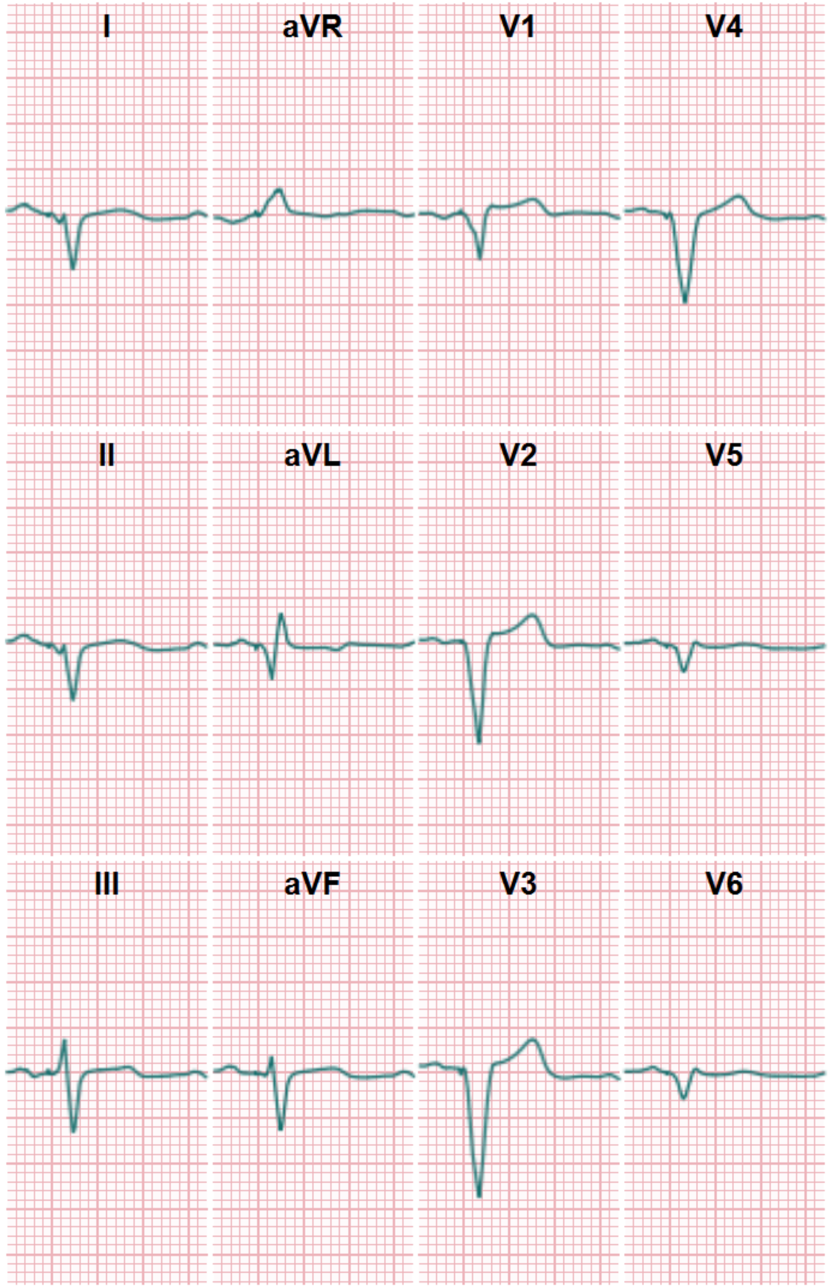


25 mm/s, 10 mm/mV

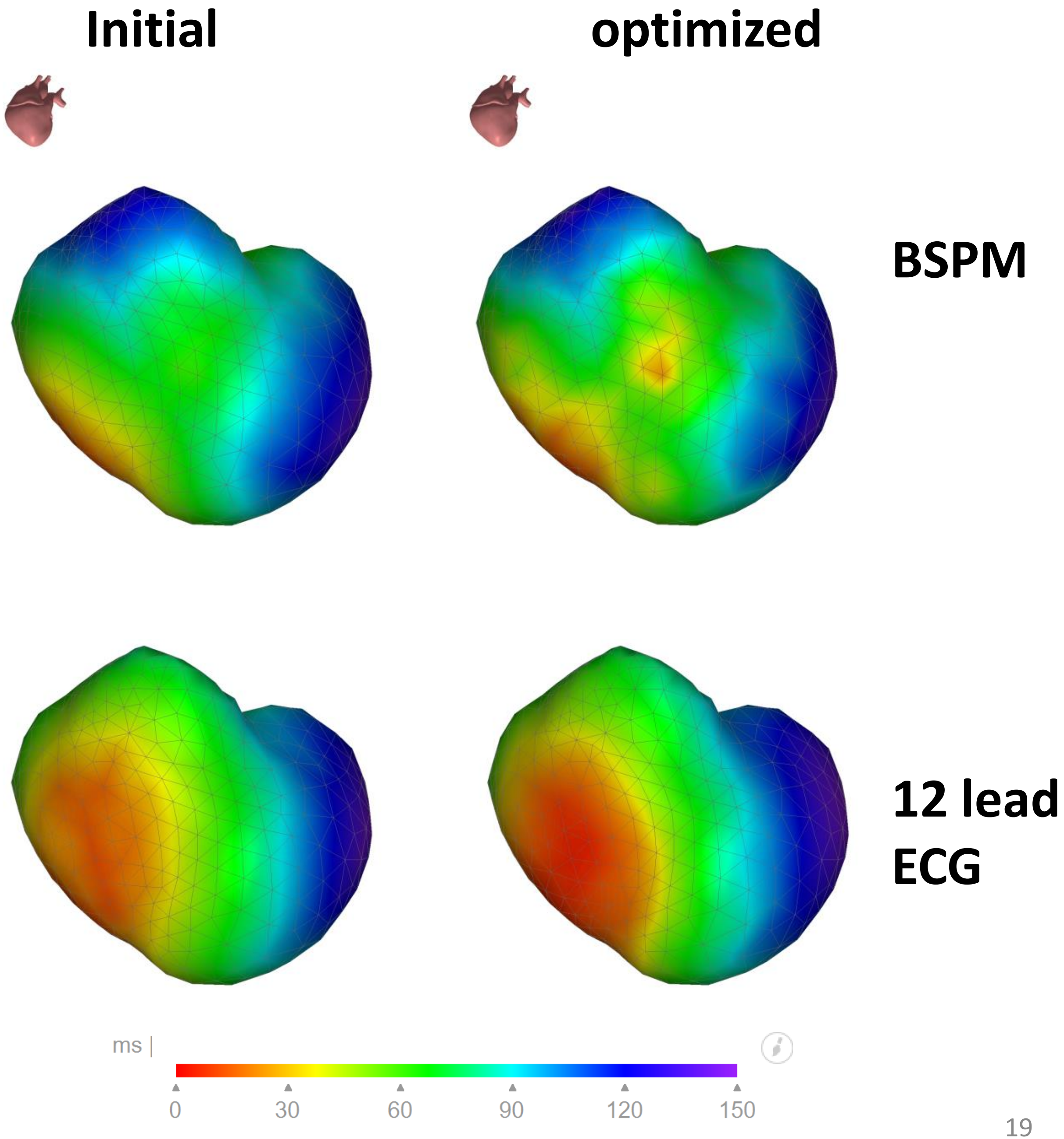

# Pat007

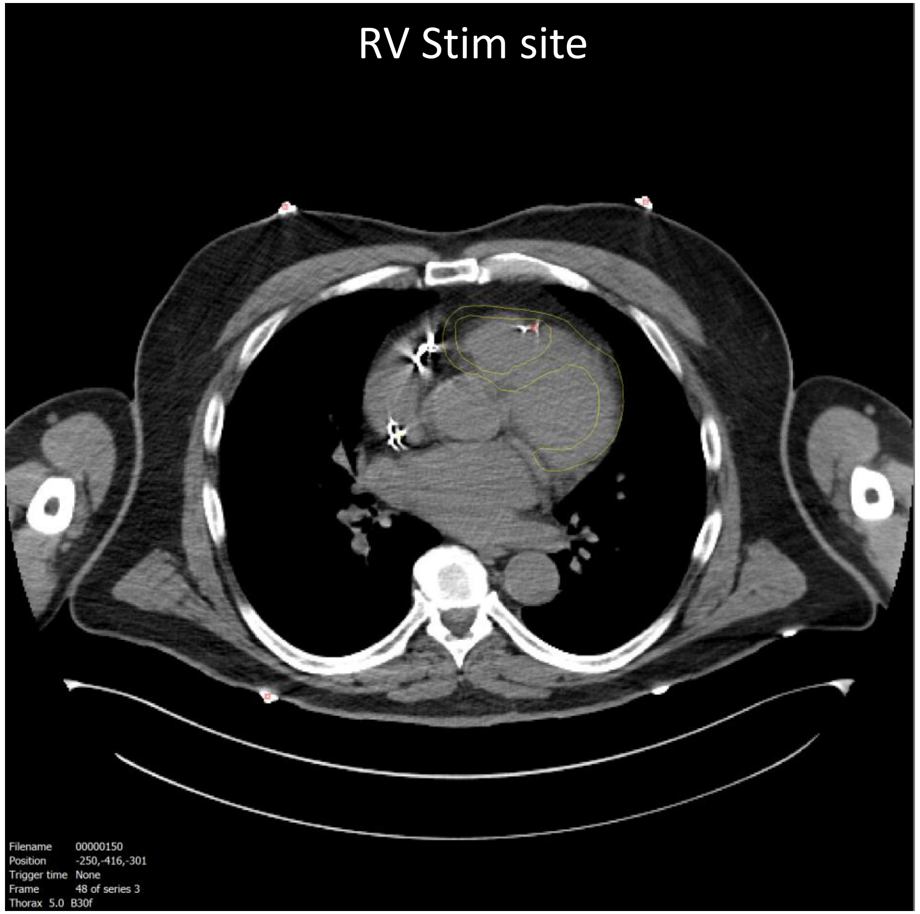
RV Stim site
Filename 00000150
Position -250,-416,-301
Trigger time None
Frame 48 of series 3
Thorax 5.0 B30f

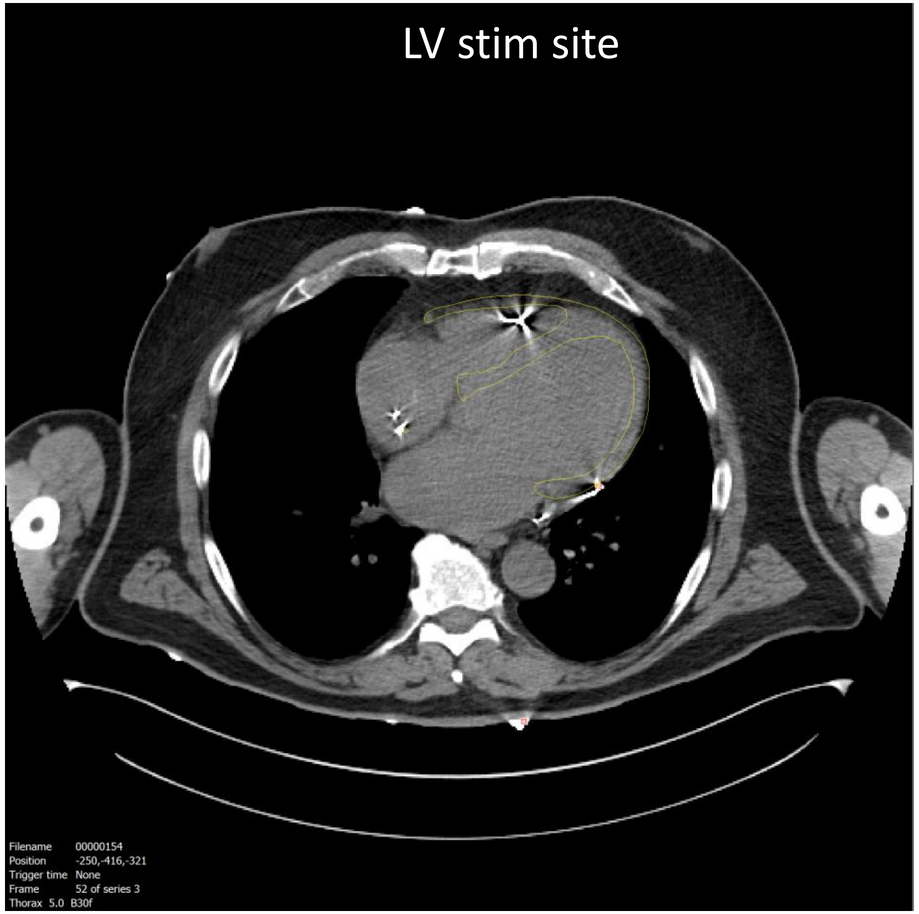
LV stim site
Filename 00000154
Position -250,-416,-321
Trigger time None
Frame 52 of series 3
Thorax 5.0 B30f

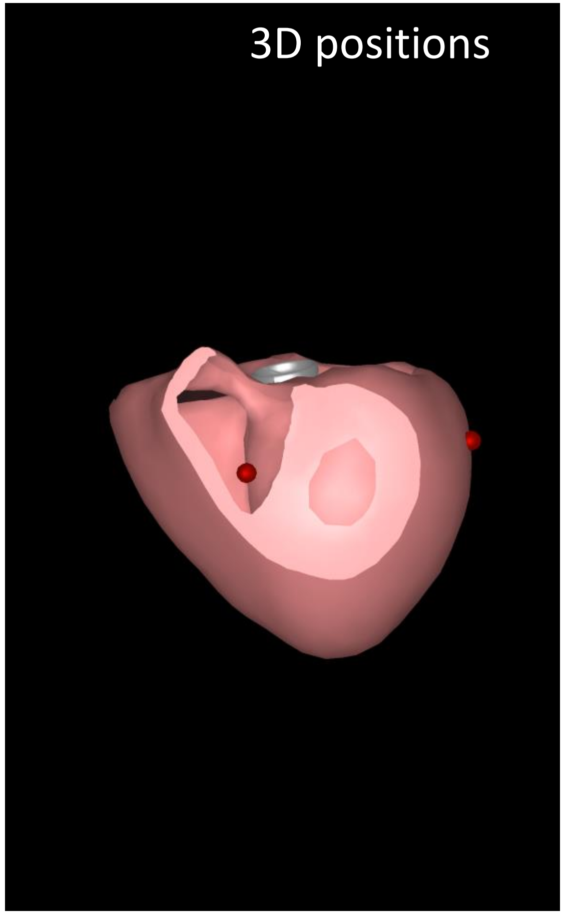
3D positions

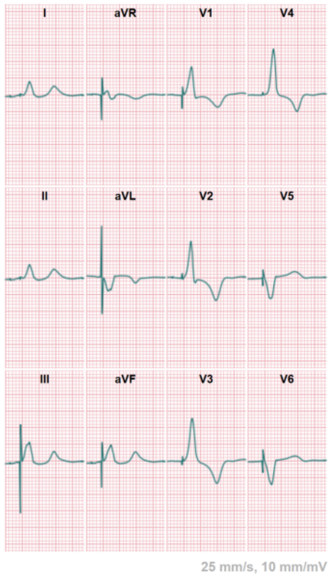


# LV STIM localization

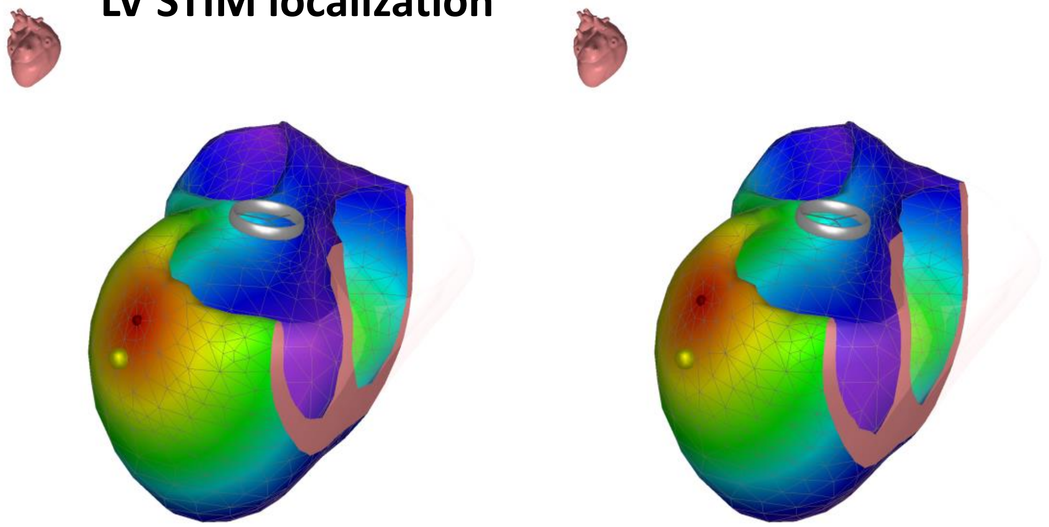

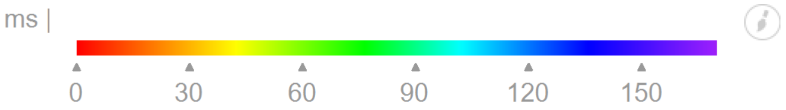


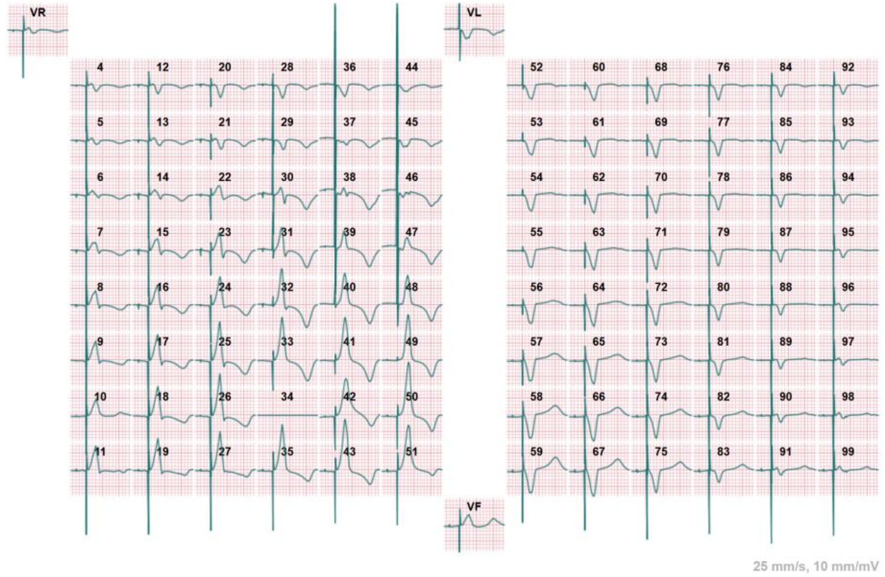


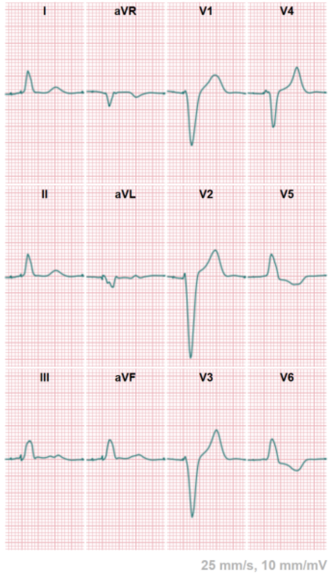


# RV STIM localization

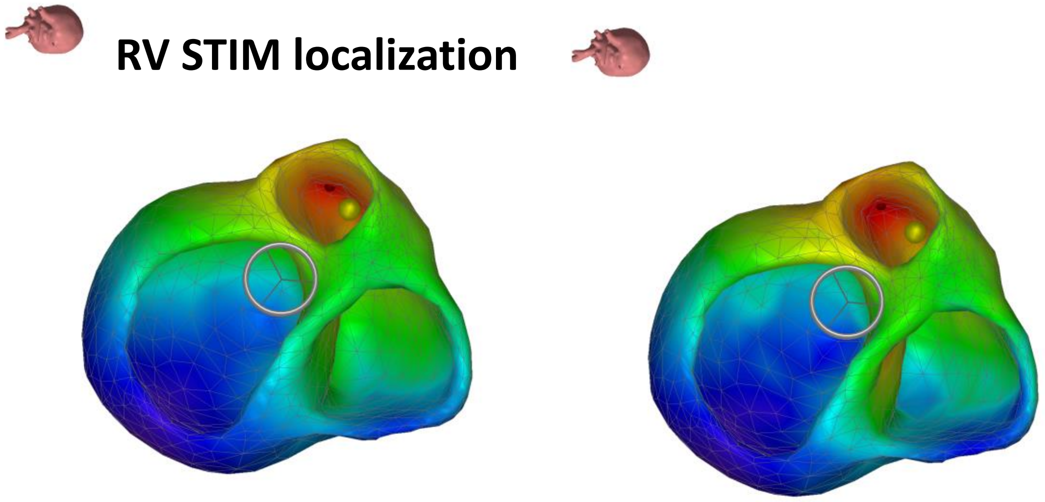

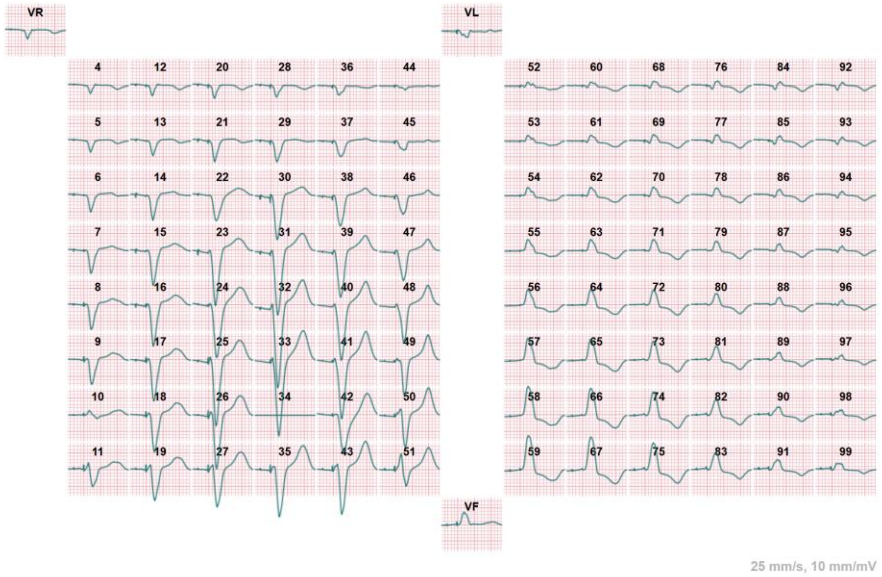

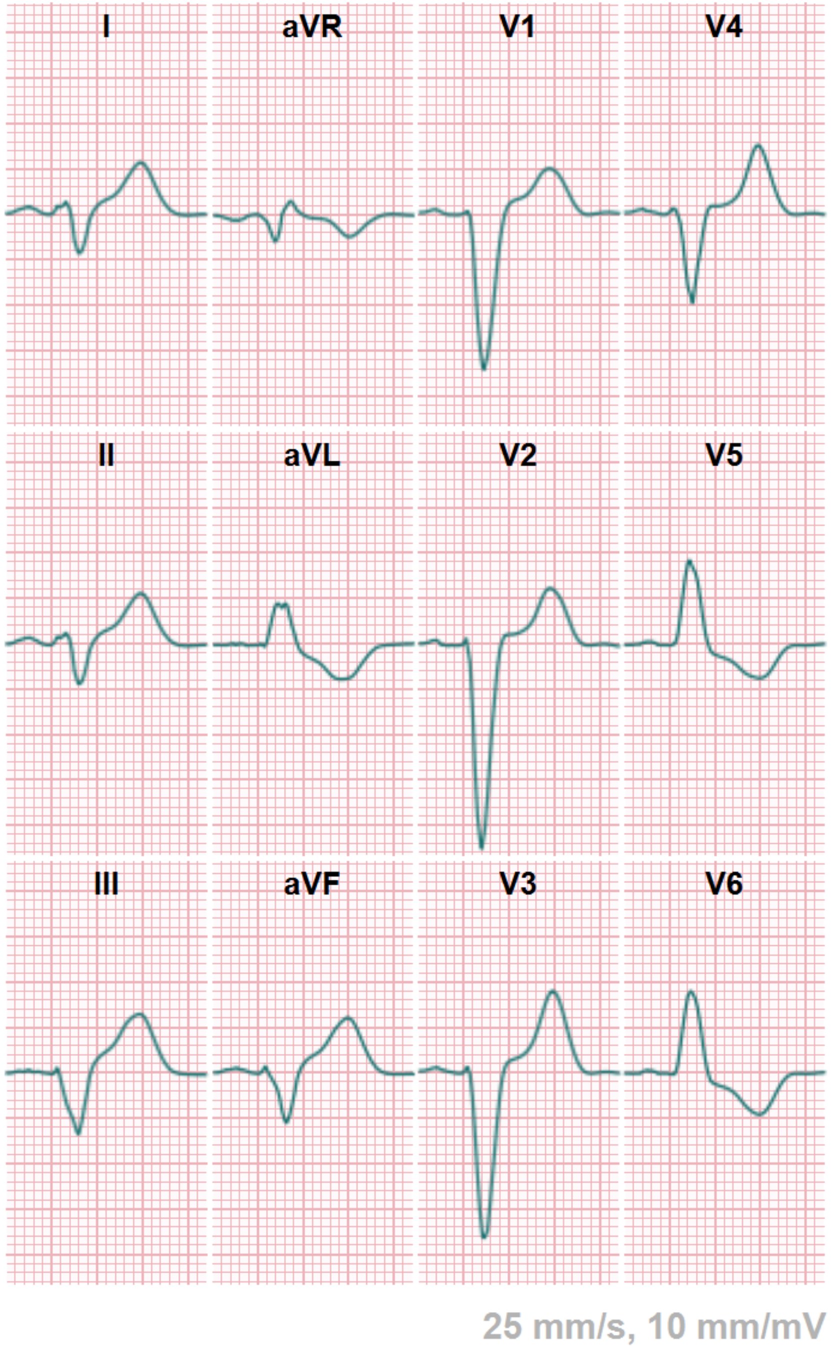
I
aVR
V1
V4
II
aVL
V2
V5
III
aVF
V3
V6
25 mm/s, 10 mm/mV

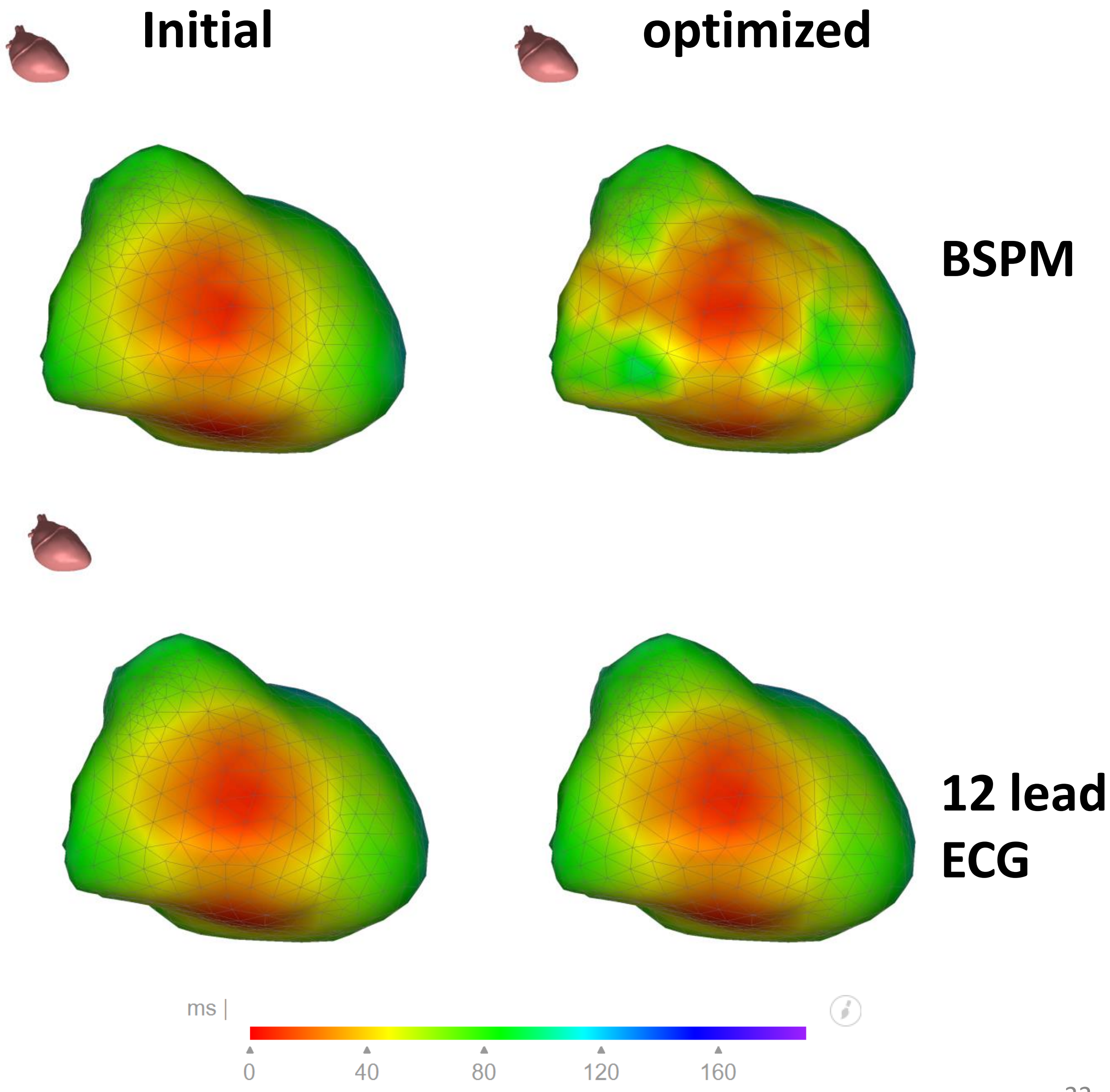
Initial
optimized
BSPM
12 lead ECG
ms |
0
40
80
120
160

# Pat008

RV Stim site

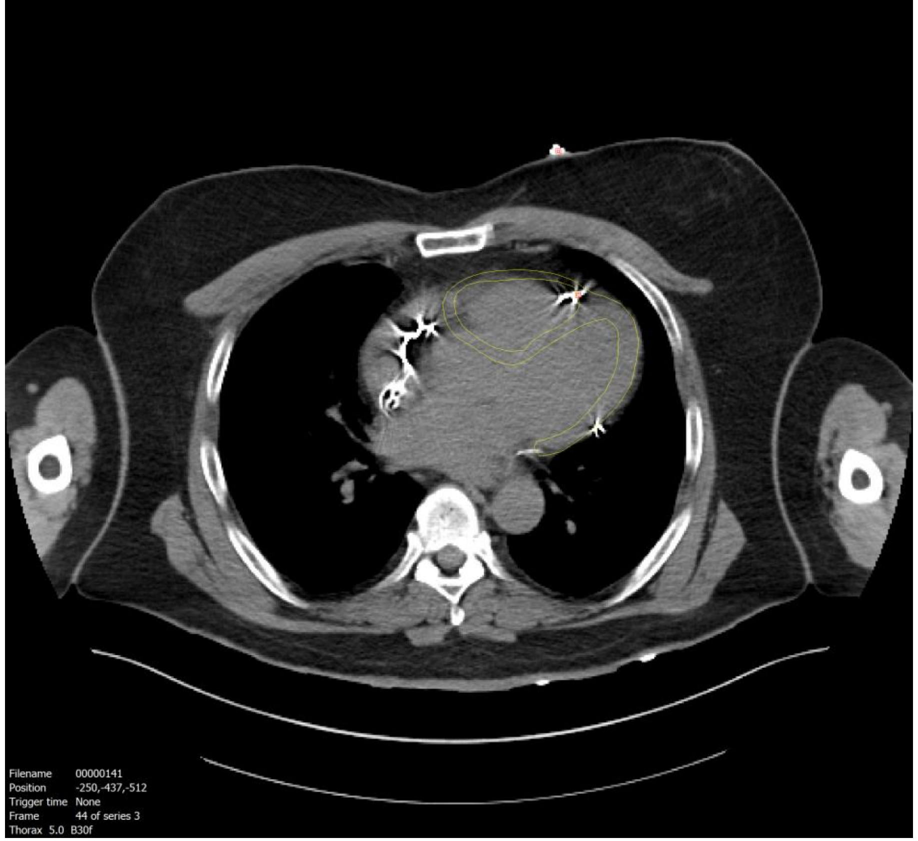


LV stim site

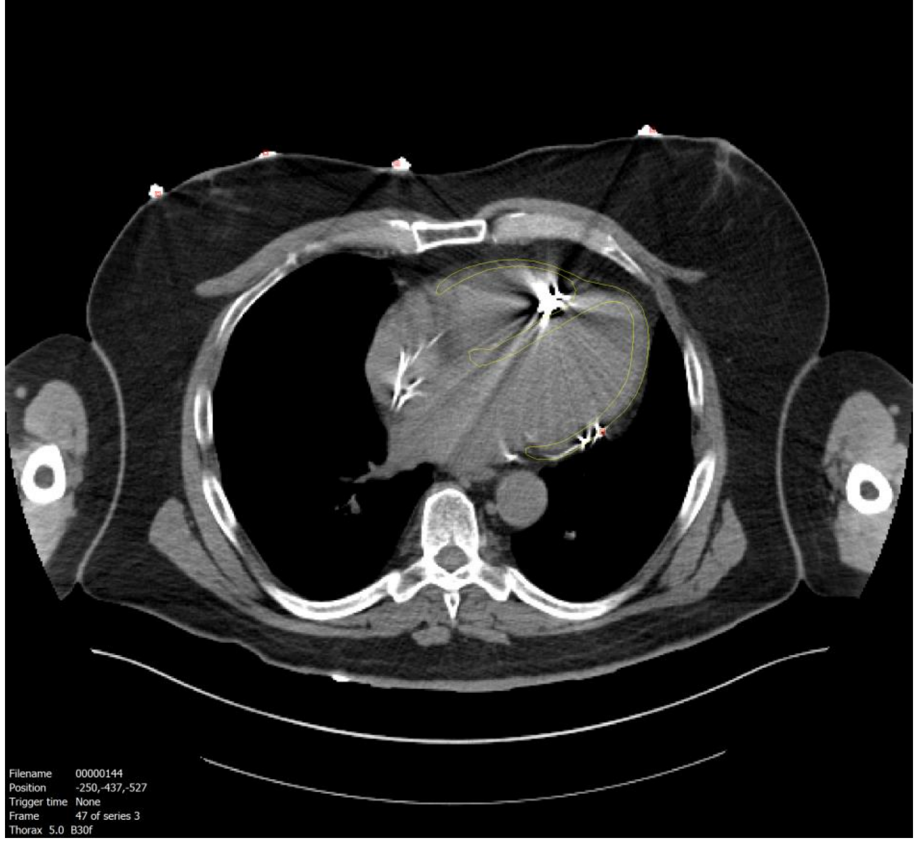


3D positions

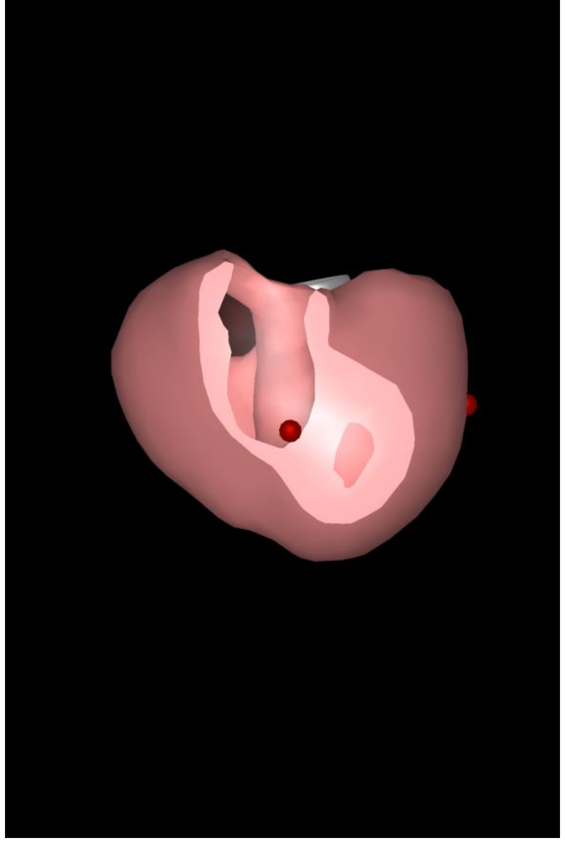

# LV STIM localization

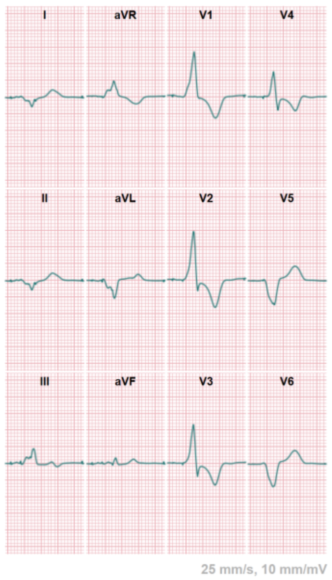


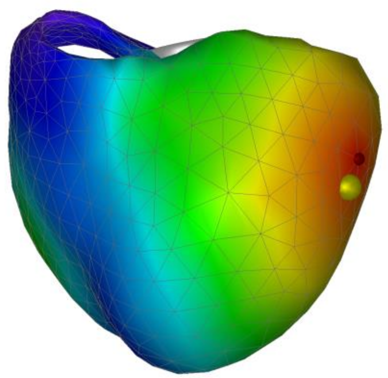

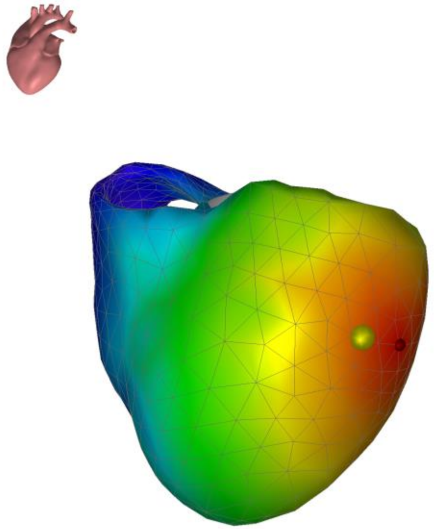

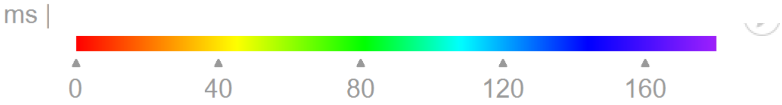


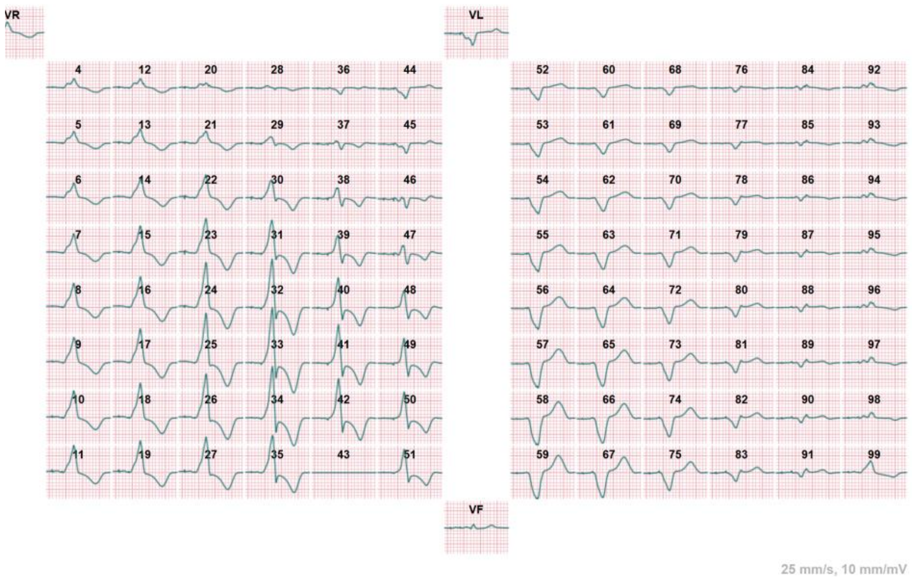

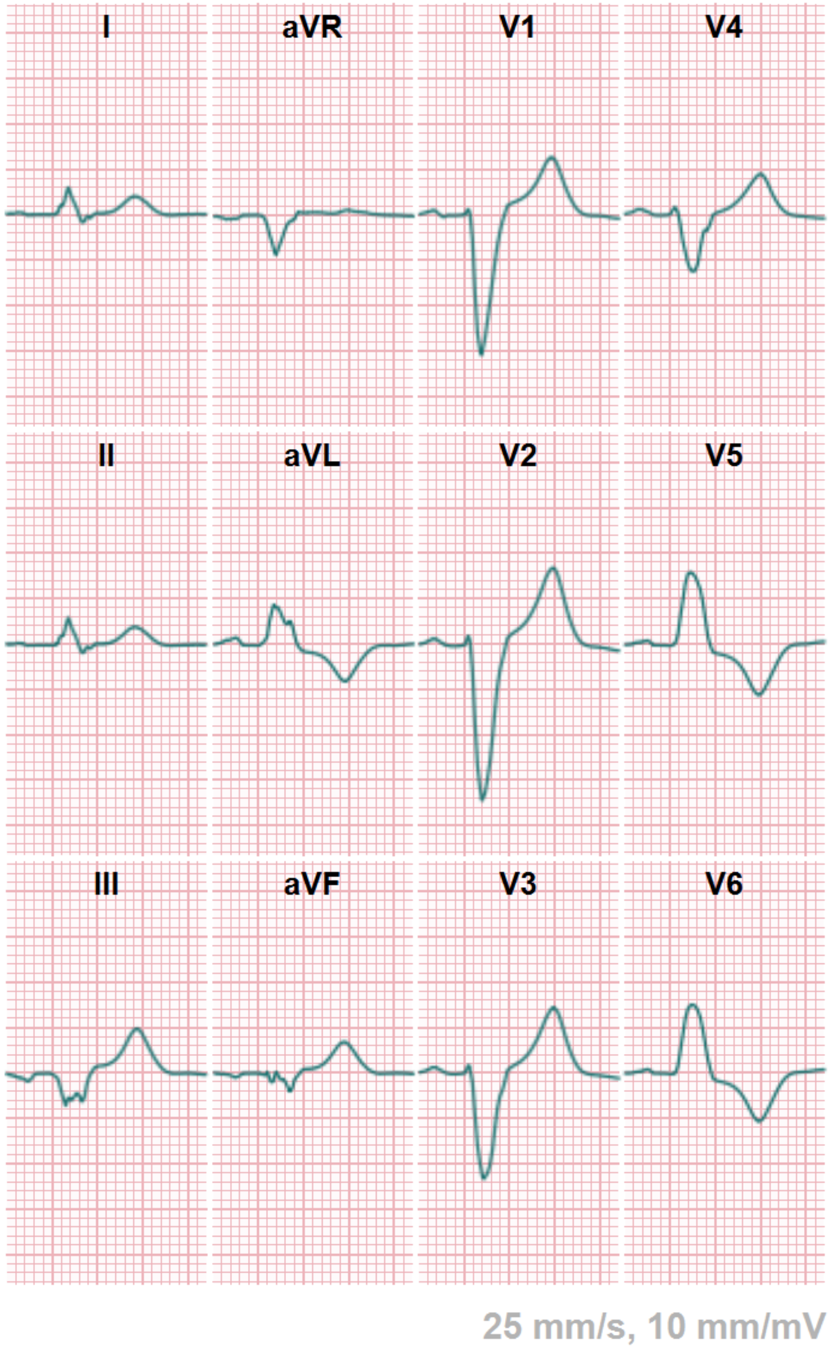
I
aVR
V1
V4
II
aVL
V2
V5
III
aVF
V3
V6
25 mm/s, 10 mm/mV

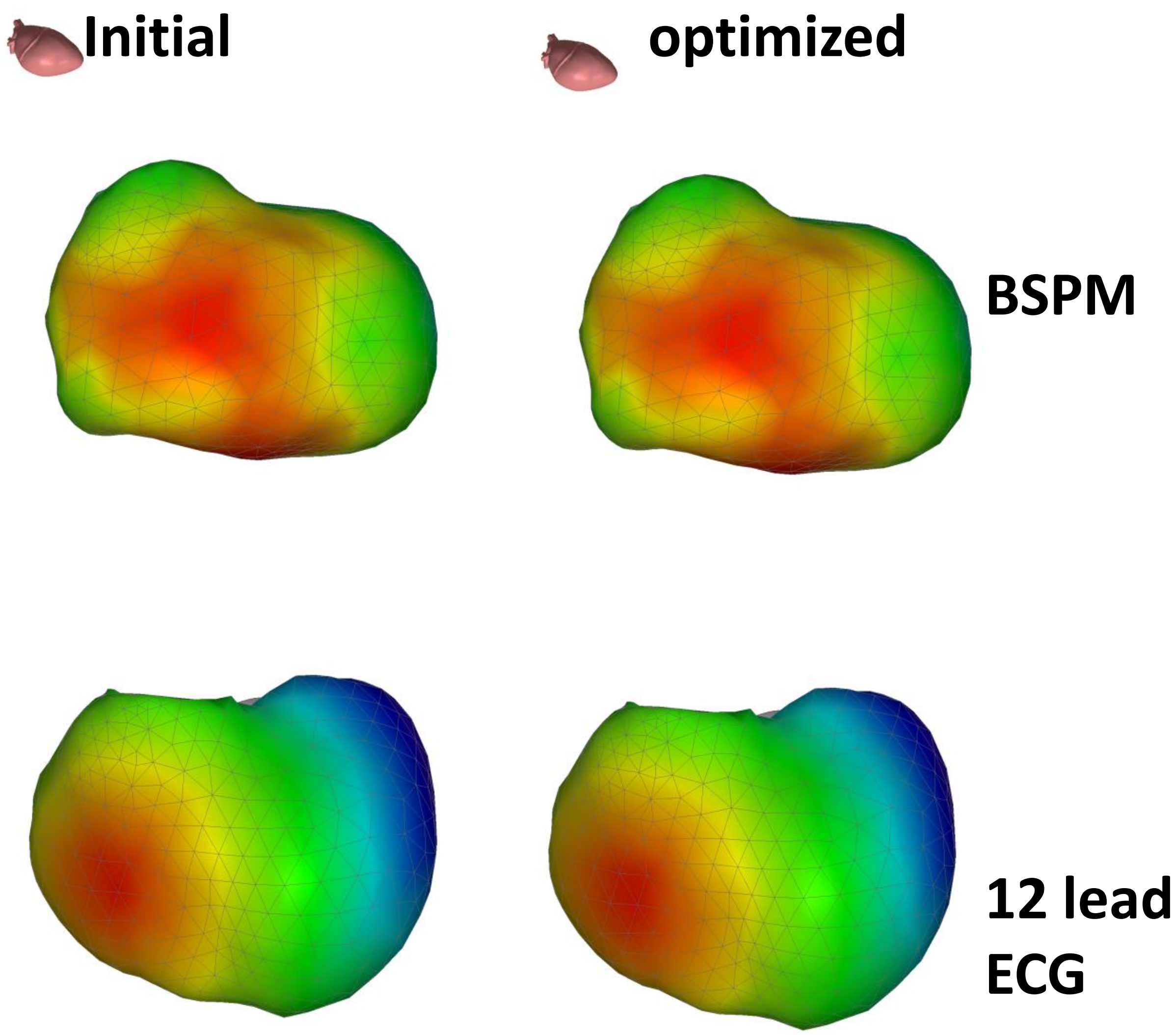
Initial
optimized
BSPM
12 lead
ECG

# Pat009

RV Stim site

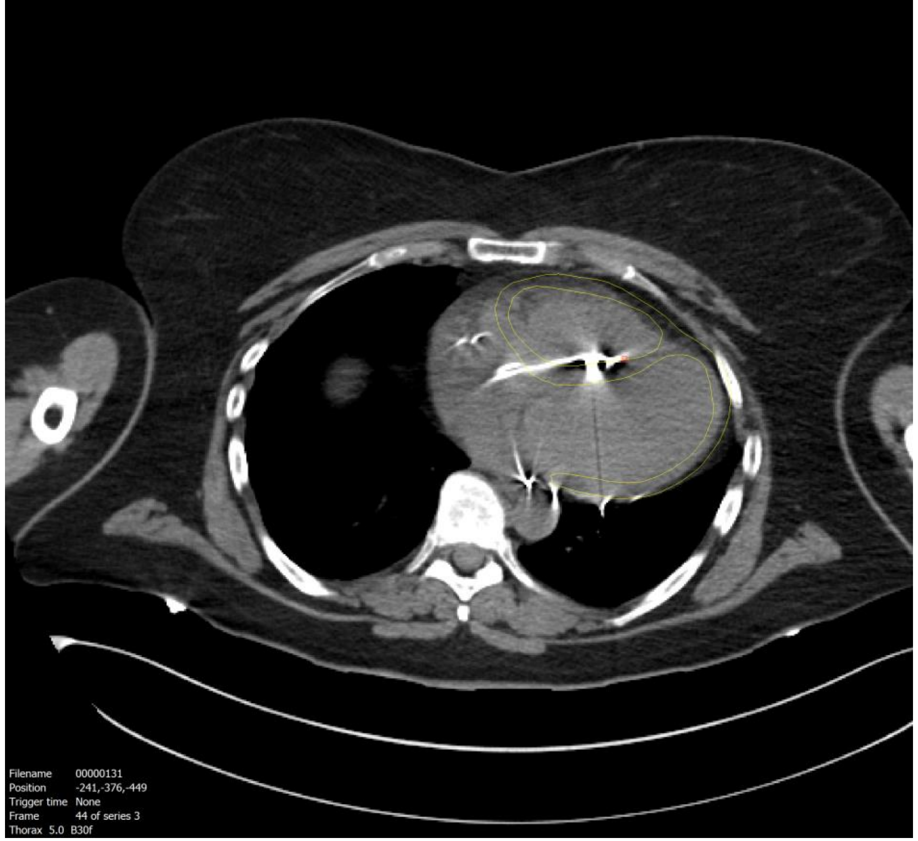


LV stim site

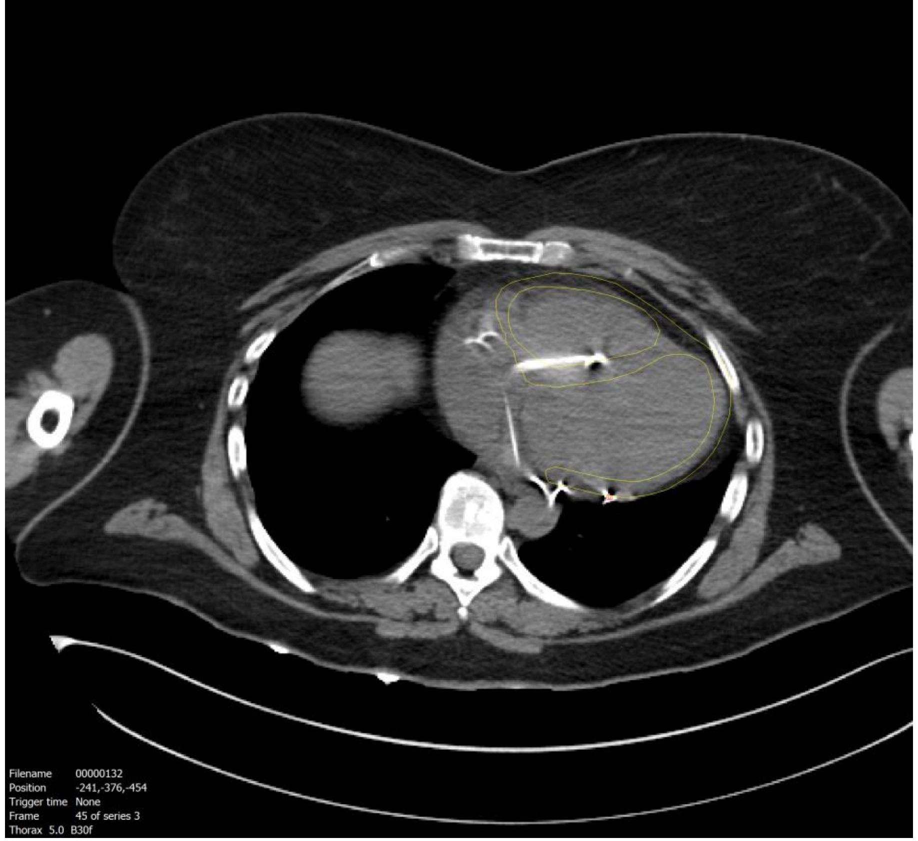


3D positions

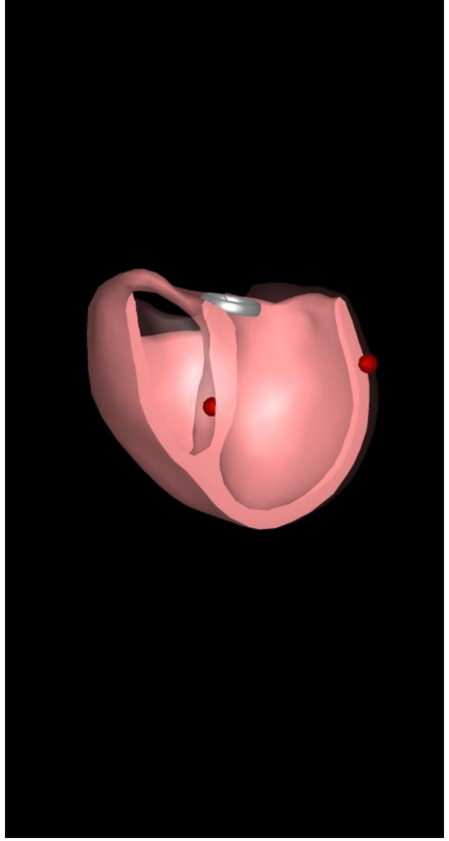

## LV STIM localization

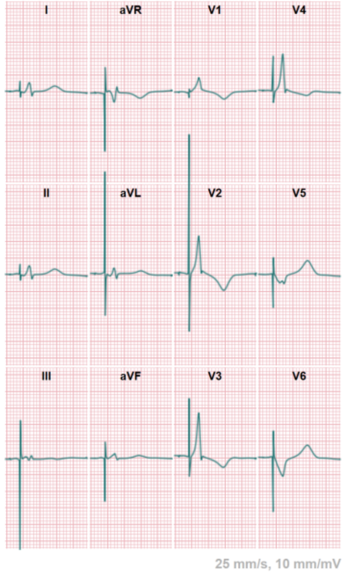


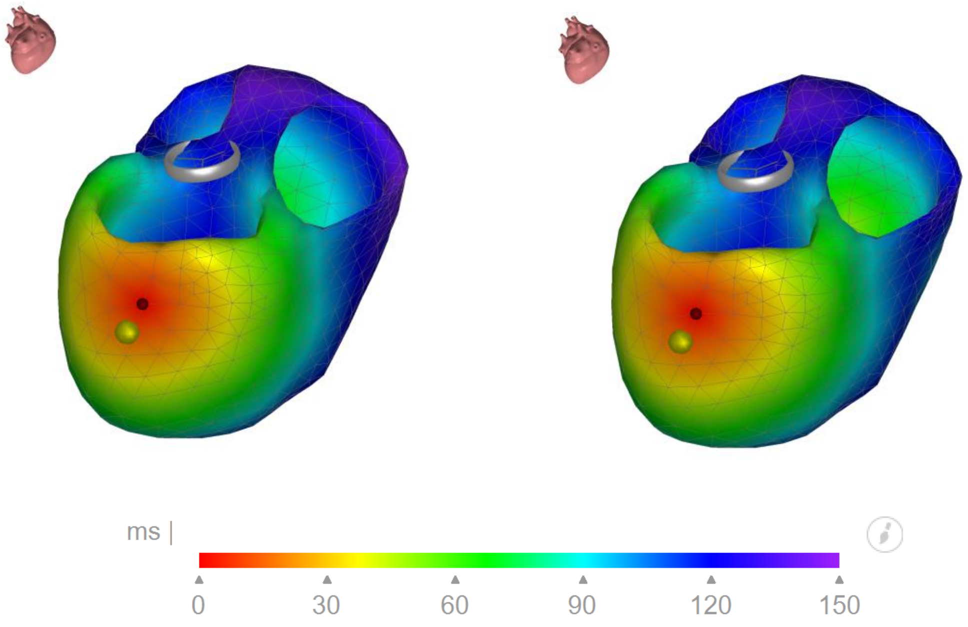


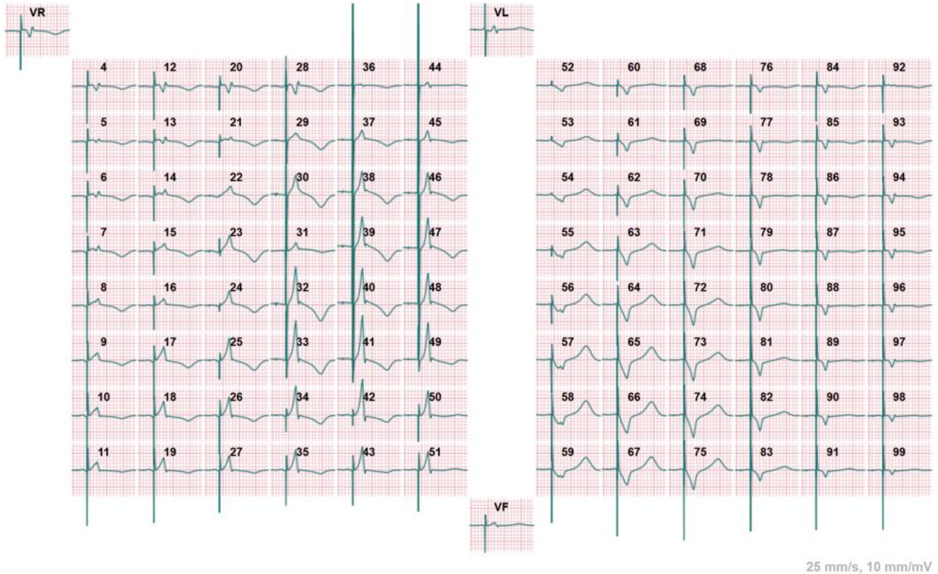


## RV STIM localization

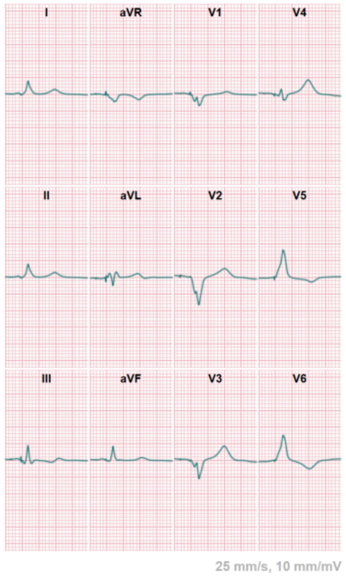


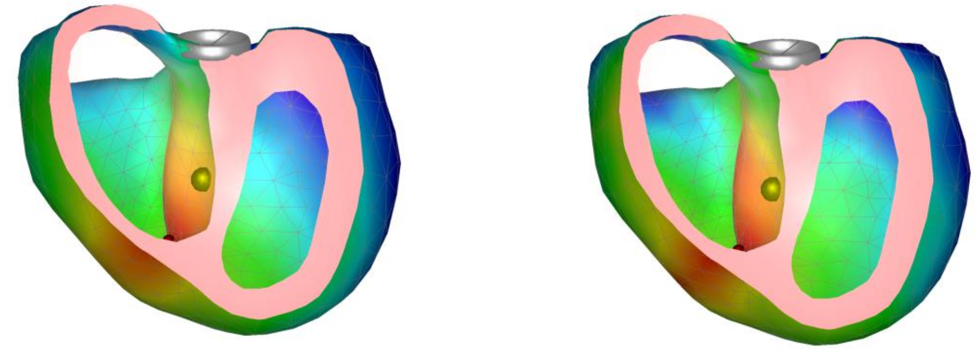

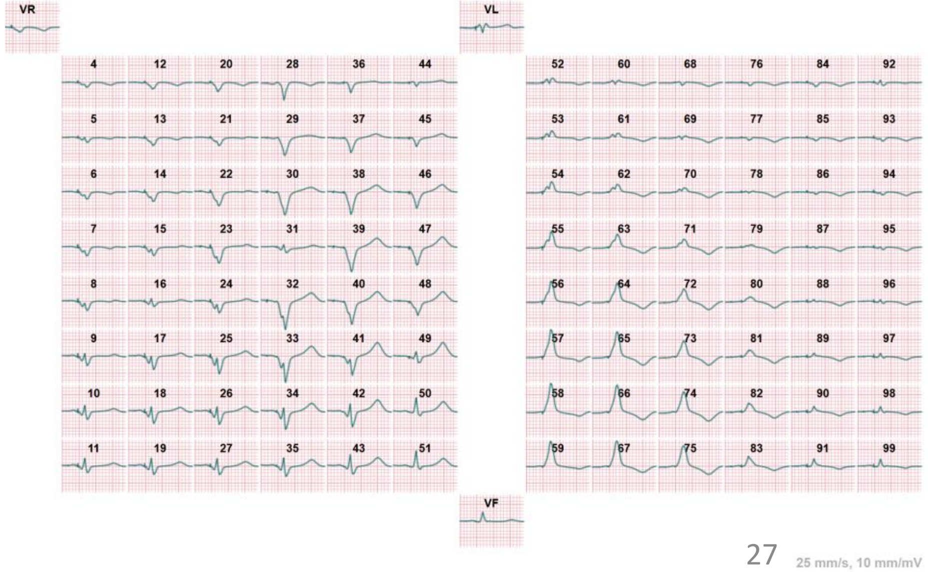

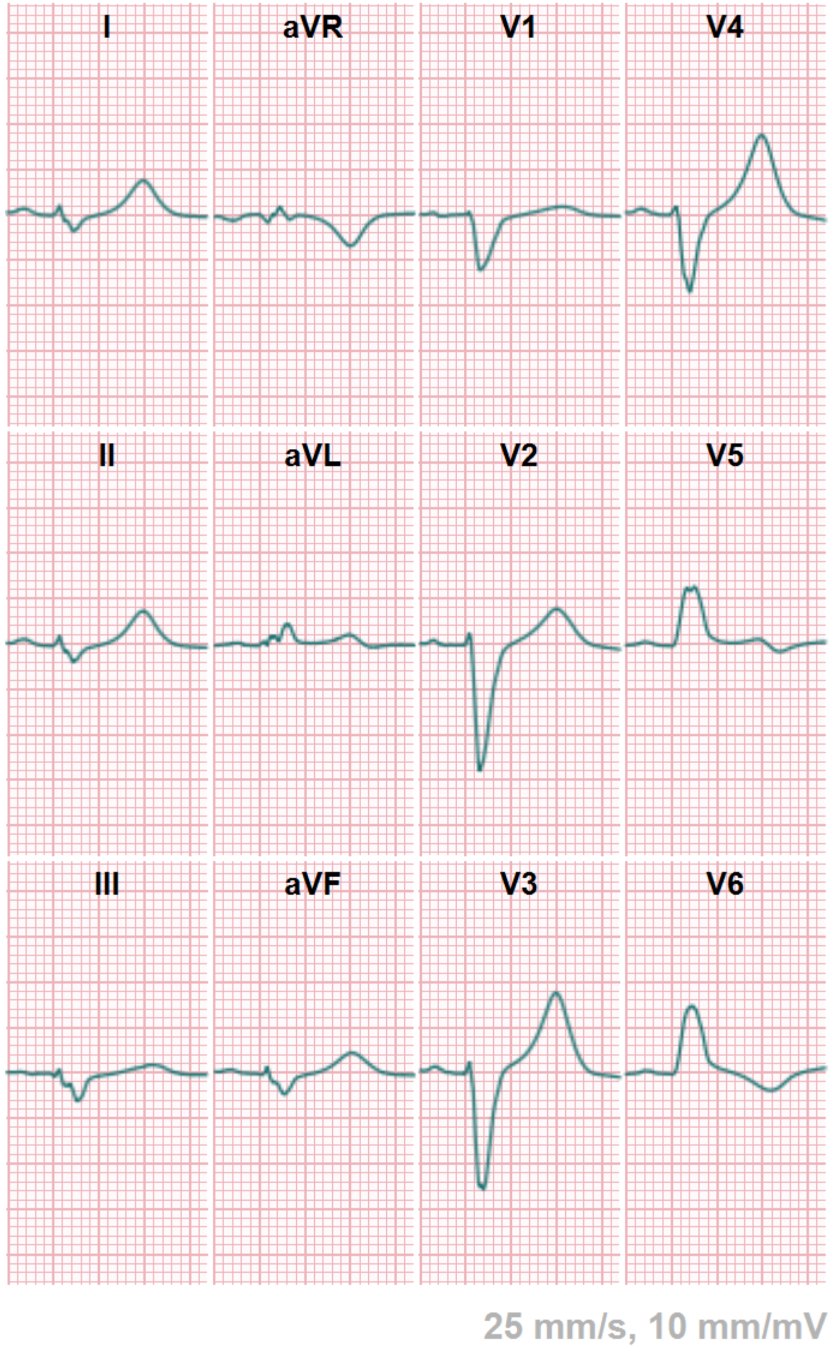
I
aVR
V1
V4
II
aVL
V2
V5
III
aVF
V3
V6
25 mm/s, 10 mm/mV

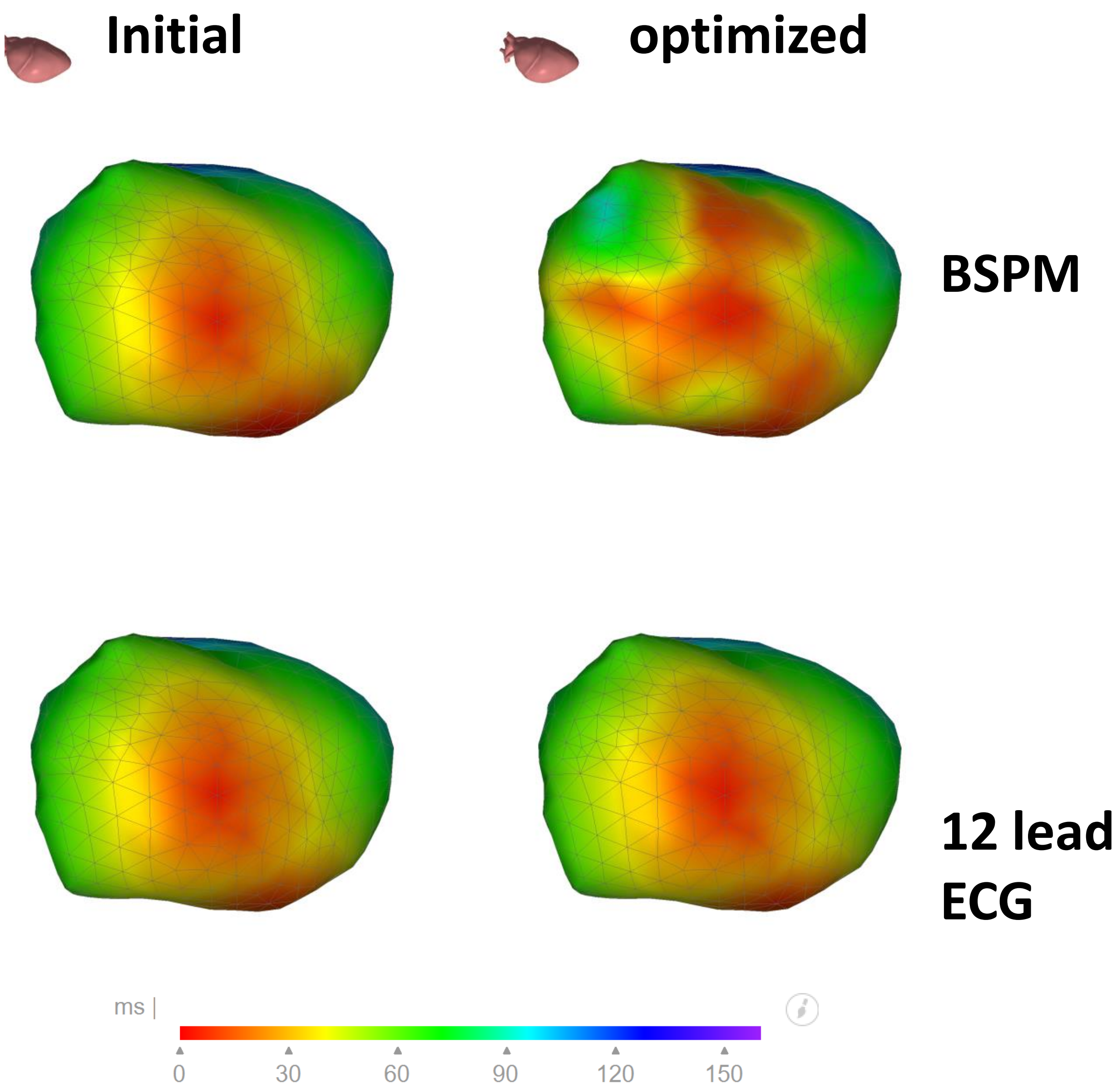
Initial
optimized
BSPM
12 lead
ECG
ms
0
30
60
90
120
150

# Pat010

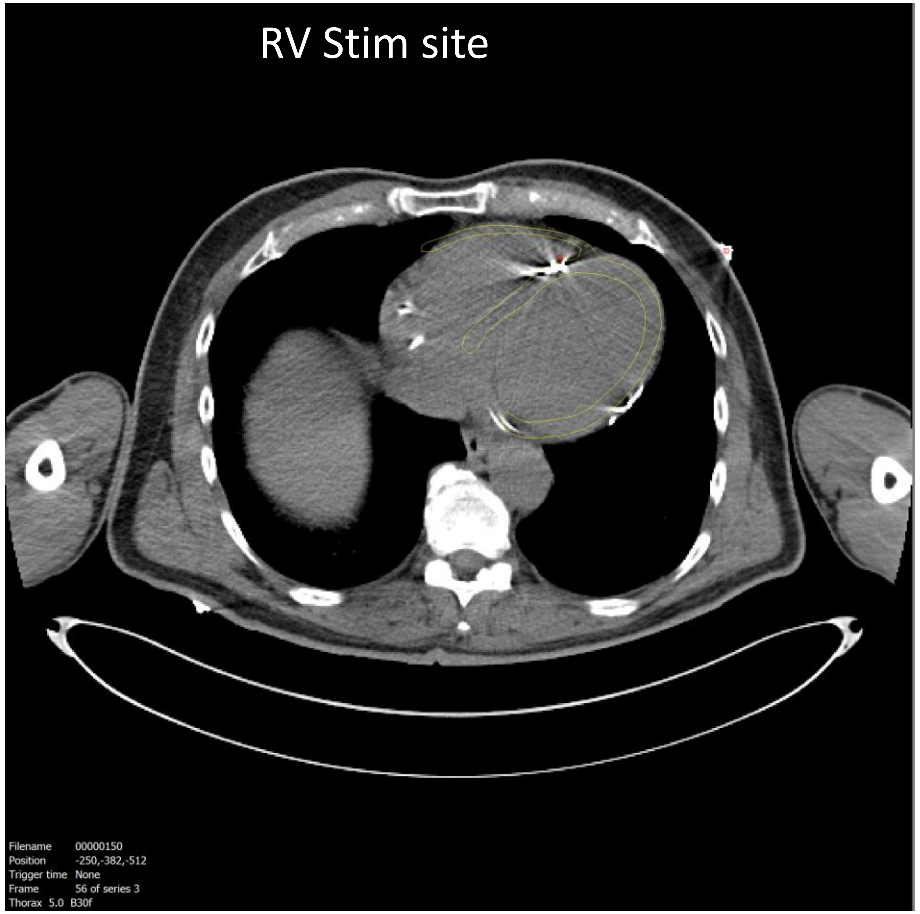
RV Stim site
Filename 00000150
Position -250,-382,-512
Trigger time None
Frame 56 of series 3
Thorax 5.0 B30f

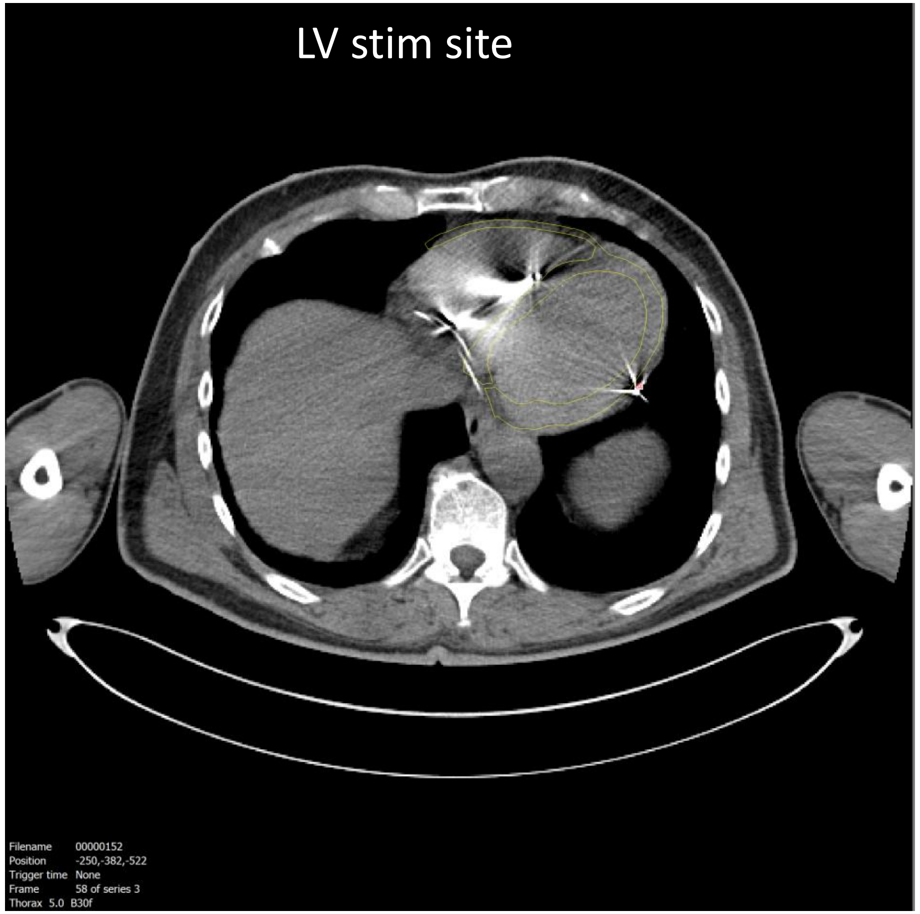
LV stim site
Filename 00000152
Position -250,-382,-522
Trigger time None
Frame 58 of series 3
Thorax 5.0 B30f

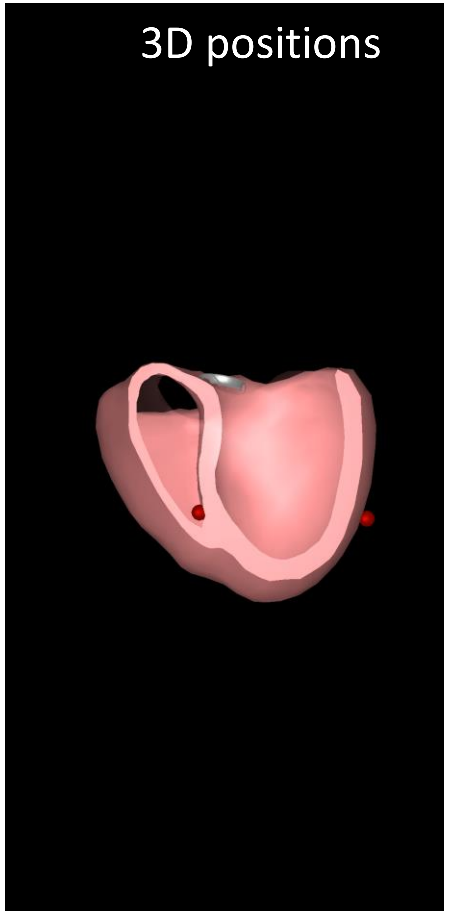
3D positions

# LV STIM localization

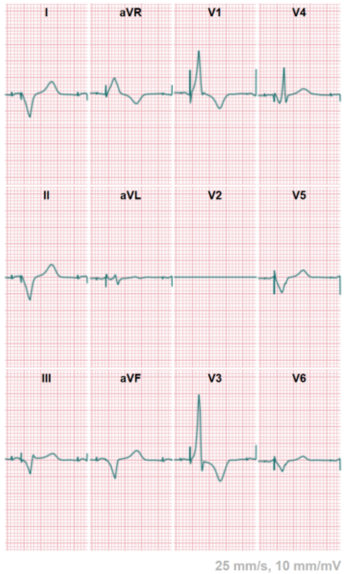


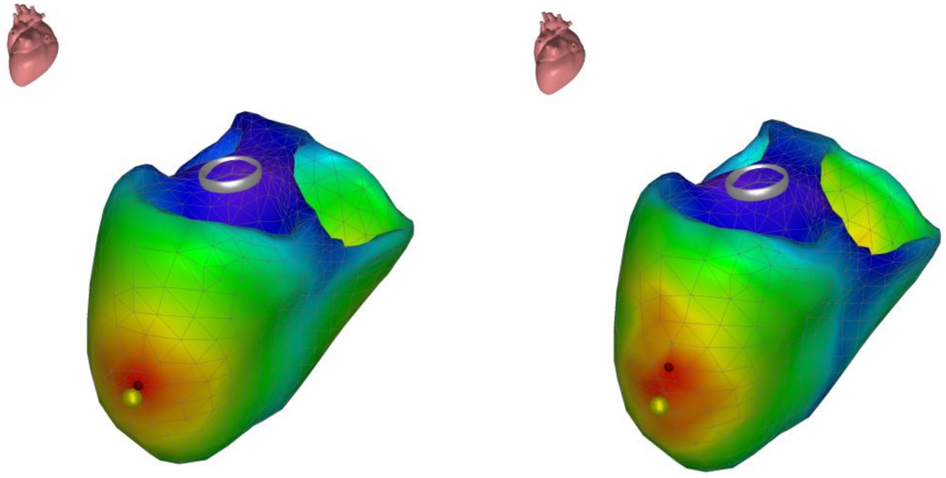

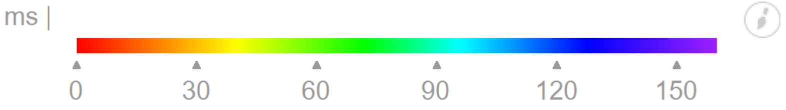


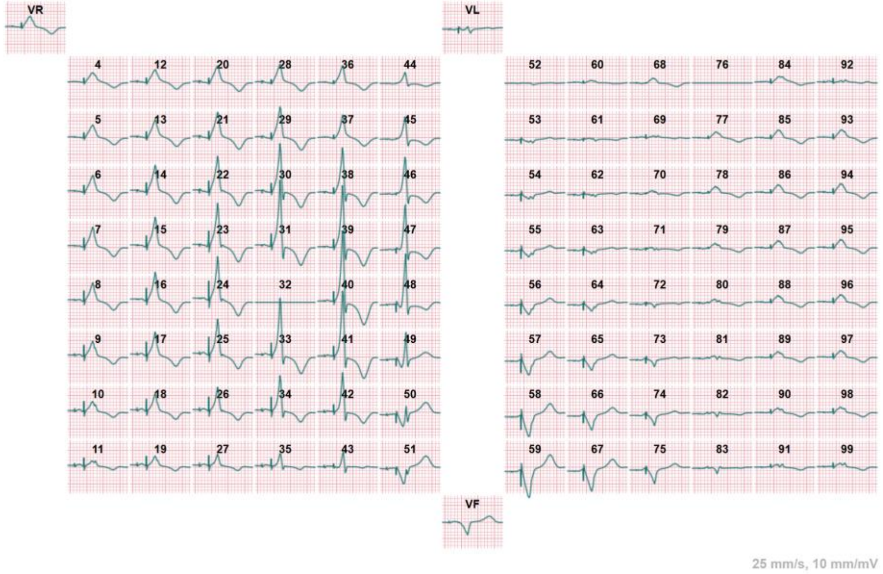


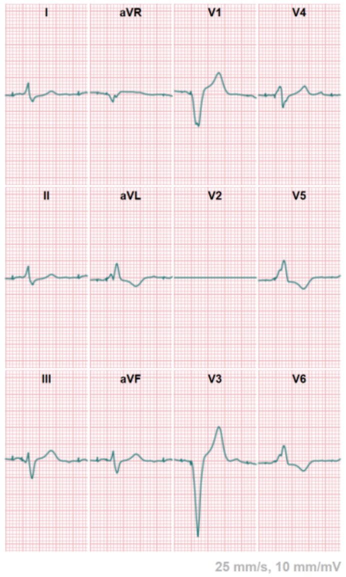


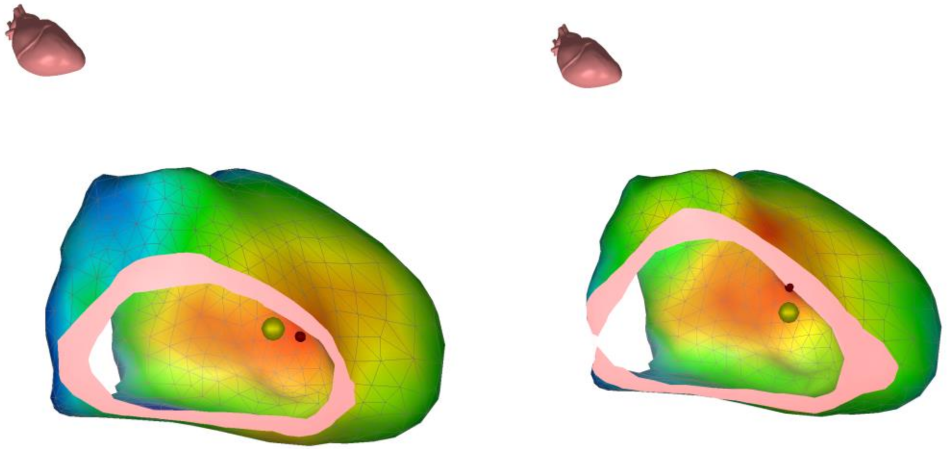

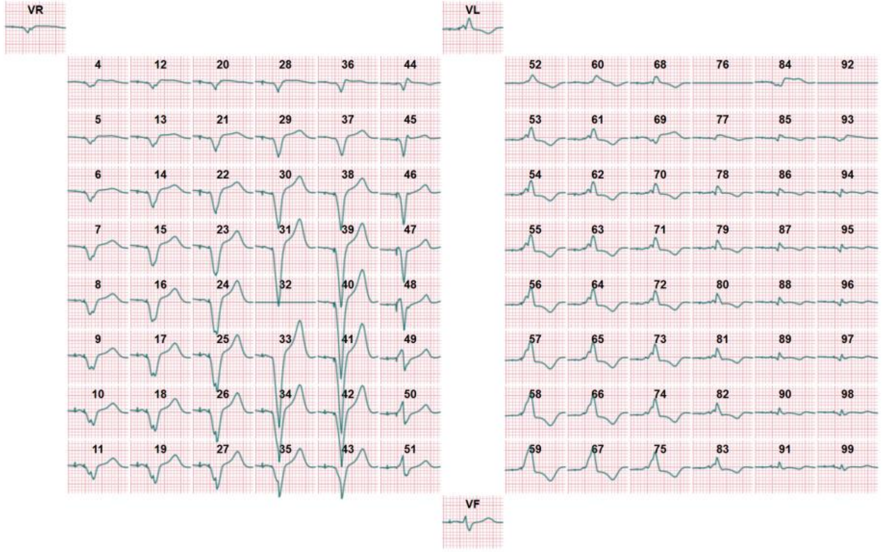

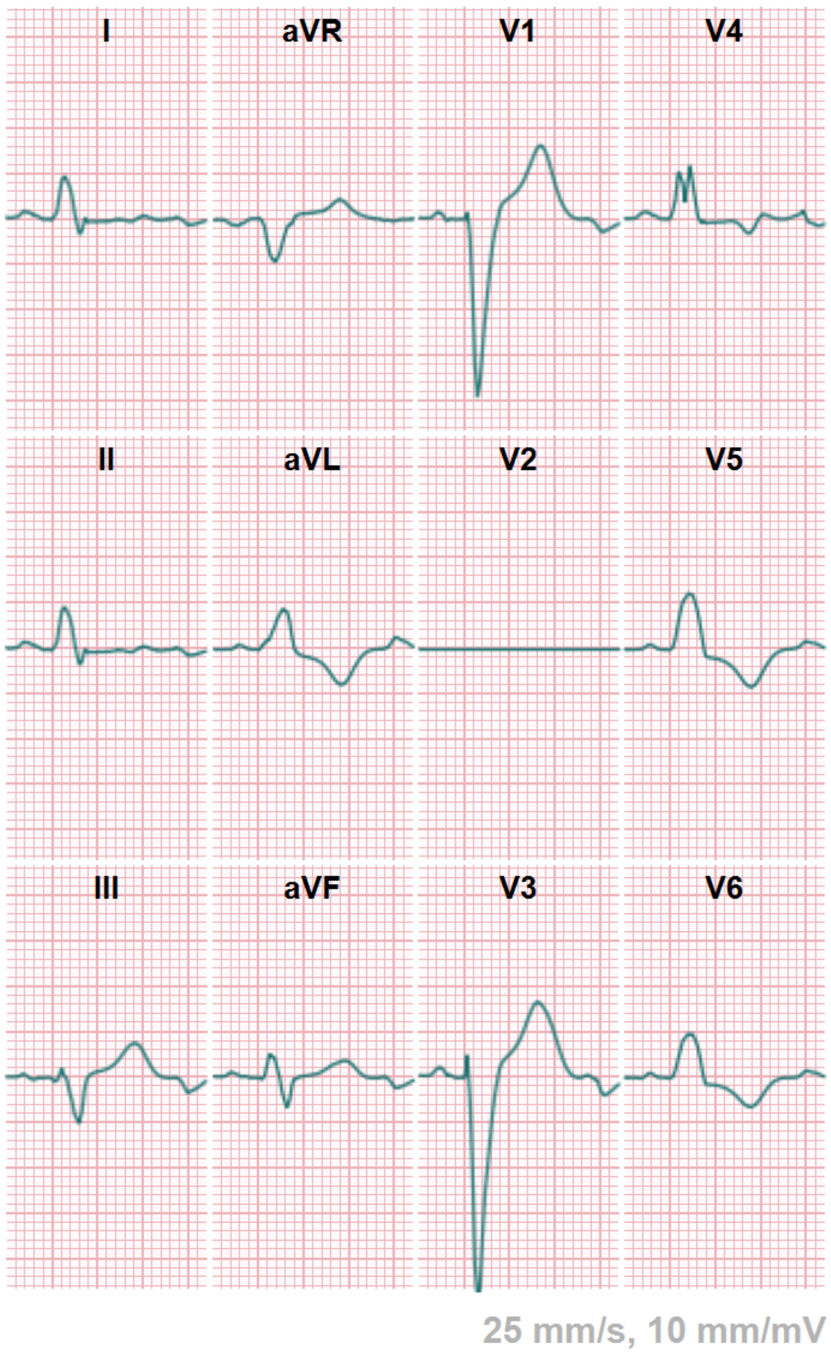
I
aVR
V1
V4
II
aVL
V2
V5
III
aVF
V3
V6
25 mm/s, 10 mm/mV

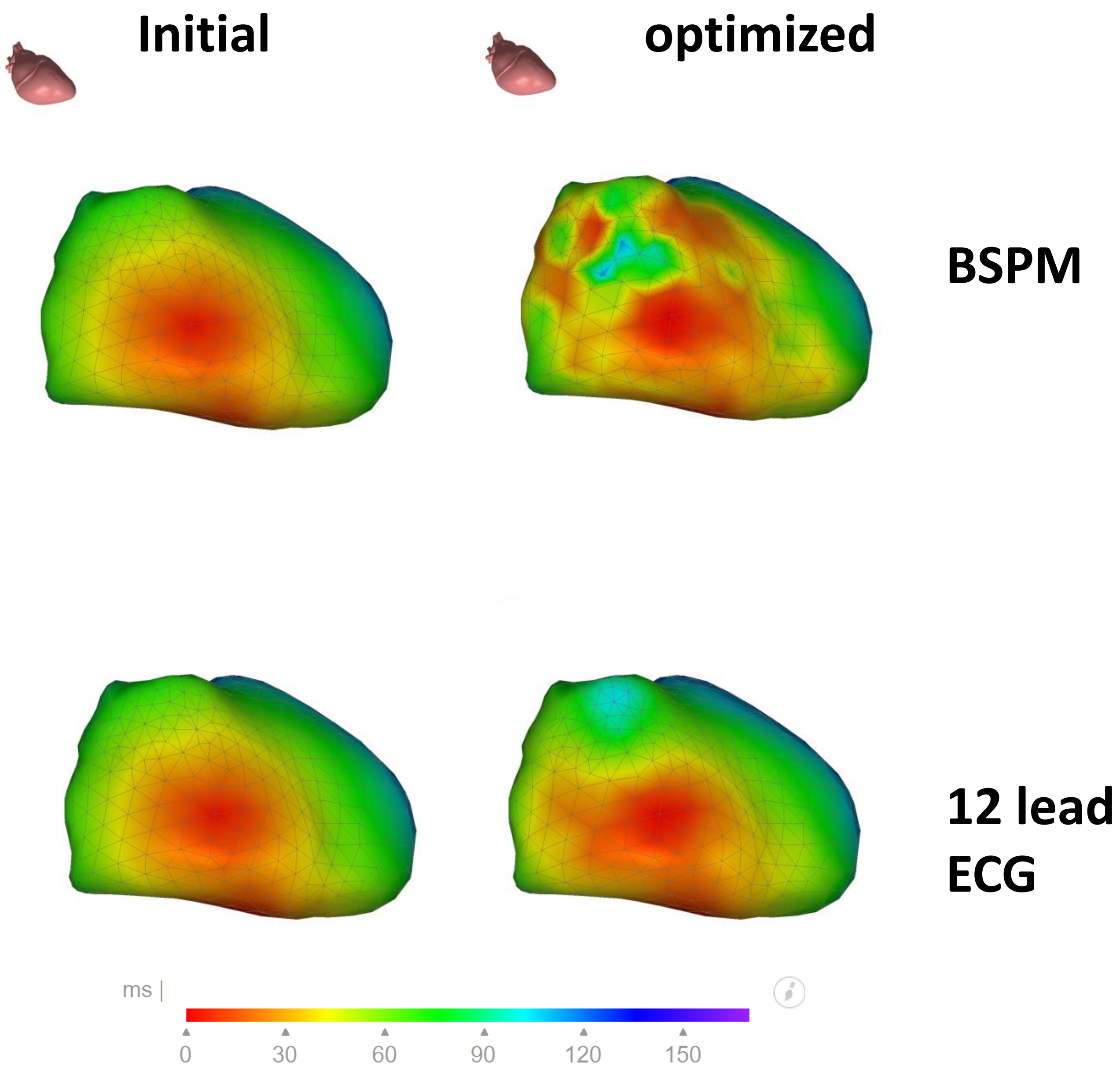
Initial
optimized
BSPM
12 lead
ECG
ms
0
30
60
90
120
150

# Pat011

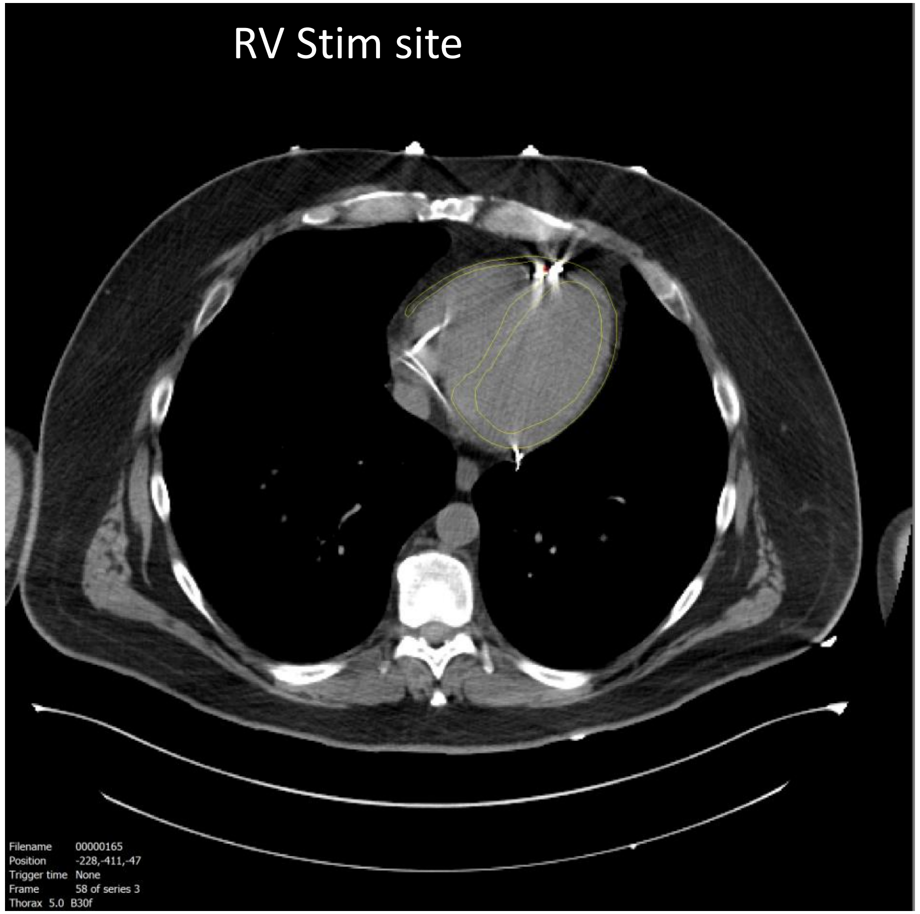
RV Stim site
Filename 00000165
Position -228,-411,-47
Trigger time None
Frame 58 of series 3
Thorax 5.0 B30f

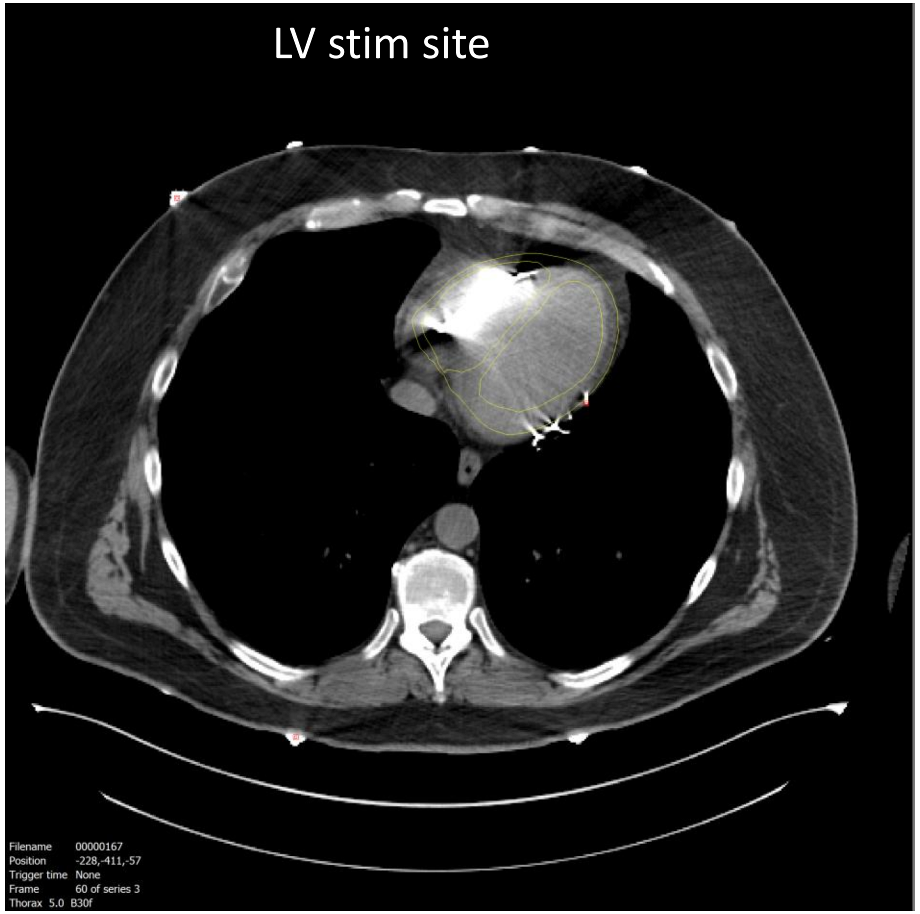
LV stim site
Filename 00000167
Position -228,-411,-57
Trigger time None
Frame 60 of series 3
Thorax 5.0 B30f

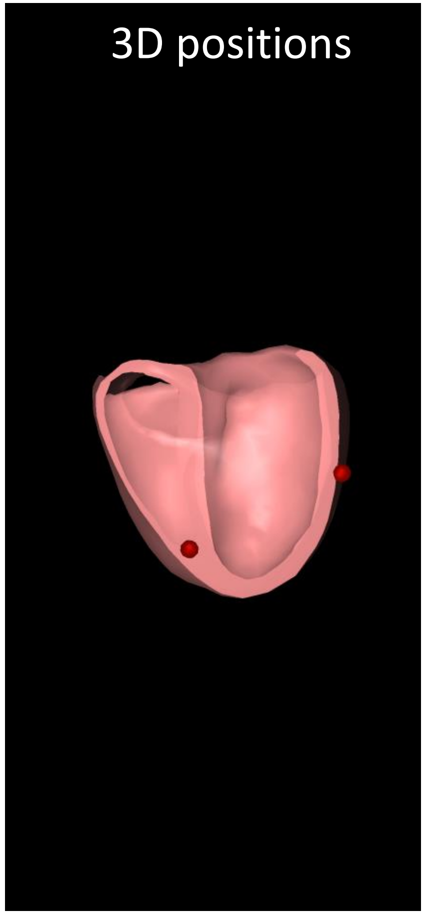
3D positions

# LV STIM localization

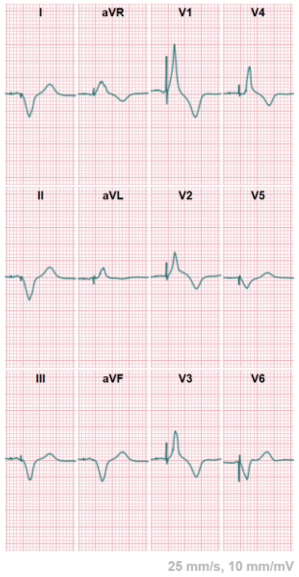


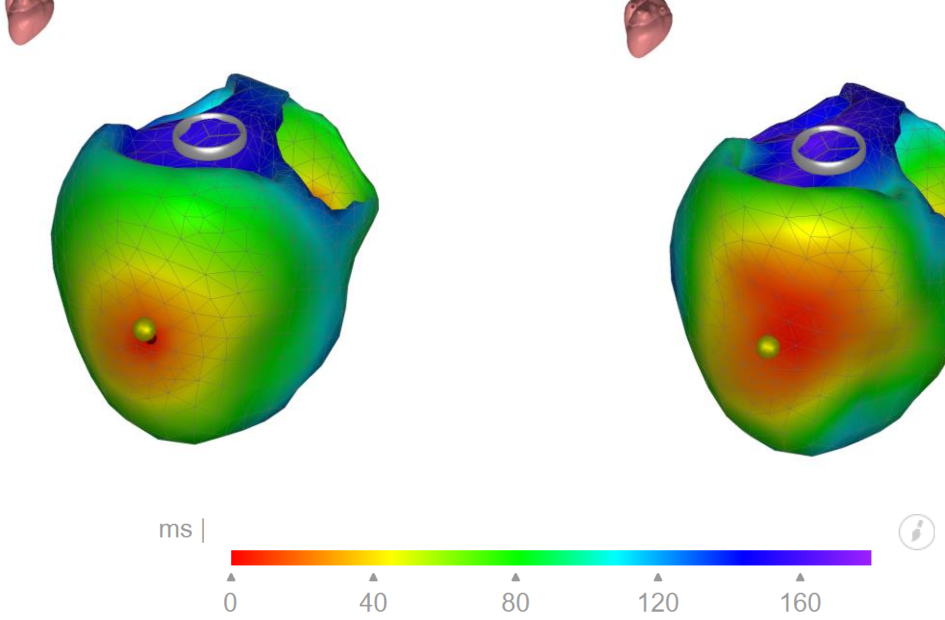


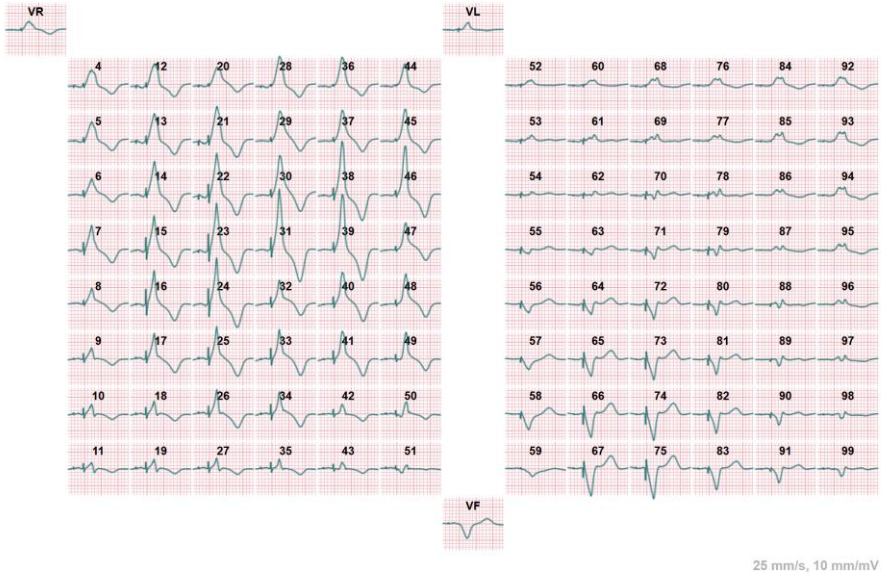


# RV STIM localization

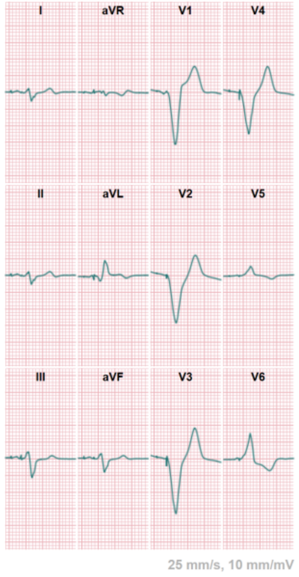


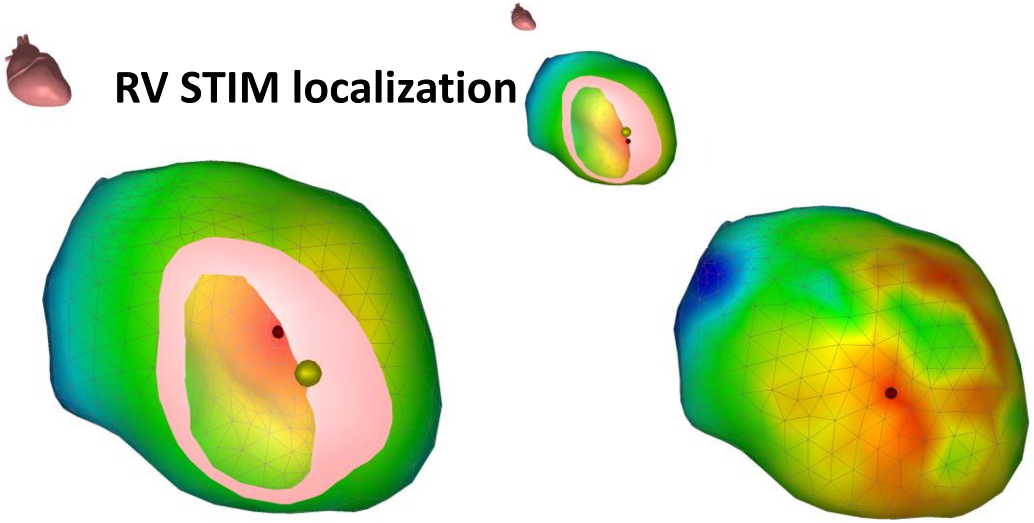

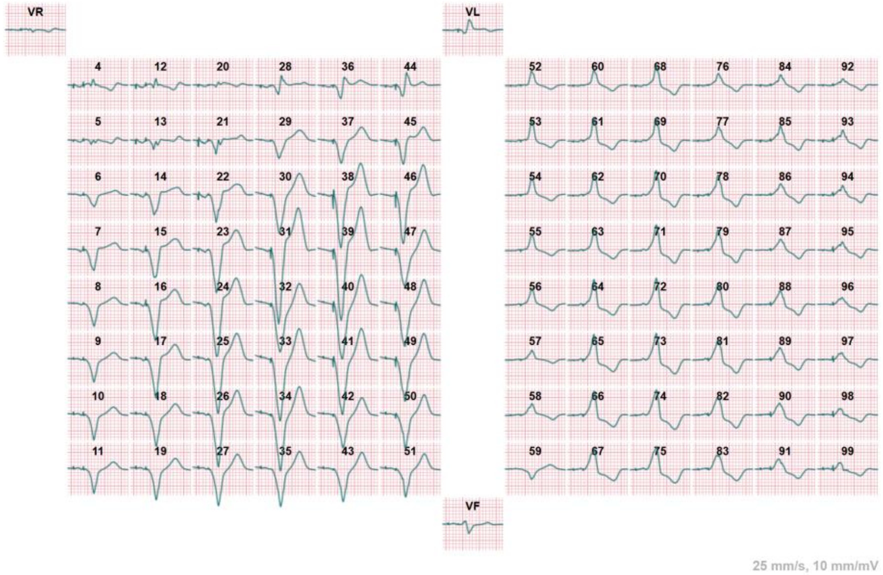

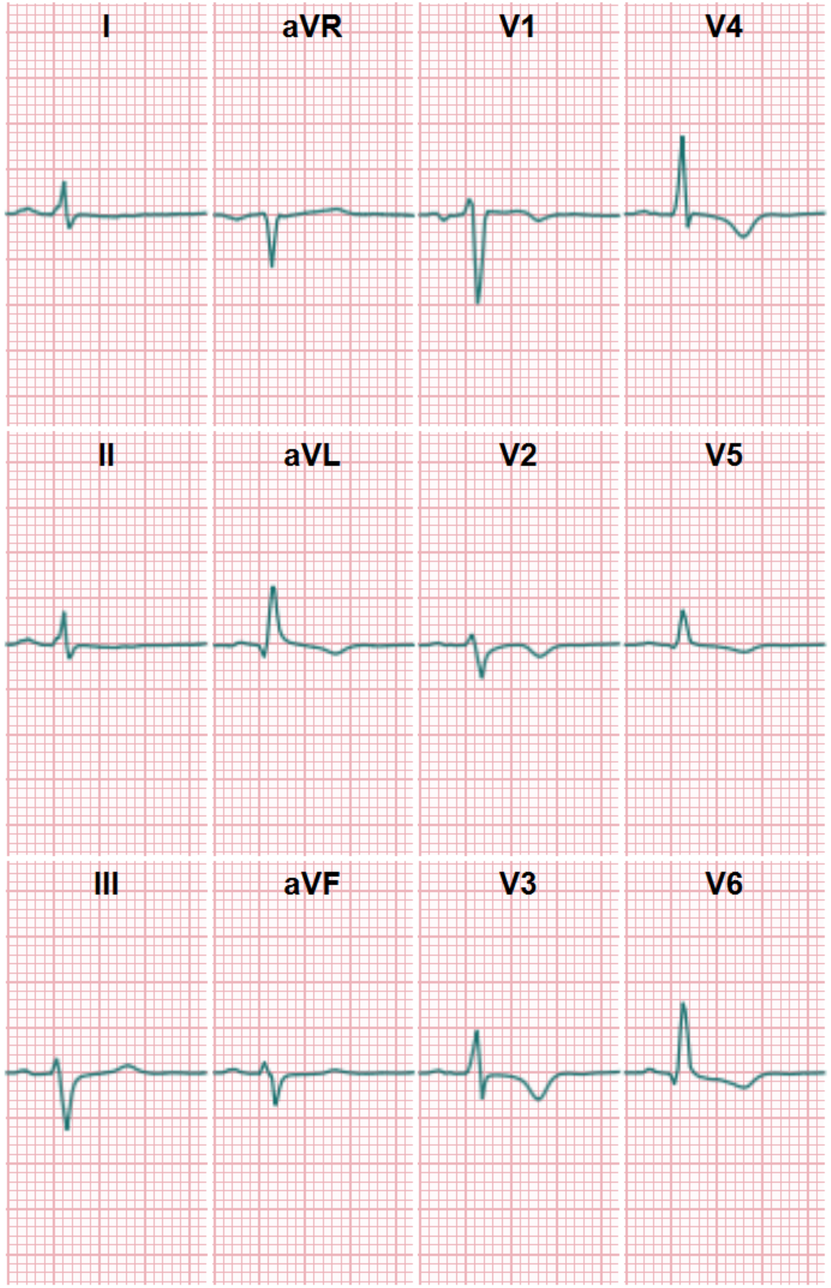


25 mm/s, 10 mm/mV

Initial

optimized

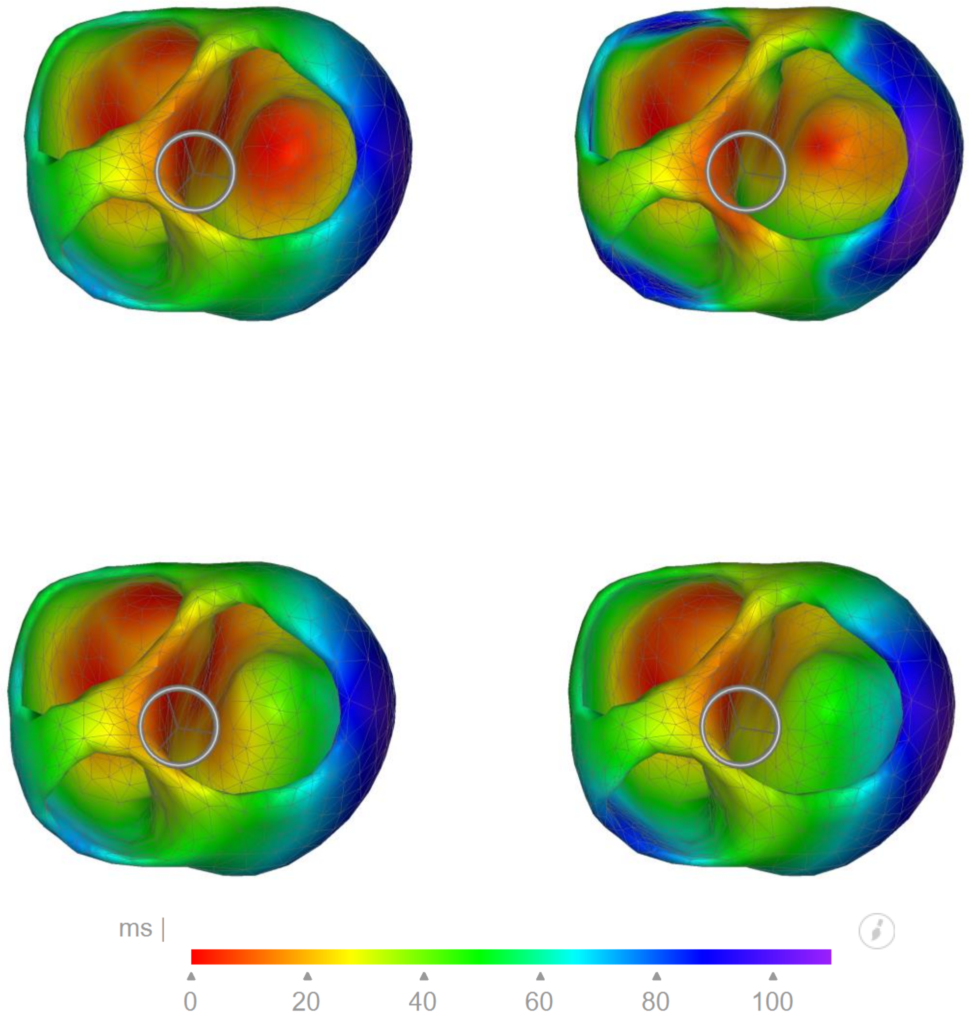


BSPM

12 lead ECG

# Pat012

RV Stim site

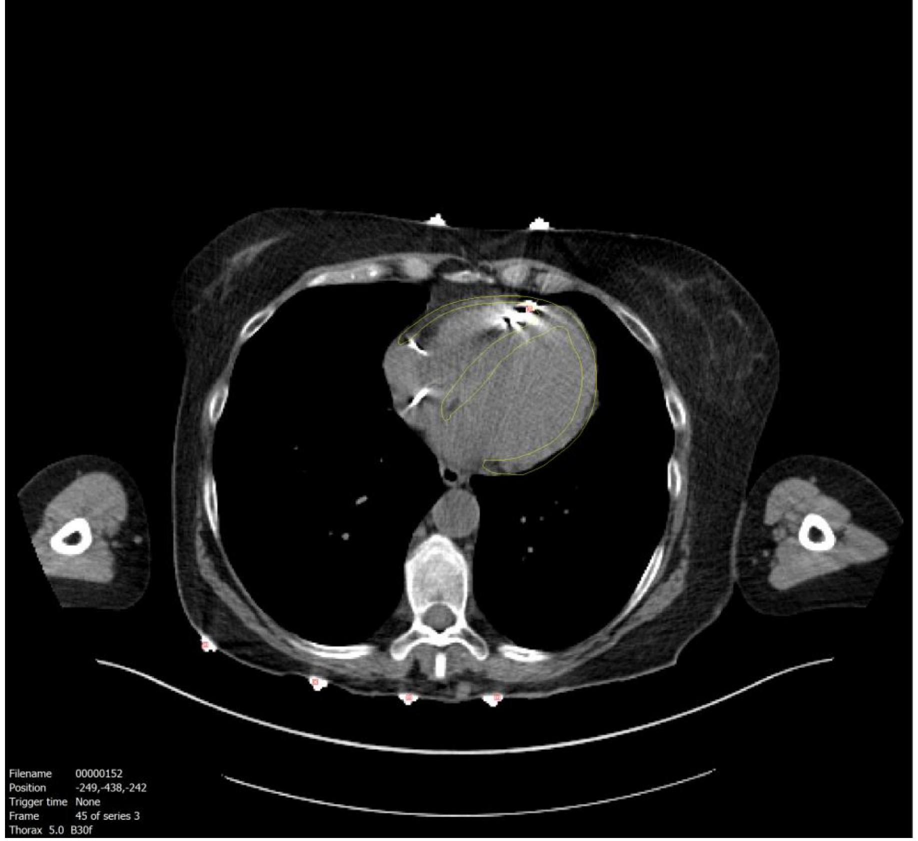


LV stim site

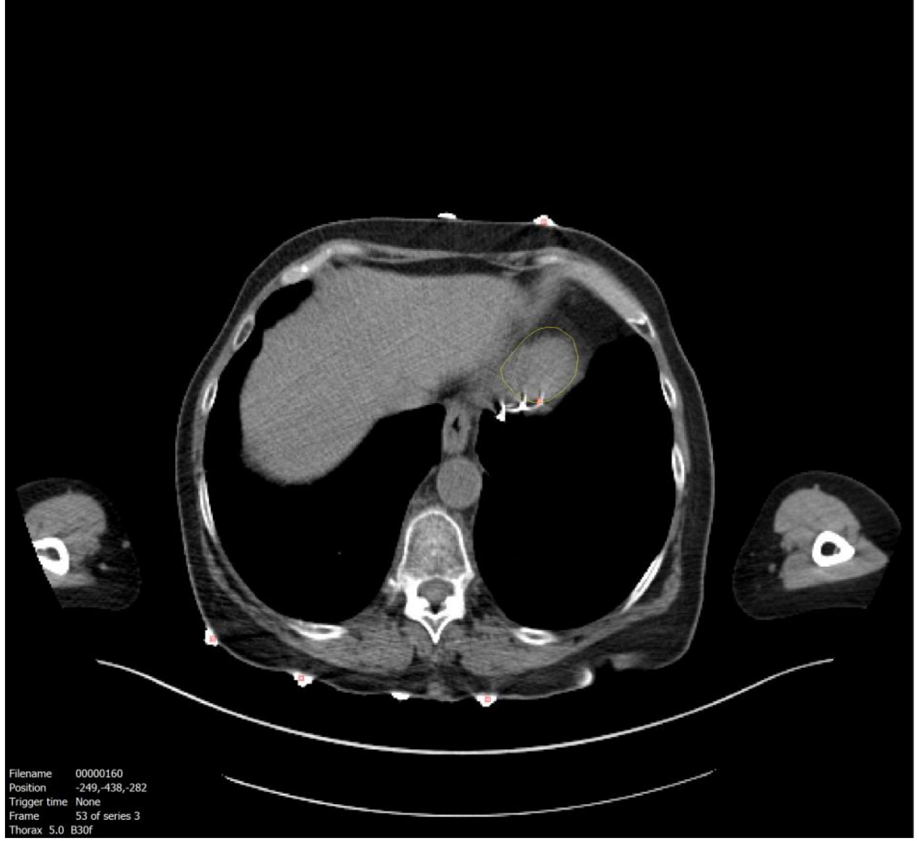


3D positions

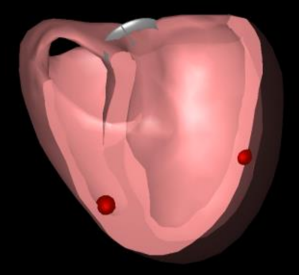

# LV STIM localization

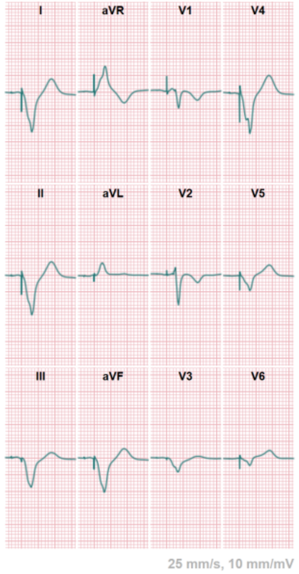


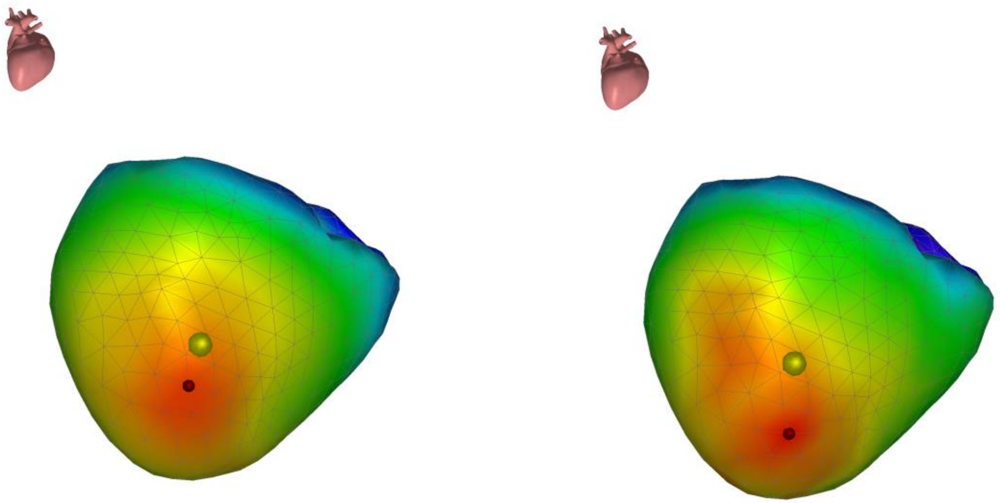

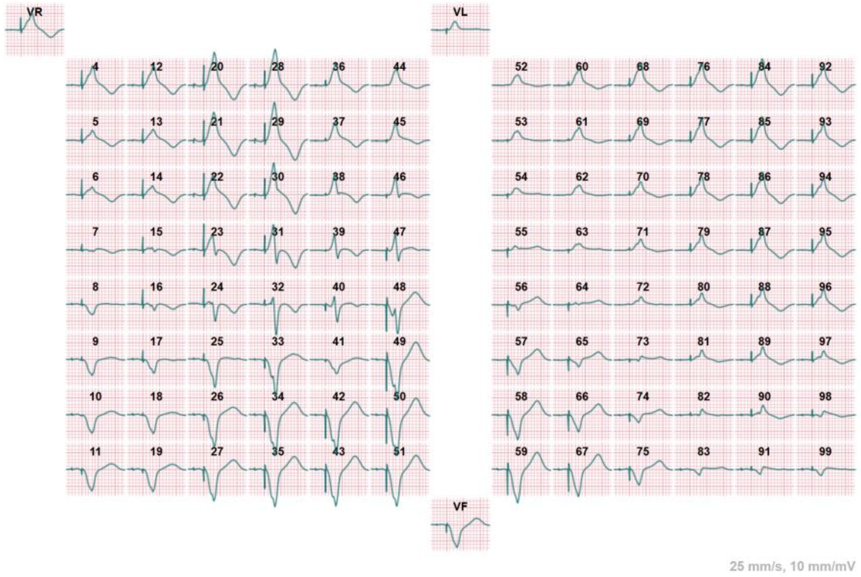


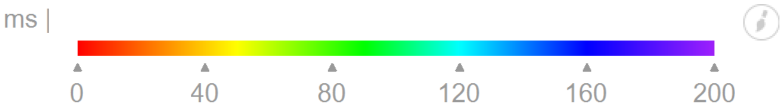


# RV STIM localization

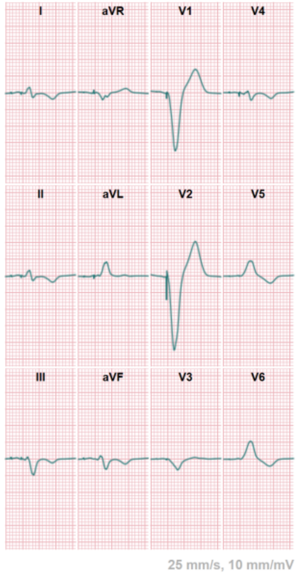


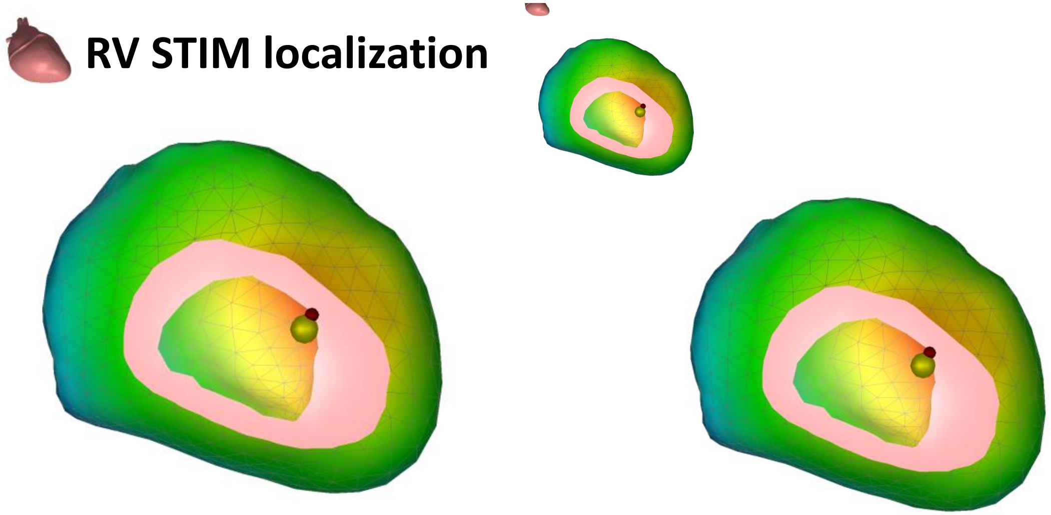

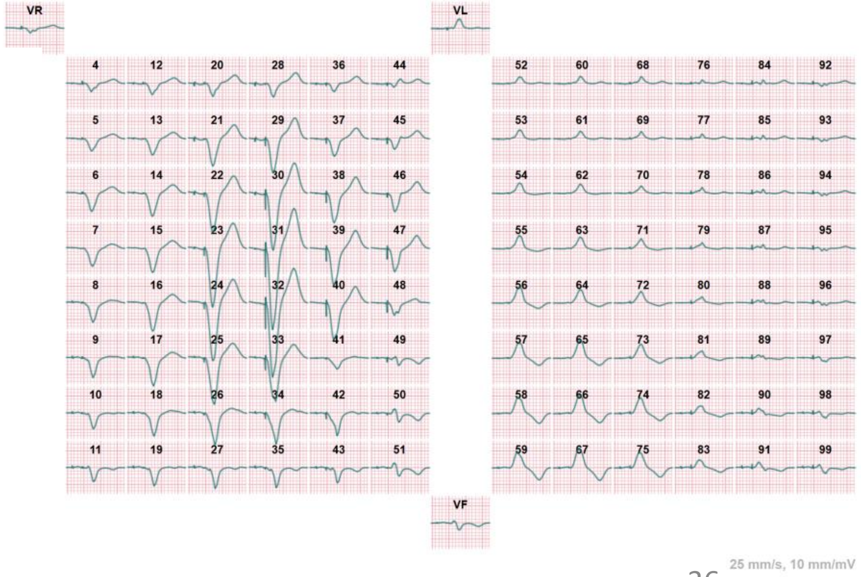

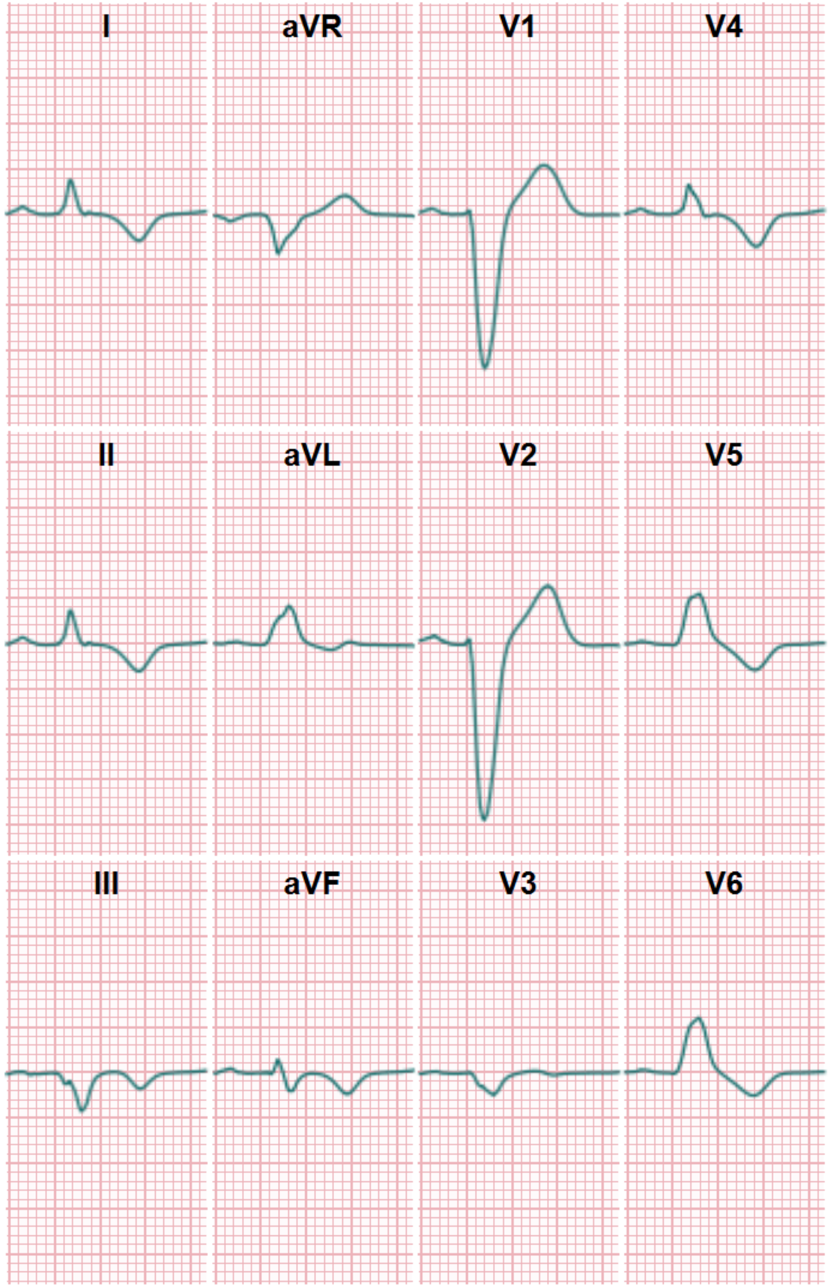


25 mm/s, 10 mm/mV

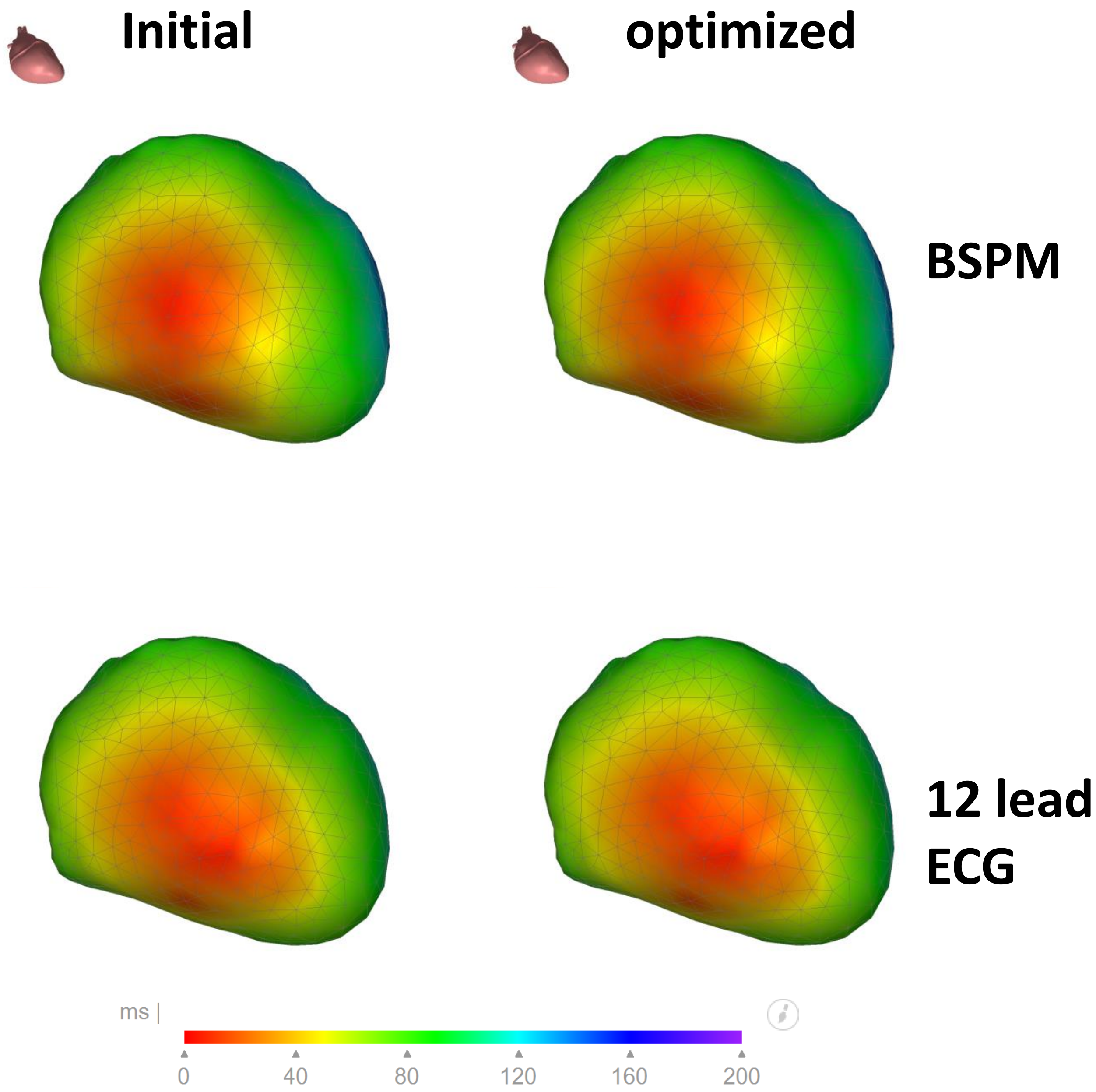

# Pat013

RV Stim site

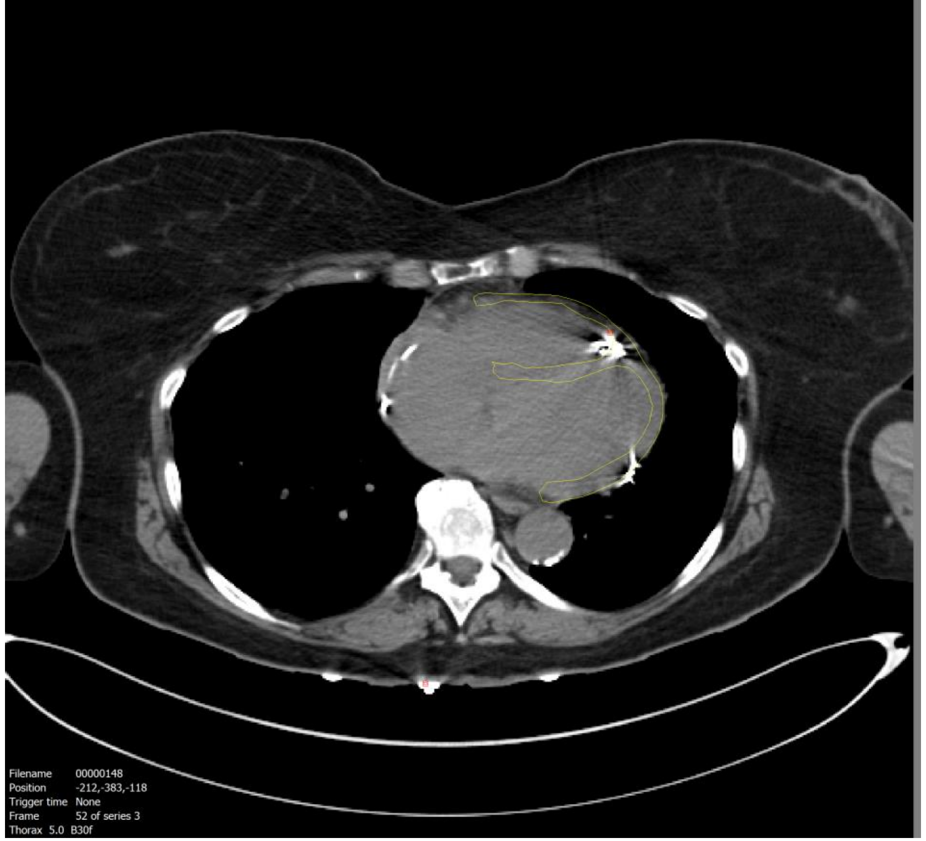


LV stim site

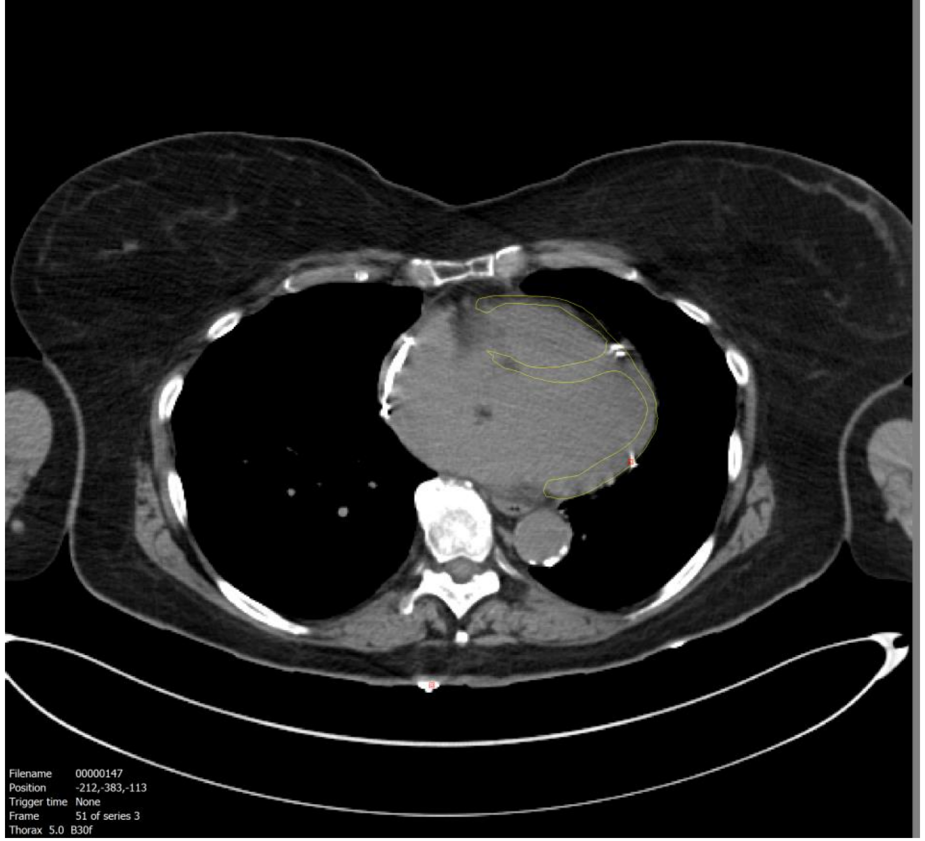


3D positions

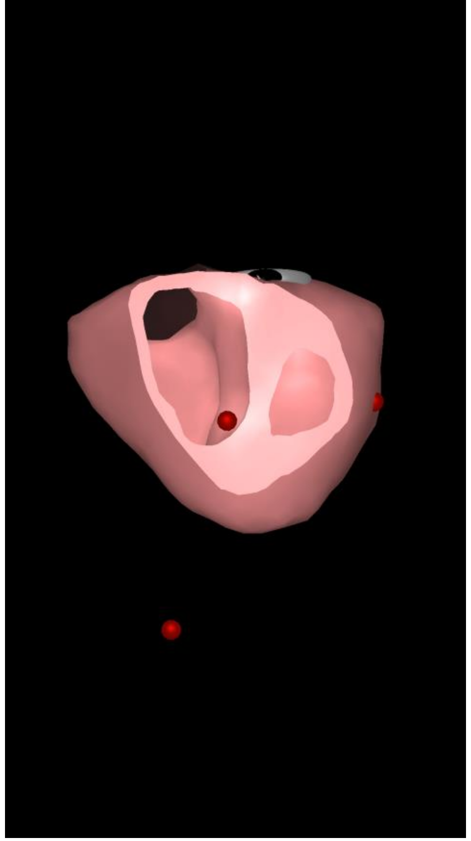

## LV STIM localization

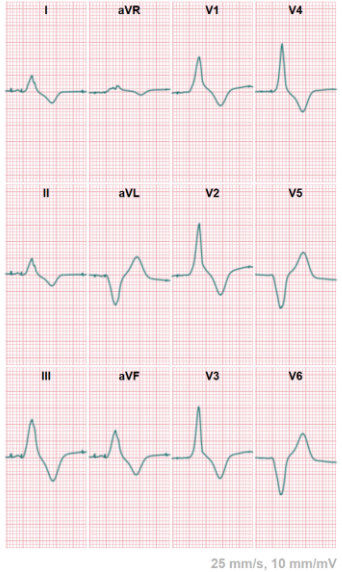


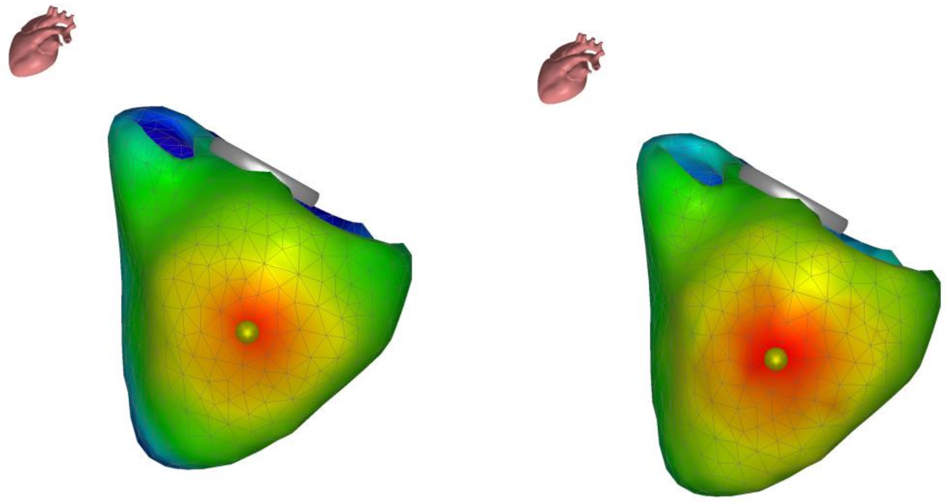

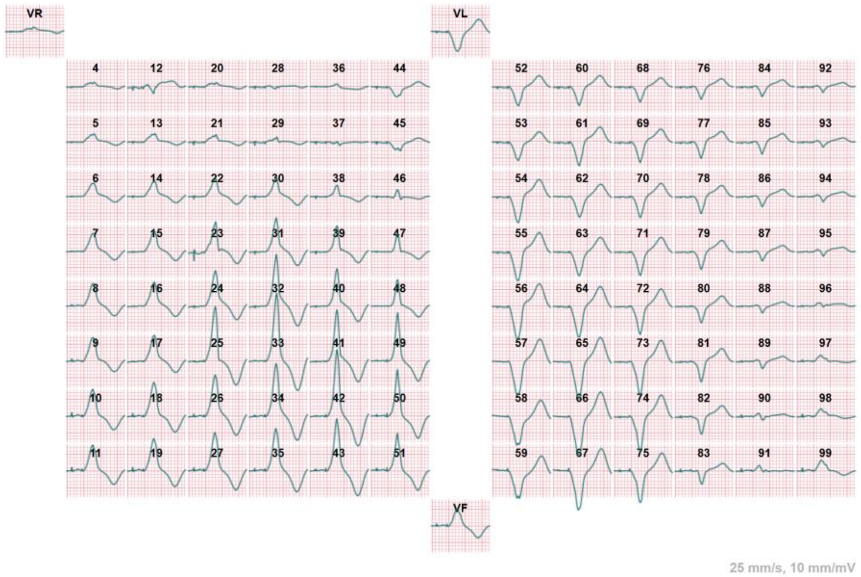


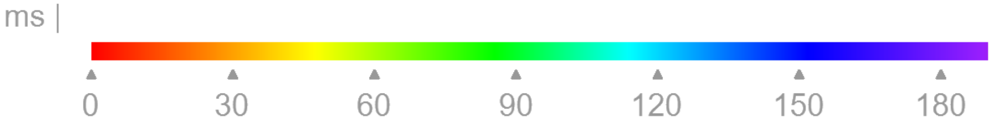


## RV STIM localization

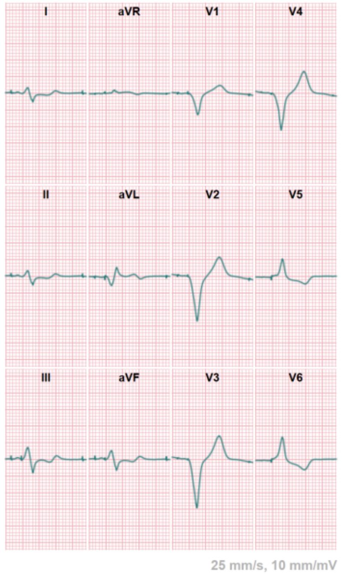


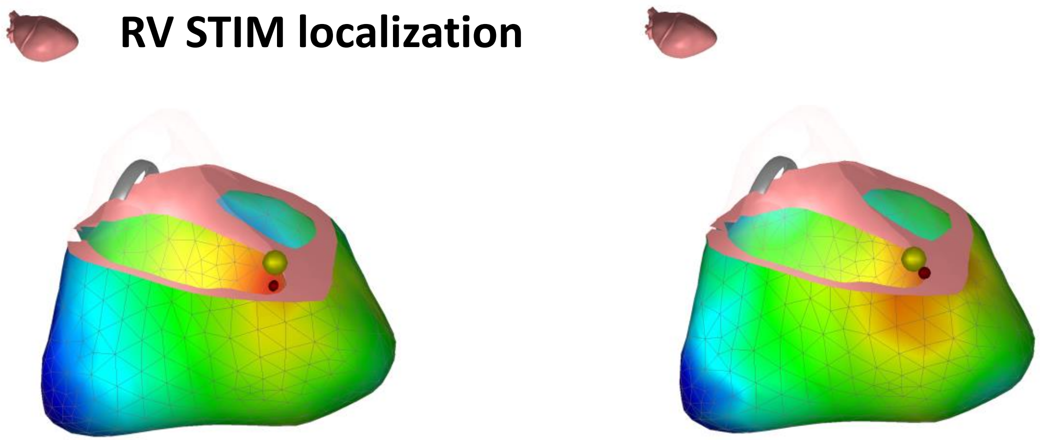

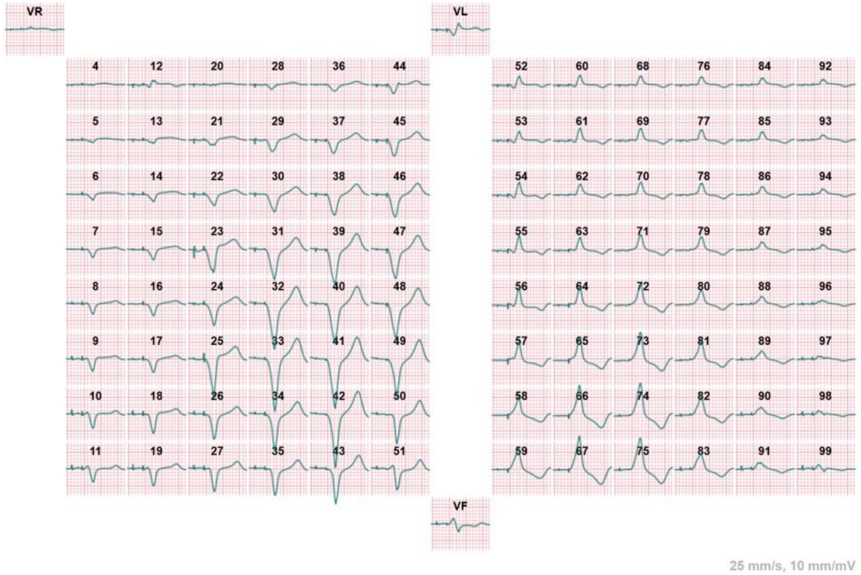

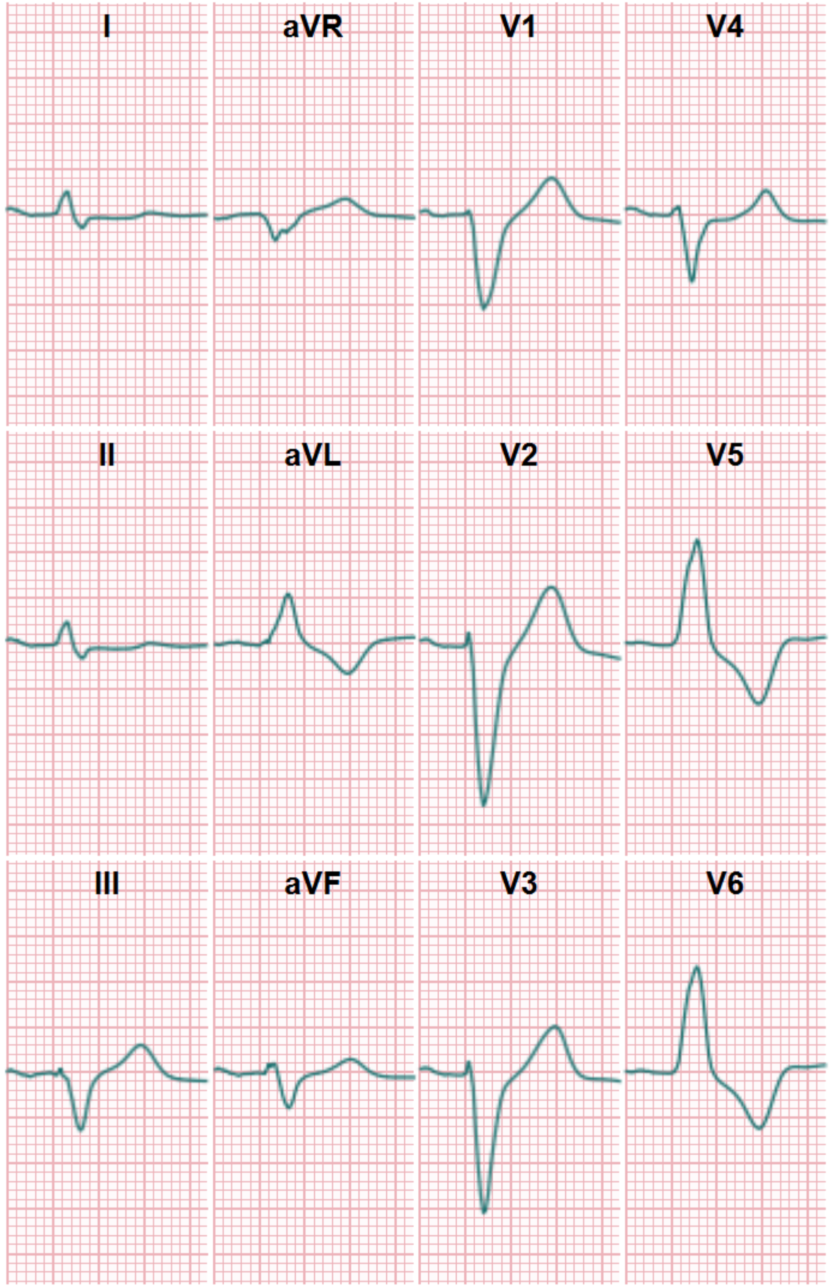


25 mm/s, 10 mm/mV

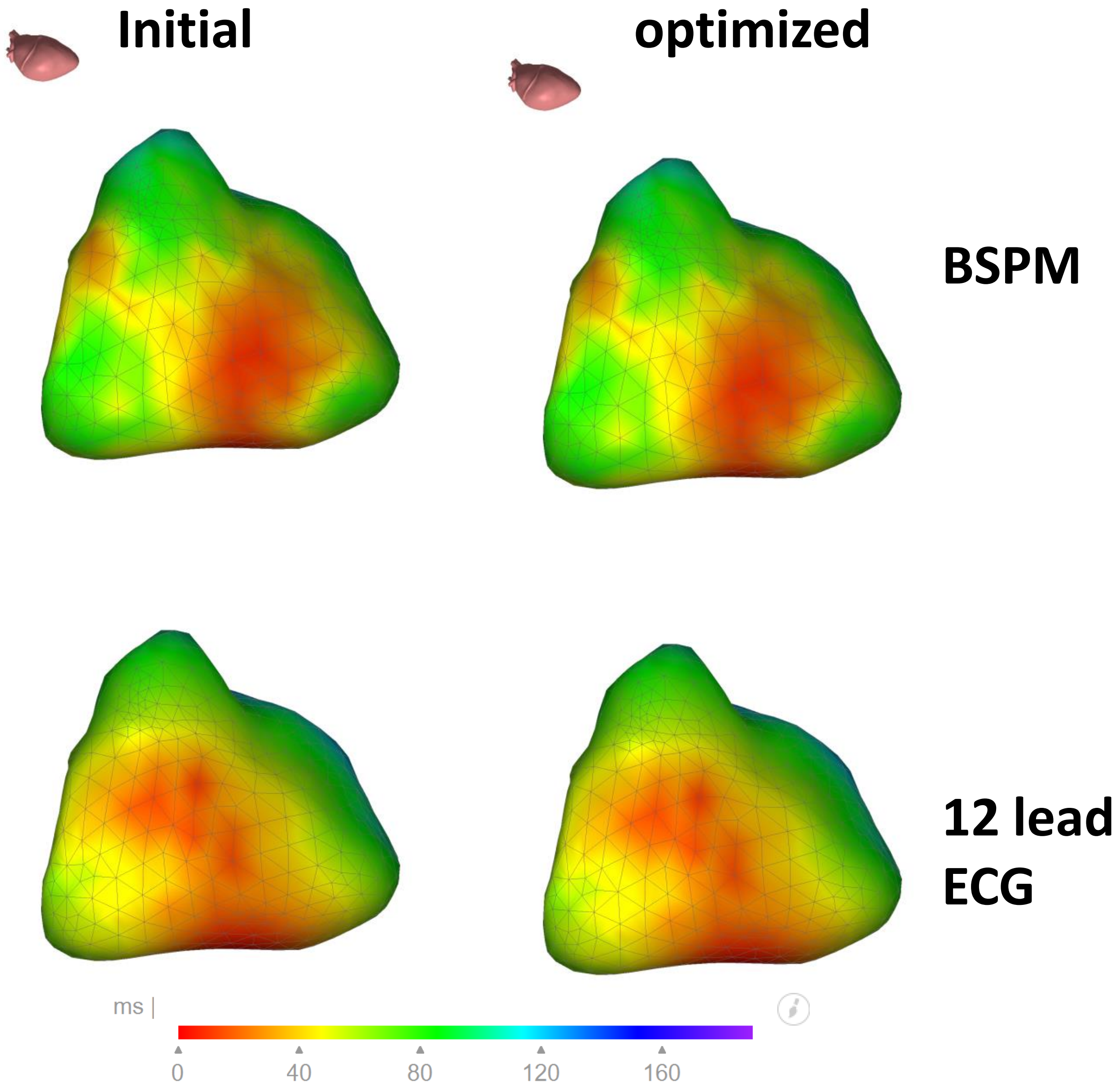

# Pat014

RV Stim site

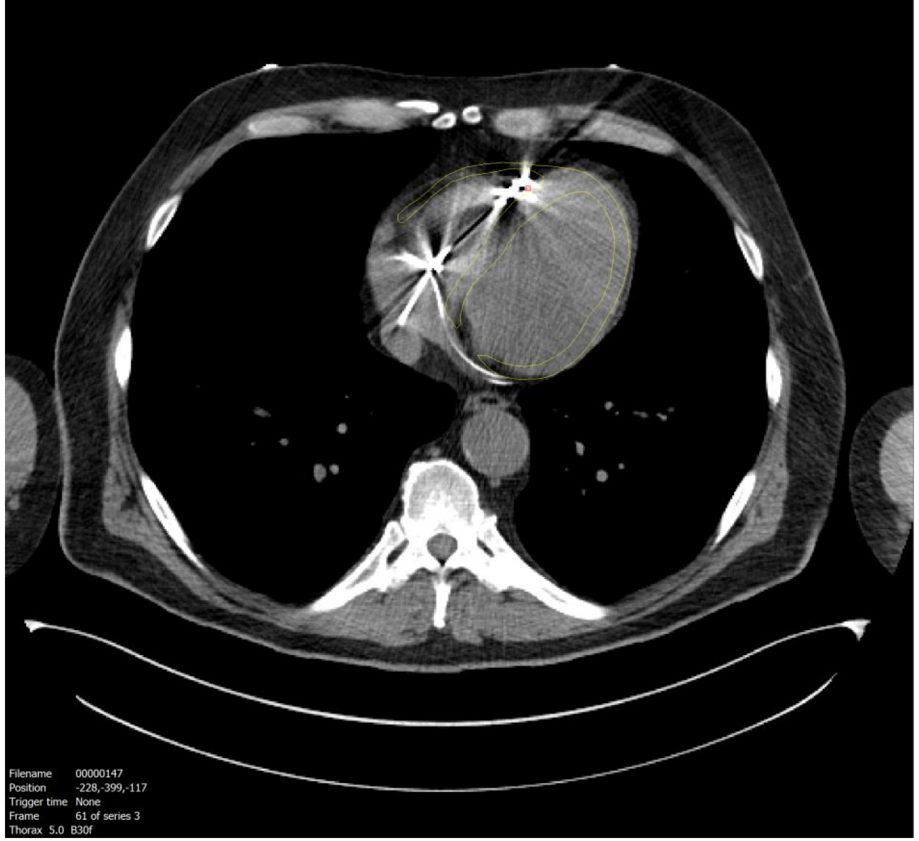


LV stim site

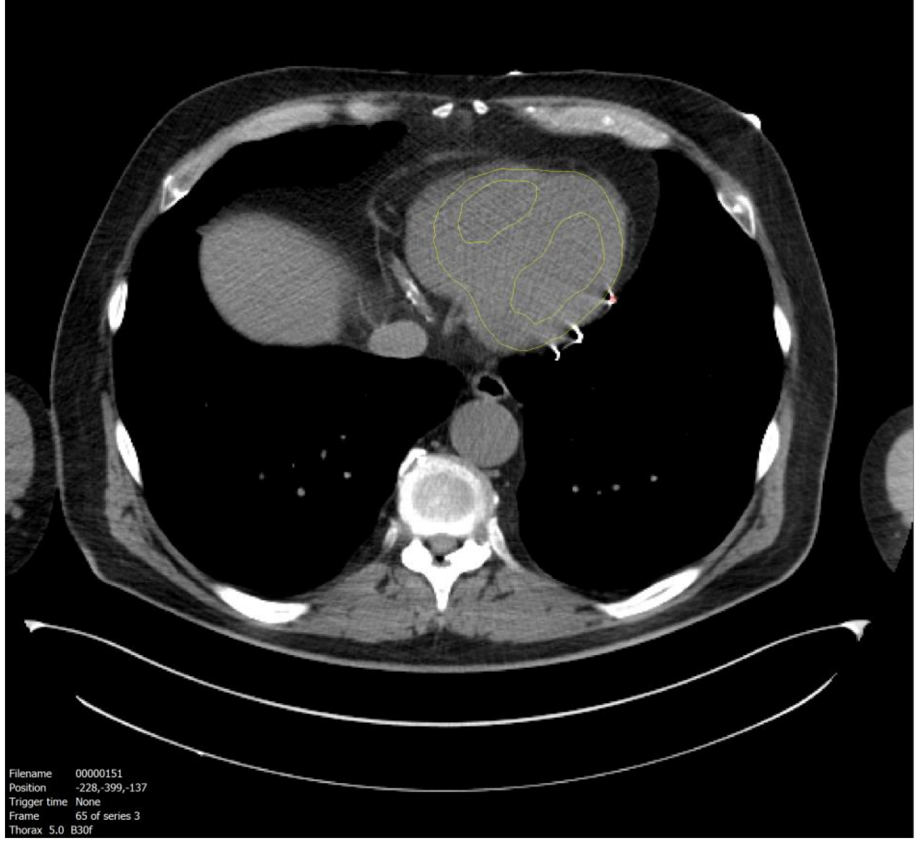


3D positions

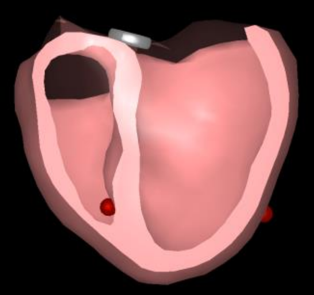

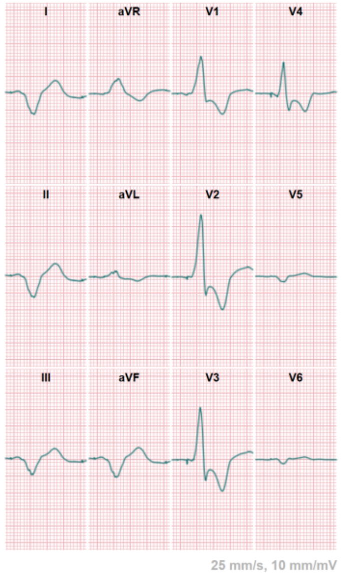


# LV STIM localization

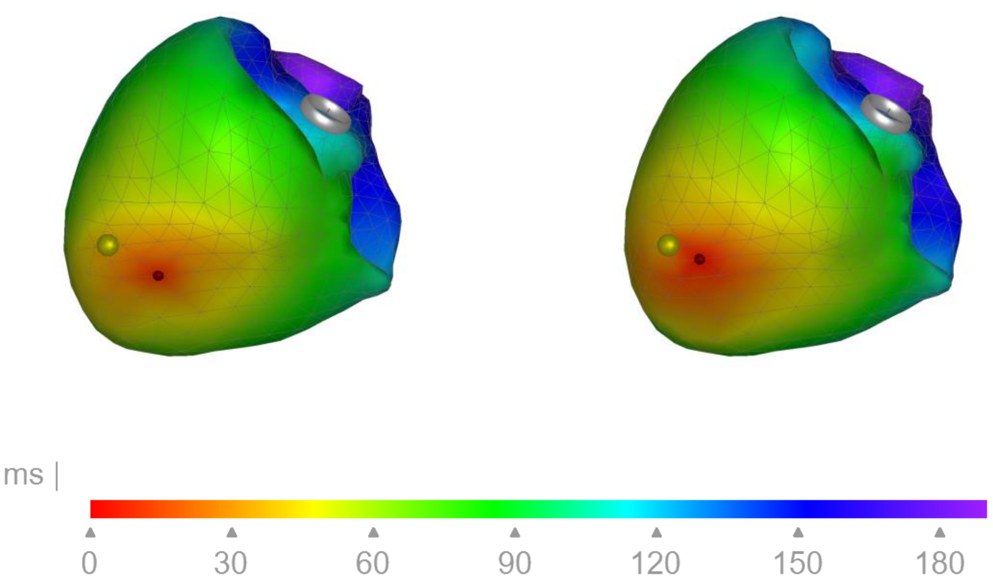


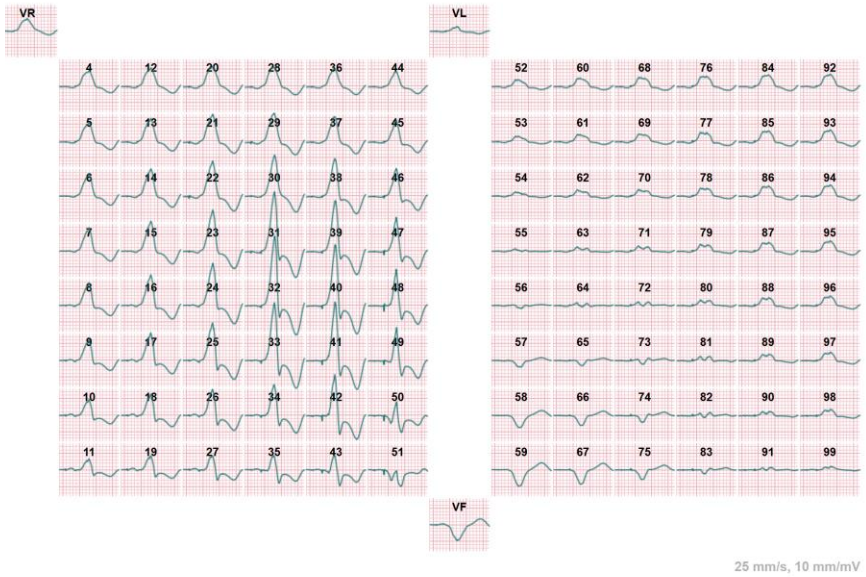


# RV STIM localization

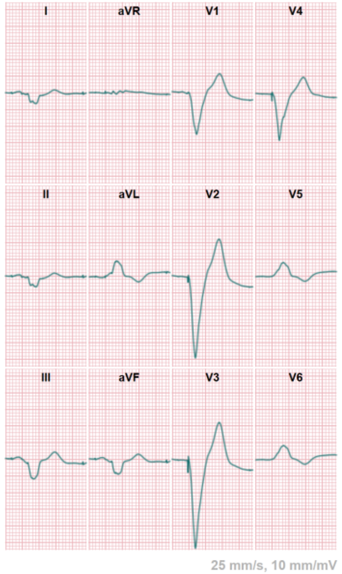


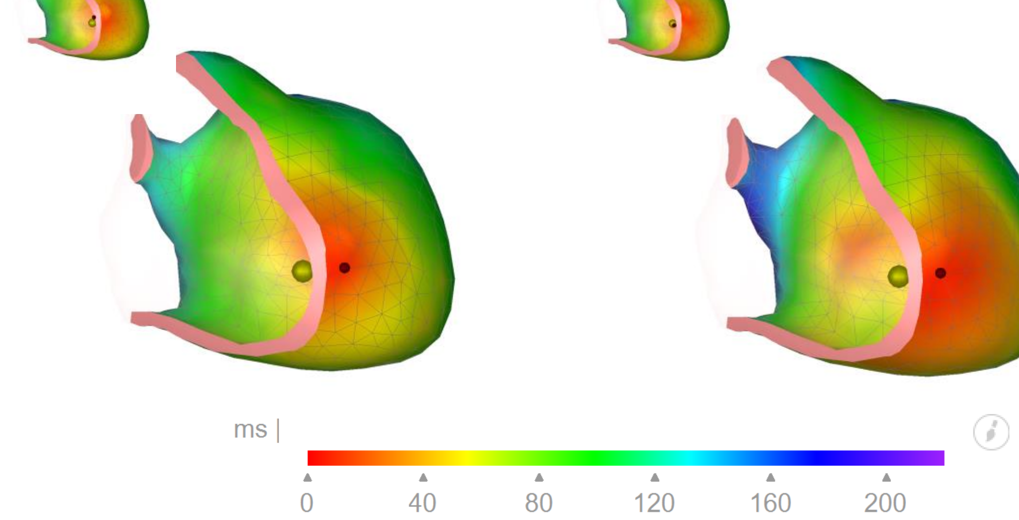


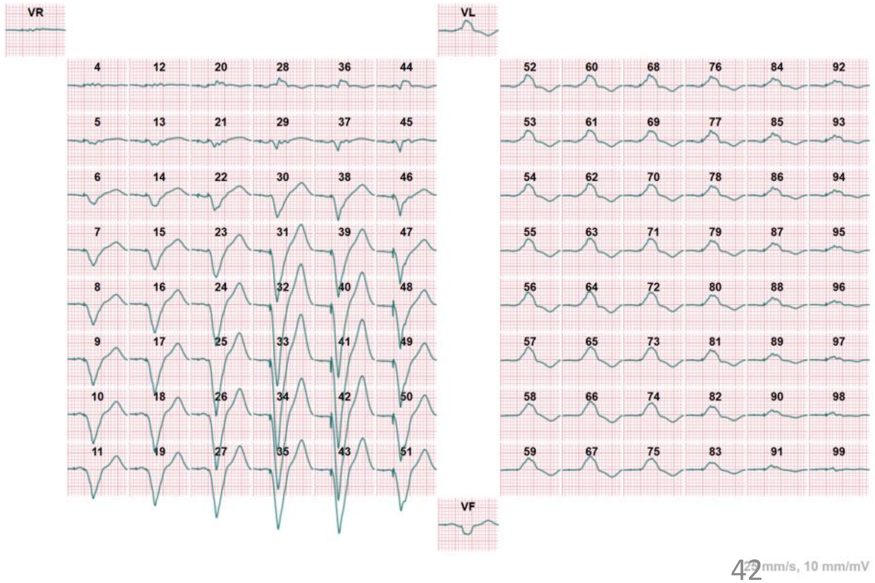

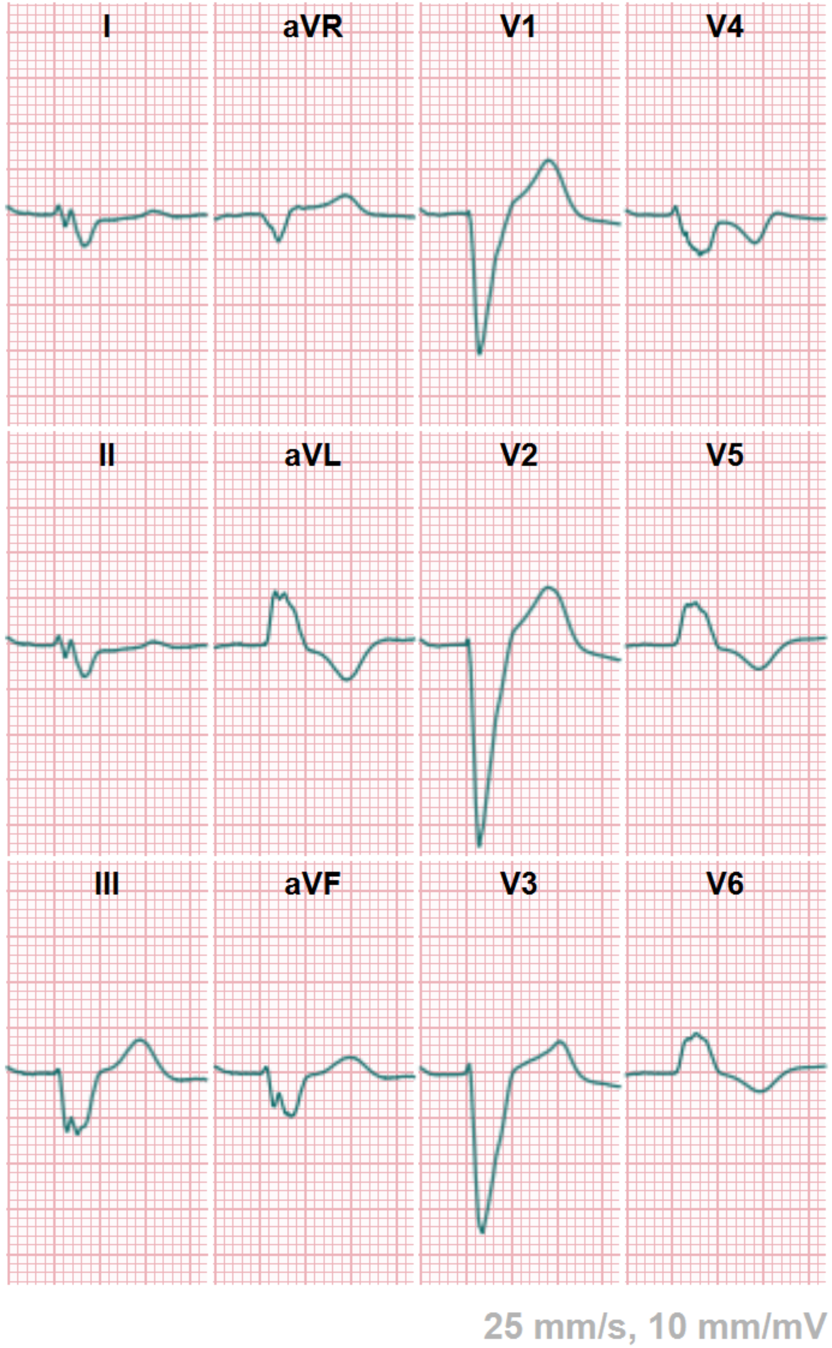
I
aVR
V1
V4
II
aVL
V2
V5
III
aVF
V3
V6
25 mm/s, 10 mm/mV

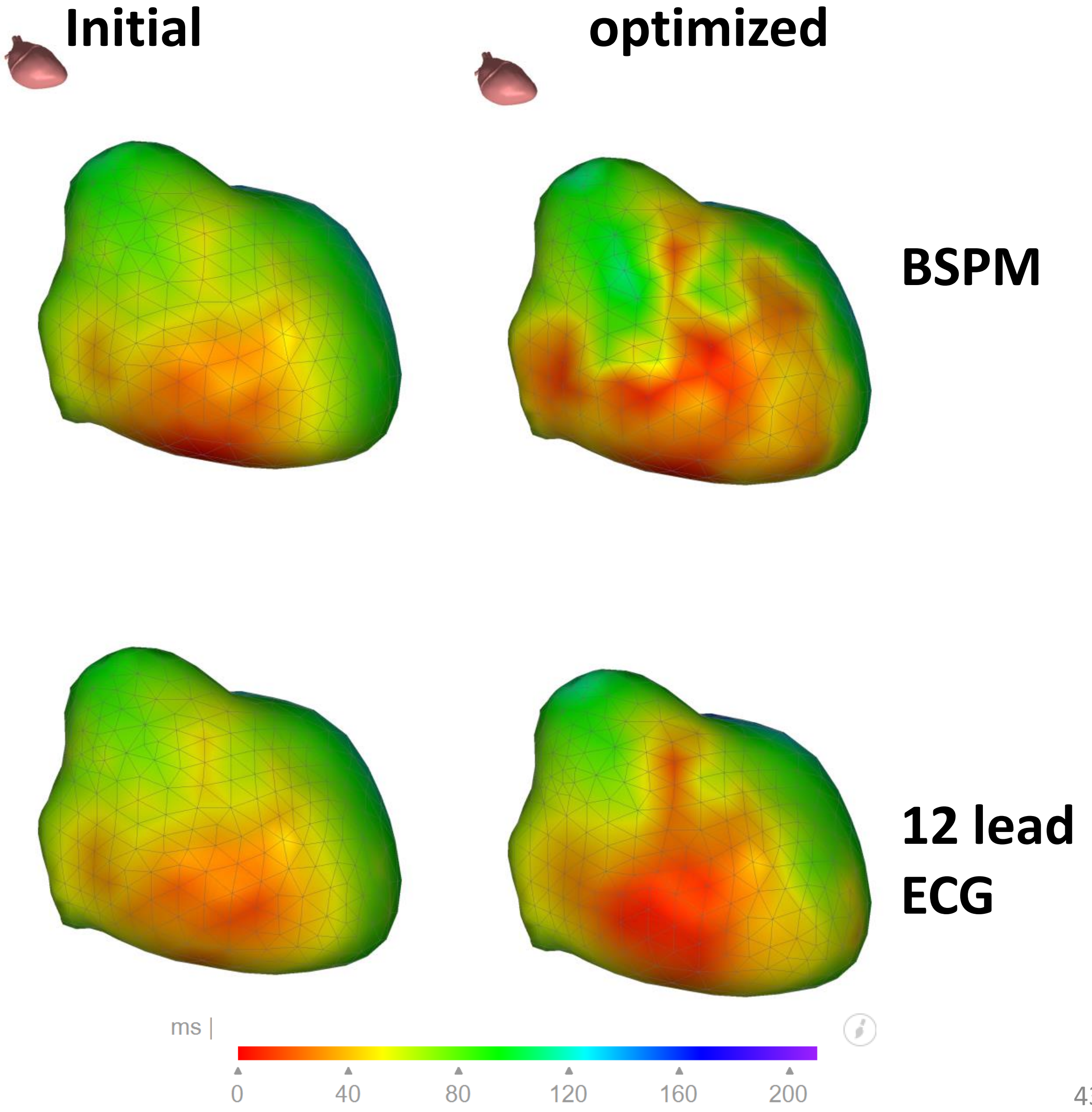
Initial
optimized
BSPM
12 lead
ECG
ms |
0
40
80
120
160
200

# Pat015

RV Stim site

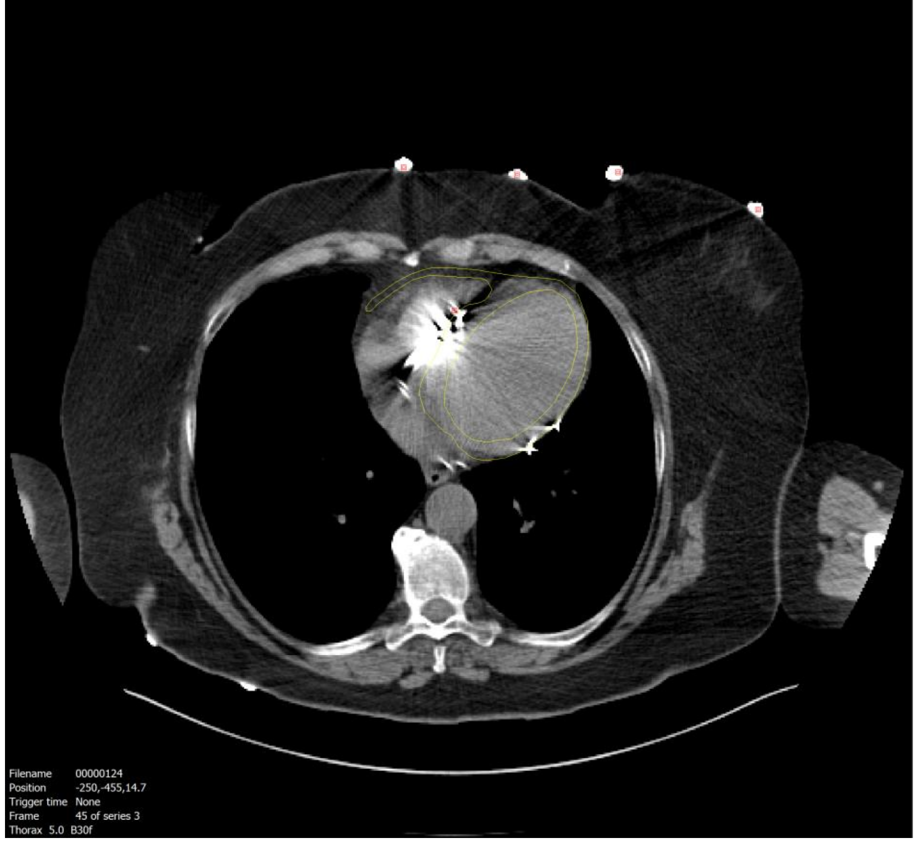


LV stim site

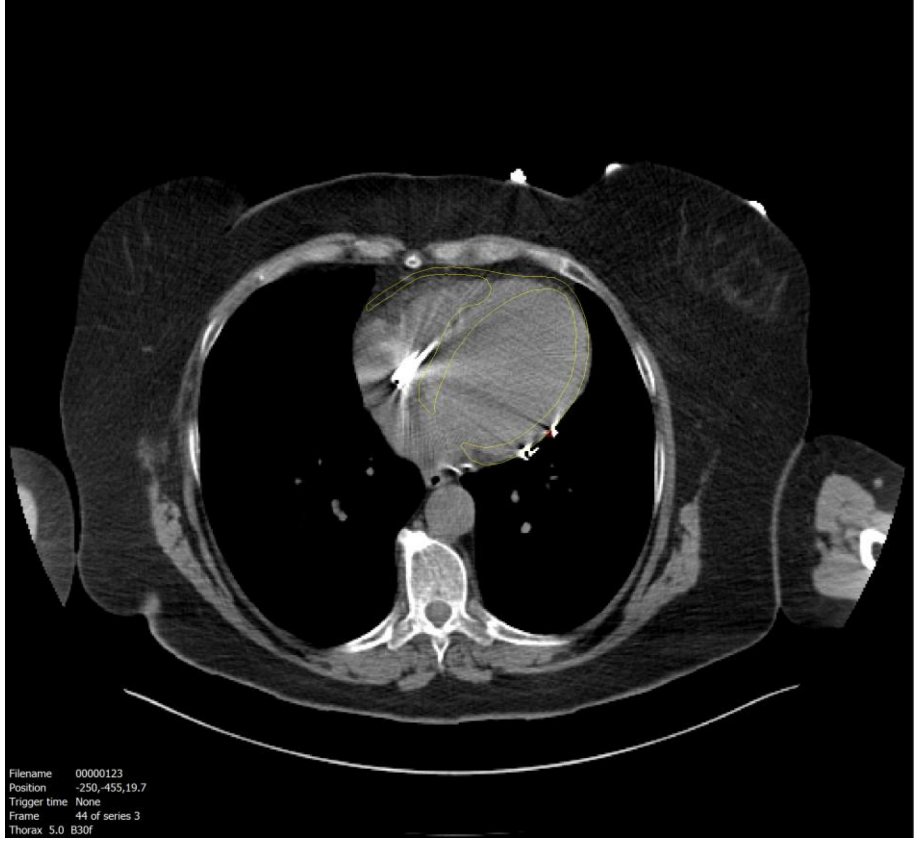


3D positions

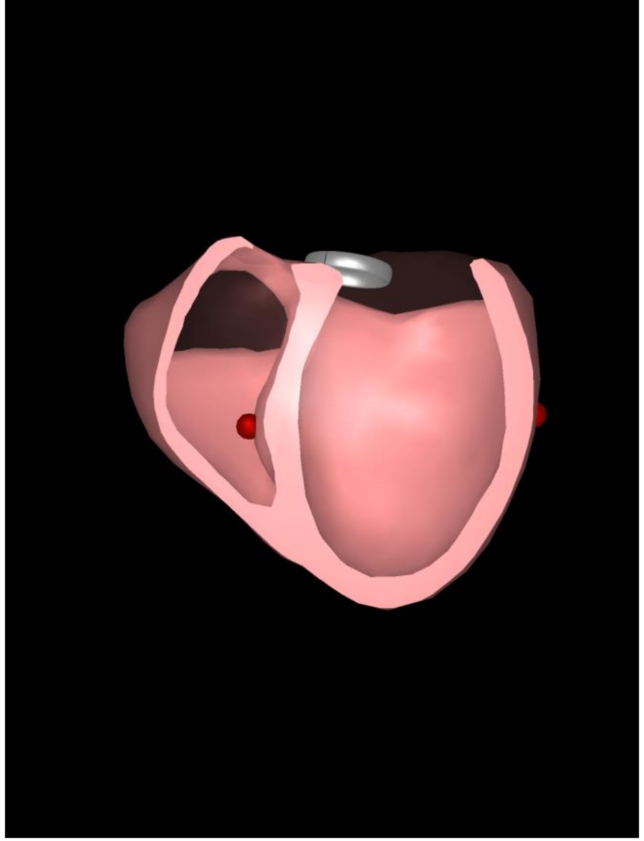

# LV STIM localization

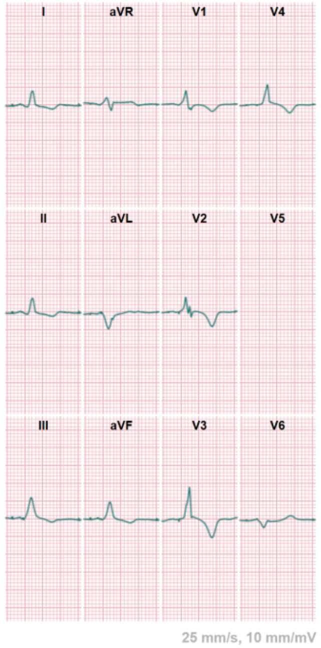


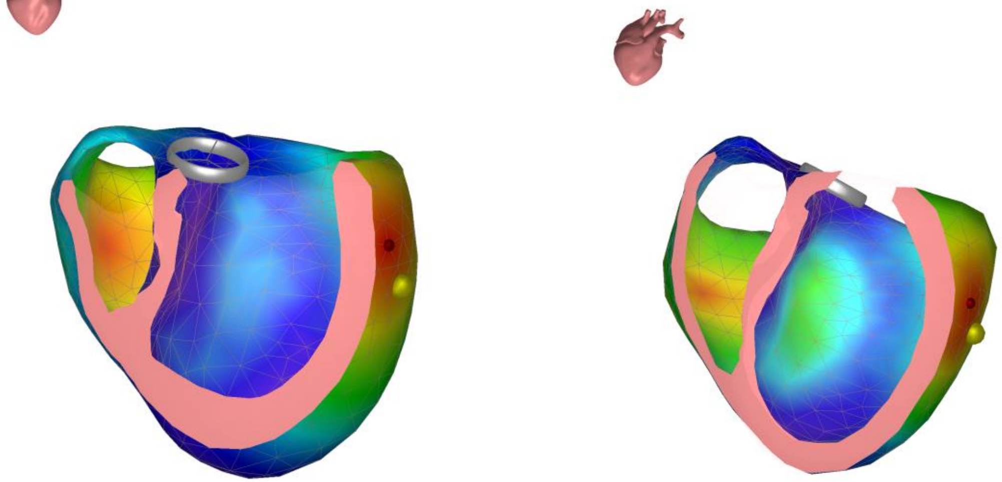

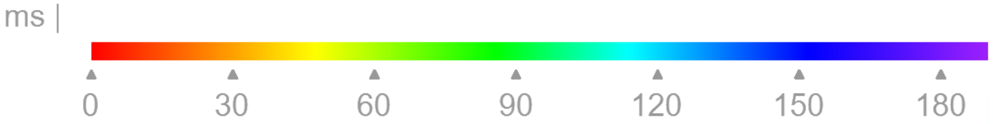


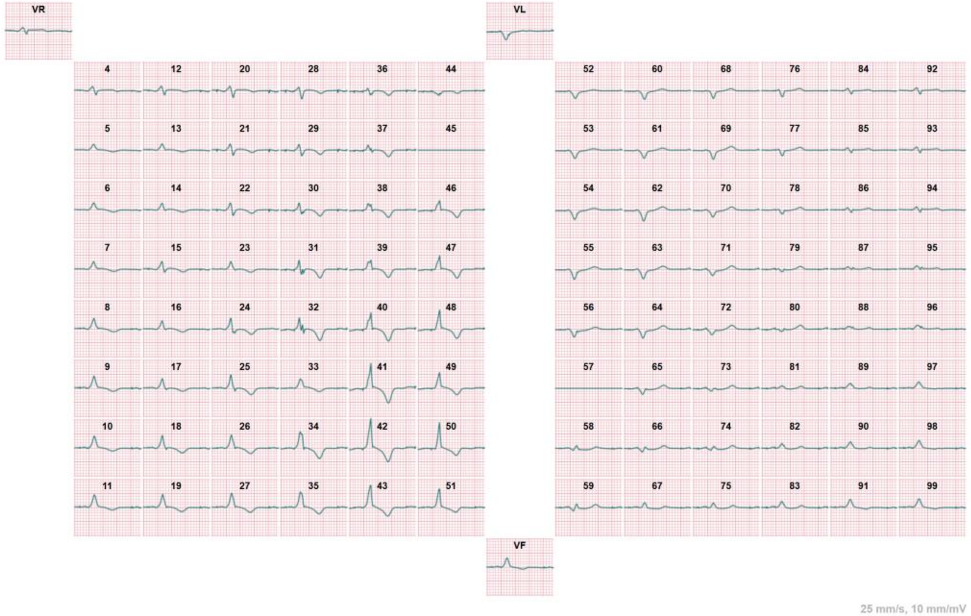


# RV STIM localization

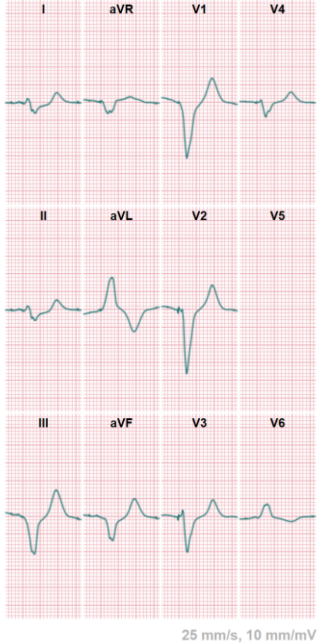


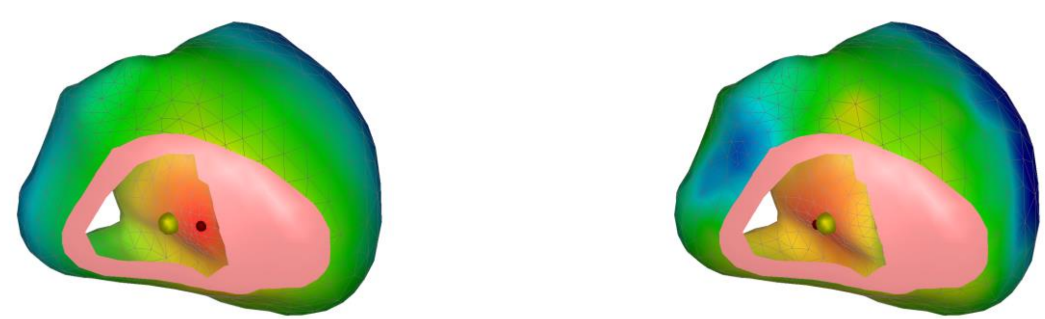

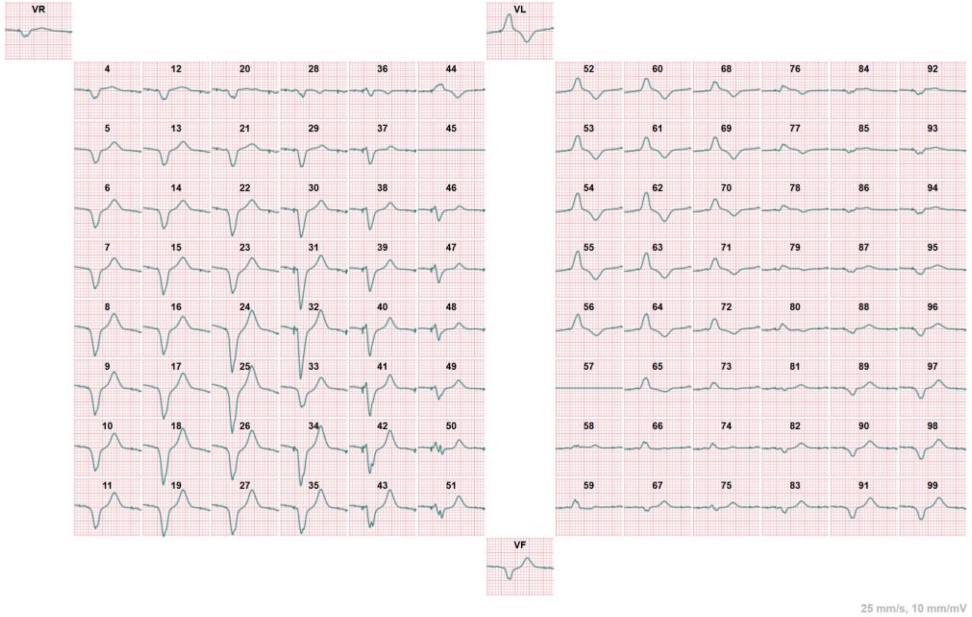

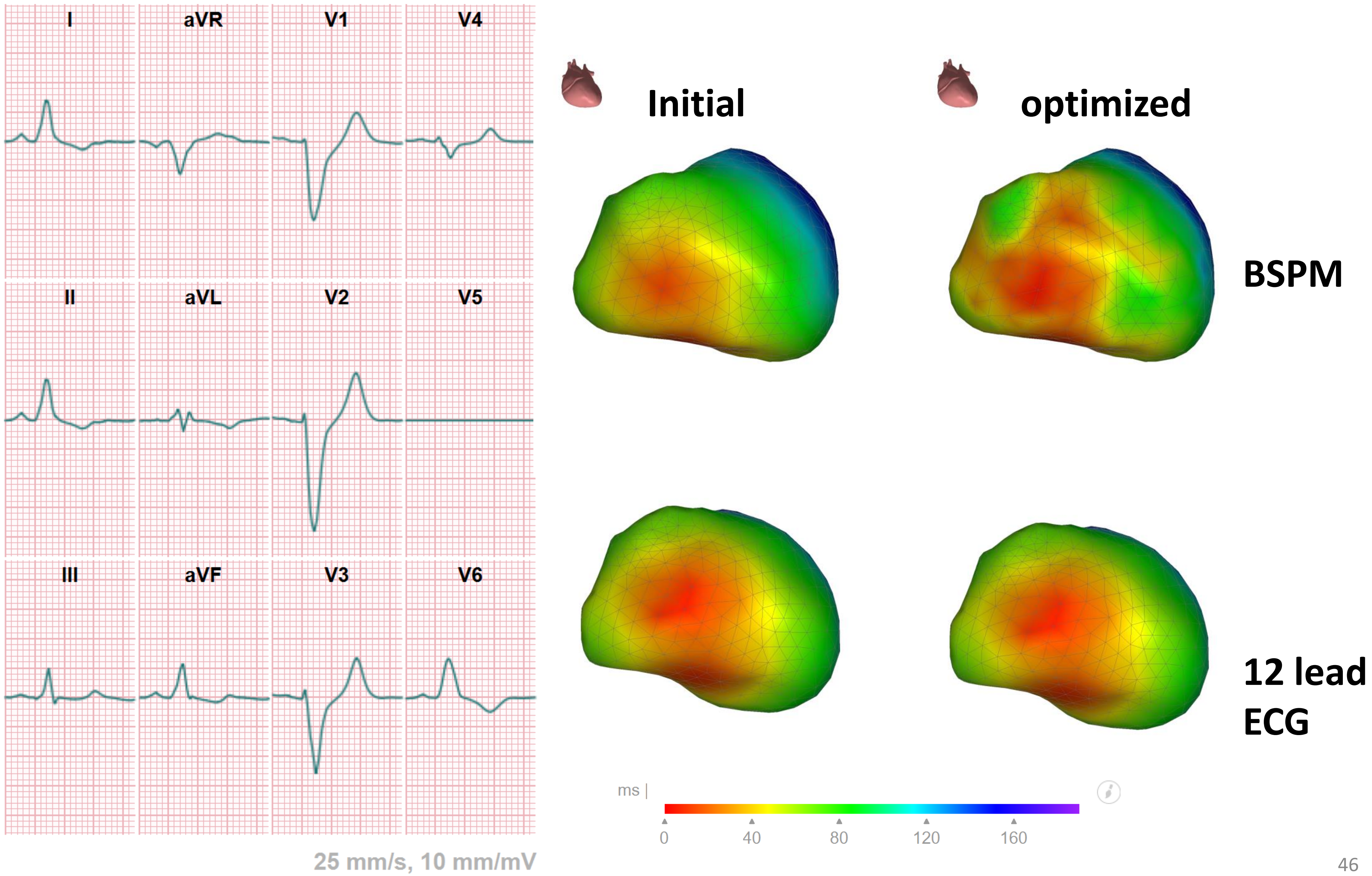
I
aVR
V1
V4
II
aVL
V2
V5
III
aVF
V3
V6
25 mm/s, 10 mm/mV
Initial
optimized
BSPM
12 lead
ECG
ms |
0
40
80
120
160

# Pat016

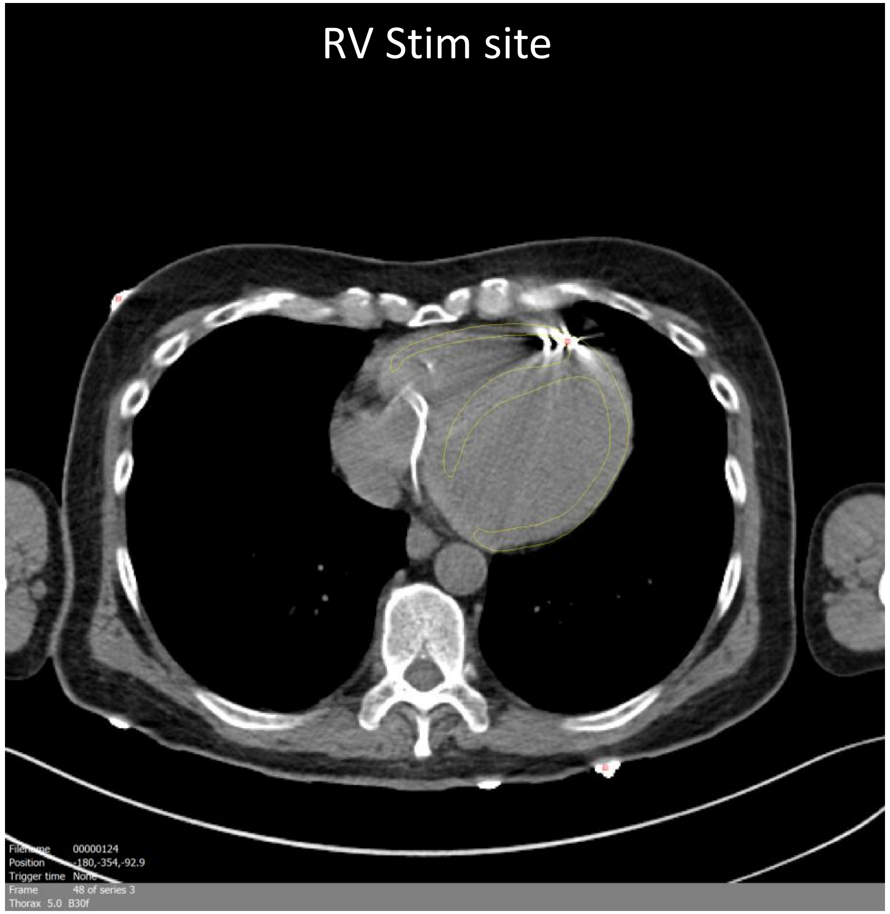
RV Stim site
Filename 00000124
Position -180,-354,-92.9
Trigger time None
Frame 48 of series 3
Thorax 5.0 B30f

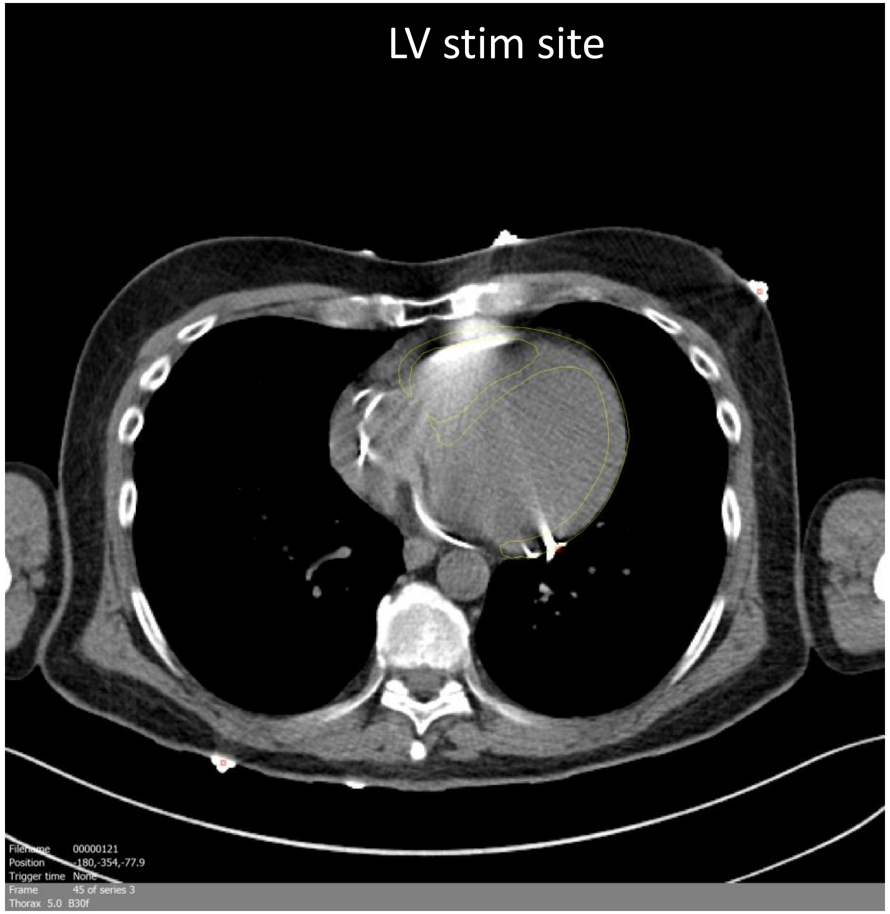
LV stim site
Filename 00000121
Position -180,-354,-77.9
Trigger time None
Frame 45 of series 3
Thorax 5.0 B30f

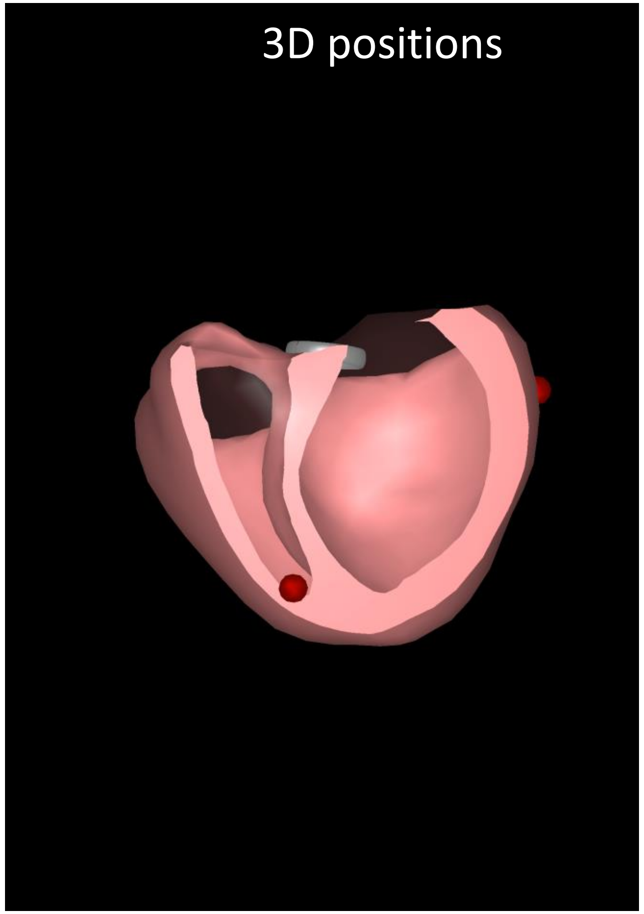
3D positions

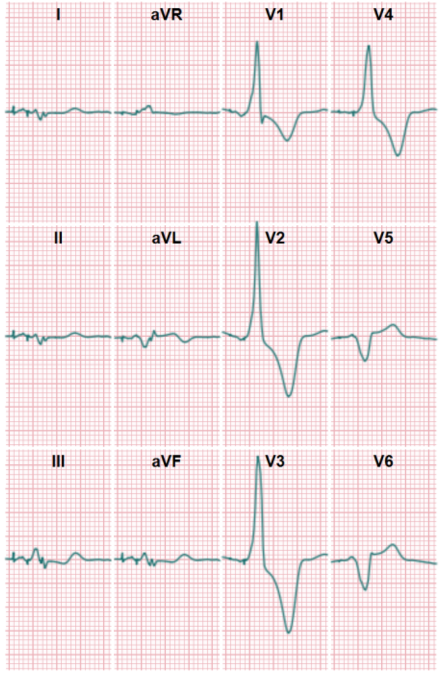


25 mm/s, 10 mm/mV

## LV STIM localizati

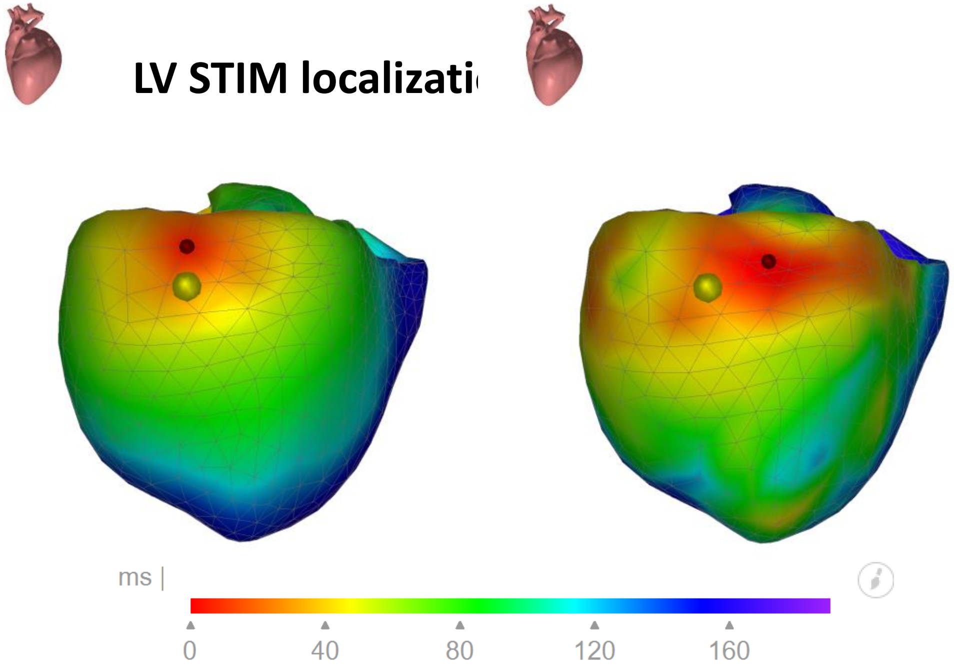


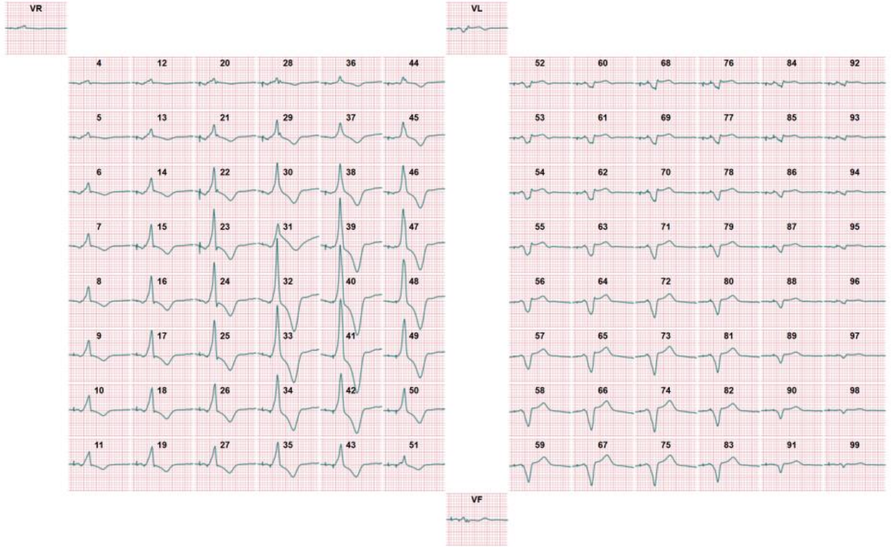

## RV STIM localization

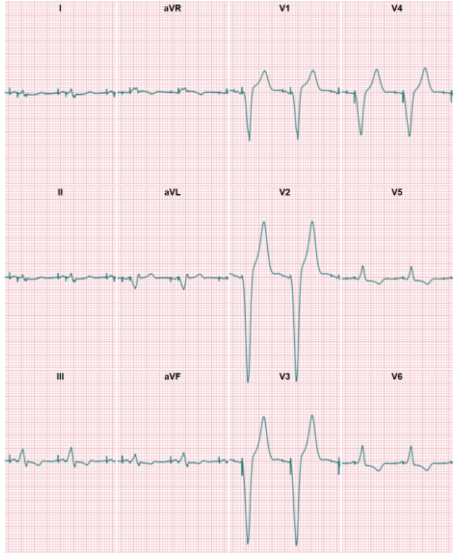


25 mm/s, 10 mm/mV

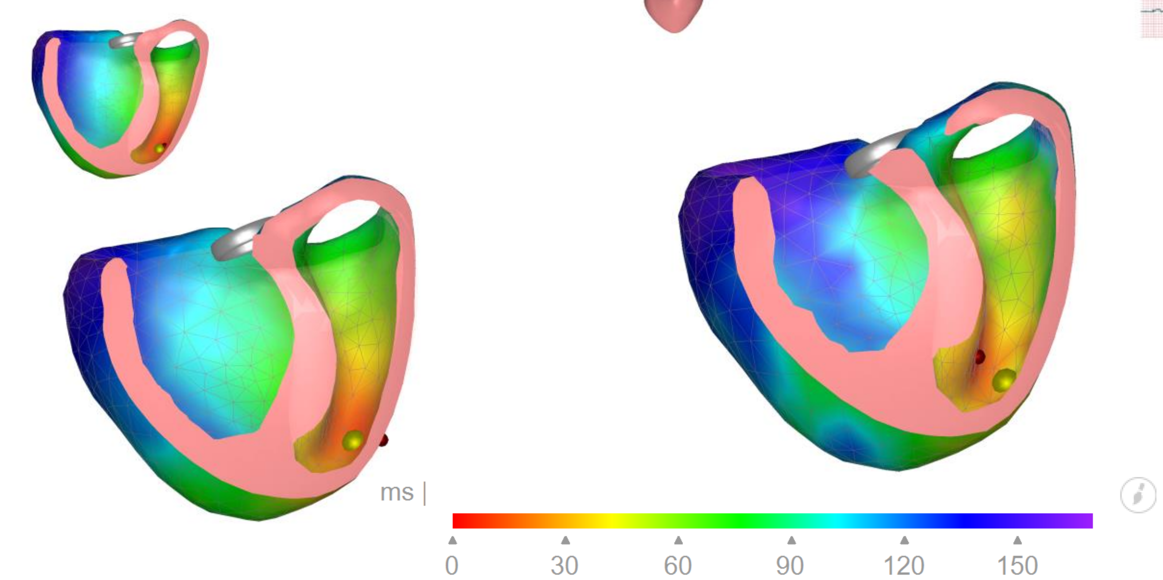


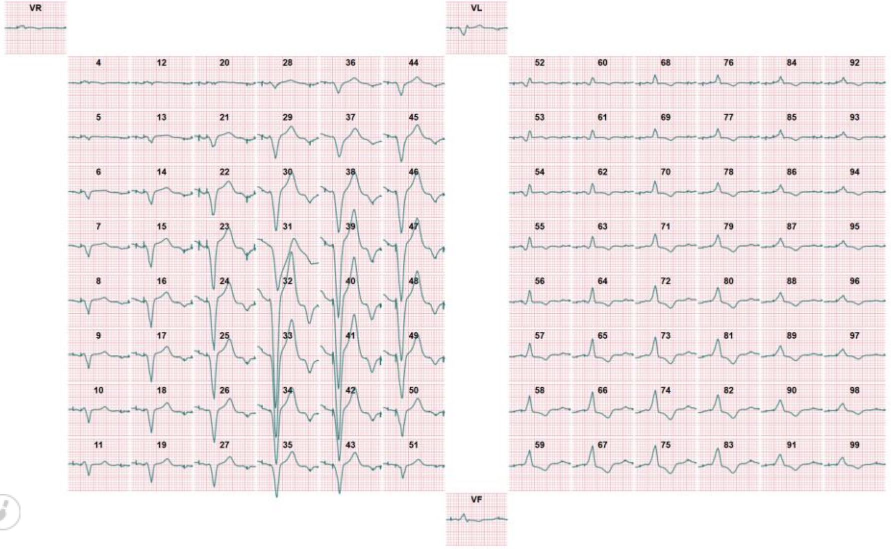

25 mm/s, 10 mm/mV

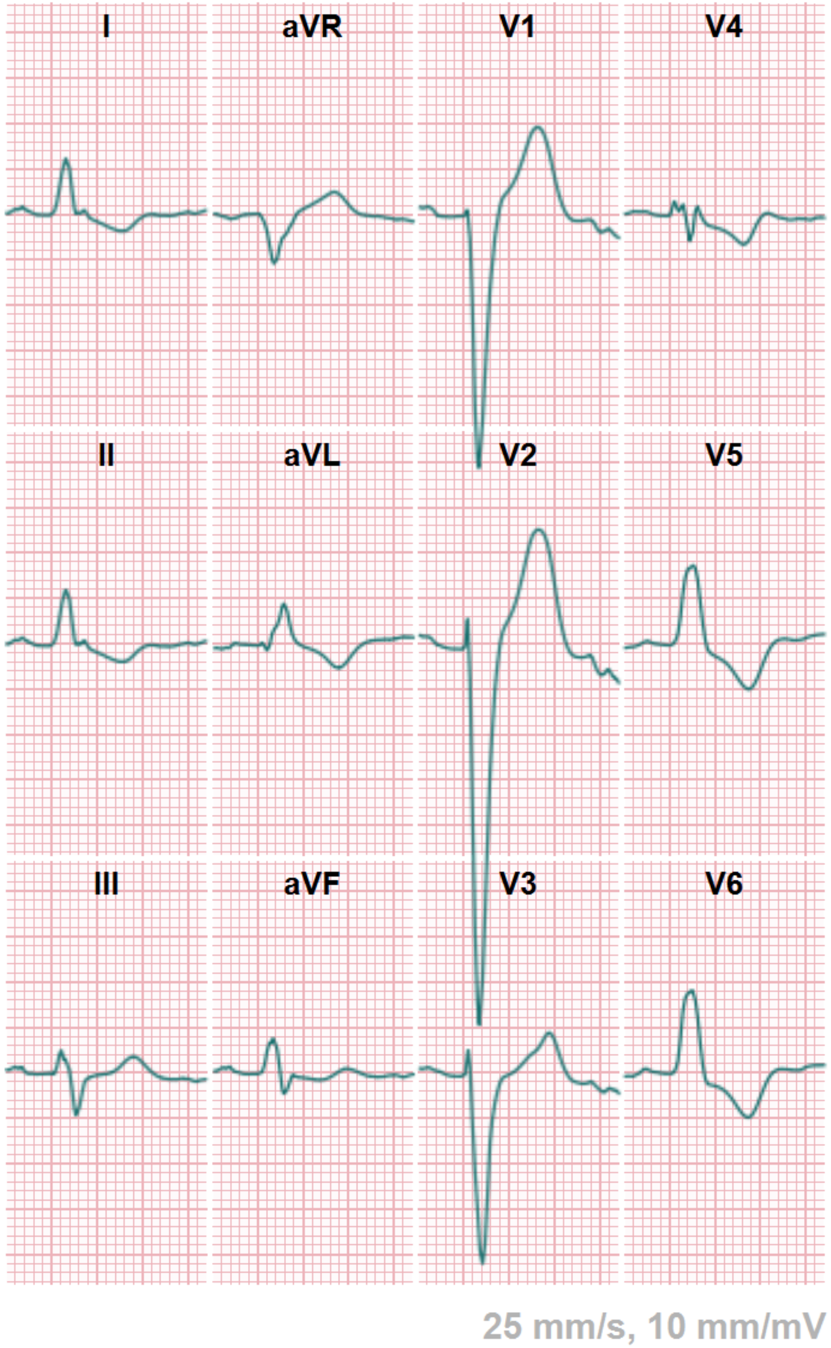
I
aVR
V1
V4
II
aVL
V2
V5
III
aVF
V3
V6
25 mm/s, 10 mm/mV

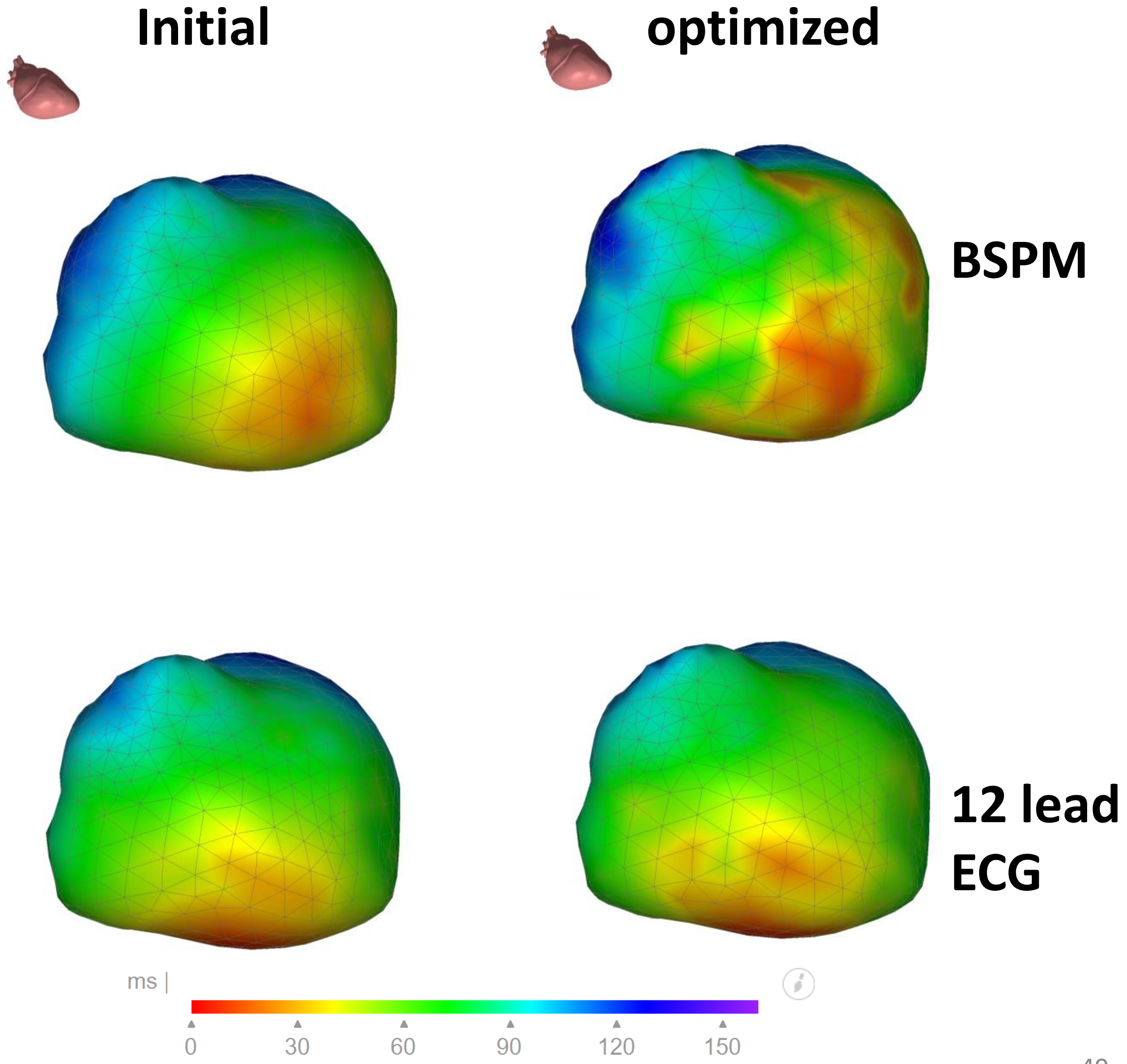
Initial
optimized
BSPM
12 lead
ECG
ms
0
30
60
90
120
150

# Pat017

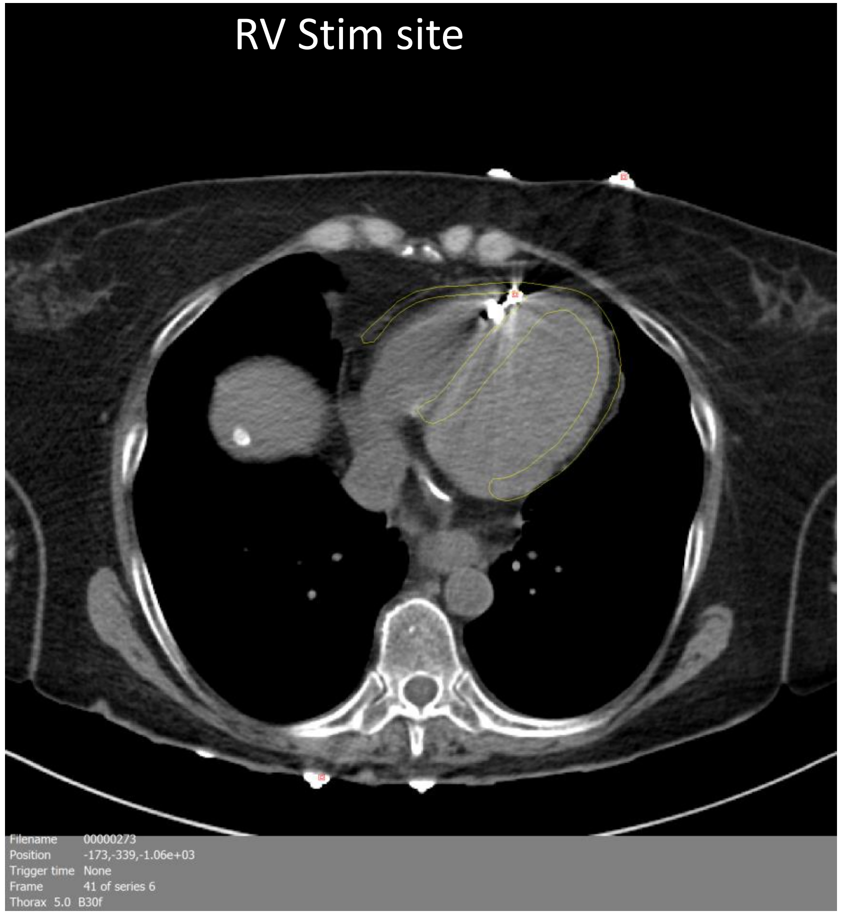
RV Stim site
Filename 00000273
Position -173,-339,-1.06e+03
Trigger time None
Frame 41 of series 6
Thorax 5.0 B30f

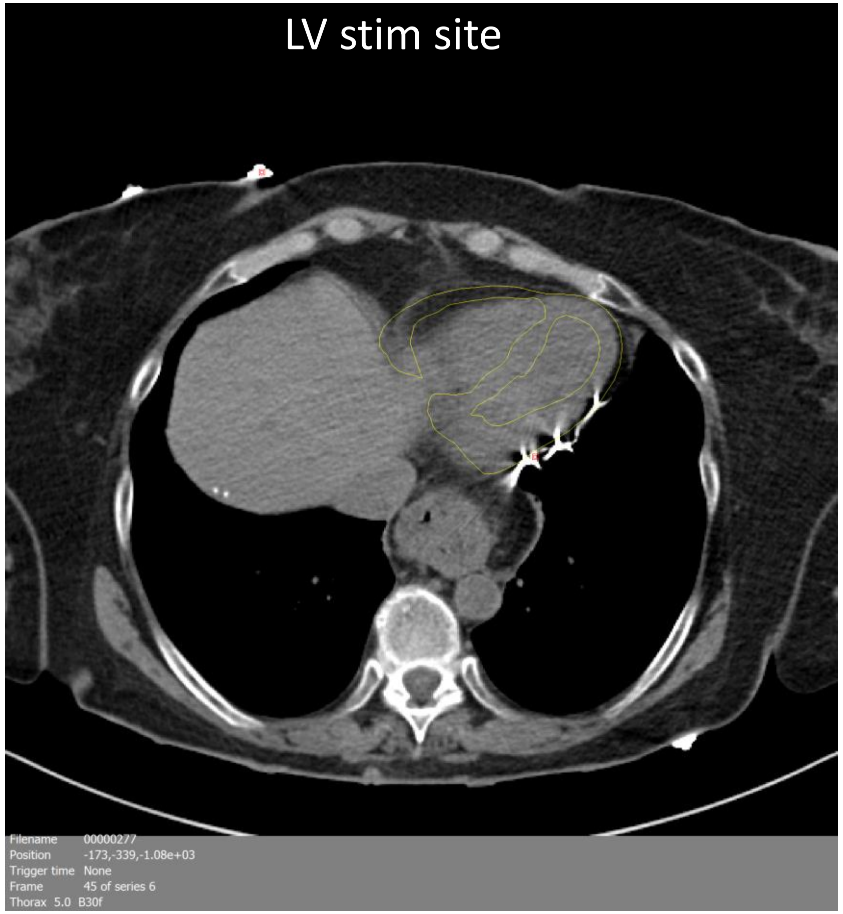
LV stim site
Filename 00000277
Position -173,-339,-1.08e+03
Trigger time None
Frame 45 of series 6
Thorax 5.0 B30f

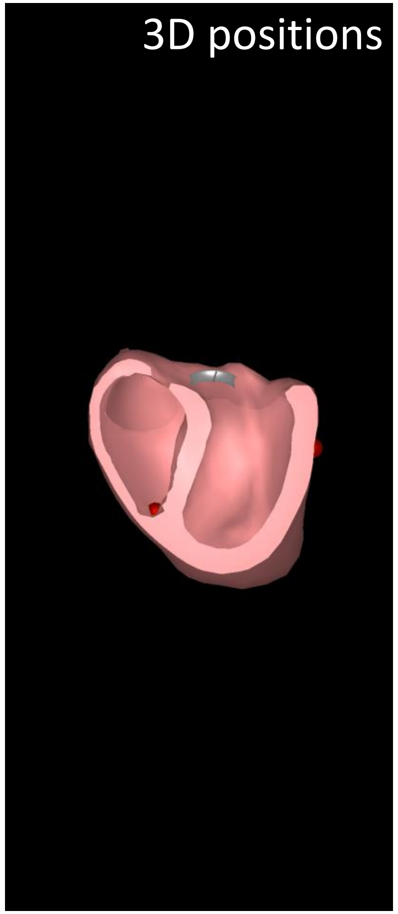
3D positions

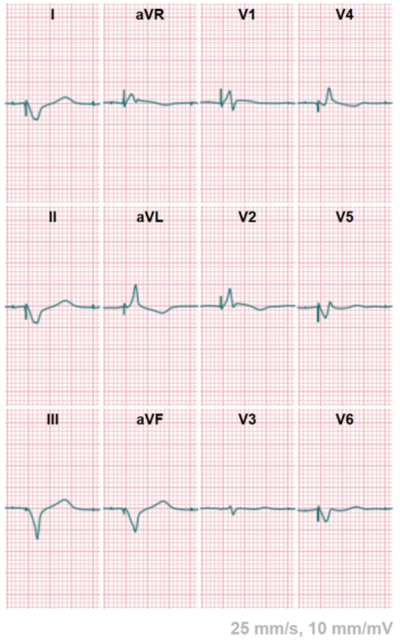


# LV STIM localization

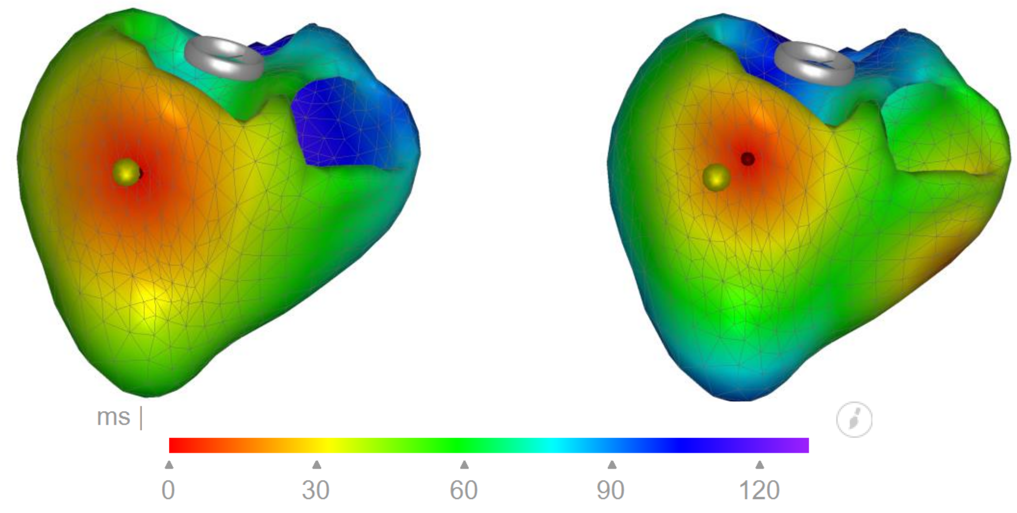


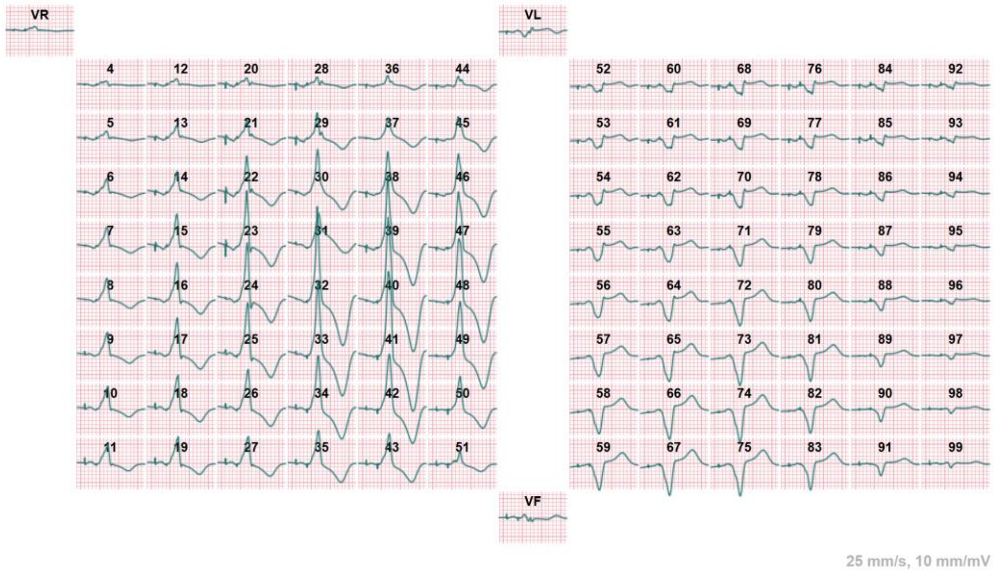


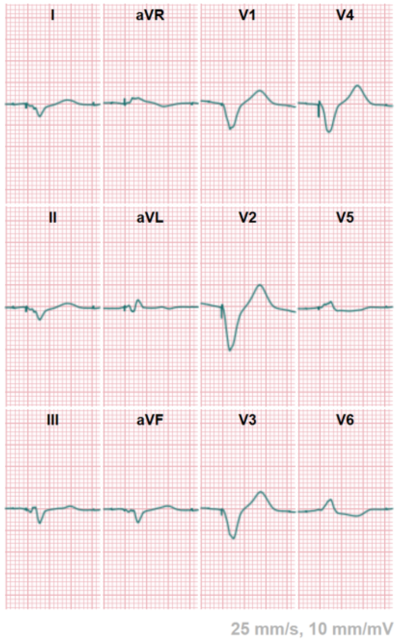


# RV STIM localization

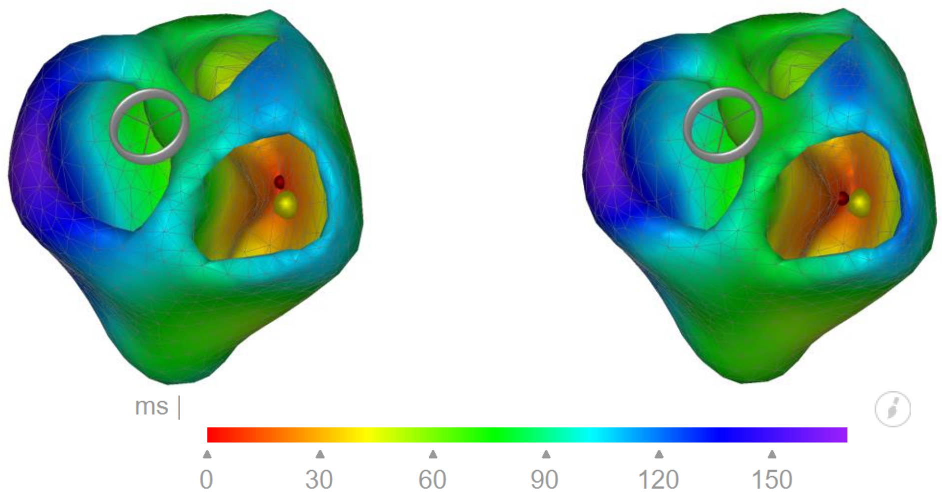


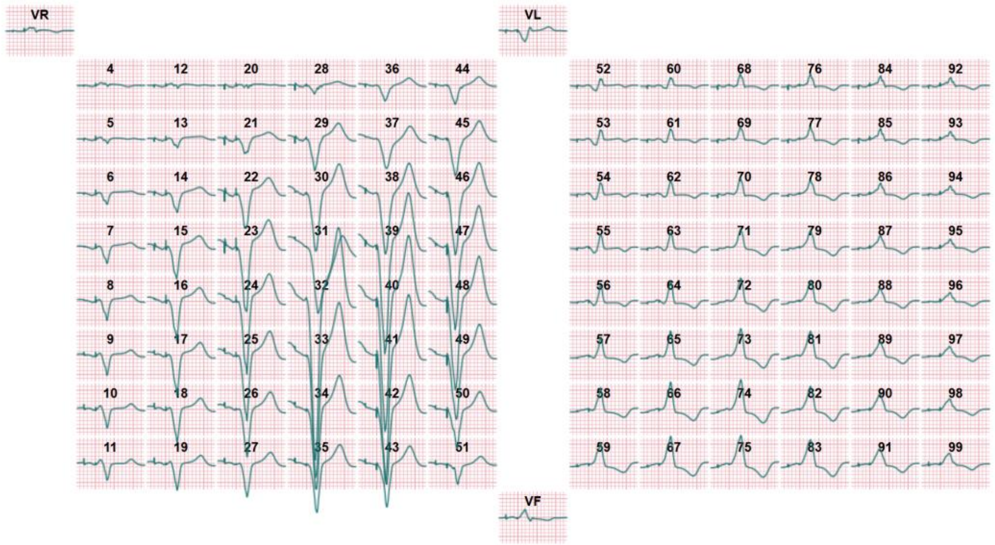

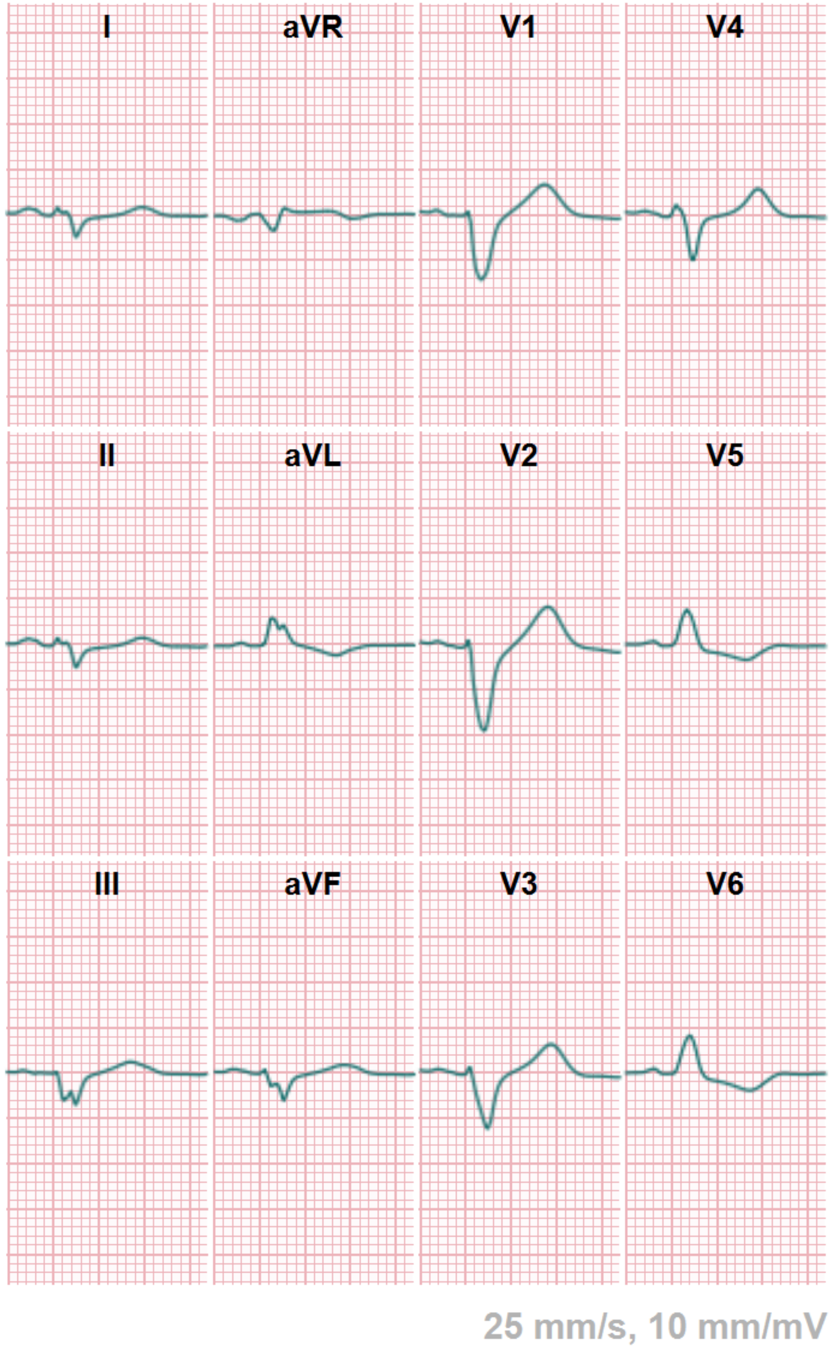
I
aVR
V1
V4
II
aVL
V2
V5
III
aVF
V3
V6
25 mm/s, 10 mm/mV

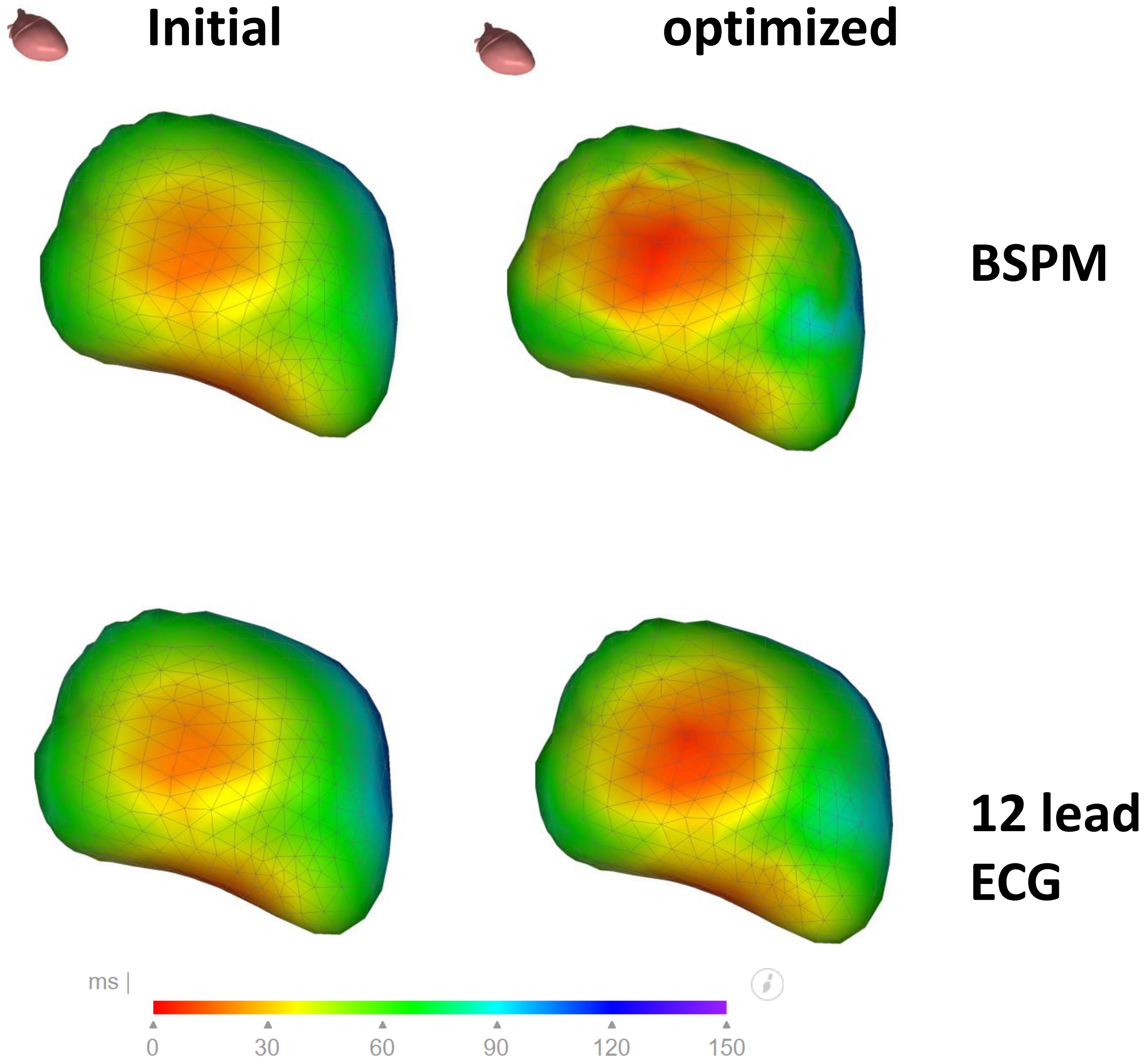
Initial
optimized
BSPM
12 lead
ECG
ms |
0
30
60
90
120
150

# Pat018

RV Stim site

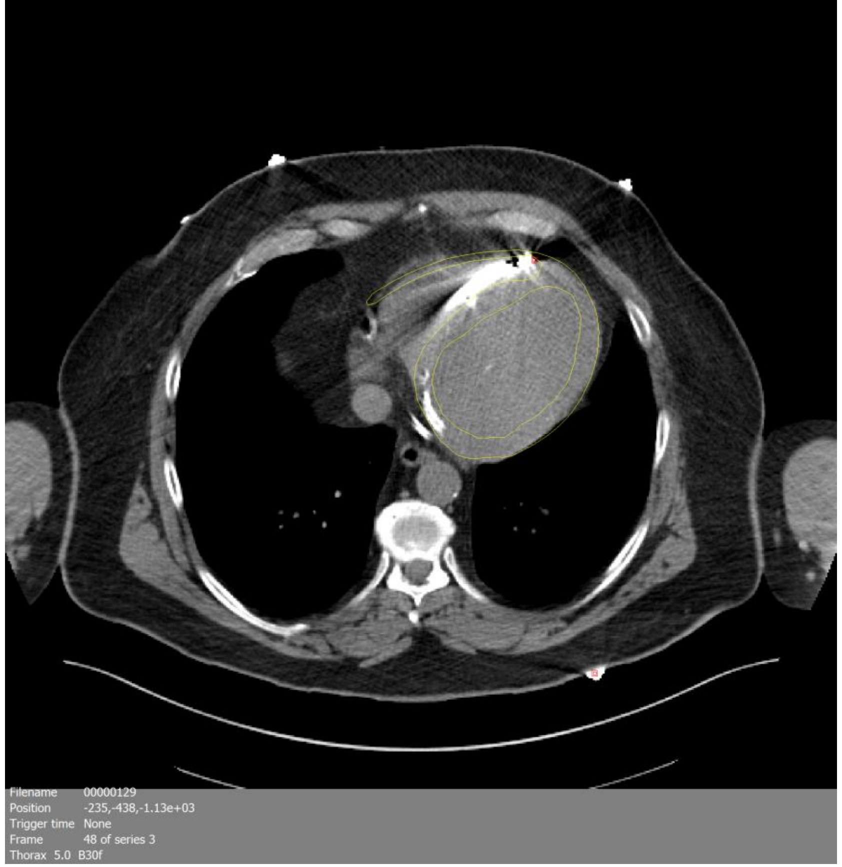


LV stim site

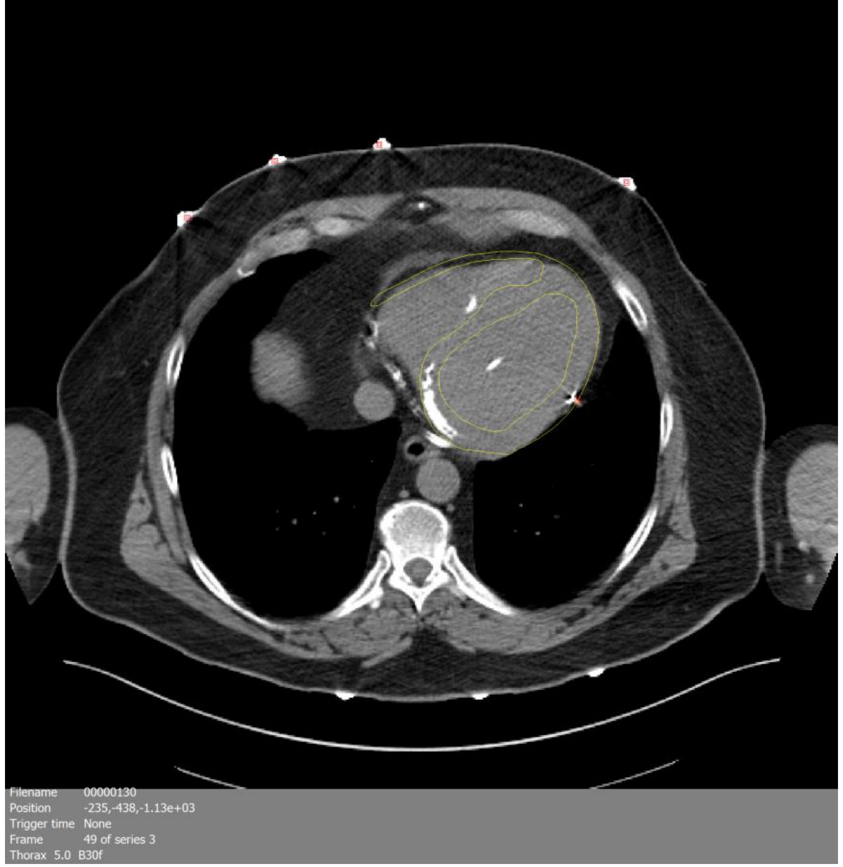


3D positions

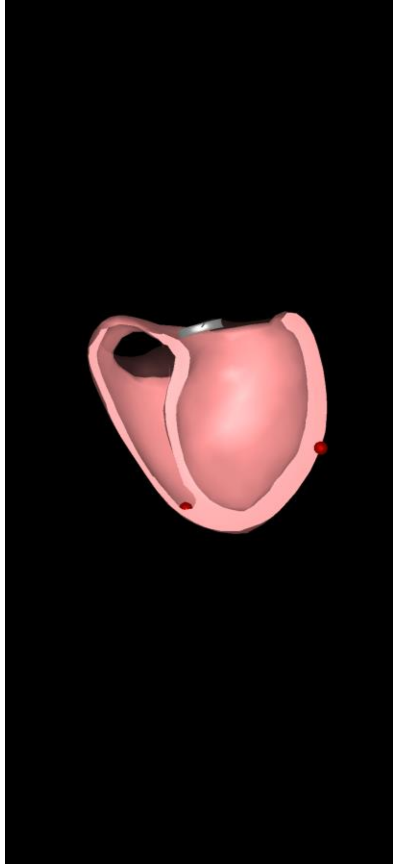

## LV STIM localization

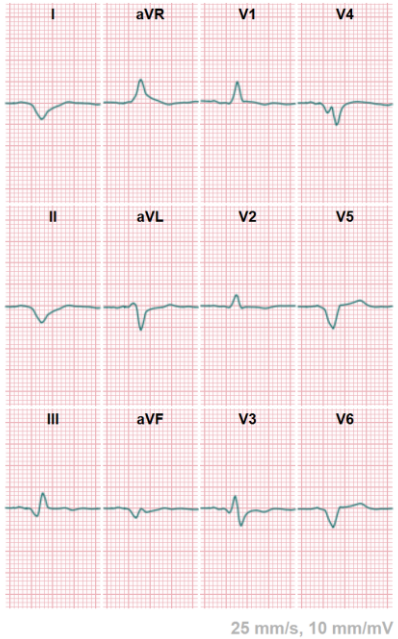


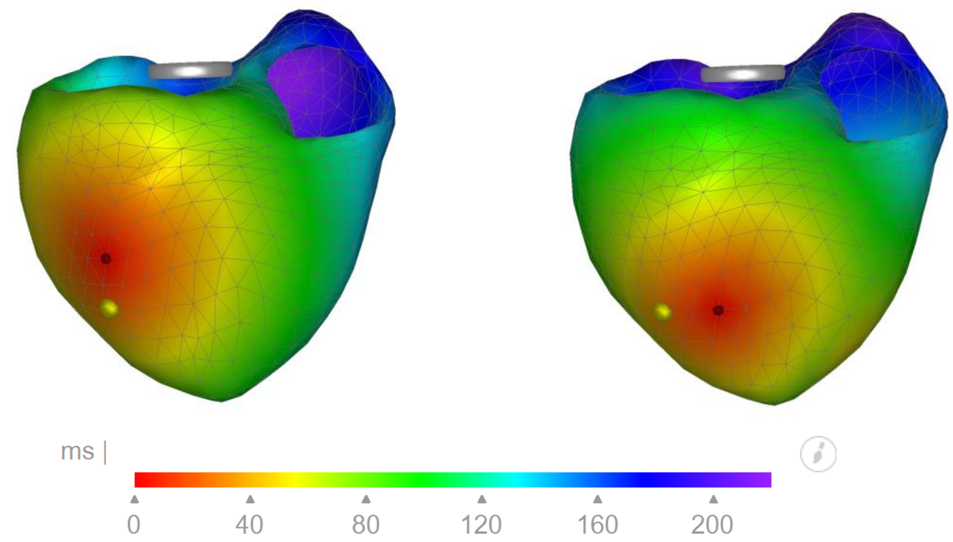


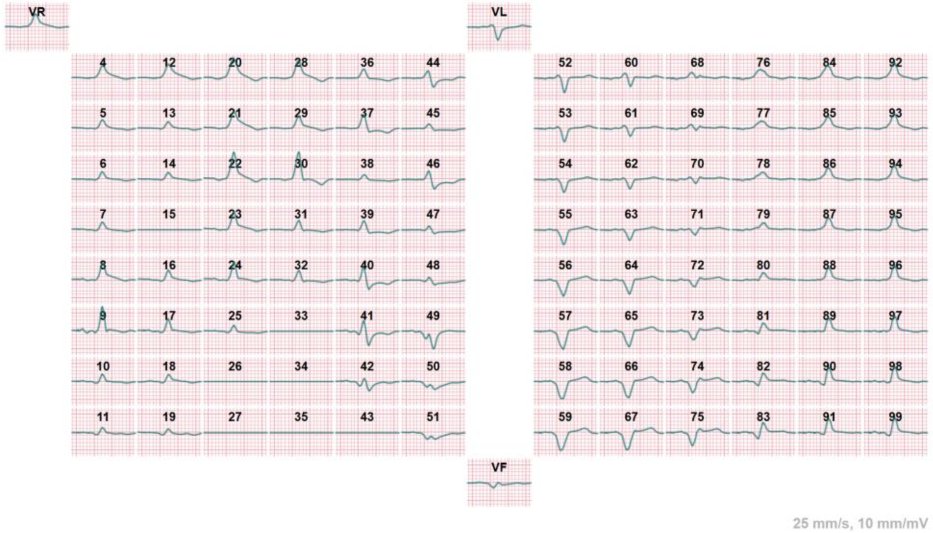


## RV STIM localization

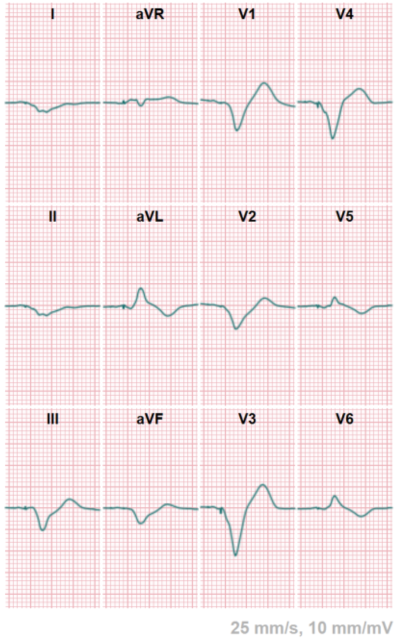


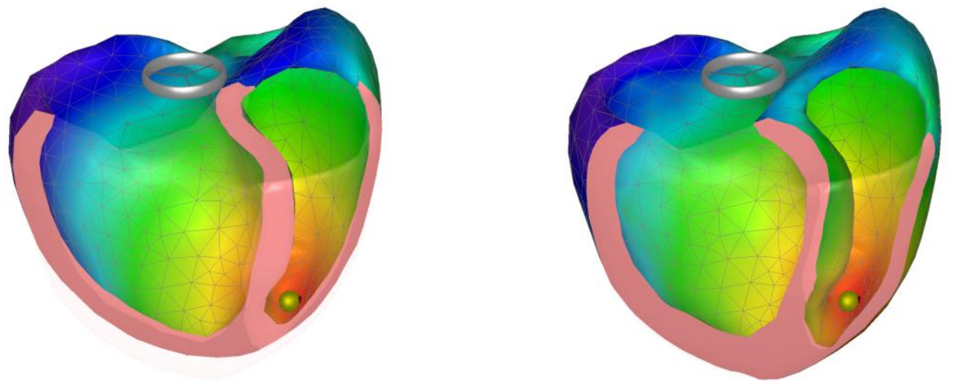

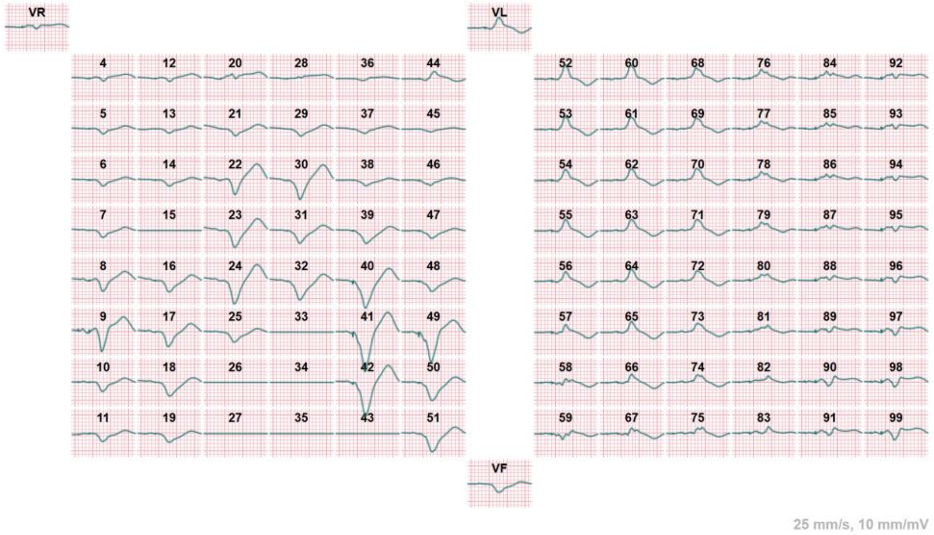

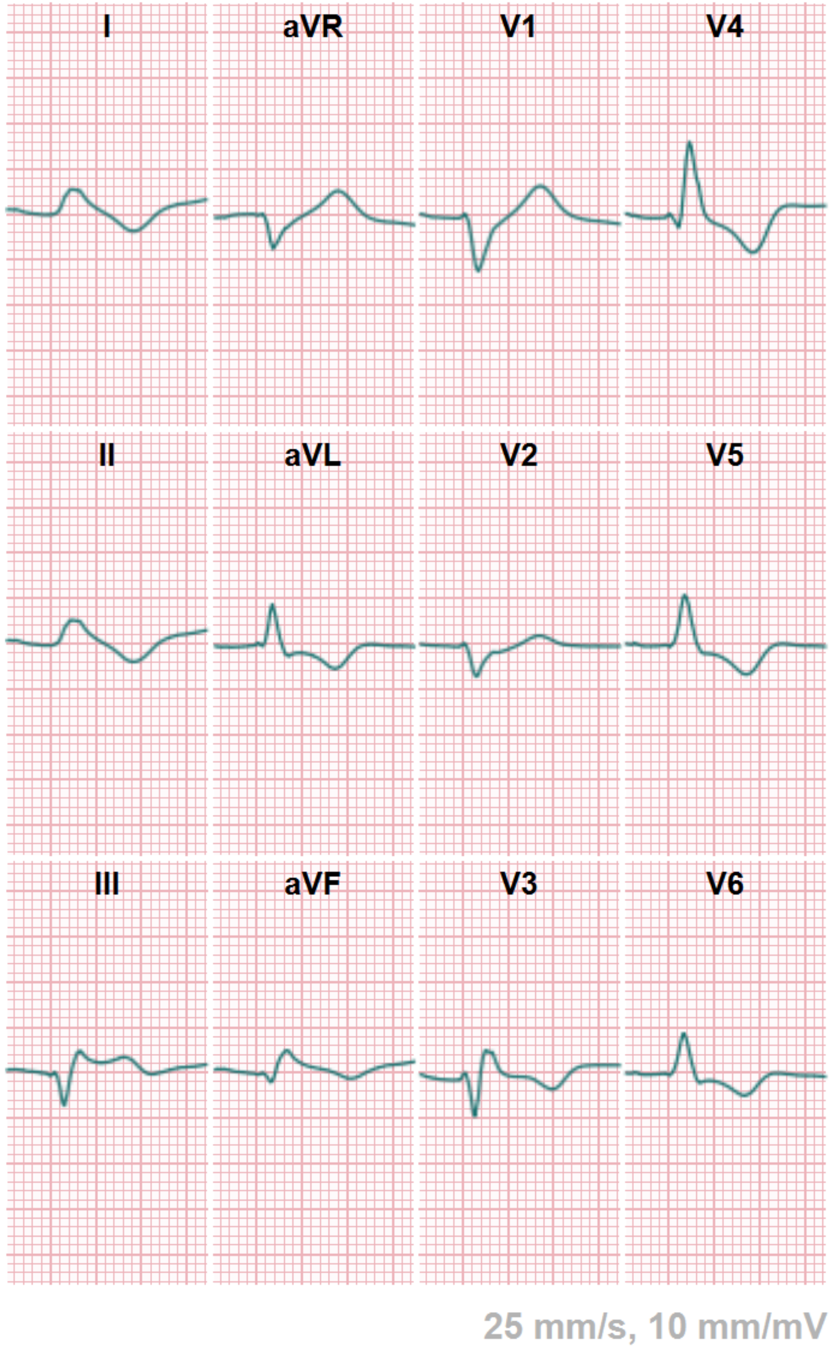


**Initial** **optimized**

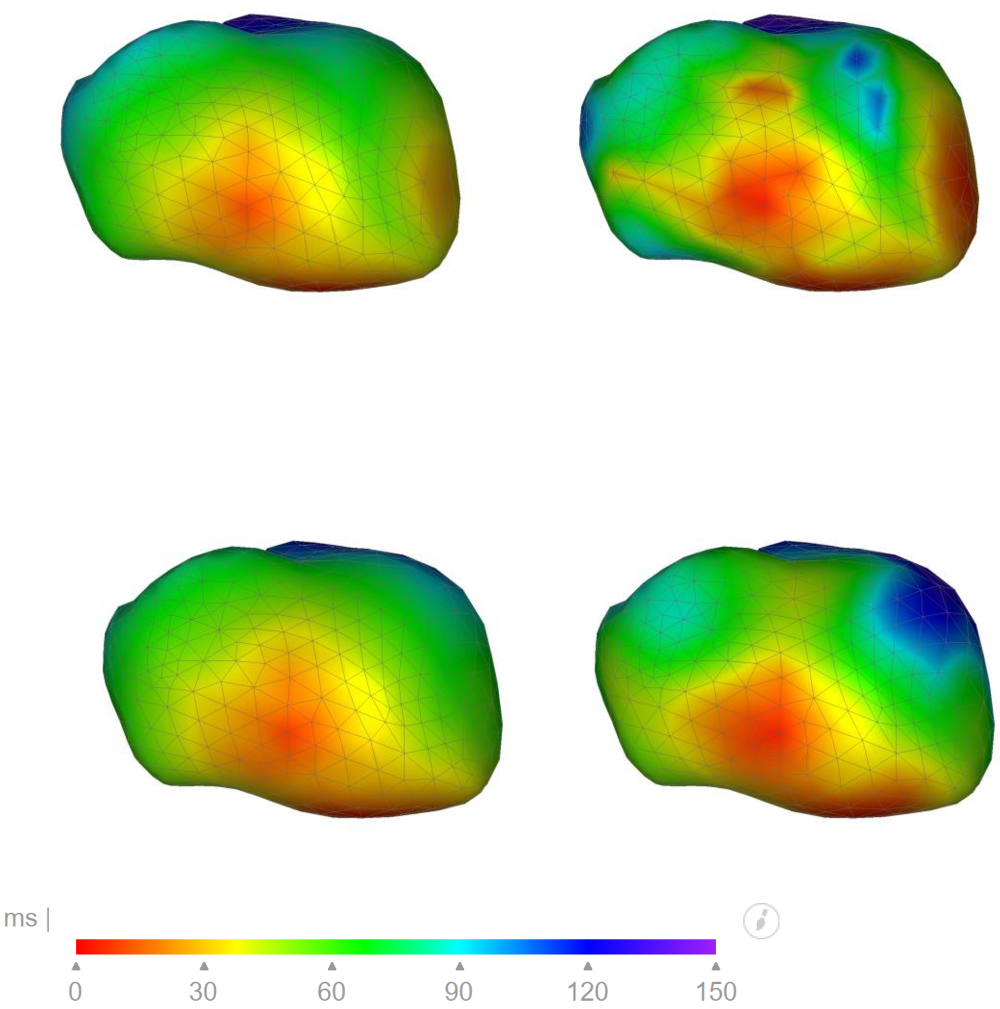


**BSPM**

**12 lead ECG**

# Pat019

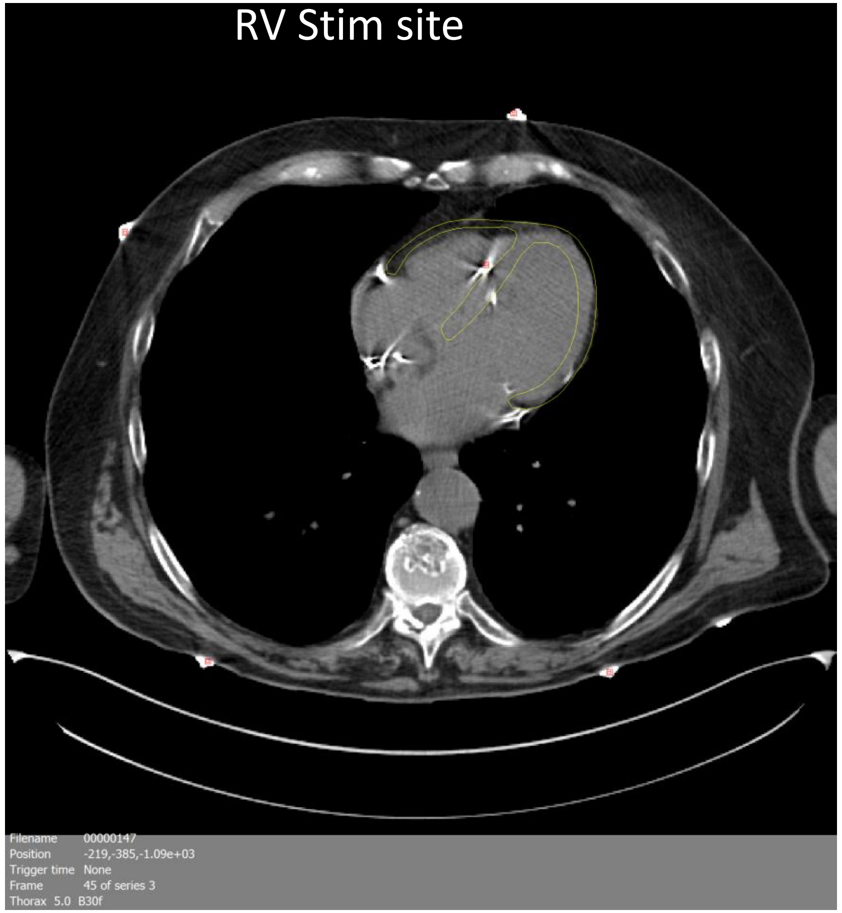
RV Stim site
Filename 00000147
Position -219,-385,-1.09e+03
Trigger time None
Frame 45 of series 3
Thorax 5.0 B30f

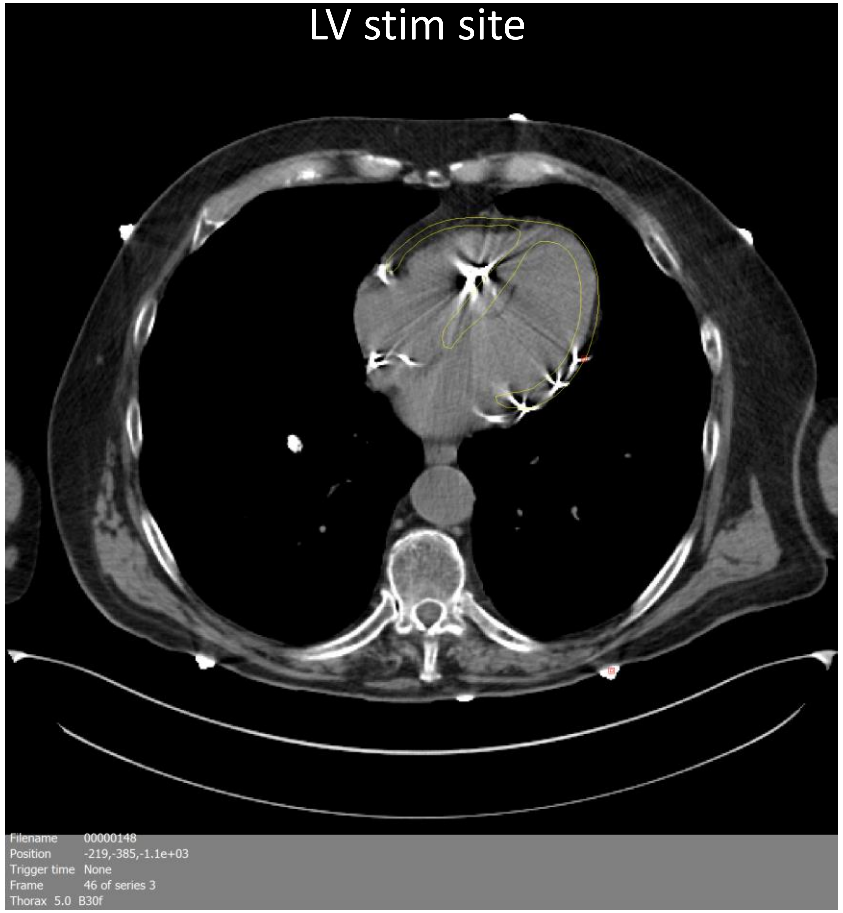
LV stim site
Filename 00000148
Position -219,-385,-1.1e+03
Trigger time None
Frame 46 of series 3
Thorax 5.0 B30f

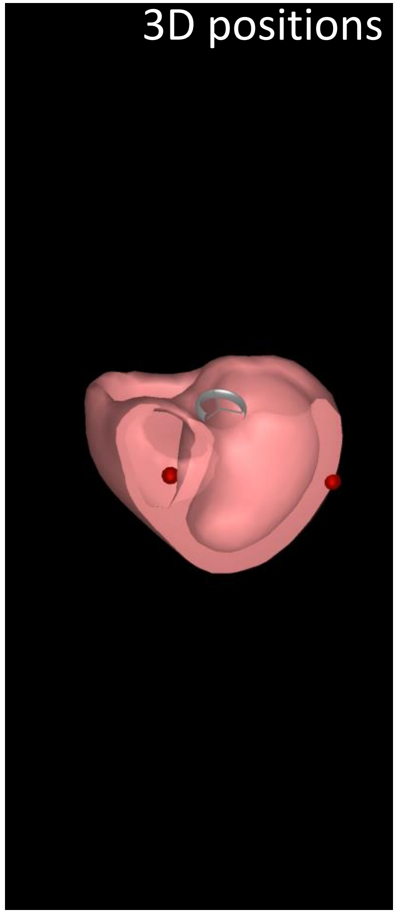
3D positions

# LV STIM localization

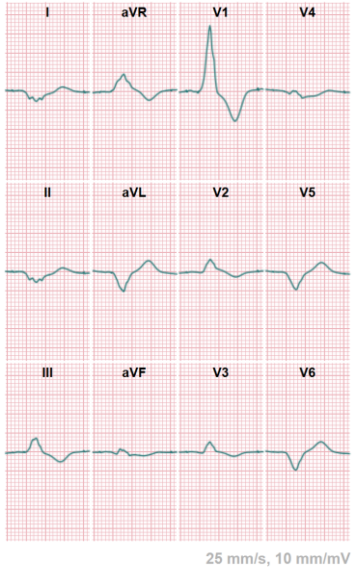


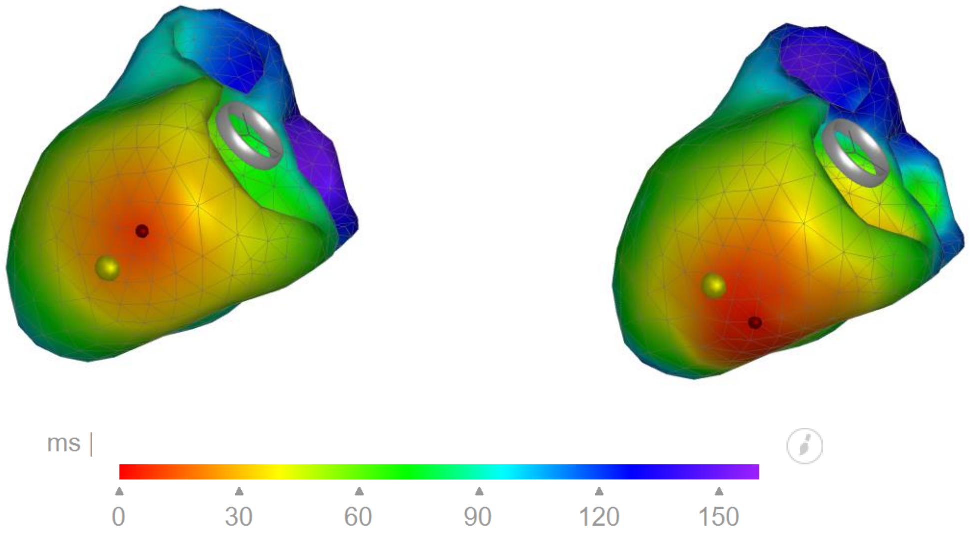


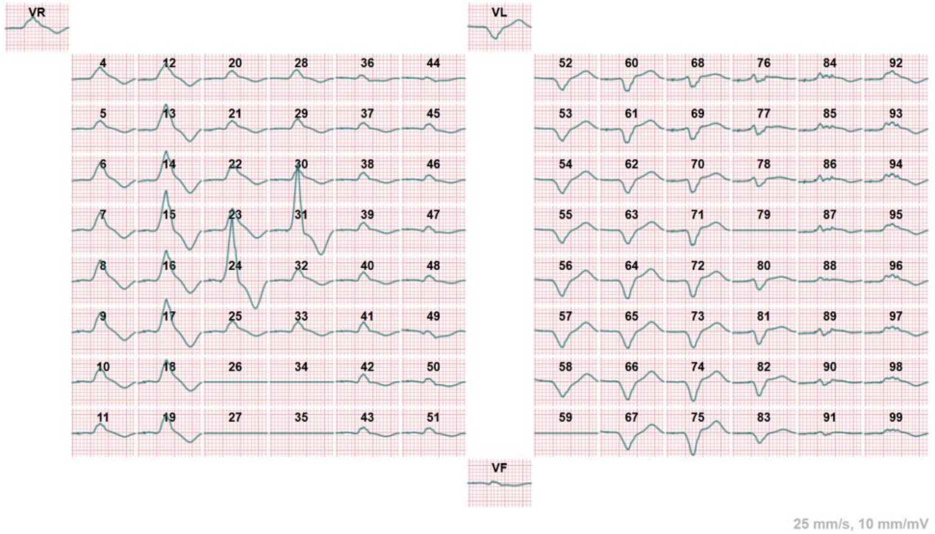


# RV STIM localization

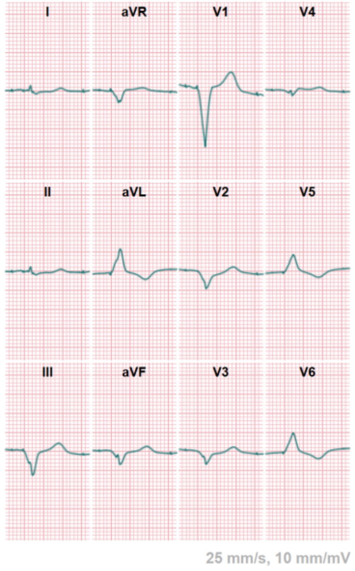


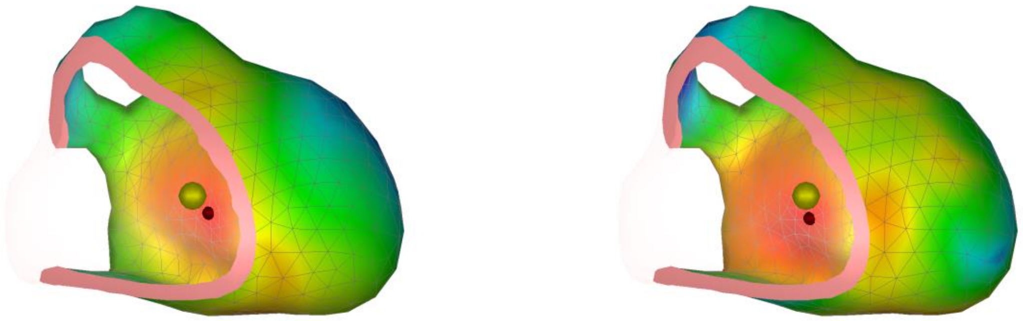

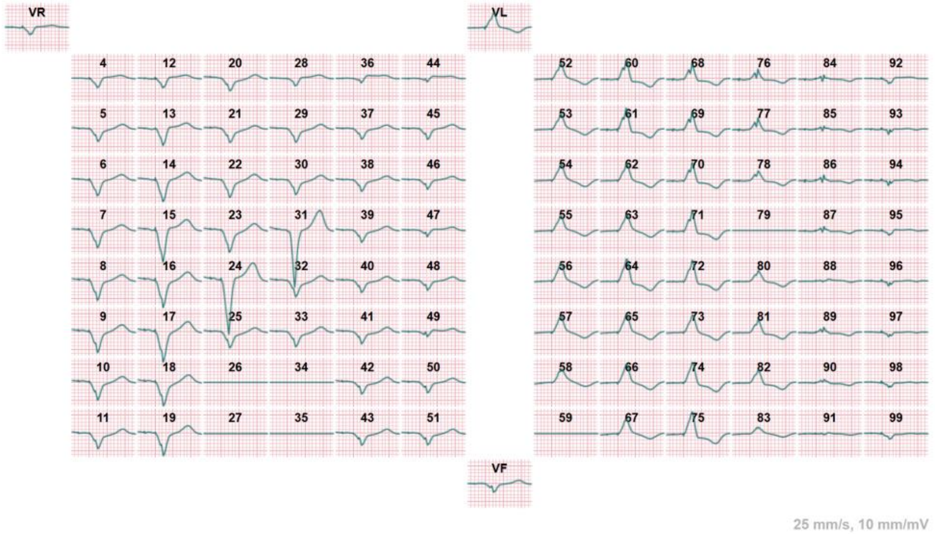

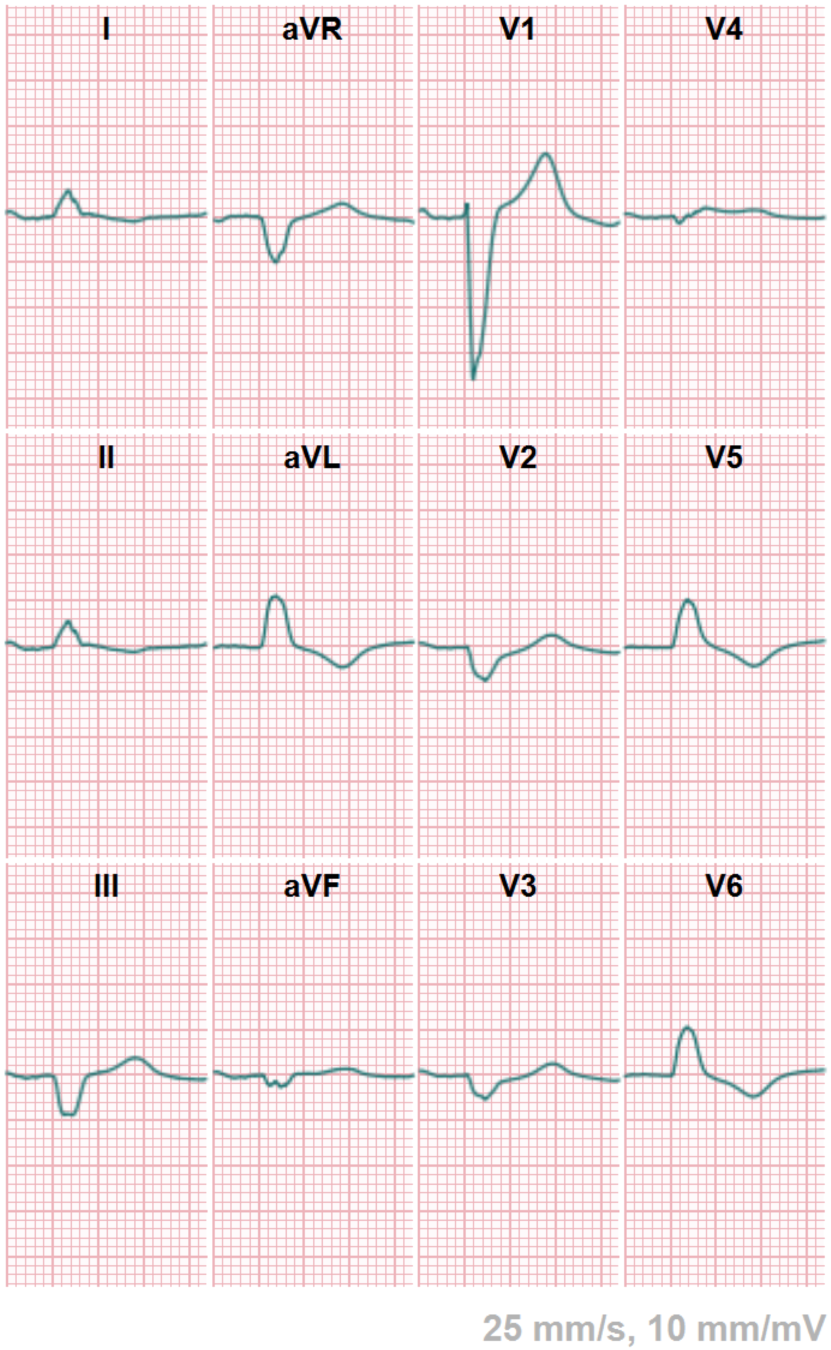
I
aVR
V1
V4
II
aVL
V2
V5
III
aVF
V3
V6
25 mm/s, 10 mm/mV

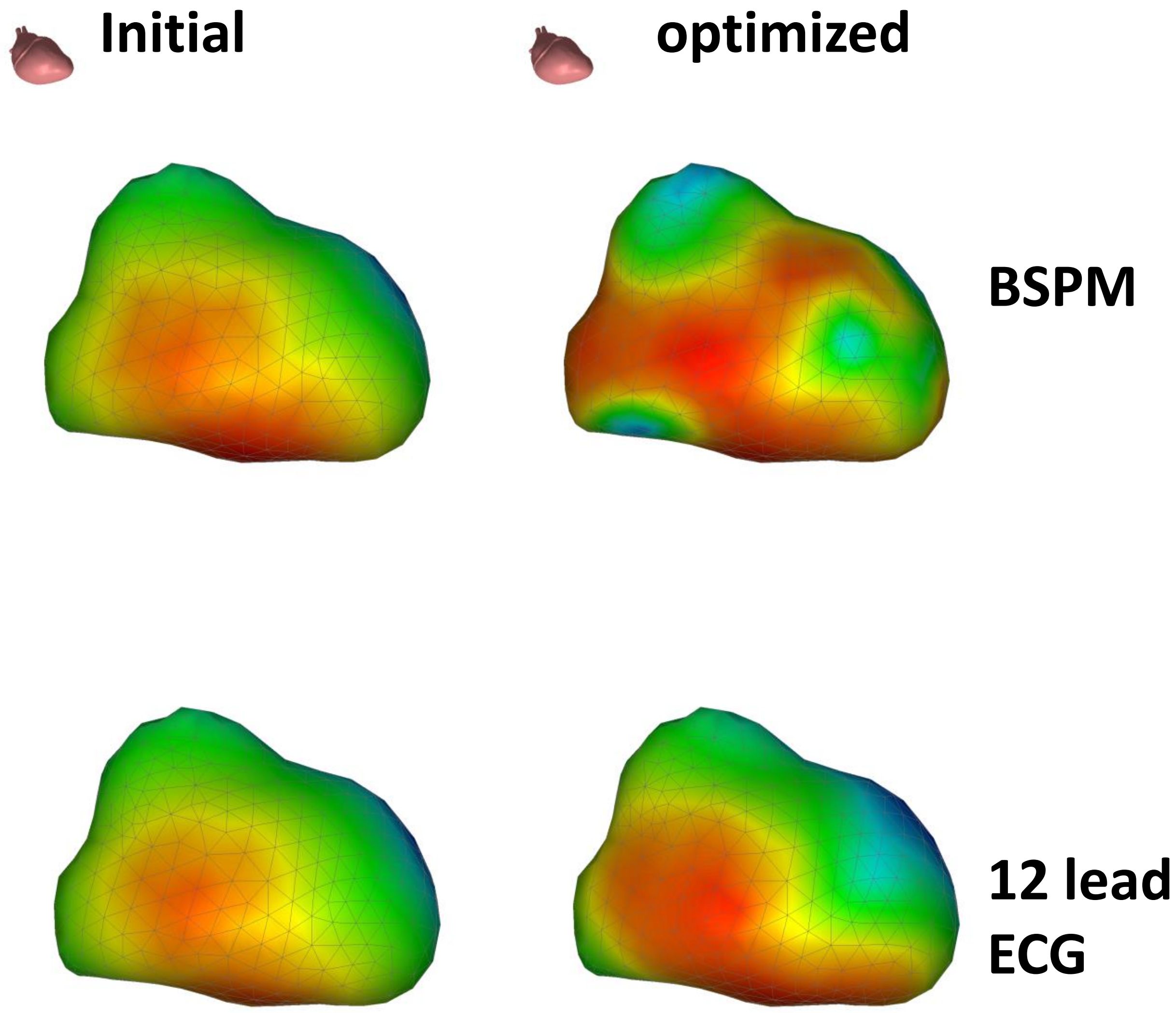
Initial
optimized
BSPM
12 lead
ECG
